# ﻿Systematic revision of the South American “*Nuncia*” (Opiliones, Laniatores, Triaenonychidae)

**DOI:** 10.3897/zookeys.1207.120068

**Published:** 2024-07-12

**Authors:** Willians Porto, Shahan Derkarabetian, Gonzalo Giribet, Abel Pérez-González

**Affiliations:** 1 División Aracnología, Museo Argentino de Ciencias Naturales–CONICET, Av. Ángel Gallardo 470, C1405DJR Buenos Aires, Argentina Museo Argentino de Ciencias Naturales–CONICET Buenos Aires Argentina; 2 Sección Aracnología y Miriapodología, Universidad Nacional de La Plata, Facultad de Ciencias Naturales y Museo, Paseo del Bosque s/n, (1900) La Plata, Argentina Universidad Nacional de La Plata La Plata Argentina; 3 Museum of Comparative Zoology, Department of Organismic and Evolutionary Biology, Harvard University, Cambridge, MA 02138, USA Harvard University Cambridge United States of America; 4 San Diego Natural History Museum, Department of Entomology, San Diego, CA 92101, USA San Diego Natural History Museum San Diego United States of America

**Keywords:** Argentina, Chile, genital morphology, harvestmen, Insidiatores, phylogenetic analysis, systematics, taxonomy

## Abstract

The genus *Nuncia* has long been the most speciose within the Opiliones family Triaenonychidae, comprising 63 species and subspecies distributed across New Zealand and South America. Recent molecular studies utilizing Sanger sequencing and ultraconserved elements (UCEs) have indicated that this genus is not monophyletic, and true *Nuncia* are actually confined to New Zealand. Here, the morphology of all South American triaenonychids is re-examined and DNA sequence data compiled from three markers (18S rRNA, 28S rRNA and cytochrome *c* oxidase subunit I) for a large number of triaenonychid species, including specimens from all areas with species currently and formerly classified in *Nuncia* to reassess their phylogenetic position. Based on our findings we 1) revalidate the genus *Chilenuncia* (Muñoz-Cuevas, 1971) **nom. rest.**; 2) describe five new genera: *Fresiax***gen. nov.**, *Mistralia***gen. nov.**, *Laftrachia***gen. nov.**, *Lautaria***gen. nov.**, *Nerudiella***gen. nov.**; 3) redescribe five species: *Fresiaxspinulosa***comb. nov.**, *Mistraliaverrucosa***comb. nov.**, *Chilenunciachilensis***comb. nov.**, *Chilenunciarostrata***comb. nov.**, *Nerudiellaamericana***comb. nov.**; and 4) describe 22 new species of South American triaenonychids: *Fresiaxconica***sp. nov.**, *Fresiaxfray***sp. nov.**, *Fresiaxmauryi***sp. nov.**, *Fresiaxpichicuy***sp. nov.**, *Mistraliaramirezi***sp. nov.**, *Laftrachiarobin***sp. nov.**, *Lautariaceachei***sp. nov.**, *Nerudiellacachai***sp. nov.**, *Nerudiellacaramavida***sp. nov.**, *Nerudiellacautin***sp. nov.**, *Nerudiellachoapa***sp. nov.**, *Nerudiellacuri***sp. nov.**, *Nerudiellagoroi***sp. nov.**, *Nerudiellajaimei***sp. nov.**, *Nerudiellamalleco***sp. nov.**, *Nerudiellapenco***sp. nov.**, *Nerudiellapichi***sp. nov.**, *Nerudiellaportai***sp. nov.**, *Nerudiellaquenes***sp. nov.**, *Nerudiellavilches***sp. nov.**, *Nerudiellawekufe***sp. nov.**, and *Nerudiellazapallar***sp. nov.** Furthermore, we provide detailed illustrations of all the South American species belonging to these lineages formerly classified in *Nuncia*.

## ﻿Introduction

**Figure 1.**
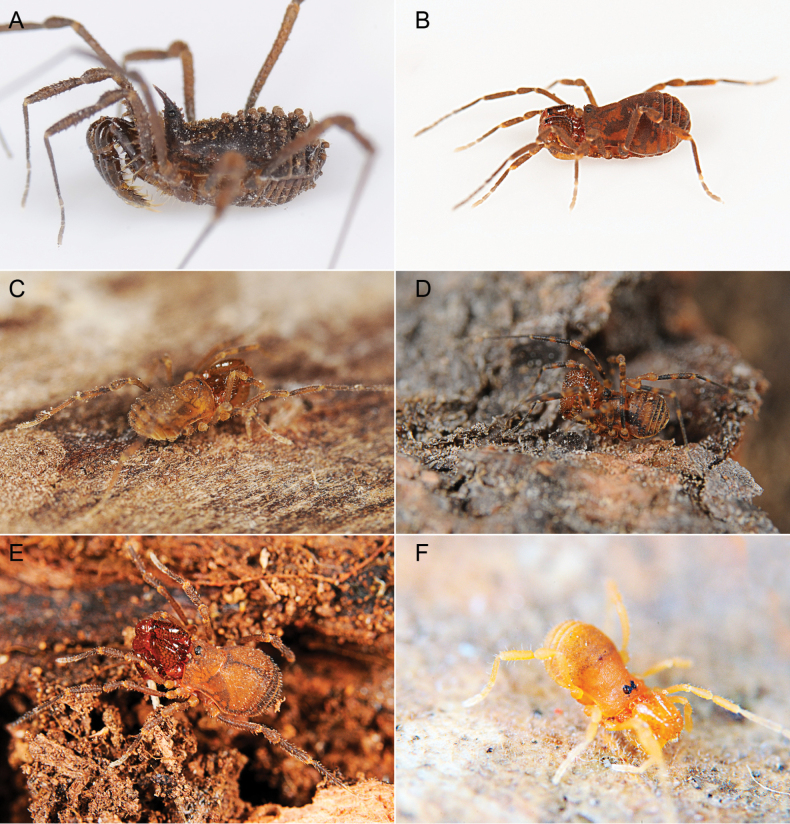
Photographs of live Triaenonychidae**A***Mistraliaverrucosa* comb. nov. **B***Chilenunciachilensis* comb. nov. **C***Nerudiella* gen. nov. **D***Fresiaxspinulosa* comb. nov. **E***Lautariaceachei* sp. nov. **F***Laftrachiarobin* sp. nov. Photographs copyright of Abel Pérez-González.

Triaenonychidae Sørensen, 1886, stands as the fourth most diverse family of Opiliones, encompassing 404 known species (Kury et al. 2022). These Laniatores, which are characterized by their small to medium-sized bodies and ranging in color from yellowish to brown (Fig. 1), thrive in the southern temperate regions of the ancient supercontinent Gondwana (Giribet and Kury 2007). Their presence extends across Australia, Madagascar, New Caledonia, New Zealand, South Africa, and southern South America, with at least one species having dispersed to the Crozet Islands (Enderlein 1909; Roewer 1914; Hickman 1939; Kury 2007; Mendes and Kury 2008; Mendes and Kury 2012; Baker et al. 2020; Porto and Pérez-González 2020; Derkarabetian et al. 2021a). In their natural habitat, triaenonychids are primarily nocturnal, actively navigating the moist temperate forests they inhabit. During daytime, they often seek refuge beneath woody debris, rocks, and even in caves (Pérez-Schultheiss et al. 2021).

*Nuncia*, described by Loman in 1902 from New Zealand, is recognized as the most speciose genus within Triaenonychidae, encompassing 63 species and subspecies (Kury et al. 2022). Initially, *Nuncia* was believed to have a distribution that included the Crozet Islands, New Zealand, and South America, a rare transoceanic distribution among triaenonychids (Mendes and Kury 2008). However, recent investigations incorporating molecular and morphological evidence have revealed that *Nuncia* does not form a monophyletic group, the nominal clade being exclusively confined to New Zealand (Porto and Pérez-González 2019; Baker et al. 2020; Derkarabetian et al. 2021a). Here we deal with the remaining *Nuncia*, as the “*Nuncia*” from the Crozet Islands is reinstated in its original genus, *Promecostethus*Enderlein 1909 (Kury et al. 2014; Porto and Pérez-González 2020).

The genus *Nuncia* was established by Loman (1902) for the species *Nunciasperata* Loman, 1902. Forster (1954) considered it a junior synonym of *Triaenonyxobesus* Simon, 1899, and transferred *T.obesus* to *Nuncia* under the name *Nunciaobesaobesa* (Simon, 1899). Forster (1954) made a significant contribution to the taxonomy of *Nuncia* through his comprehensive revision of the New Zealand Laniatores. His work resulted in the revision and description of 53 new species and subspecies of *Nuncia*. Furthermore, Forster’s revision encompassed the reorganization of the infra-generic status of *Nuncia*, resulting in the description of two subgenera (Forster 1954). In the subsequent years (Forster 1965) continued to contribute by documenting additional species of *Nuncia* from New Zealand.

The history of the South American *Nuncia* began when Roewer (1961) described *Nunciaamericana*, which marked the first occurrence of a ‘transcontinental’ genus within Triaenonychidae. Subsequently, Soares (1968) added the Tasmanian *Parattahia* Roewer, 1915, to the list of transcontinental genera with the description of *Parattahiachilensis* Soares, 1968, from Chile. In his influential work, Maury (1990) strengthened and expanded the Roewerian concept of transcontinental *Nuncia* when he described three new species from Chile and Argentina, *N.rostrata*, *N.spinulosa*, and *N.verrucosa*, and also transferred *Parattahiachilensis* to this genus. Maury (1990) not only established the new combination *Nunciachilensis* but also considered *Chilenunciadonosoi* Muñoz-Cuevas, 1971, its junior synonym; therefore, the hitherto monotypic Chilean genus *Chilenuncia* also fell into the synonymy of *Nuncia*. In the same work, Maury (1990) also reinstated the synonymy of Crozet Island’s monotypic *Promecostethus* with *Nuncia*. Since this work, *Nuncia* has been considered the triaenonychid genus with the largest distributional range and a textbook example of transcontinental distribution in opilionid genera.

Recent studies that have examined the relationships within Triaenonychidae worldwide utilizing molecular Sanger markers and ultraconserved element (UCE) data (Baker et al. 2020; Derkarabetian et al. 2021a, 2021b), have confirmed that the South American and New Zealand *Nuncia* represent separate genetic lineages, thus constituting a polyphyletic group. Furthermore, while the New Zealand *Nuncia* are monophyletic, the South American *Nuncia* belong to multiple independent genetic lineages, necessitating taxonomic revision and the proposal of new names that more accurately reflect their systematic classification as was claimed in recent works (Baker et al. 2020; Derkarabetian et al. 2021b).

In this study, our primary focus is on the taxonomy and systematics of the South American triaenonychid species traditionally included in the genus *Nuncia* Loman, 1902, aiming to provide a comprehensive understanding of their phylogeny through molecular analysis and examination of external and genital morphology. Before this study, there were five known species of “*Nuncia*” in South America. Our study has resulted in the redefinition of the Neotropical lineage within this group, as well as the formalization of new taxa. As part of our research, we have revalidated one genus, described five new genera, redescribed the five previously known species, and described 22 additional species. Furthermore, we provide detailed illustrations of all the South American species belonging to these lineages.

## ﻿Materials and methods

### ﻿Sample preparation

The specimens used in this study are deposited in the following institutions: American Museum of Natural History (**AMNH**), California Academy of Sciences (**CAS**), Field Museum of Natural History (**FMNH**), Museo Argentino de Ciencias Naturales (**MACN**), Museum of Comparative Zoology (**MCZ**), and Museo Nacional de Historia Natural, Santiago de Chile (**MNHNCL**). Examination and photography of the specimens were conducted using a Leica M205A stereomicroscope equipped with a Leica DF295 digital camera. Specimens were carefully cleaned using a fine bristle paint brush. For scanning electron microscopy (SEM) preparations, body parts were dissected, cleaned, dehydrated using an ethanol series (80%–90%–96%–100%), mounted on aluminum stubs, and coated with gold-palladium using a VG Scientific SC 7620 mini sputter-coater. SEM micrographs were captured under high vacuum using a Philips FEI XL30 TMP at the MACN.

**Figure 2.**
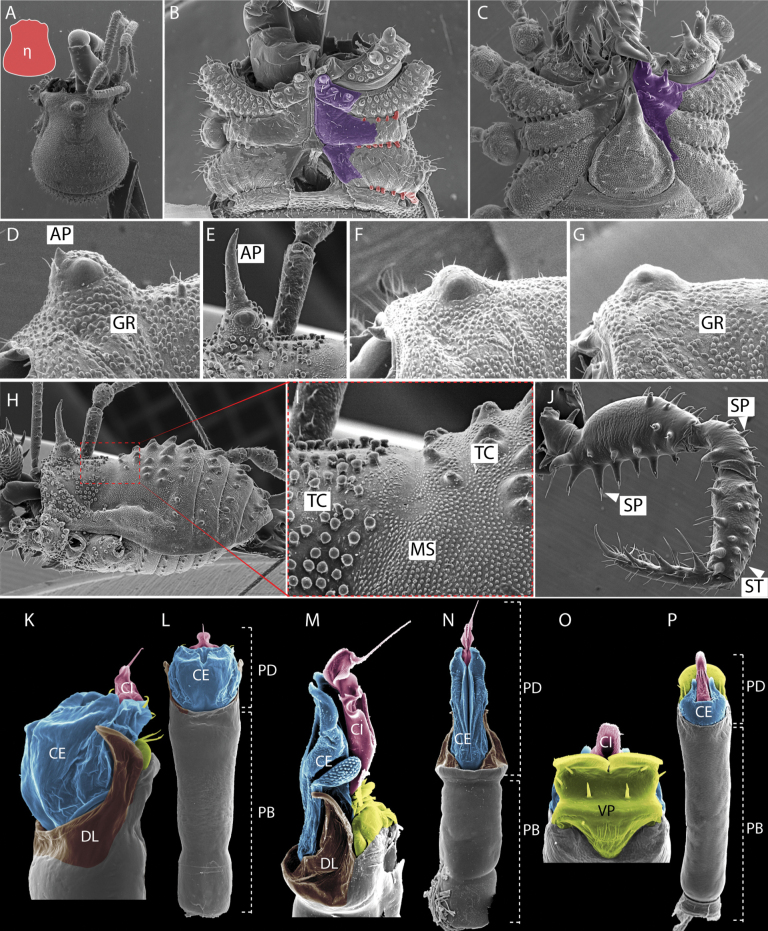
Guide to some taxonomic characters used in this study **A** shape dorsal scutum of Triaenonychidae**B, C** smooth areas of ventral coxae (purple) and ventral bridges(red) **D–G** shapes of ocularium **H, I** styles of integument **J** styles of pedipalps tubercles **K–P** styles of male genital morphology. Abbreviations: AP apophyses, CE capsula externa, CI capsula interna, DL dorsolateral plate,GR granules, MS microgranulation, PB pars basalis, PD pars distalis, SP (spines)large tubercles, ST setae, TC tubercles, VP ventral plate.

Male genitalia were dissected and temporarily mounted on microscope slides following the technique described in Acosta et al. (2007), with clove oil used as a clearing agent. Examination of male genitalia was conducted using an Olympus BH2 compound microscope, after which they were returned to 80% ethanol and stored in microvials alongside their respective specimens. The morphological nomenclature in this study adheres to the classifications provided by Mendes and Kury (2012), Murphree (1988)Pérez-González and Werneck (2018) and Porto and Pérez-González (2019). Scutum shape outline nomenclature follows the guidelines established by Kury and Medrano (2016). Other details about the external and genital morphology can be analyzed according to the models proposed herein (Fig. 2). All measurements in mm, unless otherwise stated.

### ﻿Molecular data

We targeted three markers, including two conserved nuclear ribosomal genes (18S and 28S rRNAs) and the mitochondrial gene cytochrome *c* oxidase subunit I. Molecular data were obtained from Baker et al. (2020), with additional sequences obtained from sequence capture of ultraconserved element (UCE) data available from Derkarabetian et al. (2021a) as “bycatch” (Suppl. material 1). For these UCE-derived sequences, we used Geneious v. 11.0 (https://www.geneious.com) to conduct local nucleotide BLAST searches for the associated UCE assembly file for each sample against closely related COI sequences which were available from Baker et al. (2020). Sequences for 15 additional taxa were generated for this study from specimens deposited in the MACN collected in 96% ethanol and kept at -20 °C. All voucher specimens for this study are thus deposited in the MACN or in the MCZ, the latter being available through MCZbase (https://mczbase.mcz.harvard.edu/).

For the new sequences, DNA was extracted from leg muscle using Chelex 10%, with an overnight incubation. 18S rRNA was amplified in two non-overlapping fragments using primer pairs 1F–5R, and 18Sa2.0–9R (Giribet et al. 1996; Whiting et al. 1997). A portion of 28S rRNA was sequenced in two overlapping fragments using primer pairs 28Sa–28Srd5b and 28Srd4.8a–28Srd7bi (Schwendinger and Giribet 2005). COI was amplified with the primer pair LCO1490–HCO2198 (Folmer et al. 1994). PCR reactions were carried out with 0.5–1.5 μL of DNA template, using Taq DNA polymerase in a 25-mL reaction. A 1% agarose gel electrophoresis was used to visualize amplification reactions. Successful reactions were cleaned using ExoSAP. Sanger sequencing was conducted at Macrogen Inc., Korea. New sequences have been deposited in GenBank (Suppl. material 1).

Sequences for each marker were checked and trimmed with Geneious 7.1.3 and aligned with Muscle (Edgar 2004) in MEG. Azevedo-X (Kumar et al. 2018), with the gap opening set at -400.00, 100 maximum iterations, and using UPGMA as clustering method. Aligned fragments were concatenated in Sequence Matrix (Vaidya et al. 2011) and the concatenated dataset was then subjected to subsequent phylogenetic analysis.

### ﻿Phylogenetic analysis

Model testing and phylogenetic analysis were performed in IQ-TREE (Nguyen et al. 2015), implementing the ModelFinder function (Kalyaanamoorthy et al. 2017) and partitioning by locus (Chernomor et al. 2016). Nodal support was assessed with a Shimodaira–Hasegawa approximate likelihood ratio test (SH-aLRT) and ultrafast bootstrap analysis (UFBoot) (Hoang et al. 2018), specifying 1000 replications for each. The runs were repeated 10 times and three topology tests were conducted: approximately unbiased AU (Shimodaira 2002), bootstrap proportion (Kishino et al. 1990), Kishino–Hasegawa (Kishino and Hasegawa 1989), and expected likelihood weight (Strimmer and Rambaut 2002), using 10,000 resampling-estimated log-likelihoods in IQ-TREE with the 10 trees. A consensus tree summarizing the 10 runs was constructed. This tree served as a reference for the discussion and was further refined and customized using FigTree v. 1.4.3 and Adobe Illustrator 2019. The maps in this study were constructed using shapefiles sourced from Natural Earth data. Additionally, the ecoregions shapefiles were obtained from WWF terrestrial ecoregions (Olson et al. 2001). The map visualization was performed using R Statistical Software (R Core Team 2022) and the following packages: sf (Pebesma 2018, Pebesma and Bivand 2023), ggplot2 (Wickham 2016), ggspatial (Dunnington et al. 2023), and ggpubr (Kassambara 2023).

## ﻿Results and discussion

### ﻿Phylogenetics of South American lineages

**Figure 3.**
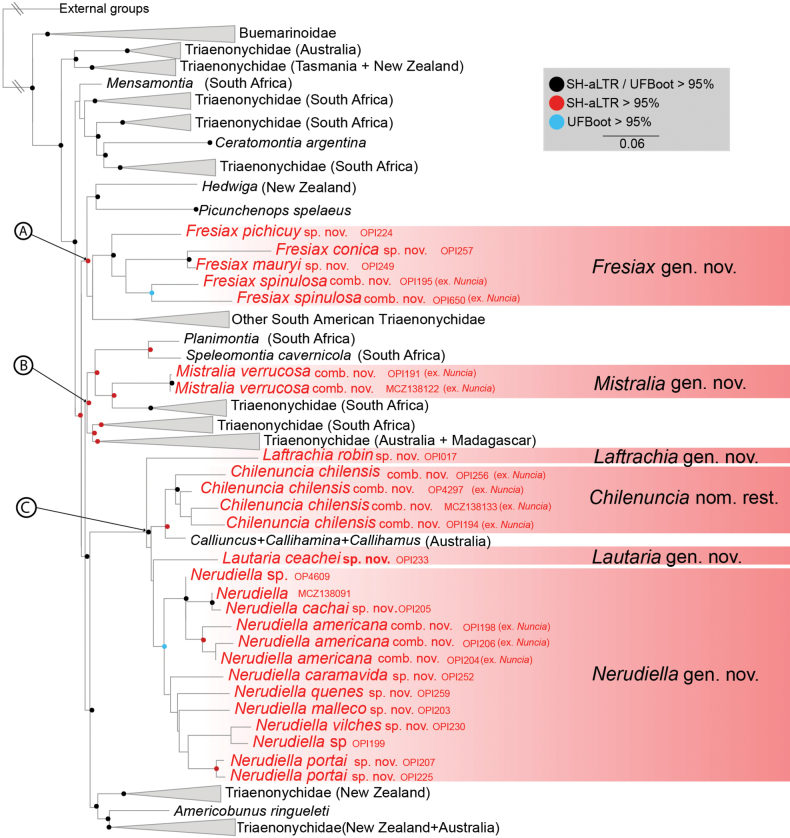
Phylogenetics relationships of Triaenonychidae, with clade names as discussed in the main text. Phylogeny is derived from the IQ-TREE analysis. Nodes without circles have < 95% bootstrap support to AH-aLR and UFBoot.

In our phylogenetic analysis (Fig. 3), we found support for the hypothesis that the previously known South American “*Nuncia*” species are polyphyletic assemblage of taxa, where “*Nuncia*” *spinulosa* and three related new species being sister to other South American triaenonychids clade, “*Nuncia*” *verrucosa* being related to a core of South African species. Nevertheless, "*Nuncia" chilensis* being closely related to a series of Australian genera (*Calliuncus*, *Callihamina*, and *Callihamus*), and "*Nuncia" americana* form a clade with other six new South American species. This finding is in most part consistent with previous hypotheses based on both Sanger data and UCEs (Baker et al. 2020; Derkarabetian et al. 2021a; Porto et al. 2022).

Our analyses consistently recovered three independent lineages with SH-aLTR high support (clades A and B) or SH-aLTR and UFboot high support (clade C) where the representatives of the South American “*Nuncia*” are nested. We discuss the phylogenetic relationships of the South American “*Nuncia*” based on the preferred tree obtained in the IQ-TREE results (Fig. 3).

We recovered the species originally described as *Nunciaspinulosa* Maury, 1990, deeply nested inside the big clade A (SH-aLTR > 95%). We herein combined under *Fresiax* gen. nov. the species: *Fresiaxspinulosa* comb. nov. and related species (*F.conica* sp. nov., *F.mauryi* sp. nov., *F.pichicuy* sp. nov. – based on molecular and morphological evidence – and *F.fray* sp. nov. based on morphological evidence only). The monophyly of *Fresiax* gen. nov. received strong support from both SH-aLTR and UFBoot methods. This new genus exhibits a close relationship with other South American Triaenonychidae genera, including *Adrianonyx*, *Araucanobunus*, *Diasia*, *Nahuelonyx*, *Triaenonychoides*, *Triaenonyx* and *Valdivionyx* (see Suppl. material 2). Additionally, it shares connection with the New Zealand genus *Hedwiga* and the South American cave relictual species *Picunchenopsspelaeus*. The early divergence within clade A suggests that *Picunchenopsspelaeus* has more affinities with the New Zealand genus *Hedwiga* than with other South American representatives of this clade. Besides *Fresiaxspinulosa* comb. nov., we described four other new species, three of which have accompanying molecular data.

In contrast “*Nuncia*” *verrucosa* Maury, 1990, does not show a relationship with South American triaenonychids. Instead, it is recovered as the sister group of South African species, including *Adaeulum*, *Larifuga*, *Larifugella*, and *Paradaeum* (SH-aLTR > 95%) (see Suppl. material 2). This group of species is deeply nested in the clade B (SH-aLTR > 95%), which is composed of South African, Malagasy, and Australian species (Fig. 3). To name this distinct genus, we described *Mistralia* gen. nov., and consequently, we introduce the new combination *Mistraliaverrucosa*. Due to the external and genital morphological affinities (no molecular data available), we described a second species to this genus, *Mistraliaramirezi* sp. nov. The unique morphology of *Mistralia* was first noted by Maury (1990) when he established the “verrucosa group.”

Clade C (SH-aLTR and UFBoot > 95%) represents the most diverse and intricate group, comprising triaenonychids that were previously classified or could be classified within the former concept of South American “*Nuncia*”. Clade C includes the previously known species “*Nuncia” americana* and “*Nuncia” chilensis*. However, both species are recovered as part of different lineages. For “*Nuncia” chilensis*, we have reinstated the previously synonymized genus *Chilenuncia* Muñoz-Cuevas, 1971, and consequently established the new combination *Chilenunciarostrata*. Unfortunately, we do not have molecular data of “*Nuncia*” *rostrata* Maury, 1990, but the morphological similarities with *Chilenunciachilensis* support our decision of include this species in the genus *Chilenuncia* combining them as *Chilenunciarostrata* comb. nov. Our four terminals identified as *Chilenunciachilensis* comb. nov. were strongly (SH-aLTR > 95%) recovered as monophyletic and are sister of a monophyletic group that includes the Australian genera: *Calliuncus* Roewer, 1931, *Callihamina* Roewer, 1942, and *Callihamus* Roewer, 1931. This group exhibits an extraordinary stasis in their external morphology, but the genital morphology supports the South American representatives as a different genus as well as the molecular evidence. This morphological stasis could be responsible for the original description as *Parattahiachilensis* Soares, 1968, but the examination of the type species *Parattahiau-signata* Roewer, 1914, and their male genital morphology (data not showed) not support the inclusion in the same genus and reinforce the restoration of *Chilenuncia*. The relationship and limits of the genera *Parattahia*, *Calliuncus*, *Callihamina*, and *Callihamus* deserve further attention. The phylogenetic relationship between *Chilenuncia* and the Australian genera was also recovered by Derkarabetian et al. (2021a) who estimated a divergence between the South American and Australian species of approximately 40 million years ago.

As part of the Clade C, we recovered a clade with strong UFBoot support that included the previous species *Nunciaamericana* Roewer, 1961, plus several new species. We described the genus *Nerudiella* gen. nov. to accommodate all the species closely related with “*Nuncia*” *americana*. Besides *Nerudiellaamericana* comb. nov. we have described fifteen new species based on morphology and six of them also based on molecular evidence. This is a remarkably diverse group of South American triaenonychid that exhibits a very interesting radiation that deserves further studies. Finally, in the Clade C, we described two new monotypic genera, namely *Laftrachia* gen. nov. and *Lautaria* gen. nov.

The entire Clade C is recovered with high support (SH-aLTR and UFBoot > 95%) as sister of another clade, primarily composed of Australian and New Zealand species, were the South American monotypic relict taxon *Americobunusringueleti* Muñoz-Cuevas, 1972, is nested.

### ﻿Biogeographical remarks

A comprehensive biogeographic study of Triaenonychidae has been conducted by Derkarabetian et al. (2021a), where all South American lineages recovered in our study were represented. The lineages which are here called A, B, and C are equivalent to clades C, D, and E in Derkarabetian et al. (2021a). Our study contains a denser sampling of South American triaenonychids and thus, examining them in the context of the divergence dating in Derkarabetian et al. (2021a) reinforces interesting biogeographic relationships. We know that Triaenonychidae is an ancient group, they existed for ~ 237 million years, predating the fragmentation of Gondwana initiated around 180 million years ago. Our South American lineages A, B, and C exhibit a highly complex biogeographic history with their origin and early diversification predating Gondwanan fragmentation. Other groups like the new genus *Mistralia*, appear to have originated from a cladogenetic event coinciding with the split of West Gondwana into South America and Africa, occurring ~ 140 million years ago. The divergence between the Australian taxa *Calliuncus*–*Callihamus*–*Callihamina* and the South American restored genus *Chilenuncia* (clade C) is estimated to have occurred approximately 45 million years ago, suggesting the possibility of a land connection between South America and Australia via Antarctica (see Derkarabetian et al. 2021a). This is not only an interesting biogeographic pattern to be further studied—a pattern that nonetheless includes a remarkable long-distance oceanic dispersal to the Crozet Islands (Baker et al. 2020)—but also from a morphological point of view because it contains an astonishing stasis.

A time-calibrated phylogeny with denser sampling, including the new taxa described here and other representatives of South American triaenonychids, is currently underway where this and other biogeographic and evolutionary questions will be addressed on a more regional scale within South America.

### ﻿Taxonomic section

#### ﻿Clade A

##### ﻿Genus Fresiax gen. nov.

Taxon classificationAnimaliaOpilionesTriaenonychidae

BF683A71-A2C0-58C6-8A36-B56B81A4031A

https://zoobank.org/88A21A16-8380-4FDE-9EB5-DEC3BC2276DD

Figs 5 , 6 , 7 , 8 , 9 , 10 , 11 , 12 , 13 , 14 , 15 , 16 , 17 , 18 , 19 , 20 , 21 , 22 , 23 , 24 , 25 , 26 , 27 , 28 , 29 , 30 , 31 , 32 , 33 , 34

Nuncia [part] (only references to Nunciaspinulosa): Maury 1990: 108; Maury 1992: 5; Acosta and Maury 1998: 579; Kury 2003: 22.

###### Etymology.

The genus name *Fresiax* is a combination of “Fresia,” the name of the wife of Mapuche’s military leader Caupolicán, and the Greek word ὄνυξ (onyx = nail, claw), which is part of the generic name *Triaenonyx*, the type genus of the family Triaenonychidae. The genus name *Fresiax* is feminine.

###### Diagnosis.

The male genitalia of *Fresiax* exhibits a pars distalis considerably reduced (as in *F.mauryi* sp. nov.), a ventral plate longer than the capsula externa (as in *F.conica* sp. nov.), and a capsula externa divided into two lamellae, a character that distinguishes it from any other triaenonychid genus. Additionally, the pedipalp femora possesses at least three dorsal and four ventral spines.

###### Type species.

*Nunciaspinulosa* Maury, 1990

###### Included species.

*Fresiaxconica* sp. nov., *Fresiaxfray* sp. nov., *Fresiaxmauryi* sp. nov., *Fresiaxpichicuy* sp. nov., *Fresiaxspinulosa* (Maury, 1990), comb. nov.

###### Distribution.

**Figure 4.**
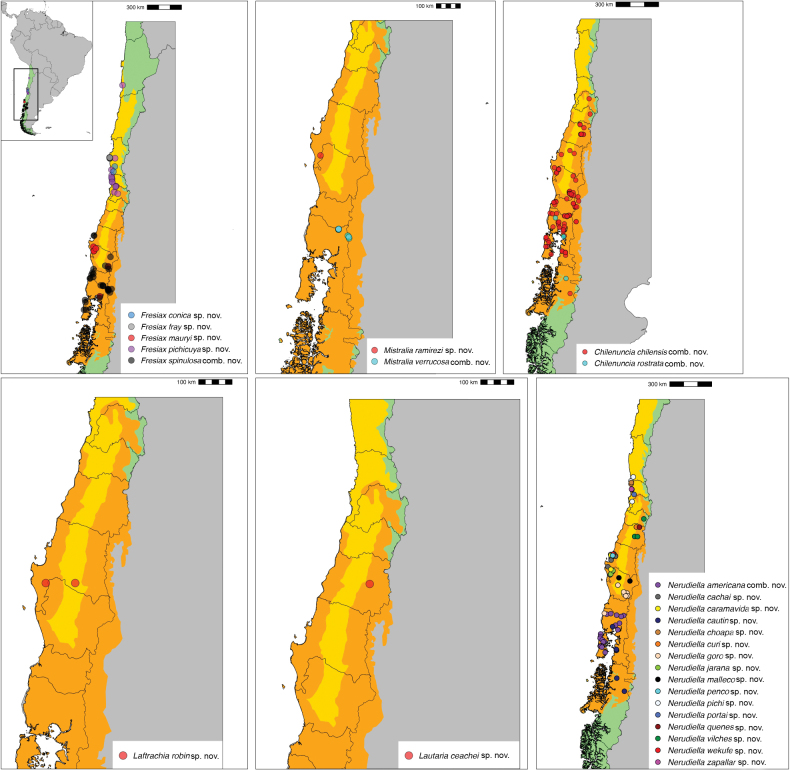
Maps showing the distribution of the species used in this work. South America (Chile and western Argentina inset). Chile in green, Argentina/ South America in grey. Terrestrial ecoregions (following Olson et al. 2001) highlighted in yellow (Chilean Matorral) and orange (Valdivian temperate forests).

Argentina: Río Negro Province. Chile: Coquimbo, Valparaíso, Araucanía, Los Ríos, Los Lagos (Fig. 4A).

##### ﻿ Fresiax conica sp. nov.

Taxon classificationAnimaliaOpilionesTriaenonychidae

F4E975D9-DD0C-5C9F-A57E-5B46ADB439DE

https://zoobank.org/5E85033C-CB20-4C7D-B42D-EEE6FFFF443B

**Figure 5.**
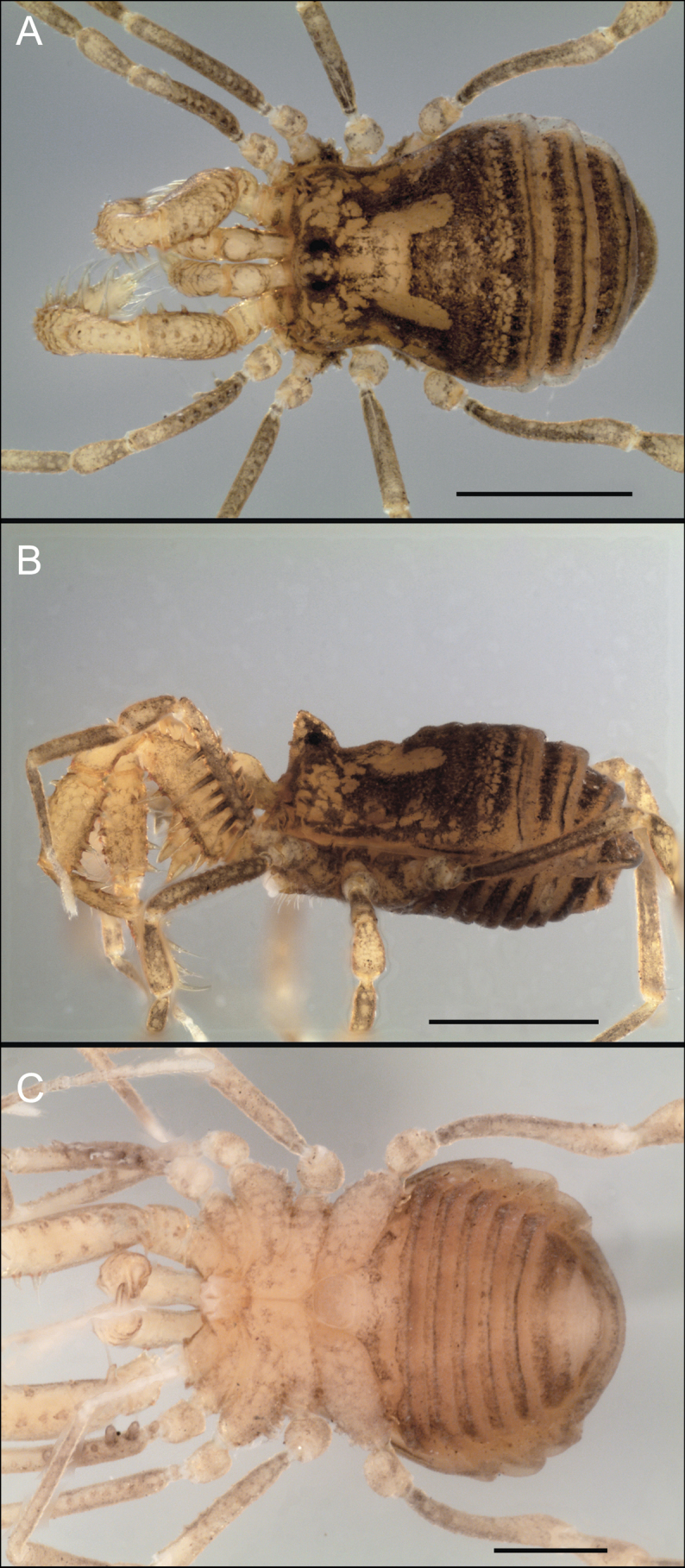
*Fresiaxconica* sp. nov. male **A** dorsal view **B** lateral view **C** ventral view. Scale bars: 1 mm. Species of Clade A, see Fig. 3.

**Figure 6.**
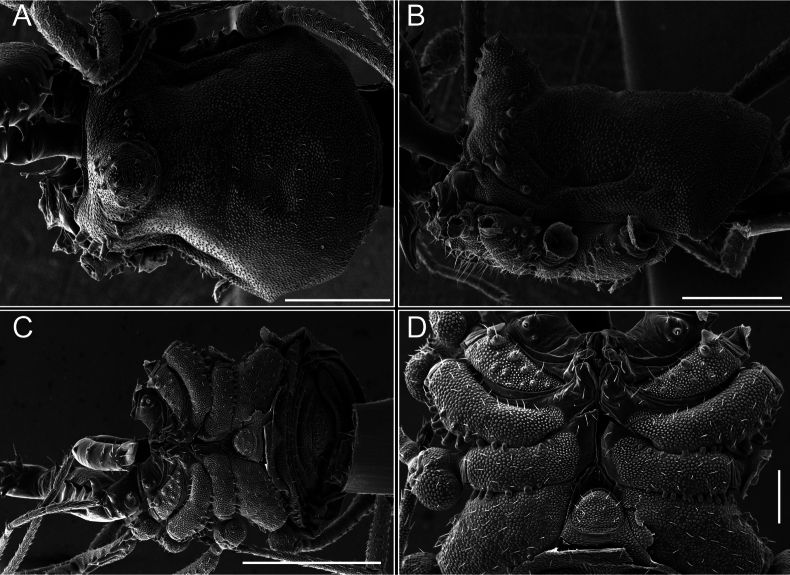
*Fresiaxconica* sp. nov. male, SEM images of habitus **A** dorsal view **B** lateral view **C, D** ventral view. Scale bars: 500 µm (**A, B, C**); 200 µm (**D**).

**Figure 7.**
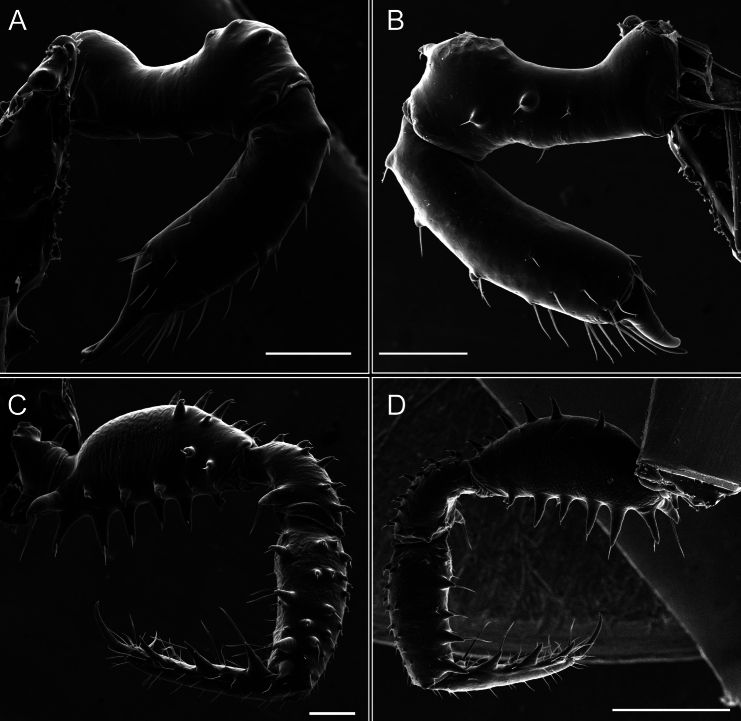
*Fresiaxconica* sp. nov. chelicerae: mesal **A** ectal **B** pedipalps: mesal **C** ectal **D**. Scale bars: 200 µm.

**Figure 8.**
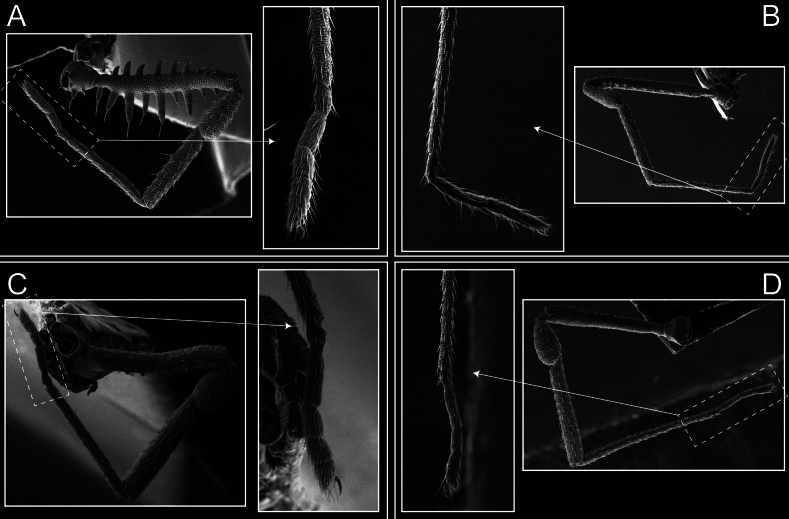
*Fresiaxconica* sp. nov. legs I **A** II **B** III **C** IV **D**. Scale bars: 200 µm (**A, C**); 500 µm (**B, D**).

**Figure 9.**
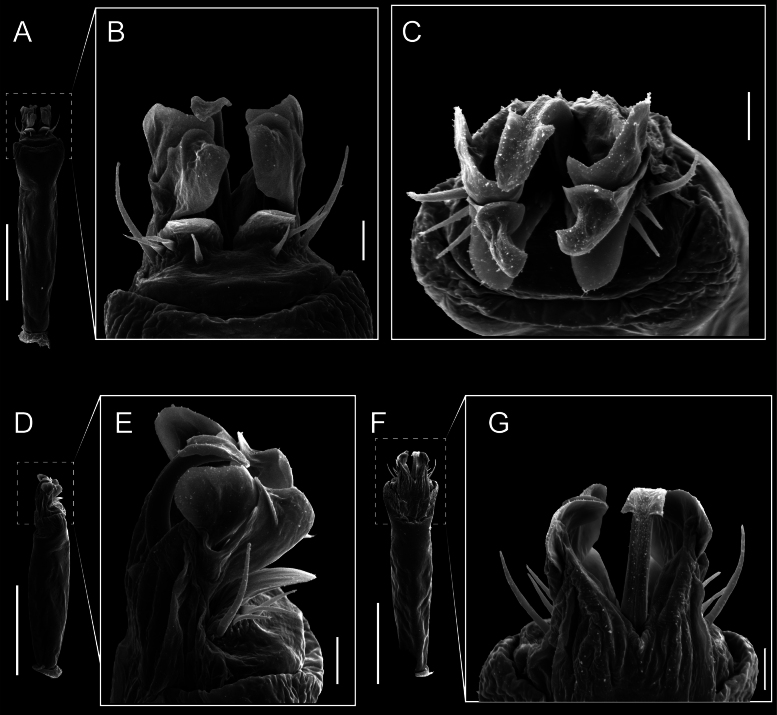
*Fresiaxconica* sp. nov. penis: ventral **A, B** apical **C** lateral **D, E** dorsal **F, G**. Scale bars: 200 µm (**A, D, F**); 20 µm (**B, C**, **E, G**).

**Figure 10.**
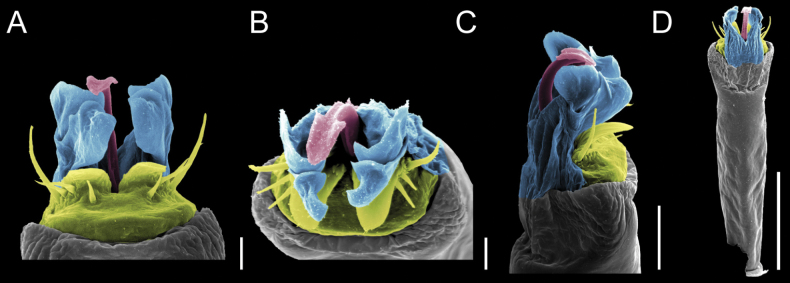
*Fresiaxconica* sp. nov. penis: ventral **A** apical **B** lateral **C** dorsal **D**. Colors: ventral plate (yellow), capsula externa (blue), capsula interna (red). Scale bars: 20 µm (**A, B**); 50 µm (**C, D**).

Figs 5 , 6 , 7 , 8 , 9 , 10

###### Material examined.

***Holotype*.** ♂ **Chile.** Choapa: Cuesta Cavilolén, 31.76669°S, 71.32727°W, 300 m, M. Ramírez, A. Ojanguren, J. Pizarro coll. 11.II.2011 (MNHNCL). ***Paratypes*. Chile.** Choapa: Los Vilos, Cuesta Caviolén, 30 km NE de Los Vilos, E. Maury coll., 1 ♀ 12.XII.1987 (MACN). Choapa: Los Vilos, Cuesta Caviolén, 30 km NE de Los Vilos, E. Maury coll., 1 ♀ 12.XII.1987 (MACN).

###### Etymology.

The term “conica” refers to the shape of the ocularium, which is conical and lacks an apophysis. The specific epithet was also a name “in schedula” by the late Dr. Emilio Maury that labeled the specimens. Maury early recognized this species as a new but never published. We keep the Maury’s specific name to maintain the name preference of the researcher who first recognized this species as new.

###### Diagnosis.

The conical ocularium, without acute apophysis, clearly distinguishes this species from others in the genus. There is only one row of tubercles on the anterior region of the dorsal scutum. The apical section of the tubular capsula interna is subtriangular.

###### Distribution.

Chile: Coquimbo Region (Fig. 4A).

###### Description of male.

Measurements: Total length 2.01, carapace length 0.78, dorsal scutum length 1.30, carapace max. width 1.05, mesotergum max. length 1.50. Appendage measurements: Pedipalps. Length of trochanter 0.13, length of femora 0.80, length of patella 0.46, length of tibia 0.53, length of tarsus 0.62. Leg I: trochanter (tr) 0.16, femora (fe) 0.16, patella (pa) 0.74, tibia (ti) 0.38, metatarsus (mt) 0.51, tarsus (ta) 0.62. II: tr 0.46, fe 0.22, pa 1.03, ti 0.41, mt 0.80, ta 0.98. III: tr 0.93, fe 0.19, pa 0.68, ti 0.36, mt 0.60, ta 0.67. IV: tr 0.62, fe 0.26, pa 1.04, ti 0.46, mt 0.80, ta 1.11.

Dorsum (Fig. 5, 6). Eta (η) or hourglass-shaped dorsal scutum (Kury and Medrano 2016). Ocularium conical with a group of ~ 20 setiferous tubercles. Eyes located on the middle of the ocularium. Dorsal scutum microgranulate with a row of setiferous tubercles on each side of the ocularium, without clear delimitation of the mesotergal areas. Areas I–IV and posterior margin with seta, I with two, II and III with four, and IV and posterior margin with six. All free tergites bear a row of small, setiferous, rounded granules.

Chelicerae (Fig. 7A, B). Segment I with few sparse setae. Segment II with three small frontal tubercles and sparse setae.

Pedipalps (Fig. 7C, D). Trochanter with long ventral and dorsal spines. Femora bearing a remarkable ventral-proximal subtriangular spine with long subdistal setae; a row of four ventral spines, with subdistal setae and six dorsal small spines with setae. Patella bearing a notable mesal spine with long setae. There are two rows of small setiferous tubercles on the dorsal patella-tarsus. Tibia shows a ventral row of four long spines. Tarsus with three mesal and ectal setiferous spines.

Legs (Fig. 8). Coxa I with two rows of setiferous tubercles, the distal one with a subdistal setae. II–IV are microgranulate, with four bridges between the legs II and III, 6–8 bridges between the legs III and IV, seven between the leg IV and the opisthosoma. Spiracles not covered by bridges. The smooth surface represents < ¼ of coxa and leg III. Sternum arrow-shaped. Legs I–IV covered in setae, tarsal area and calcaneus are also setose. Trochanter I with small ventral granules. Femora I with a row of nine dorsal and ventral setiferous tubercles. Calcaneus is shorter than astragalus (7× shorter in leg I, 8× in II, 13× in III, 15× in IV). Tarsal count 3–6–4–4.

Penis (Figs 9, 10). Pars distalis has a large ventral plate with a cleft separating the plate into two lamellae. Each one bears three acute macrosetae on the ventral surface and one acute macroseta on the dorsal surface. Capsula externa is cleft and covers the dorsal and lateral surfaces. The Capsula interna is tubular with an apical subtriangular portion.

**Female.** Similar to male, with shorter pedipalpal femora.

Female measurements. Total length 1.91, length of carapace 0.76, length of dorsal scutum 1.29, max. width of carapace 1.04, max. width of mesotergum 1.47. Appendage measurements: Pedipalps. Length of trochanter 0.20, length of femora 0.76, length of patella 0.43, length of tibia 0.63, length of tarsus 0.56. Leg I: trochanter (tr) 0.26, femora (fe) 0.88, patella (pa) 0.38, tibia (ti) 0.56, metatarsus (mt) 0.64, tarsus (ta) 0.384. II: tr 0.23, fe 1.14, pa 0.53, ti 0.84, mt 0.90, ta 0.73. III: tr 0.22, fe 0.88, pa 0.36, ti 0.69, mt 0.88, ta 0.43. iv: tr 0.23, fe 1.07, pa 0.49, ti 0.95, mt 1.26, ta 0.61.

##### ﻿ Fresiax fray sp. nov.

Taxon classificationAnimaliaOpilionesTriaenonychidae

0FDE6857-82E9-547B-AFDC-F6658E7A65CB

https://zoobank.org/0F310737-611E-4AB6-B41D-FD12B585ECA5

**Figure 11.**
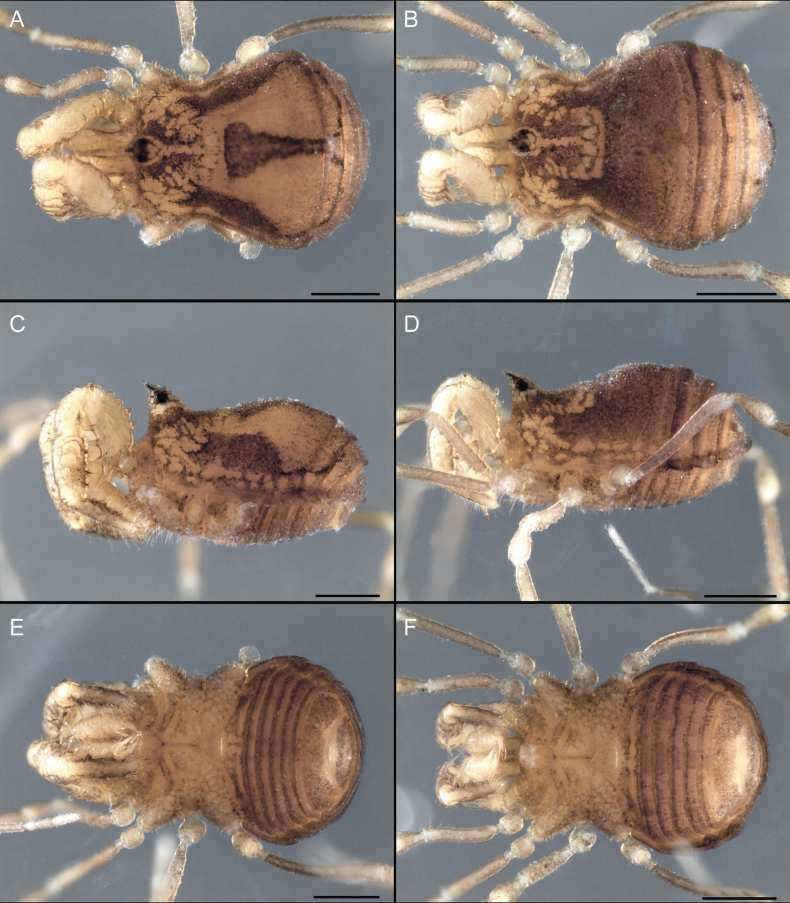
*Fresiaxfray* sp. nov. habitus, male **A** dorsal view **C** lateral view **E** ventral view. Female **B** dorsal view **D** lateral view **F** ventral view. Scale bars: 500 µm. Species of Clade A, see Fig. 3.

**Figure 12.**
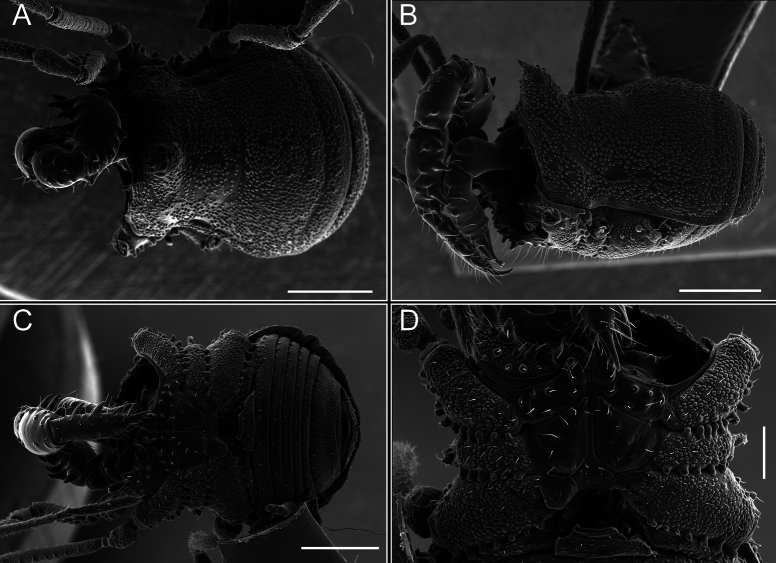
*Fresiaxfray* sp. nov. male, SEM images of habitus **A** dorsal view **B** lateral view **C, D** ventral view. Scale bars: 500 µm (**A, B, C**); 200 µm (**D**).

**Figure 13.**
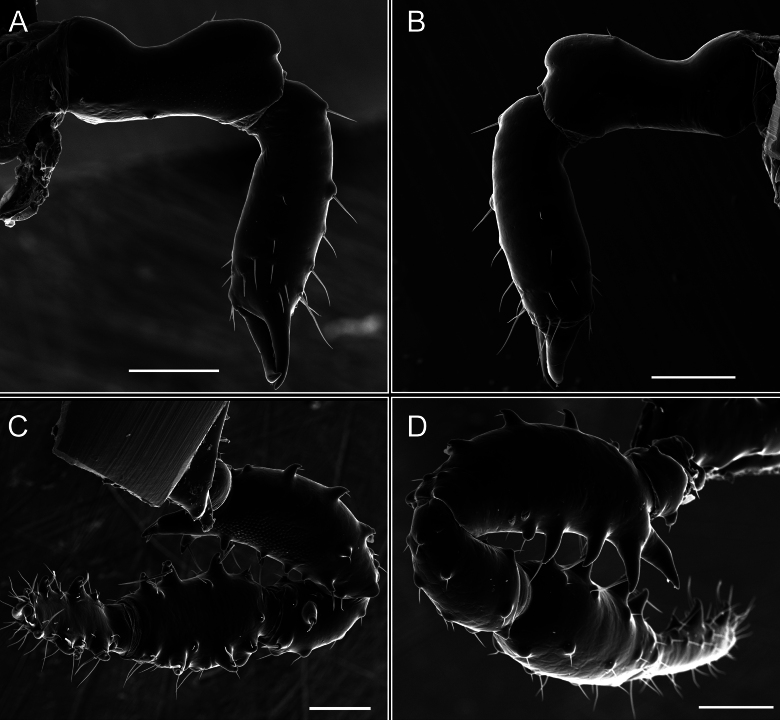
*Fresiaxfray* sp. nov. chelicerae: mesal **A** ectal **B** pedipalps: mesal **C** ectal **D**. Scale bars: 200 µm.

**Figure 14.**
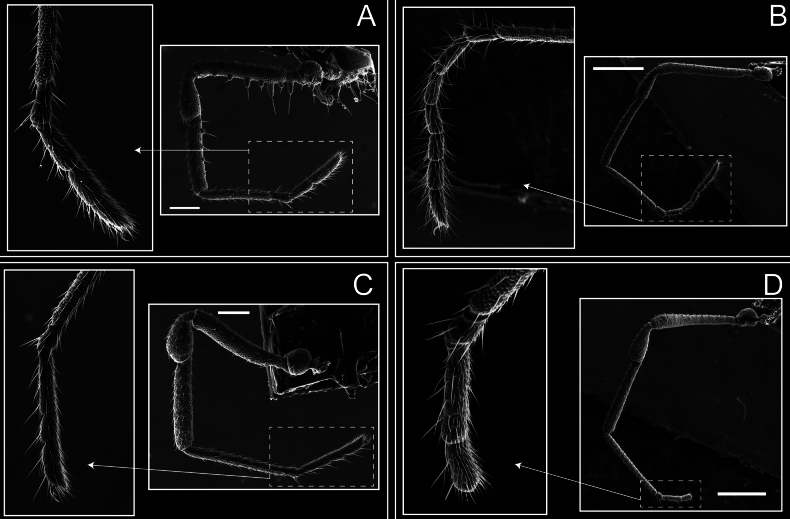
*Fresiaxfray* sp. nov. legs I **A** II **B** III **C** IV **D**. Scale bars: 200 µm (**A, C**); 500 µm (**B, D**).

**Figure 15.**
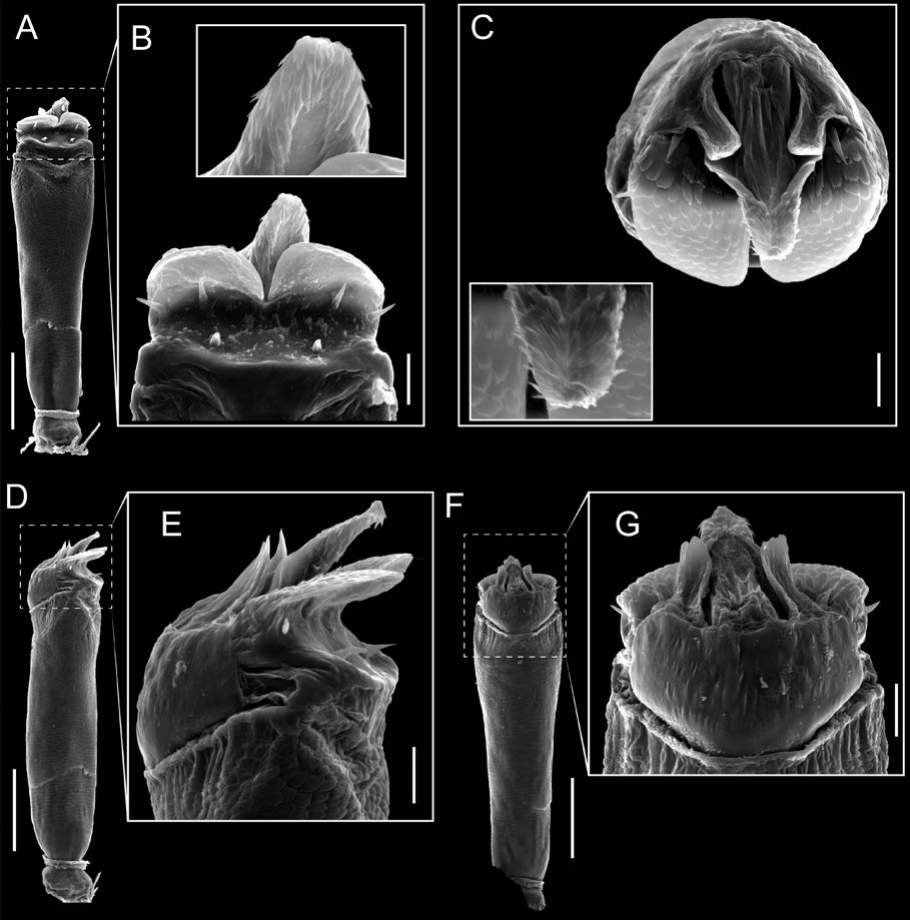
*Fresiaxfray* sp. nov. penis: ventral **A, B** apical **C** lateral **D, E** dorsal **F, G**. Scale bars: 100 µm (**A, D, F**); 20 µm (**B, C, E, G**).

**Figure 16.**
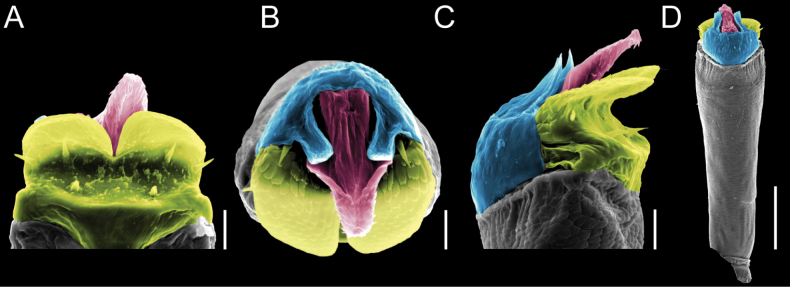
*Fresiaxfray* sp. nov. penis: ventral **A** apical **B** lateral **C** dorsal **D**. Colors: ventral plate (yellow), capsula externa (blue), capsula interna (red). Scale bars: 20 µm.

Figs 11 , 12 , 13 , 14 , 15 , 16

###### Material examined.

***Holotype*.** ♂ **Chile.** Coquimbo: Bosque de Fray Jorge, E. Maury coll. 03.XI.1988 (MNHNCL). ***Paratypes*. Chile.** Coquimbo: Limarí, Bosque Fray Jorge, P.N. Fray Jorge, E. Maury coll. 03.XI.1988, 38 ♂ 24 ♀, amm. (MACN).

###### Additional material.

Chile: Coquimbo: Limarí, Bosque Talinay, P.N. Fray Jorge, relict Valdivian fog forest, R. Schuh, N. Platnick coll., 08.II.1986, 66 specimens (AMNH).

###### Etymology.

The epithet *fray*, a noun in apposition, refers to the type locality of the species, Bosque Fray Jorge National Park.

###### Diagnosis.

This species can be easily distinguished from the other species of the genus by its small size (< 2 mm long), conical ocularium, with an apophysis at an angle of 45 °.

###### Distribution.

Chile, Coquimbo Region (Fig. 4A).

###### Description of male.

Measurements: Total length 1.81, carapace length 0.69, dorsal scutum length 1.47, carapace max. width 1.14, mesotergum max. length 1.49. Appendage measurements: Pedipalps. Length of trochanter 0.13, length of femora 0.80, length of patella 0.46, length of tibia 0.53, length of tarsus 0.62. Leg I: trochanter (tr) 0.16, femora (fe) 0.74, patella (pa) 0.38, tibia (ti) 0.51, metatarsus (mt) 0.62, tarsus (ta) 0.46. II: tr 0.22, fe 1.03, pa 0.41, ti 0.80, mt 0.98, ta 0.93. III: tr 0.19, fe 0.68, pa 0.36, ti 0.60, mt 0.67, ta 0.62. IV: tr 0.26, fe 1.04, pa 0.46, ti 0.80, mt 1.11, ta 0.57.

Dorsum (Fig. 11, 12). Eta (η) hourglass-shaped dorsal scutum. Ocularium conical, with a small, forward-pointing apical spine and small setae. Eyes located on the distal region of the ocularium. Dorsal scutum microgranulate with no clear area delimitation. Areas I–III with two small setiferous granules, IV with three, posterior margin with a row of small setiferous granules. All free tergites with a row of small setiferous granules.

Chelicerae (Fig. 13A, B). Segment I characterized by a smooth surface without any prominent tubercles or setae. In contrast, segment II with a small frontal tubercle and covered in setae. Segment II with more pronounced texture and setal coverage compared to the smooth surface of segment I.

Pedipalps (Fig. 13C, D). Trochanter with a dorsal and a ventral spine, both with setae. Femora with a row of four dorsal spines with subdistal setae, a row of five ventral spines with setae, the proximal one with a subtriangular shape; two dorsal and distal tubercles with setae, a row of five small mesal tubercles with setae, and three small distal tubercles with setae. Patella with two rows of three tubercles with setae, two small mesal tubercles with setae, and few ventral granules. Tibia with four ectal and mesal spines with subdistal setae; three ventral tubercles with setae and two rows of 4–5 granules with setae on the dorsal surface. Tarsus with three mesal and ectal spines with subdistal setae, as well as few setae and granules.

Legs (Fig. 14). Coxa I bearing small setiferous tubercles and a row of three long tubercles with subdistal setae; II–IV with microgranulation, bearing 4–6 bridges between legs II and III, five or six between III and IV, six or seven between leg IV and the opisthosoma. Spiracles not visible. Smooth area occupies 1/3 of the leg II, almost ½ of III, and only a small proximal portion of the leg IV. Smooth area of leg II with five small setiferous tubercles, III with a row of four and an anterior process directed to the sternum. Sternum arrow-shaped, with a triangular posterior area. Leg I trochanter with three small ventral setiferous tubercles and one dorsal tubercle. Femora of leg I with a row of seven setiferous tubercles and a dorsal row of small setiferous tubercles. Tibia of leg I with 3–4 ventral tubercles with setae. Legs II–IV covered in setae, with the tarsus and calcaneus area densely covered in setae. Calcaneus smaller than astragalus, ≥ 4× smaller in leg I, 5× smaller in leg II, 8× smaller in leg III, and 7× smaller in leg IV. These differences in size ratios between the calcaneus and astragalus serve as distinguishing characteristics among the legs of this species. Tarsal count 3–6–4–4.

Penis (Figs 15, 16). Pars distalis with a ventral plate and a prominent cleft that divides the plate into two lamellae. Each lamella is equipped with three macrosetae on the ventral surface and one macroseta on the dorsal surface. Capsula externa nearly as long as the capsula interna, having a notch that divides the capsula externa into two halves.

**Female.** Similar to male, with shorter pedipalpal femora.

Female measurements. Total length 1.54, length of carapace 0.61, length of dorsal scutum 1.31, max. width of carapace 0.96, max. width of mesotergum 1.35. Appendage measurements: Pedipalps. Length of trochanter 0.11. Length of femora 0.58, length of patella 0.37, length of tibia 0.45, length of tarsus 0.46. Leg I: trochanter (tr) 0.18, femora (fe) 0.62, patella (pa) 0.33, tibia (ti) 0.46, metatarsus (mt) 0.53, tarsus (ta) 0.42. II: tr 0.18, fe 0.88, pa 0.34, ti 0.71, mt 0.84, ta 0.86. III: tr 0.18, fe 0.62, pa 0.26, ti 0.53, mt 0.68, ta 0.49. IV: tr 0.21, fe 0.80, pa 0.37, ti 0.73, mt 0.98, ta 0.57.

##### ﻿ Fresiax mauryi sp. nov.

Taxon classificationAnimaliaOpilionesTriaenonychidae

F531F20A-176D-5008-A940-D44A8E7FEE3B

https://zoobank.org/4DA933F8-1369-4BCE-B27B-E068D0A74188

**Figure 17.**
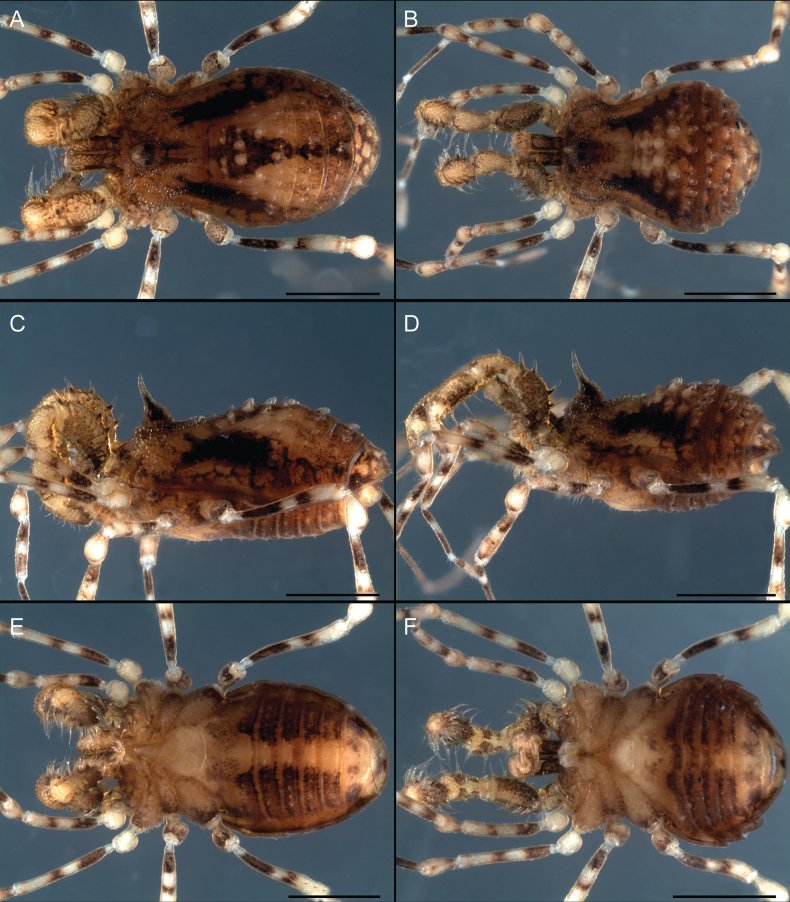
*Fresiaxmauryi* sp. nov. habitus, male **A** dorsal view **C** lateral view **E** ventral view. Female **B** dorsal view **D** lateral view **F** ventral view. Scale bars: 1 mm. Species of Clade A, see Fig. 3.

**Figure 18.**
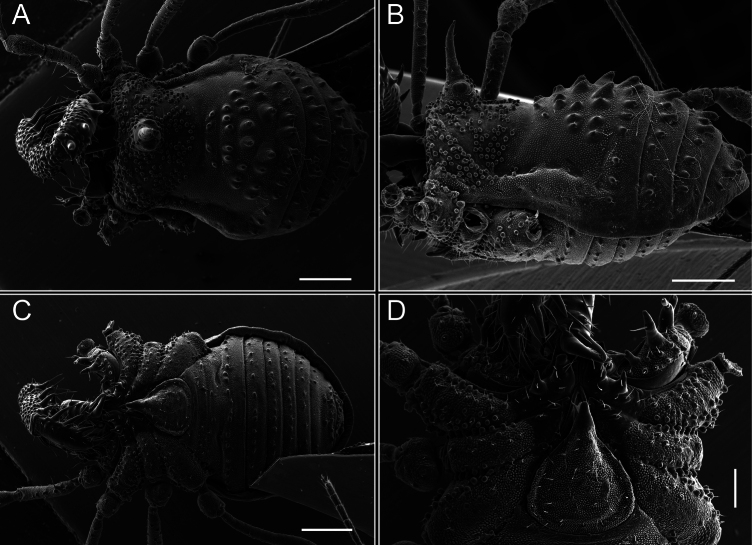
*Fresiaxmauryi* sp. nov. male, SEM images of habitus **A** dorsal view **B** lateral view **C, D** ventral view. Scale bars: 500 µm (**A, B, C**); 200 µm (**D**).

**Figure 19.**
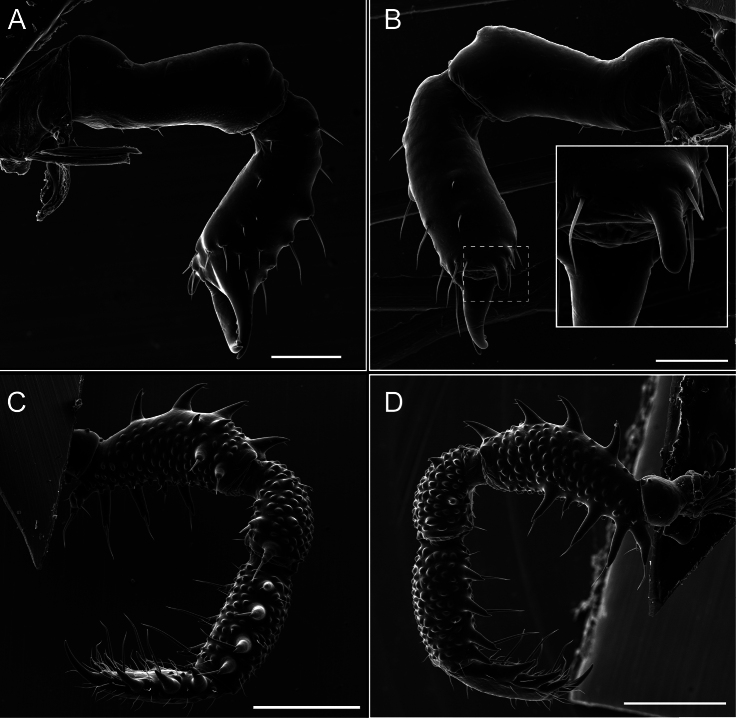
*Fresiaxmauryi* sp. nov. chelicerae: mesal **A** ectal **B** pedipalps: mesal **C** ectal **D**. Scale bars: 200 µm (**A, B**); 500 µm (**C, D**).

**Figure 20.**
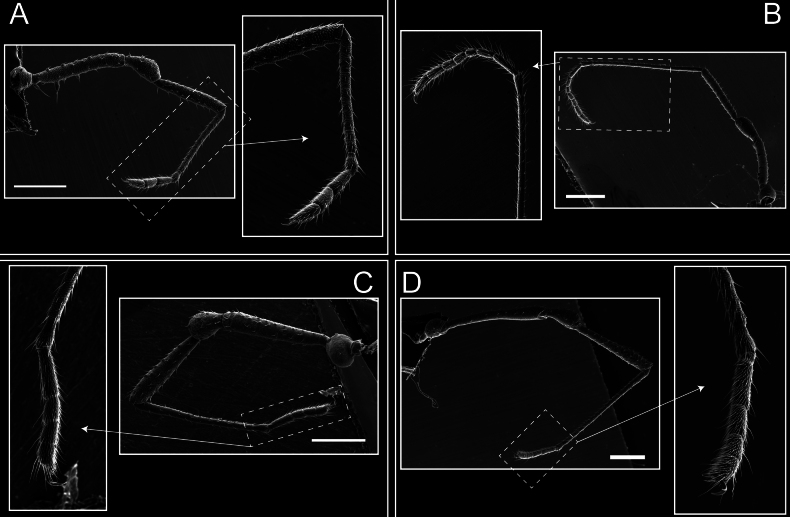
*Fresiaxmauryi* sp. nov. legs I **A** II **B** III **C** IV **D**. Scale bars: 500 µm.

**Figure 21.**
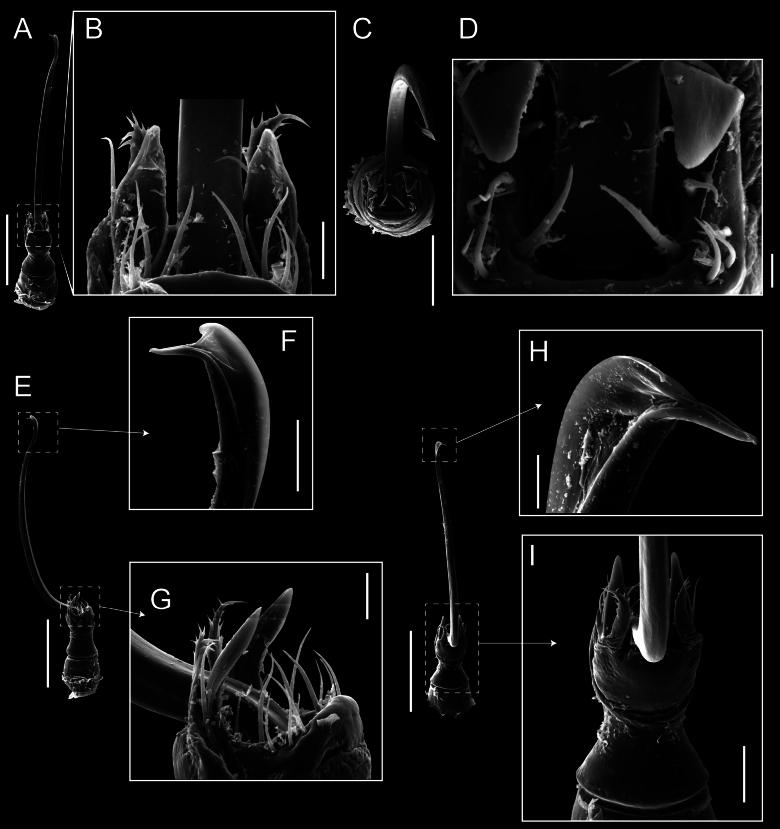
*Fresiaxmauryi* sp. nov. penis: ventral **A, B** apical **C, D** lateral **E, G** dorsal **H, I**. Scale bars: 500 µm (**A, E**); 100 µm (**C**); 20 µm (**D, H**); 50 µm (**B, F, G**).

**Figure 22.**
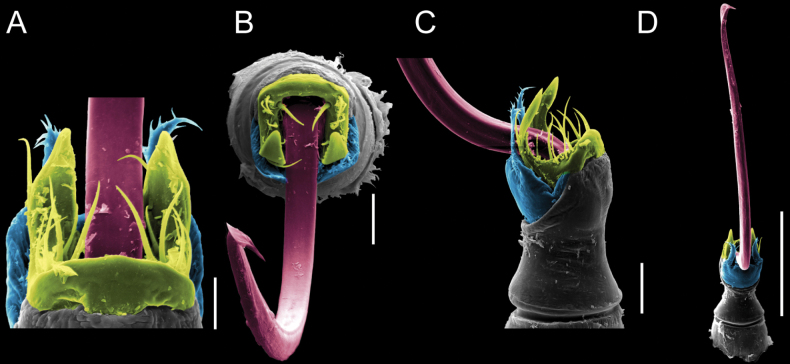
*Fresiaxmauryi* sp. nov. penis: ventral **A** apical **B** lateral **C** dorsal **D**. Colors: ventral plate (yellow), capsula externa (blue), capsula interna (red). Scale bars: 50 µm (**A**); 100 µm (**B, C**); 500 µm (**D**).

Figs 17 , 18 , 19 , 20 , 21 , 22

###### Material examined.

***Holotype*.** ♂ **Chile.** Monumento Natural Contulmo, 38.01501°S, 73.17981°W, 360 m, M. Ramírez & F. Labarque coll., 10.II.2005 (MNHNCL). ***Paratypes*. Chile.** Cautín: Lago Caburgua, 39.20749°S, 71.80529°W, S. Peck J. Peck coll., 15.XII.1984, 1 ♂ (AMNH). Llanquihue: P.N. Alerce Andino, Correntoso, sendero Huillifoten, 41.58235°S, 72.61738°W, 135 m, M. Ramírez & F. Labarque coll., 03.II.2005, 1 ♀ (MACN). Malleco, Monumento Natural Contulmo, 38.01501°S, 73.17981°W, E. Maury coll., 13.I.1989, 1 ♂ 1 ♀ (MACN). Malleco: P.N. Nahuelbuta, 37.81477°S, 72.9967°W, M. Ramírez & F. Labarque coll., 12.II.2005, 1 ♂ (MACN). Malleco: Monumento Natural Contulmo, 38.01501°S, 73.17981°W, 360 m, M. Ramírez & F. Labarque coll., 10.II.2005, 1 ♀ (MACN), P.N. Nahuelbuta, 37.81477°S, 72.9967°W, M. Ramírez & F. Labarque coll., 12.II.2005, 5 ♂ 13 ♀ 6 imm. (MACN).

###### Additional material.

Chile. Malleco, Monumento Natural Contulmo, 38.01624°S, 73.17942°W, 361 m, G. Giribet, G. Hormiga, A. Pérez-González coll. 13.XI.2014 (MACN). Same locality, 38.01625°S, 73.17902°W, M. Ramírez, M. Izquierdo, P. Michalik, C. Wirkner, K. Huckstorf coll., 09.II.2012, 1 imm. (MACN). Caramávida, San Alfonso, Quebrada Caramávida, San Alfonso, Reserva Arauco, 37.70942°S, 73.17107°W, 750 m, 15.I.2018, 1 ♀ (MACN), Quebrada Caramávida, “sector 9”, Reserva Arauco, 37.66839°S, 73.22683°W, 800 m, 16.I.2018 (MACN).

###### Etymology.

Patronym after the late Argentine arachnologist Emilio Maury, in honor of his contributions to the study of the Triaenonychidae (and Opiliones in general) of South America’s Southern Cone.

###### Diagnosis.

The prominent interocular apophysis, the carapace densely covered with rounded tubercles, the long tubercles of the mesotergum, the long drop-shaped genital operculum, the ectal-distal process of cheliceral segment II, and the hypertelic genitalia distinguish this species from all its congeners.

###### Distribution.

Chile: Araucanía Region (Fig. 4A).

###### Description of male.

Measurements: Total length 2.93, carapace length 0.97, dorsal scutum length 1.99, carapace max. width 1.40, mesotergum max. width 1.81. Appendage measurements: Pedipalps. Trochanter length 0.14, femora length 0.87, patella length 0.61, tibia length 0.66, tarsus length 0.56. Leg I: trochanter (tr) 0.25, femora (fe) 0.97, patella (pa) 0.42, tibia (ti) 0.73, metatarsus (mt) 0.90, tarsus (ta) 0.55. II: tr 0.29, fe 1.36, pa 0.43, ti 0.94, mt 1.55, ta 1.00. III: tr 0.28, fe 1.00, pa 0.41, ti 0.82, mt 1.24, ta 0.60. IV: tr 0.26, fe 1.33, pa 0.49, ti 1.11, mt 1.85, ta 0.72.

Dorsum (Fig. 17, 18). Eta (η) hourglass-shaped dorsal scutum. Ocularium raised, with a long backward-bending apophysis between the eyes, covered with rounded tubercles and with few setae on the apophysis. Carapace densely covered in rounded tubercles, mesotergum with areas delimited by strong tubercles. Areas I and II with straight rows of four and five setiferous tubercles, respectively, and III and IV with a row of eight setiferous tubercles (the two central ones are stronger than others). Posterior margin with a row of 12–14 setiferous tubercles. Free tergites with a row of setiferous tubercles similar to the posterior margin of the dorsal scutum.

Chelicerae (Fig. 19A, B). Segment I with a small, granulated area on the dorso-distal surface and two ventral granules. Segment II with an ectal-distal process that bears a few setae.

Pedipalps (Fig. 19C, D). Trochanter with three small dorsal granules, and two ventral and a small dorsal tubercle. Granules cover the femora, patella, tibia, and tarsus. Femora with a row of five spines with one subdistal seta, the proximal one bifurcated. Patella with three mesal spines and one ectal spine with subdistal setae. Tibia with four ectal and mesal spines with subdistal setae. Tarsus with three ectal and mesal spines with subdistal setae.

Legs (Fig. 20). Coxae I–IV bearing small setiferous tubercles, leg I with three long subdistal setiferous tubercles, the distal one forked at its terminal end. Spiracles visible. A smooth area occupies ¼ of legs II (which has a tubercle with subdistal seta) and III. The drop-shaped genital operculum is larger than all species of the genus. Opisthosomal sternites with a row of small setiferous tubercles. Sternum reduced due to the large size of the genital operculum.

Legs I–IV covered in setae; tarsal area and calcaneus densely covered in setae. Trochanter I with a small ventral tubercle. Tibia I with three proximal tubercles with setae, II–IV with sparse ventral granules. Calcaneus smaller than astragalus, ≥ 7× smaller in legs I, 9× (II), 8× (III), and 11× (IV). Tarsal count: 3–7–4–4.

Penis (Figs 21, 22). Pars distalis has a ventral plate with a notch that divides the plate into two halves, each with six long ventral macrosetae and one dorsal macroseta, a capsula externa shorter than the capsula interna, divided into two halves, and a finger-like apical region.

**Female.** Similar to male, with shorter pedipalpal femora and reduced genital operculum.

Female measurements. Total length 2.67, carapace length 0.80, dorsal scutum length 1.65, carapace max. width 1.25, mesotergum max. width 1.74. Appendage measurements: Pedipalps. Trochanter length 0.13, femora length 0.78, patella length 0.45, tibia length 0.56, tarsus length 0.53. Leg I: trochanter (tr) 0.19, femora (fe) 0.83, patella (pa) 0.35, tibia (ti) 0.66, metatarsus (mt) 0.80, tarsus (ta) 0.48. II: tr 0.27, fe 1.26, pa 0.43, ti 1.03, mt 1.45, ta 0.86. III: tr 0.23, fe 0.96, pa 0.38, ti 0.82, mt 1.15, ta 0.52. IV: tr 0.24, fe 1.29, pa 0.42, ti 1.04, mt 1.56, ta 0.64.

##### ﻿ Fresiax pichicuy sp. nov.

Taxon classificationAnimaliaOpilionesTriaenonychidae

B49E9E19-3490-5323-AF10-07D1C6B622A0

https://zoobank.org/58DB7CF4-A314-4B5F-9BB1-E7C4AF64C4CD

**Figure 23.**
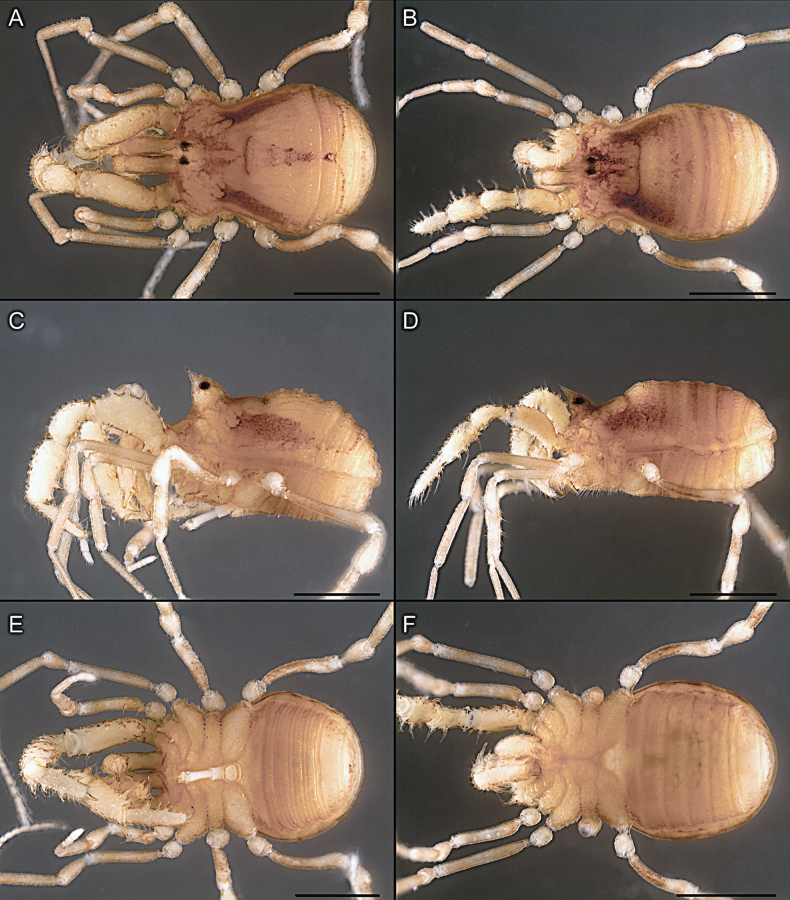
*Fresiaxpichicuy* sp. nov. habitus, male **A** dorsal view **C** lateral view **E** ventral view. Female **B** dorsal view **D** lateral view **F** ventral view. Scale bars: 1 mm. Species of Clade A, see Fig. 3.

**Figure 24.**
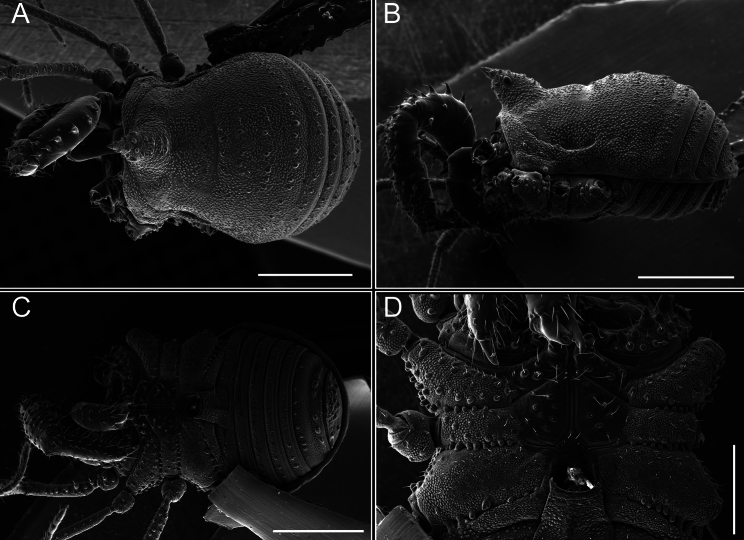
*Fresiaxpichicuy* sp. nov. male, SEM images of habitus **A** dorsal view **B** lateral view **C, D** ventral view. Scale bars: 1 mm (**A, B, C**); 500 µm (**D**).

**Figure 25.**
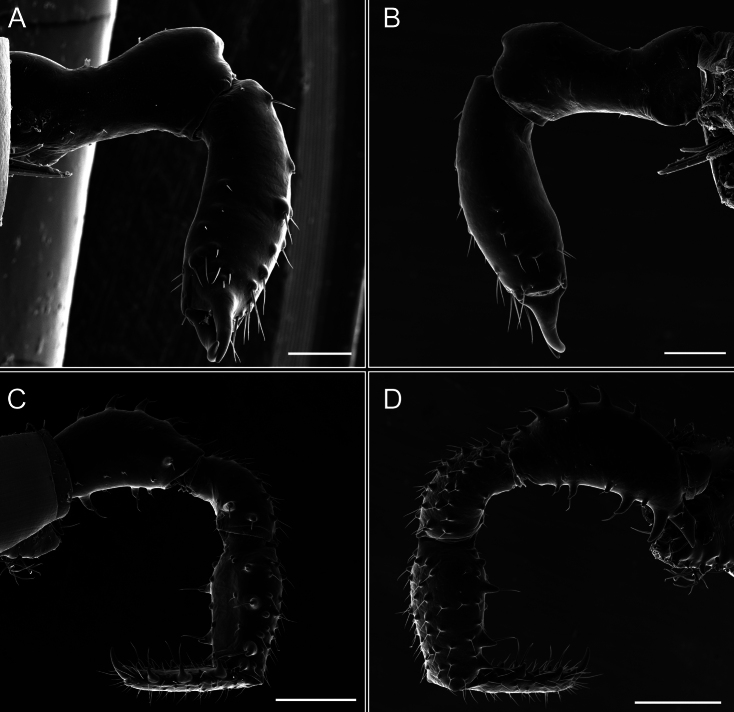
*Fresiaxpichicuy* sp. nov. chelicerae: mesal **A** ectal **B** pedipalps: mesal **C** ectal **D**. Scale bars: 200 µm (**A, B**); 500 µm (**C, D**).

**Figure 26.**
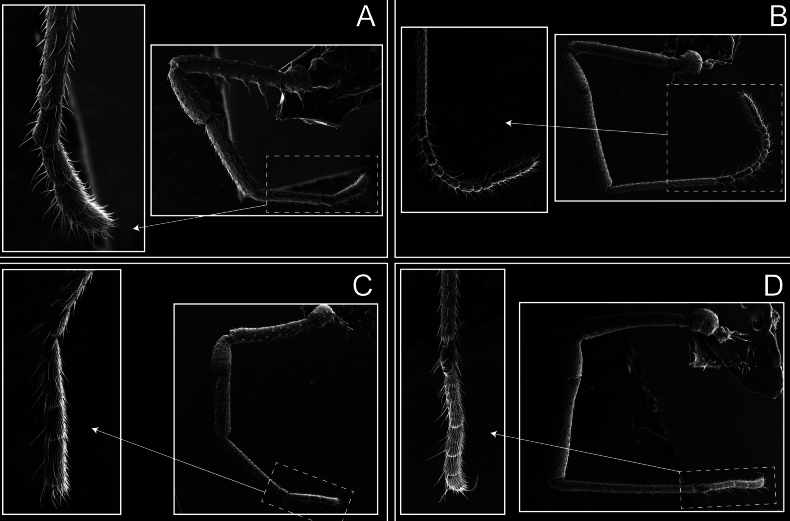
*Fresiaxpichicuy* sp. nov. legs I **A** II **B** III **C** IV **D**. Scale bars: 200 µm (**A, C**); 500 µm (**B, D**).

**Figure 27.**
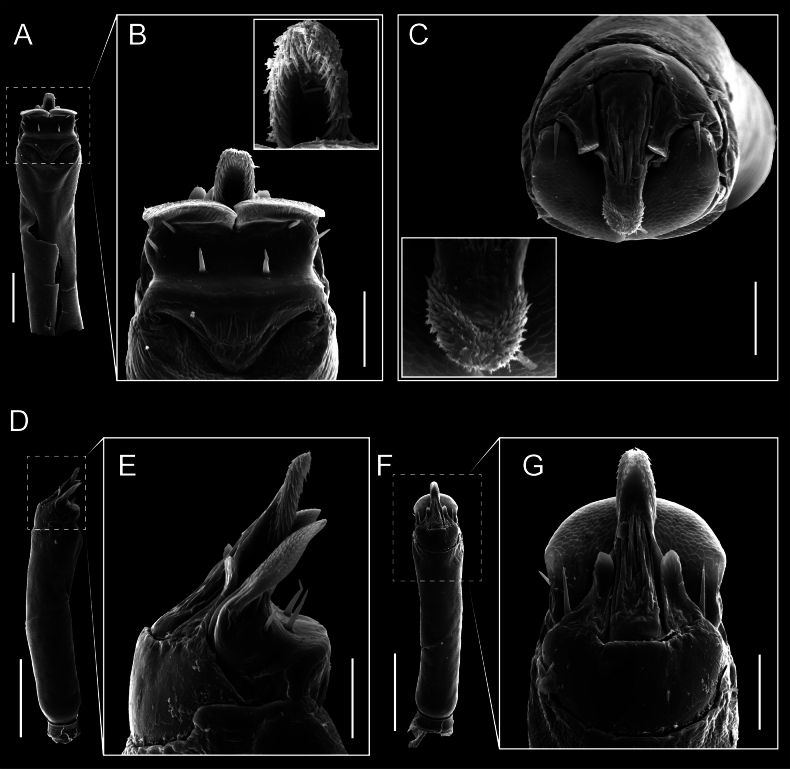
*Fresiaxpichicuy* sp. nov. penis: ventral **A, B** apical **C** lateral **D, E** dorsal **F, G**. Scale bars: 200 µm (**A, D, F**); 50 µm (**B, C, E, G**).

**Figure 28.**
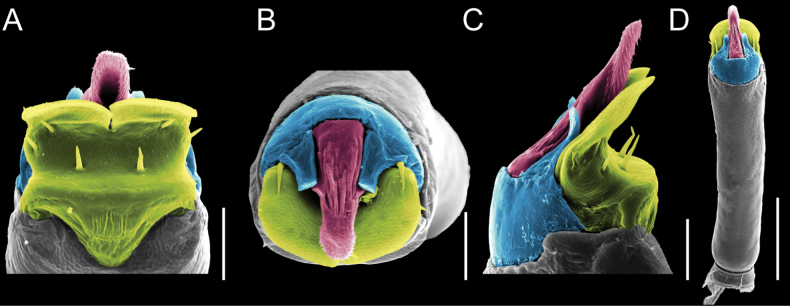
*Fresiaxpichicuy* sp. nov. penis: ventral **A** apical **B** lateral **C** dorsal **D**. Colors: ventral plate (yellow), capsula externa (blue), capsula interna (red). Scale bars: 50 µm.

Figs 23 , 24 , 25 , 26 , 27 , 28

###### Material examined.

***Holotype*.** ♂ **Chile.** Choapa: Pichidangui, Coquimbo, Fundo Palo Colorado, 16 km N of Pichidangui, E. Maury coll. 21.X.1988 (MNHNCL). ***Paratypes*.** Chile. Choapa: Pichidangui, Coquimbo, Fundo Palo Colorado, 16 km N de Pichidangui, E. Maury coll. 21.X.1988, 1 ♀ (MACN). Cuesta de Zapata, A. Porta coll., 2018, 11 imm. (MACN). Petorca: Cachagua, Quebrada El Tigre, E. Maury coll., 08.XII.1988, 5 ♂ 2 ♀ (MACN). Quillota: Parque Nacional La Campana, Palmas de Ocoa, E. Maury coll., 08.XII.1987, 2 ♀ (MACN). Petorca: Pichicuy, V región Valparaíso, Quebrada Huaquén, E. Maury coll., 29.X.1988, 2 ♂ 3 ♀ (MACN). Choapa, Los Vilos, Quebrada a Playa, Agua Dulce 46 km N de Los Vilos, E. Maury coll., 05.XII.1988, 1 ♀ (MACN). Quillota: Parque Nacional La Campana, Palmas de Ocoa, E. Maury coll., 27.X.1988, 2 ♂ 2 ♀ (MACN). Coquimbo: Limarí, Bosque Talinay, P.N. Fray Jorge, relict Valdivian fog forest, R. Schuh, N. Platnick coll., 08.II.1986, 2 ♂ (AMNH). Petorca: Pichicuy, Quebrada con Peumusboldus, A. Roig coll., 07.I.1984, 1 ♂ (MACN). Valparaíso, Cerro de La Campana, G. Betancourt coll., 12.III.1979, 1 ♀. Coquimbo: Limarí, P.N. Fray Jorge, relict Valdivian fog forest, N. Platnick, K. Catley, M. Ramírez, T. Allen coll., 10.XI.1993, 1 ♂ (AMNH). Petorca: Cachagua, V región Valparaíso, Quebrada El Tigre, E. Maury coll., 14.XII.1987, 7 imm. (MACN).

###### Etymology.

The species name derives from the species' distribution locality, Pichicuy, located in the commune of La Ligua, Petorca province, Chile. Noun in apposition.

###### Diagnosis.

This species can be easily distinguished from the other species in the genus by its conical forward-facing ocularium, with a 45 ° angled process. Dorsal scutum only covered in small granules. Capsula interna with digitiform structures on the apical portion. Genitalia similar to that of *F.fray*, slightly longer and with variations in the surface of the ventral plate and apex of the capsula interna with longer projections.

###### Distribution.

Chile: Valparaíso Region, Petorca Province (Fig. 4A).

###### Description of male.

Measurements: Total length 2.01, carapace length 0.64, dorsal scutum length 1.37, carapace max. width 1.03, mesotergum max. width 1.42. Appendage measurements: Pedipalps. Trochanter length 0.17, femora length 0.76, patella length 0.44, tibia length 0.67, tarsus length 0.54. Leg I: trochanter (tr) 0.16, femora (fe) 0.75, patella (pa) 0.36, tibia (ti) 0.51, metatarsus (mt) 0.54, tarsus (ta) 0.55. II: tr 0.19, fe 0.94, pa 0.39, ti 0.79, mt 0.87, ta 1.11. III: tr 0.24, fe 0.72, pa 0.26, ti 0.34, mt 0.65, ta 0.62. IV: tr 0.18, fe 0.99, pa 0.43, ti 0.82, mt 1.03, ta 0.71.

Dorsum (Fig. 23, 24). Eta (η) hourglass-shaped dorsal scutum. Conical ocularium, forward-looking, with a small forward-pointing apical spine and two dorsal rows of small setiferous tubercles. Eyes located high. Dorsal scutum microgranulate, without clear delimitation of areas. Areas I–IV with four, six, eight, and ~ 17 small rounded setiferous tubercles with setae, respectively. Posterior border and free tergites with a row of small rounded setiferous tubercles. All free tergites with a row of small setiferous granules.

Chelicerae (Fig. 25A, B). Segment I smooth; segment II with setae and sparse granules.

Pedipalps (Fig. 25C, D). Trochanter smooth. Femora with a row of six dorsal spines with subdistal setae, a row of four ventral spines with setae interspersed by small tubercles with subdistal setae, the proximal one subtriangular in shape, a row of four small mesal granules with setae, and a small distal tubercle with mushroom-shaped tubercles. Patella covered in small tubercles with setae on the dorsal surface, with two small mesal setiferous tubercles. Tibia with four ectal and mesal spines with subdistal setae, nine ventral granules with setae, and dorsal surface covered in small tubercles with setae. Tarsus with three mesal and ectal spines, as well as a few setae and granules.

Legs (Fig. 26). Coxa I with small setiferous tubercles and a row of three long tubercles with subdistal setae, coxae II–IV microgranulate, bearing five or six bridges between legs II and III, eight between III and IV, 6–8 between leg IV and opisthosoma. Spiracles not visible. Smooth area occupies 1/3 of leg II, almost ½ of III, and only a small proximal portion of IV. Smooth area II with five small setiferous tubercles, III with eight small setiferous tubercles. Opisthosomal sternites III with four small setiferous tubercles on each side, IV with three small setiferous tubercles on each side, and V with an anterior row of small setiferous tubercles and a posterior row of rounded setiferous tubercles. Anal plate covered in small setiferous tubercles. Trochanter I with three small ventral and one dorsal setiferous tubercle; tibia I with a row of five tubercles with setae and a dorsal row of small setiferous tubercles; femora III with ~ 20 small setiferous tubercles. Legs II–IV covered in setae, tarsal area, and calcaneus densely setose. Calcaneus smaller than astragalus, ≥ 5× smaller in legs I, 6× (II), 7× (III) and 10× (IV). Tarsal count 3–9–4–4.

Penis (Figs 27, 28). Pars distalis with a ventral plate bearing a cleft dividing the plate into two halves. Ventral surface of each half with three macrosetae and dorsal surface with one macroseta; ventral plate covered in scale-like structures. Capsula externa shorter than capsula interna, having a notch that divides the capsula externa into two halves.

**Female.** Similar to male, with shorter pedipalpal femora.

Female measurements. Total length 1.93, carapace length 0.60, dorsal scutum length 1.34, carapace max. width 1.07, mesotergum max. width 1.45. Appendage measurements: Pedipalps. Trochanter length 0.15. Femora length 0.65, patella length 0.34, tibia length 0.53, tarsus length 0.49. Leg I: trochanter (tr) 0.15, femora (fe) 0.62, patella (pa) 0.34, tibia (ti) 0.51, metatarsus (mt) 0.53, tarsus (ta) 0.50. II: tr 0.18, fe 0.79, pa 0.37, ti 0.70, mt 0.81, ta 1.03. III: tr 0.18, fe 0.68, pa 0.31, ti 0.57, mt 0.67, ta 0.49. IV: tr 0.20, fe 0.94, pa 0.36, ti 0.72, mt 0.87, ta 0.62.

##### ﻿ Fresiax spinulosa

Taxon classificationAnimaliaOpilionesTriaenonychidae

(Maury, 1990) comb. nov.

B6DE539A-1346-5A82-BAE9-78A91E1F89FD

**Figure 29.**
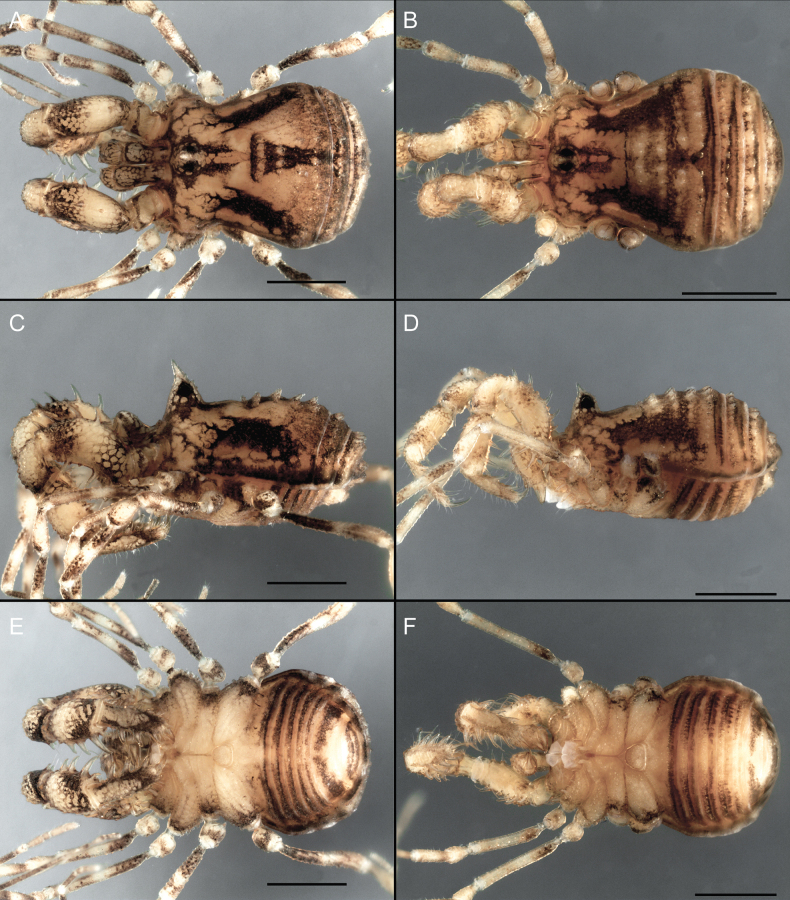
*Fresiaxspinulosa* comb. nov. habitus, male **A** dorsal view **C** lateral view **E** ventral view. Female **B** dorsal view **D** lateral view **F** ventral view. Scale bars: 1 mm. Species of Clade A, see Fig. 3.

**Figure 30.**
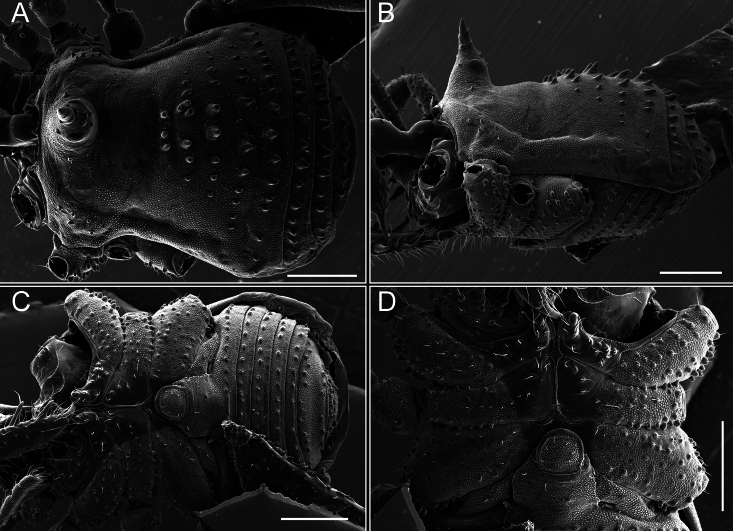
*Fresiaxspinulosa* comb. nov. male, SEM images of habitus **A** dorsal view **B** lateral view **C, D** ventral view. Scale bars: 500 µm.

**Figure 31.**
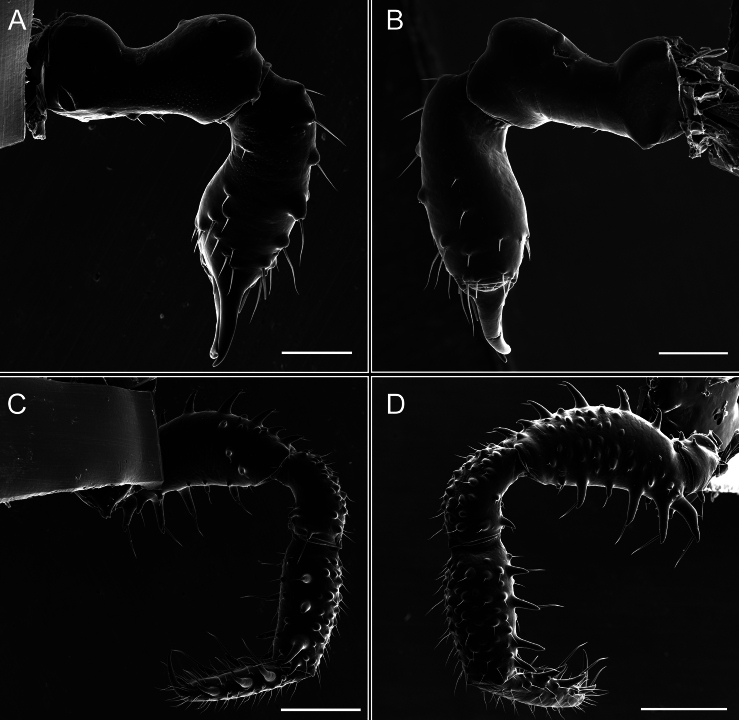
*Fresiaxspinulosa* comb. nov. chelicerae: mesal **A** ectal **B** pedipalps: mesal **C** ectal **D**. Scale bars: 200 µm (**A, B**); 500 µm (**C, D**).

**Figure 32.**
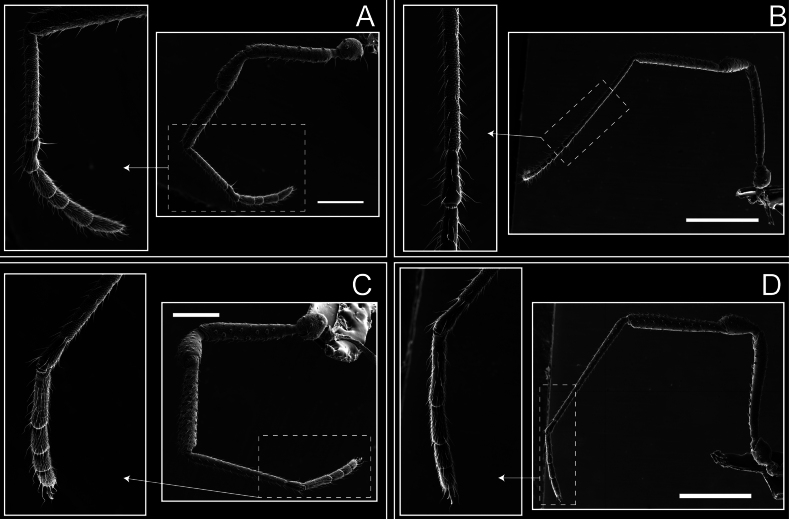
*Fresiaxspinulosa* comb. nov. legs I **A** II **B** III **C** IV **D**. Scale bars: 500 µm (**A, C**); 100 µm (**B, D**).

**Figure 33.**
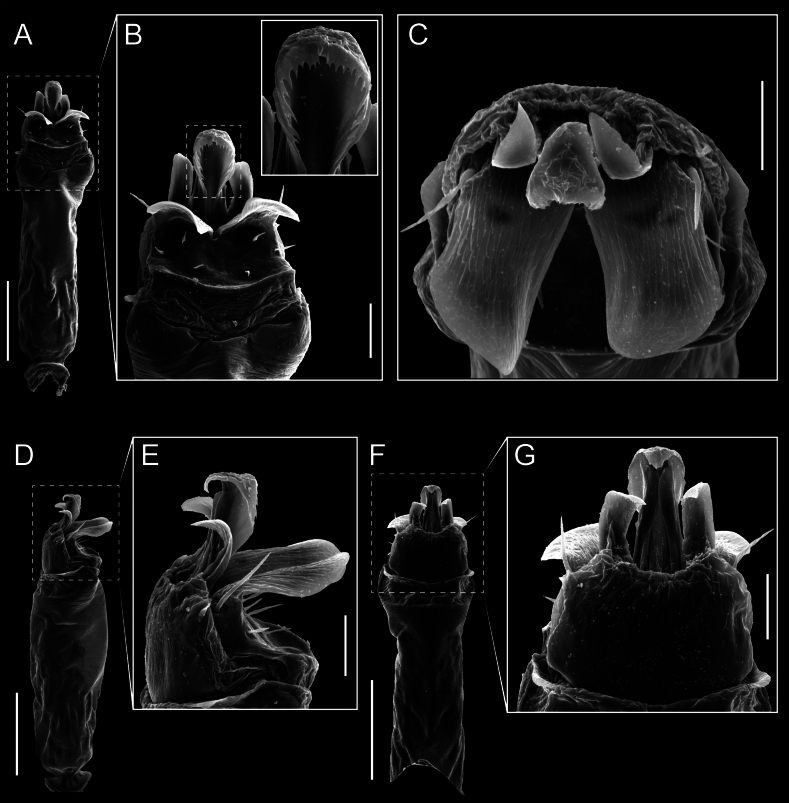
*Fresiaxspinulosa* comb. nov. penis: ventral **A, B** apical **C** lateral **D, E** dorsal **F, G**. Scale bars: 200 µm (**A, D, F**); 50 µm (**B, C, E, G**).

**Figure 34.**
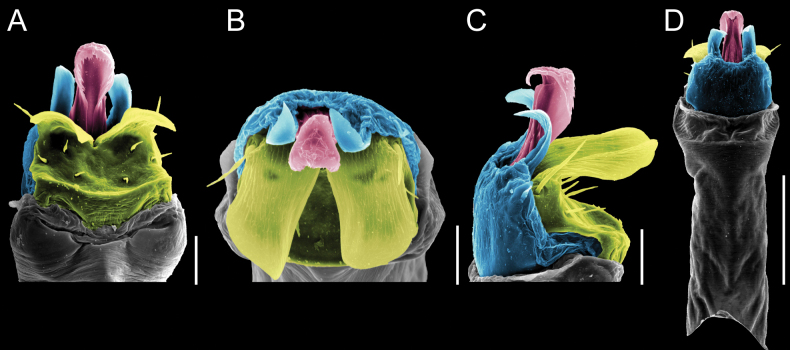
*Fresiaxspinulosa* comb. nov. penis: ventral **A** apical **B** lateral **C** dorsal **D**. Colors: ventral plate (yellow), capsula externa (blue), capsula interna (red). Scale bars: 50 µm (**A–C**); 200 µm (**D**).

Figs 29 , 30 , 31 , 32 , 33 , 34

Nuncia spinulosa Maury, 1990: 108, figs 13–24; Maury 1992: 5; Acosta and Maury 1998: 579; Kury 2003: 22.

###### Material examined.

***Holotype*.** ♂ **Argentina.** Neuquén Province, Hua Hum, E. Maury coll. (MACN 8689). ***Paratype* (allotype** ♀) **. Argentina.** Neuquén Province, Hua Hum, E. Maury coll. (MACN 8690). ***Paratypes*. Argentina.** Tromen Lake, Neuquén Province, 3 ♂ and 4 ♀ (MACN 8691).

###### Additional material.

Argentina. Neuquén: Parque Nacional Nahuel Huapi, Lago Ortiz Basualdo, M. Ramírez coll., I.1990, 2 ♀ 1 imm. (MACN). Río Negro: Parque Nacional Nahuel Huapi, Río Frías Superior, M. Ramírez coll., I.1990, 1 ♀ (MACN), D. Anghicante. coll., 26.01.1990, 1 ♀ 1 imm. Río Negro: P.N. Nahuel Huapi, Puerto Blest, M. Ramírez V. Werenkraut, S. Aysén coll., 28.XII.2010, 1 ♂ 2 ♀, Near Puerto Alegre, Lago Frías, M. Ramírez, V. Werenkraut, S. Aysén coll., 29.XII.2010, 1 ♂, A. Quaglino, L. Lopardo coll., 07.I.2000, 1 ♀. Neuquén: P.N. Nahuel Huapi, Puerto Blest, M. Ramírez coll., 05.I.1998, 1 ♀ (MACN). Río Negro: Bariloche, Lago Mascardi, Near Tronador Hotel, E. Maury coll., 02.XI.1986, 1 ♂ 2 imm. (MACN). Neuquén: Parque Nacional Nahuel Huapi, Los Lagos, M. Ramírez coll., 30. I.1985, 1 ♀ (MACN), Base Glaciar Frias, D. Anghicante coll., 24.I.1988, 1 ♂ 1 ♀. Chile. Valdivia: Res. Valdivia, B. Borry coll., I.2007, 4 imm. Cautín: M. Ramírez, F. Labarque coll., 08.II.2005, 1 ♂ 1 imm. (MACN); Bellavista, Fundo Flor del Lago, M. Ramírez, F. Labarque coll., 09.II.2005, 1 ♀ (MACN). Osorno: Termas de Puyehue, R. Schuh, N. Platnick coll., 24–25.XI.1981, 4 ♂ 1 ♀ (AMNH), 36 km W. La Union, L. Peña coll., 25.III.1987, 2 ♂ (AMNH). Valdivia: Nehuin, X Reg. Los Lagos, E. Maury coll., 16.I.1989, 1 ♂ 1 ♀ 1 imm. (MACN). Chiloé: Chiloé island, 5 km N of Quellón, R. Schuh, N. Platnick coll., 01.XII.1981, 1 ♂ (AMNH). Osorno: 7 km E de Entrelagos, Camping “No me olvides”, E. Maury coll., 30.I.1991, 1 ♂ 1 imm. (MACN). Llanquihue: P.N. Alerce Andino, M. Ramírez, F. Labarque coll., 02.IV.2005, 2 ♂ (MACN). Valdivia: Corral, X Reg. Los Lagos, Río Nahuelan 24 km E Corral, E. Maury coll., 16.I.1989, 1 ♂ 1 ♀ (MACN). Chiloé: 25 km N de Chepu, M. Ramírez coll., 08.II.1991, 1 ♀ (MACN). Valdivia: Niebla, Camping “La Herradura”, 8 km east form Niebla, E. Maury coll., 23.I.1991, 1 ♂ 2 ♀ 1 imm. (MACN). Concepción: Hualpén, Estación de Biología terrestre Univ. de Concepción, A. Ojanguren, A. Pérez-González, M. Ramírez, G. Azevedo, W. Porto coll., 14.I.2018, 1 ♂ (MACN). Valdivia: Parque Oncol, Sendero Punucahua, M. Ramírez, E. Soto, J. Wilson, D. Poy coll., 13.I.2020, 1 ♀ (MACN). Concepción: Hualpén, Estación de Biología terrestre Univ. de Concepción, A. Ojanguren, A. Pérez-González, M. Ramírez, G. Azevedo, W. Porto coll., 14.I.2018, 1 ♂ 1 ♀ (MACN). Valdivia: Res. Valdivia, B. Borry coll., I.2007, 1 imm. Cautín: Pucón, Ojos del Caburgua, 15 km NE de Pucón, E. Maury coll., 16.I.1987, 1 ♂ 2 ♀ 3 imm. (MACN), Termas de Palguin, SE de Pucón, E. Maury coll., 17.I.1987, 1 ♀ (MACN). Chiloé: Chepu, E. Maury coll., 11.XII.1985, 1 ♂ (MACN). Malleco: Malalcahuello, E. Maury coll., 08.01.1987, 3 ♂ 1 ♀ (MACN). Chiloé: Cucao, E. Maury coll., 12.XII.1985, 2 ♂ 1 ♀ (MACN). Llanquihue: Puerto Montt, Carretera Austral, Caleta La Arena, E. Maury coll., 07.VIII.1985, 3 ♂ 3 ♀ (MACN). Osorno: Los Derrumbes, 5 km al S de Termas Puyehue, E. Maury coll., 03.I.1988, 1 ♀ (MACN). Same collector, 04.XII.1985, 5 ♂ 5 ♀ 8 imm. (MACN), 36 km W. La Union, 600 m, L. Peña coll., 25–28.III.1987, 1 ♂ (AMNH). Valdivia: Las Lajas (Las Trancas), W. La Unión, L. Peña coll., 19.XI.1990, 1 ♂ (AMNH).

###### Diagnosis.

This species can be easily distinguished from the other species in the genus by its long interocular apophysis (not as long as in *F.mauryi*), the long mesotergal tubercles, and the penis with longer ventral plate than other species in the genus.

###### Distribution.

Argentina: Provinces of Neuquén and Río Negro. Chile: Regions of Araucanía, Los Ríos, and Los Lagos (Fig. 4A).

###### Description of male.

Measurements: Total length 3.29, carapace length 0.77, dorsal scutum 2.22 length. max. width of the carapace 1.72. max. width of the mesotergum 2.01. Appendage measurements: Pedipalps. Trochanter length 0.28, femora length 1.27, patella length 0.64, tibia length 1.05, tarsus length 0.95. Leg I: trochanter (tr) 0.30, femora (fe) 1.13, patella (pa) 0.58, tibia (ti) 0.92, metatarsus (mt) 1.03, tarsus (ta) 0.76. II: tr 0.34, fe 1.71, pa 0.63, ti 1.47, mt 1.75, ta 1.55. III: tr 0.36, fe 1.17, pa 0.51, ti 1.13, mt 1.44, ta 0.97. IV: tr 0.41, fe 1.56, pa 0.71, ti 1.47, mt 1.98, ta 1.10.

Dorsum (Fig. 29, 30). Eta (η) hourglass-shaped dorsal scutum. Ocularium elevated, accompanied by a long apophysis located between the eyes. Additionally, there are two dorsal rows of setae present in the ocularium. Carapace with fine microgranulation, small tubercles located on each side of the ocular region. A group of five small tubercles can be observed near leg I. Mesotergum with fine microgranulation and features areas that are delineated by prominent tubercles. These tubercles create triangular patterns on the dorsal surface of the mesotergum. Areas I–IV with rows of three, nine, ten, and 12 setiferous tubercles, respectively. The two central tubercles of rows II and III more robust than the others. Posterior margin of the mesotergum with a row of 16 setiferous tubercles. Free tergites, similarly to the posterior margin of the dorsal scutum, display a row of setiferous tubercles.

Chelicerae (Fig. 31A, B). Segment I with two ventral granules and a tiny granule on the dorso-distal surface. Segment two with small granules.

Pedipalps (Fig. 31C, D). The pedipalps exhibit several distinctive features. Trochanter of the pedipalp possesses one ventral and three small dorsal setiferous tubercles. Femora and tibia covered with granules, visible in both dorsal and ectal views. Femora of the pedipalp particularly notable, with a series of six ventral spines with subdistal setae. Proximal process subtriangular in shape, while the fourth and sixth spines are smaller compared to the others. On the mesal surface of the femora, a row of small tubercles of varying sizes can be observed. Additionally, three small distal tubercles with setae and 6–8 granules are present. Dorsal surface of the femora with a row of six spines with subdistal setae. Patella with two mesal and one ectal small spines, each adorned with subdistal setae. In ventral view, tibia displays four ectal and mesal spines with subdistal setae, with a row of setiferous granules. Tarsus with three mesal and ectal tubercles, all covered in subdistal setae.

Legs (Fig. 32). Legs I to IV characterized by short setiferous tubercles. Leg I with three long tubercles with subdistal setae. Legs II and III are connected by four or five bridges, while leg IV is connected to the opisthosoma by 6–8 bridges. Spiracles not covered by these bridges. The smooth surface occupies < ¼ of leg IV, indicating the presence of microgranulation or other textural variations. Legs II and III each with ~ 1/3 of their surfaces covered in a row of four small tubercles. Leg II with seven small tubercles. On the opisthosomal sternites, a row of tiny setiferous tubercles can be observed. Sternum arrow-shaped, with a triangular-shaped posterior. Femora I–IV bearing small setiferous tubercles, metatarsus I with a distal notch.

Penis (Figs 33, 34). Pars distalis with a ventral plate that bears a groove dividing it into two long lamellae. Each lamella has three small macrosetae on the ventral surface and one macroseta on the dorsal surface, and the ventral plate is composed of scale-like structures. Capsula externa shorter than capsula interna, with a groove dividing the dorsal fold into two halves. Capsula interna with apical projections on the ventral view.

**Female.** Similar to male, with shorter pedipalpal femora.

Female measurements. Total length 3.10, carapace length 0.94, dorsal scutum length 2.00, carapace max. width 1.48, mesotergum max. width 1.95. Appendage measurements: Pedipalps. Trochanter length 0.21, femora length 1.00, patella length 0.61, tibia length 0.74, tarsus length 0.80. Leg I: trochanter (tr) 0.28, femora (fe) 1.02, patella (pa) 0.48, tibia (ti) 0.84, metatarsus (mt) 0.95, tarsus (ta) 0.68. II: tr 0.34, fe 1.55, pa 0.59, ti 1.37, mt 1.54, ta 1.34. III: tr 0.31, fe 1.16, pa 0.48, ti 0.93, mt 1.26, ta 0.78. IV: tr 0.38, fe 1.42, pa 0.61, ti 1.31, mt 1.79, ta 1.02.

#### ﻿Clade B

##### ﻿Genus Mistralia gen. nov.

Taxon classificationAnimaliaOpilionesTriaenonychidae

21C9A3A0-0B9D-56C6-9A6B-006ED064C10E

https://zoobank.org/B295CFCF-0BC2-4562-B880-C549E61289C7

Figs 35 , 36 , 37 , 38 , 39 , 40 , 41 , 42 , 43

Nuncia [part] (only references to Nunciaverrucosa): Maury 1990: 106; Maury 1992: 5; Acosta and Maury 1998: 579; Kury 2003: 22.

###### Etymology.

The generic epithet is a reference to the Chilean poet, diplomat, and educator Gabriela Mistral (1889–1957). Feminine grammatical gender.

###### Diagnosis.

It differs from all other genera in Triaenonychidae by the morphology of the male genitalia, where the capsula interna features a lateral plate formed by a projection of the pars basalis onto the pars distalis. The dorsal scutum is covered in sharp tubercles (*M.ramirezi* sp. nov.) or wart-shaped (*M.verrucosa*). Ocularium with an apical apophysis.

###### Type species.

*Nunciaverrucosa* Maury, 1990

###### Included species.

*Mistraliaramirezi* sp. nov., *Mistraliaverrucosa* comb. nov.

###### Distribution.

Argentina: Neuquén, Río Negro. Chile, Regions: Bío-Bío, Los Lagos (Fig. 4B).

##### ﻿ Mistralia ramirezi sp. nov.

Taxon classificationAnimaliaOpilionesTriaenonychidae

46F123B6-812A-5B26-801A-A17577DDFB0A

https://zoobank.org/28A61E8F-6EC9-4908-A88C-791AB46769B5

Figs 35 , 36 , 37

###### Material examined.

***Holotype*.** ♂ **Chile.** Malleco: Monumento Natural Contulmo, 38.01314°S, 73.18648°W, M. Ramírez, F. Labarque coll. 10.II.2005 (MNHNCL).

###### Etymology.

Patronym in honor to Argentine arachnologist, Martín Ramírez, esteemed colleague and friend, for his contributions to the field of spider taxonomy and systematics.

###### Diagnosis.

This species can be easily distinguished from the other species in the genus by having sharp tubercles on the surface of the dorsal scute and by its unique genitalia, with a U-shaped capsula externa in dorsal view.

###### Distribution.

Chile: Bío-Bío Region (Fig. 4B).

###### Description of male.

Measurements: Total length 3.29, carapace length 0.77, dorsal scutum length 2.22, carapace max. width 1.72. Appendage measurements: Pedipalps. Trochanter length 0.28, femora length 1.27, patella length 0.64, tibia length 1.05, tarsus length 0.95. Leg I: trochanter (tr) 0.37, femora (fe) 0.30, patella (pa) 1.13, tibia (ti) 0.58, metatarsus (mt) 0.92, tarsus (ta) 1.03. II: tr 0.76, fe 0.34, pa 1.71, ti 0.63, mt 1.47, ta 1.75. III: tr 1.55, fe 0.36, pa 1.17, ti 0.51, mt 1.13, ta 1.44. IV: tr 0.97, fe 0.41, pa 1.56, ti 0.71, mt 1.47, ta 1.98.

**Figure 35.**
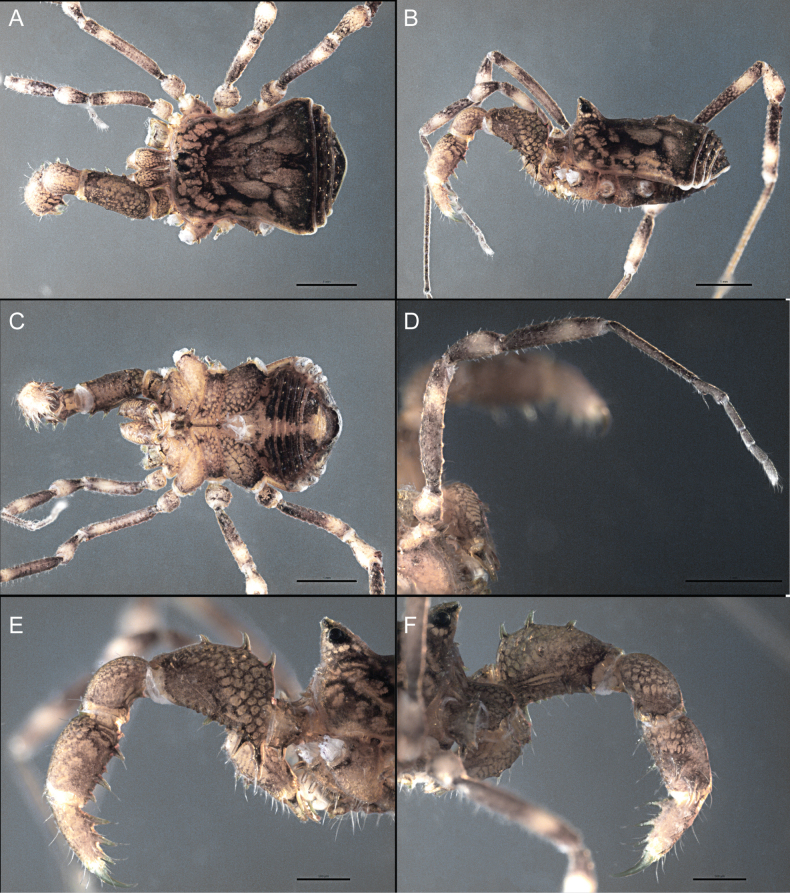
*Mistraliaramirezi*. sp. nov. habitus, male **A** dorsal view **B** lateral view **C** ventral view **D** leg I **E** pedipalp ectal **F** pedipalp mesal. Scale bars: 1 mm (**A–D**); 500 µm (**E, F**).

Dorsum (Fig. 35). Eta (η) hourglass-shaped dorsal scutum. Ocularium elevated, with apophysis between eyes. Carapace smooth; mesotergum with areas delimited by tubercles. Areas I–IV characterized by two setiferous tubercles: posterior margin with a row of ca. 10 setiferous tubercles. Free tergites bear a row of setiferous tubercles, similar to those found on the posterior margin of the dorsal scutum.

Chelicerae. Segment II with two prominent tubercles and few granules.

Pedipalps. Femora and tibia covered with granules when viewed from dorsal and ectal perspectives. Femora with a row of three distinct ventral and dorsal spines. Patella with two mesal tubercles and one ectal tubercle, each accompanied by subdistal setae. In ventral view, tibia with four ectal and mesal spines with subdistal setae, as well as with a row of setiferous granules. Tarsus characterized by three mesal and ectal spines covered with subdistal setae.

Legs (Fig. 35D). Ventral surface: I–IV with small setiferous tubercles. Sternum arrow-shaped.

**Figure 36.**
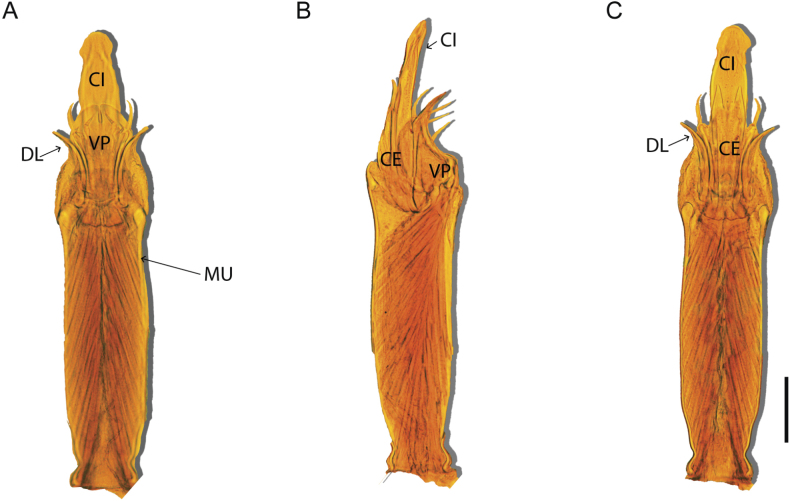
*Mistraliaramirezi* sp. nov. Penis ventral **A** lateral **B** dorsal **C**. Capsula interna (CI), Capsula externa (CE), dorsolateral plate (DL), ventral plate (VP), muscle (MU). Scale bar: 100 µm.

**Figure 37.**
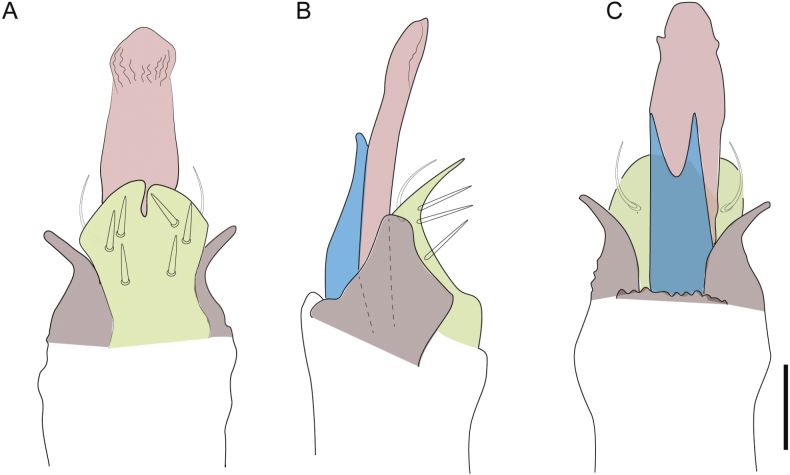
*Mistraliaramirezi* sp. nov. Penis ventral **A** lateral **B** dorsal **C** capsula interna (red), Capsula externa (blue), dorsolateral plate (brown), ventral plate (green). Scale bar: 100 µm.

Penis (Figs 36, 37). Pars distalis with a ventral plate divided into two lamellae by a small cleft. Each lamella on the ventral surface bears three tiny macrosetae, while the dorsal surface has one macroseta. Capsula externa with a U-shaped slit, shorter in length compared to the capsula interna, which is tubular in shape.

**Female.** Unknown.

##### ﻿ Mistralia verrucosa

Taxon classificationAnimaliaOpilionesTriaenonychidae

(Maury, 1990) comb. nov.

7AC2EBF5-070D-5DAA-A45C-249FB2FE7B9A

**Figure 38.**
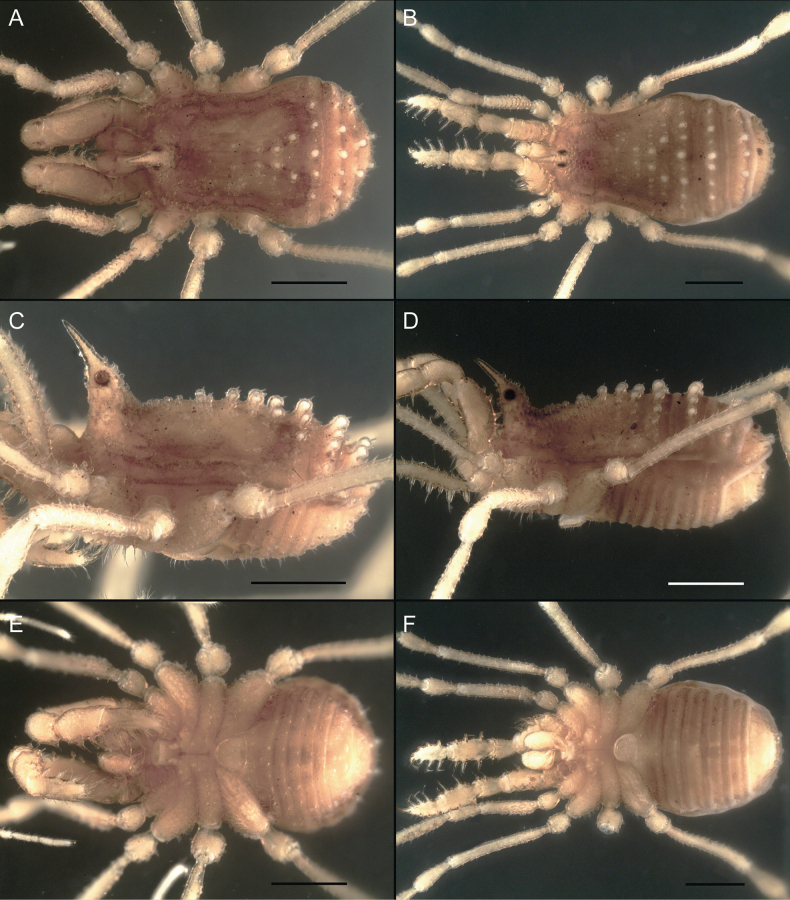
*Mistraliaverrucosa* comb. nov. habitus, male **A** dorsal view **C** lateral view **E** ventral view. Female **B** dorsal view **D** lateral view **F** ventral view. Scale bars: 1 mm. Species of Clade B, see Fig. 3.

**Figure 39.**
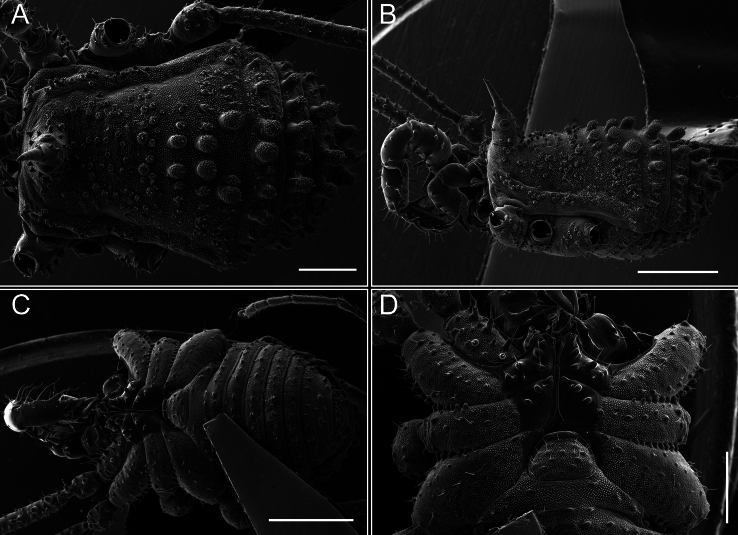
*Mistraliaverrucosa* comb. nov. male, SEM images of habitus **A** dorsal view **B** lateral view **C, D** ventral view. Scale bars: 1 mm (**A, B, C**); 500 µm (**D**).

**Figure 40.**
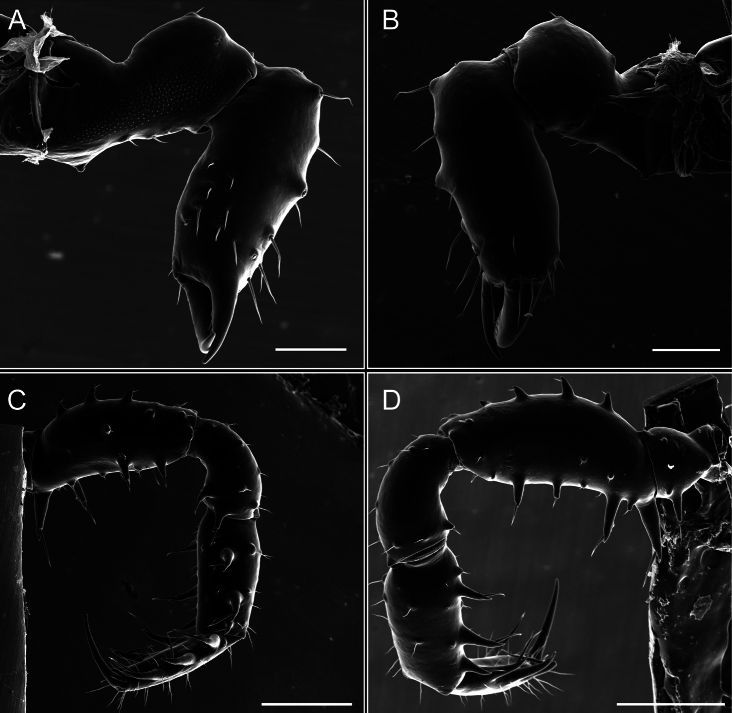
*Mistraliaverrucosa* comb. nov. chelicerae: mesal **A** ectal **B** Pedipalps: mesal **C** ectal **D**. Scale bars: 200 µm (**A, B**); 500 µm (**C, D**).

**Figure 41.**
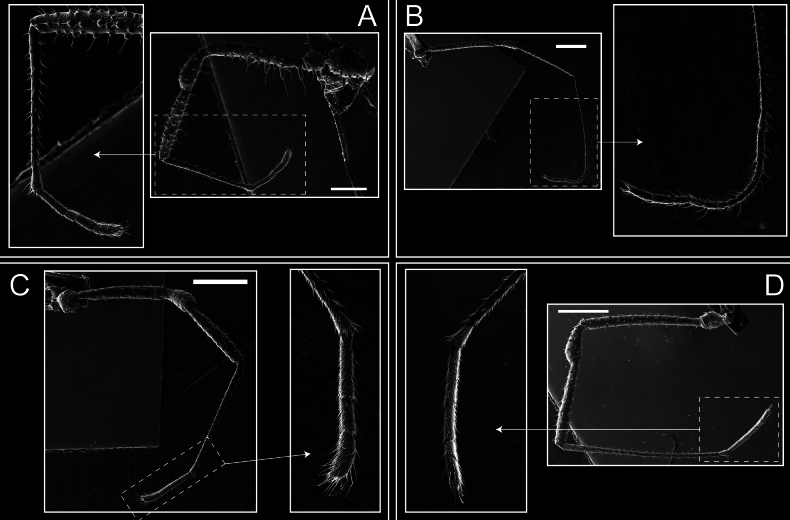
*Mistraliaverrucosa* comb. nov. legs I **A** II **B** III **C** IV **D**. Scale bars: 1 mm.

**Figure 42.**
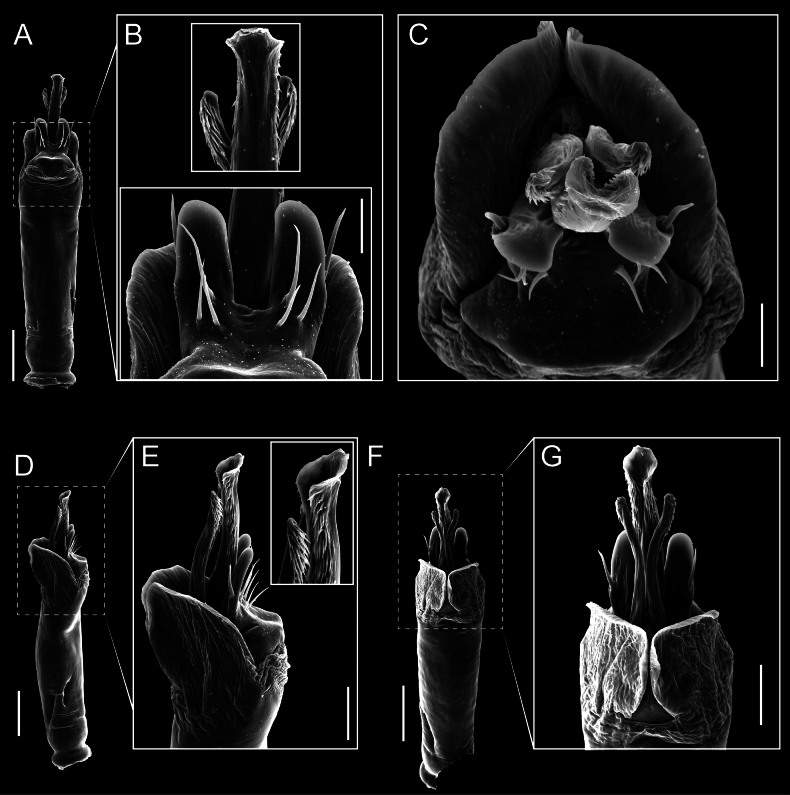
*Mistraliaverrucosa* comb. nov. penis: ventral **A, B** apical **C** lateral **D, E** dorsal **F, G**. Scale bars: 200 µm (**A, D, F**); 100 µm (**B, E, G**); 50 µm (**C**).

**Figure 43.**
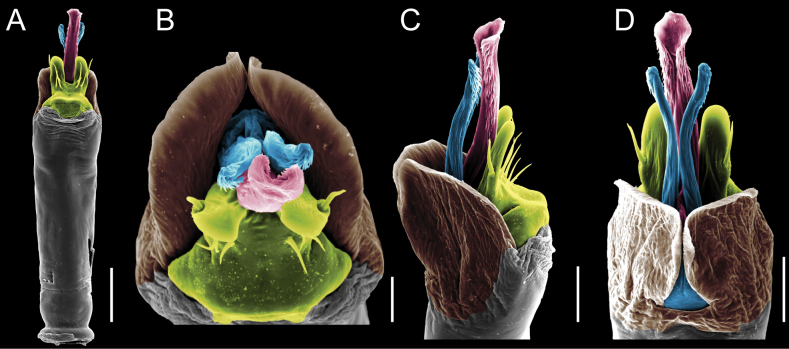
*Mistraliaverrucosa* comb. nov. penis: ventral **A** apical **B** lateral **C** dorsal **D**. Colors: ventral plate (yellow), capsula externa (blue), capsula interna (red). Scale bars: 200 µm (**A**); 50 µm (**B**); 100 µm (**C, D**).

Figs 38 , 39 , 40 , 41 , 42 , 43

Nuncia verrucosa Maury, 1990: 106, figs 1–12; Maury 1992: 5; Acosta and Maury 1998: 579; Kury 2003: 22; Pérez-Schultheiss et al. 2021: 413, fig. 3c, f.

###### Material examined.

***Holotype*.** ♂ **Chile.** Termas del Río Amarillo, Palena Province, E. Maury coll., 04.XII.1986 (MACN 8685). ***Paratype* (allotype** ♀) **. Chile.** Los Derrumbes, Puyehue, Osorno Province, E. Maury coll., 04–05.XII.1985 (MACN 8686).

***Paratypes*. Chile.** Río Palena, Aysén Province, 1 ♂ and 1 ♀, E. Maury coll., 06–07.XII.1986 (MACN 8687).

###### Additional material.

Argentina. Río Negro: Parque Nacional Nahuel Huapi, Río Frías Superior, D. Anghicante coll., 26.I.1990, 2 imm. Neuquén: Lago Ortiz Basualdo, M. Ramírez coll., I.1990, 1 ♂ (MACN), P.N. Nahuel Huapi, Puerto Blest, M. Ramírez coll., 10.I.1998, 1 ♂ 1 ♀ 1 imm. (MACN). Osorno: Los Derrumbes, 5 km al S de Termas Puyehue, A. Roig coll., I.1988, 1 ♂ (MACN). Same locality, E. Maury coll., 18.I.1989, 1 ♂ (MACN). Los Derrumbes, 5 km al S de Termas Puyehue, 40.73807°S, 72.31114°W, 536 m, E. Maury coll., 04.12.1985, 1 ♀ (MACN).

###### Diagnosis.

This species can be readily distinguished from other species within the genus by its distinctive ocularium with a prominent elongated process. The tubercles on the dorsal scutum are wart-like. The dorsal plate of the penis is divided into two elongated structures with small apical projections.

###### Distribution.

Argentina: Provinces of Neuquén and Río Negro. Chile: Los Lagos Region (Fig. 4B).

###### Redescription of male.

Measurements: Total length 3.60, length of carapace 1.16, length of dorsal scutum 2.70, max. width of carapace 1.74. max. width of mesotergum 2.00. Appendage measurements: Pedipalps. Length of trochanter 0.28, length of femora 1.15, length of patella 0.56, length of tibia 0.87, length of tarsus 0.91. Leg I: trochanter (tr) 0.36, femora (fe) 1.62, patella (pa) 0.66, tibia (ti) 1.27, metatarsus (mt) 1.44, tarsus (ta) 0.91. II: tr 0.39, fe 2.4, pa 0.73, ti 1.97, mt 2.57, ta 1.97. III: tr 0.37, fe 1.78, pa 0.63, ti 1.49, mt 2.17, ta 1.05. IV: tr 0.44, fe 2.52, pa 0.73, ti 1.77, mt 3.36, ta 1.12.

Dorsum (Fig. 38, 39). Eta (η) hourglass-shaped dorsal scutum. Anterior margin with 2–3 small setiferous tubercles on each side. Ocularium raised, with a long spine between eyes, covered in setiferous tubercles and with few setae on the spine. Carapace with fine microgranulation; adorned with warts-shaped tubercles, while the mesotergum microgranulate. Areas I–IV with setiferous tubercles, with area I having a pair of wart-shaped tubercles, and areas II–IV exhibiting 2–3 robust wart-shaped tubercles. Posterior margin adorned with a row of six wart-like tubercles. Free tergites also with a row of wart-shaped tubercles similar to those found on the posterior margin of the dorsal scutum.

Chelicerae (Fig. 40A, B). Segment I with few setae. Segment II with few granules.

Pedipalps (Fig. 40C, D). Trochanter with dorsal and ectal granules and a ventral spine with a seta. Ventral surface of femora with a row of four spines with subdistal setae and three small setiferous tubercles. In ectal view, there are 4–5 setiferous granules, while the dorsal surface features a row of three spines with subdistal setae. In mesal view, there are two distal tubercles with setae, with a row of setiferous granules on the top and bottom of the mesal surface. Dorsal surface of the patella with a few setiferous granules, while the mesal view exhibits two spines with setae, and the ectal view has setiferous granules. Tibia with setiferous granules on the dorsal and ventral surfaces, in addition to six ventral spines with subdistal setae. Tarsus with three mesal and ectal spines with subdistal setae.

Legs (Fig. 41). I with three rows of small tubercles and one row of long tubercles with subdistal setae, II and III with one row of small tubercles each, and IV exhibiting microgranulation, with six bridges between coxa II–III, 8–9 eight or nine bridges between coxa III and IV, and three or four between coxa IV and opisthosoma. Spiracles visible. The smooth surface covers ~ ¼ of leg II (which features three tubercles with setae) and III and occupies < ¼ of the IV coxa (with a row of 2–3 small tubercles and a process directed towards the sternum). The opisthosomal sternites possess a row of small setiferous tubercles. Sternum arrow-shaped.

Trochanters I–IV bearing small dorsal setiferous tubercles, trochanter I with a ventral tubercle with setae. Femora I–IV bearing small setiferous tubercles. Femora I with a ventral row of remarkable spines with setae. Tarsal count: 5–10/12–4–4.

Penis (Figs 42, 43). Pars distalis equipped with a ventral plate featuring a long cleft, dividing it into two lamellae. Each lamella with three small macrosetae on its ventral surface and one macroseta on the dorsal surface. Capsula externa shorter in length compared to the capsula interna, divided into two cylindrical structures adorned with numerous small apical denticles. Capsula interna tubular in shape, with dorsal-apical denticles.

**Female.** Similar to the male, with shorter pedipalpal femora and without interocular apophysis.

Female measurements. Total length 3.15, length of carapace 1.52, length of dorsal scutum 2.62, max. width of carapace 1.73, max. width of mesotergum 2.28. Appendage measurements: Pedipalps. Length of trochanter 0.27, length of femora 1.21, length of patella 0.75, length of tibia 0.90, length of tarsus 1.26. Leg I: trochanter (tr) 0.34, femora (fe) 1.60, patella (pa) 0.73, tibia (ti), 1.24, metatarsus (mt) 1.51, tarsus (ta) 0.81. II: tr 0.45, fe 2.42, pa 0.83, ti 2.00, mt 2.65, ta 1.98. III: tr 0.45, fe 1.77, pa 0.75, ti 1.37, mt 2.12, ta 1.07. IV: tr 0.39, fe 2.59, pa 0.89, ti 1.88, mt 3.48, ta 1.36. Tarsal count: 4–9(10)–4–4.

#### ﻿Clade C

##### ﻿Genus Chilenuncia

Taxon classificationAnimaliaOpilionesTriaenonychidae

Muñoz-Cuevas, 1971, nom. rest.

4AB2C1DC-FD5E-5048-966B-FC9B952A2480

Figs 44 , 45 , 46 , 47 , 48 , 49 , 50 , 51 , 52 , 53 , 54 , 55

Chilenuncia Muñoz-Cuevas, 1971: 873; Cekalovic 1985: 11 [considered syn. jr. of Nuncia Loman, 1902, by Maury 1990].
Parattahia [part]: Soares 1968: 266; Cekalovic 1985: 11; Maury and Roig Alsina 1985: 78.
Nuncia [part]: Maury 1990: 103, 105; Kury 2003: 21.

###### Diagnosis.

It is distinguished from all other South American Triaenonychidae by the unique morphology of the male genitalia, characterized by a capsula externa that covers nearly the entire capsula interna, with a small apical cleft. The external morphology, particularly the presence of a small curved apophysis in the ocularium, allows for differentiation from other South American triaenonychid genera, although it bears resemblance to the Australian genus *Calliuncus*, which forms part of Clade C.

###### Type species.

*Chilenunciadonosoi* Muñoz-Cuevas, 1971 (syn. jr. of *Parattahiachilensis* Soares, 1968)

###### Included species.

*Chilenunciachilensis* comb. nov., *Chilenunciarostrata* comb. nov.

###### Distribution.

Argentina: Neuquén, Río Negro. Chile, Metropolitan Region of Santiago, O’Higgins, Maule, Ñuble, Bío-Bío, Araucanía, Los Ríos, Los Lagos, and Aysén Regions (Fig. 4C).

##### ﻿ Chilenuncia chilensis

Taxon classificationAnimaliaOpilionesTriaenonychidae

(Soares, 1968) comb. nov.

44F77C3A-8578-5D3F-860B-64ECFF1C7095

**Figure 44.**
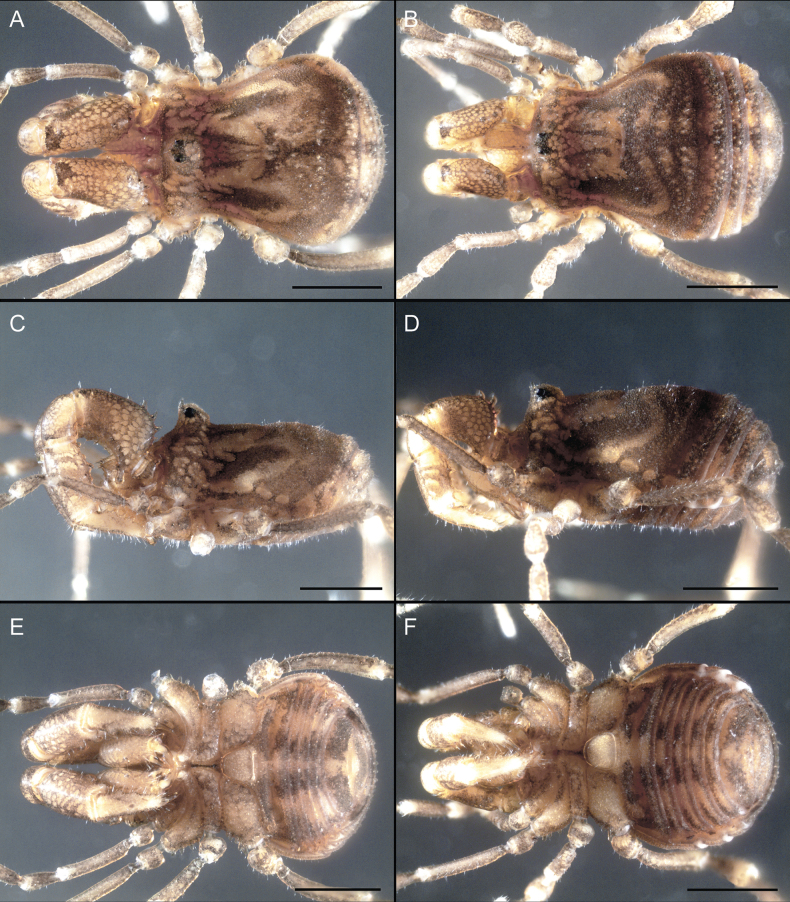
*Chilenunciachilensis* comb. nov. habitus, male **A** dorsal view **C** lateral view **E** ventral view. Female **B** dorsal view **D** lateral view **F** ventral view. Scale bars: 1 mm. Species of Clade C, see Fig. 3.

**Figure 45.**
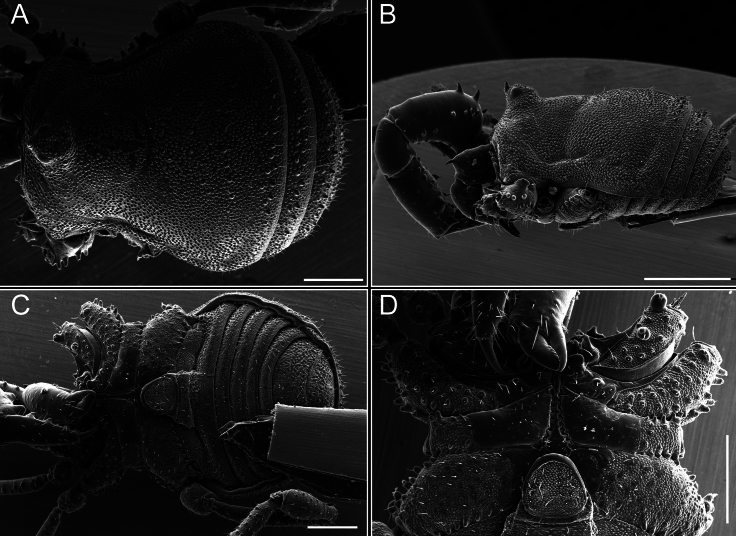
*Chilenunciachilensis* comb. nov. male, SEM images of habitus **A** dorsal view **B** lateral view **C, D** ventral view. Scale bars: 500 µm.

**Figure 46.**
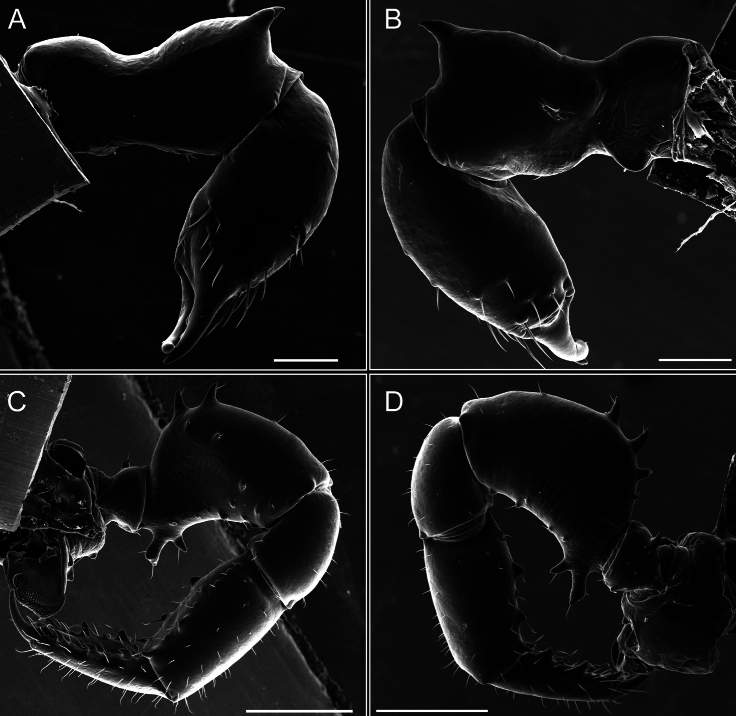
*Chilenunciachilensis* comb. nov. chelicerae: mesal **A** ectal **B** Pedipalps: mesal **C** ectal **D**. Scale bars: 200 µm (**A, B**); 500 µm (**C, D**).

**Figure 47.**
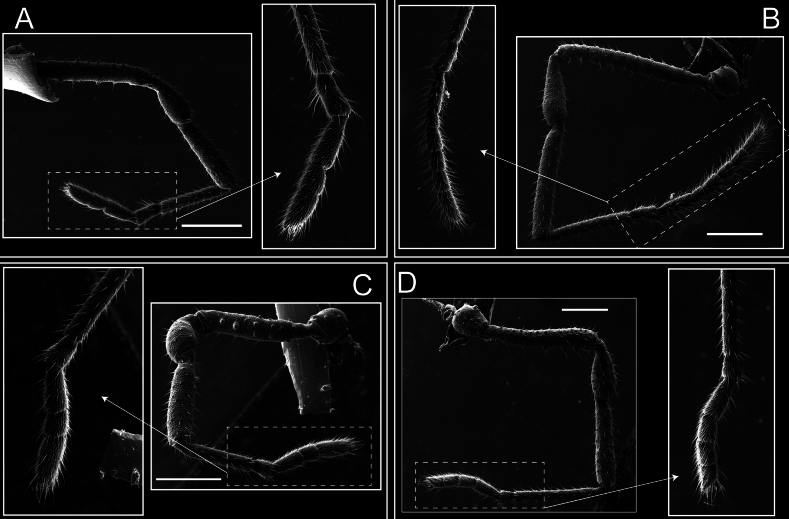
*Chilenunciachilensis* comb. nov. legs I **A** II **B** III **C** IV **D**. Scale bars: 500 µm.

**Figure 48.**
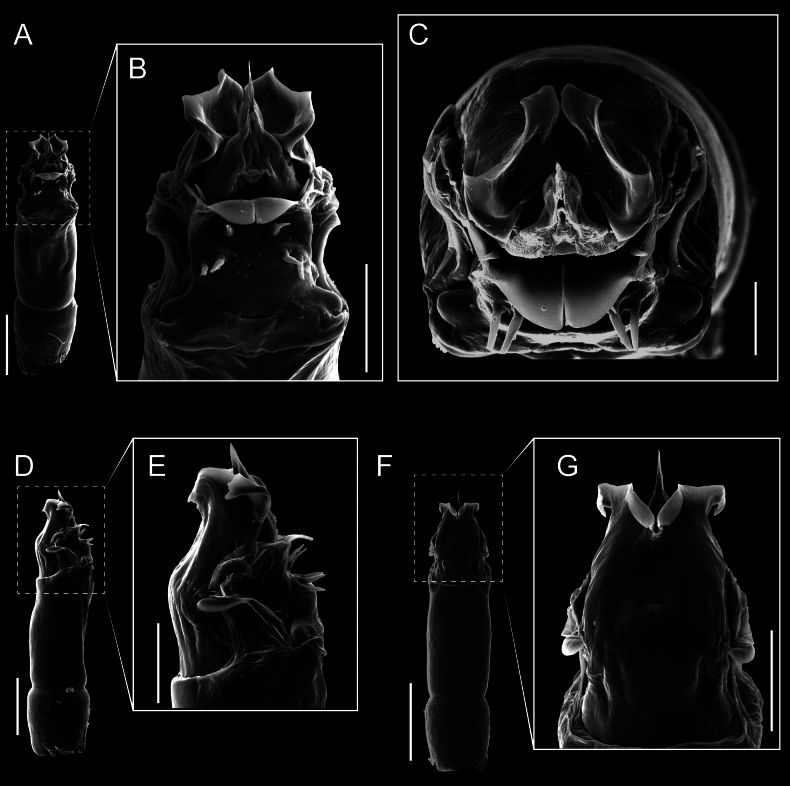
*Chilenunciachilensis* comb. nov. penis: ventral **A, B** apical **C** lateral **D, E** dorsal **F, G**. Scale bars: 200 µm (**A, D, F**); 100 µm (**B, E, G**); 50 µm (**C**).

**Figure 49.**
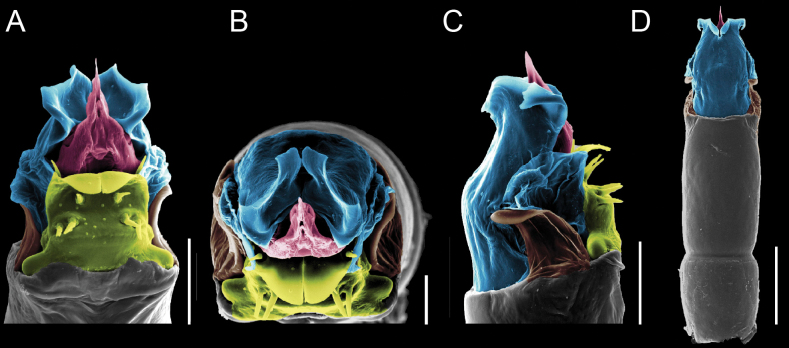
*Chilenunciachilensis* comb. nov. penis: ventral **A** apical **B** lateral **C** dorsal **D**. Colors: ventral plate (yellow), capsula externa (blue), capsula interna (red). Scale bars: 100 µm (**A, C, D**); 50 µm (**B**).

Figs 44 , 45 , 46 , 47 , 48 , 49

Parattahia chilensis Soares, 1968: 266, figs 13, 14; Muñoz-Cuevas 1971: 873, fig. 28; Cekalovic 1985: 11.
Nuncia chilensis : Maury 1990: 105; Acosta and Maury 1998: 579; Kury 2003: 21.
Chilenuncia donosoi Muñoz-Cuevas, 1971: 874, figs 1–28; Cekalovic 1985: 11. [Synonymy established by Maury, 1990].

###### Material examined.

Chile. Osorno: Puyehue, Anticura Sector, Parque Nacional Puyehue, Sendero Pionero, M. Ramírez, F. Labarque coll., 06.II.2005, 1 imm. (MACN). Llanquihue: Alerce Andino, Correntoso, M. Ramírez, F. Labarque coll., 03.II.2005, 1 imm. (MACN). Talca: RN Altos del Lircay, E Vilches Alto, A. Ojanguren, A. Pérez-González, M. Ramírez, G. Azevedo, W. Porto coll., 11.I.2018, 6 ♂ 9 ♀ 2 imm. (MACN). Cachapoal: Reserva Nacional Río de los Cipreses, near the El potrero, A. Ojanguren, A. Pérez-González, M. Ramírez, G. Azevedo, W. Porto coll., 09.I.2018, 1 ♀ 2 imm. (MACN). Curicó: El Potrero Grande, El Relvo, J. Barriga coll., 08.V.2004, 1 ♀ (MACN). Arauco: P.N. Nahuelbuta, Pichinahuel, J. Barriga coll., 22.XI.2004, 1 ♂ (MACN). Osorno: Camping “No me olvides”, 7 km E de Entrelagos, E. Maury coll., 30.I.1991, 9 ♂ 7 ♀ 16 imm. (MACN). Puerto Cárdenas, T. Cekalovic coll., 31.I.1982, 1 imm. (MACN). Curicó: Los Queñes, E. Maury coll., 15.I.1984, 1 ♀ 1 imm. (MACN). Coyhaique: 10 km N de Coyhaique, Reserva Nacional, S. Peck, J. Peck coll., 22.I.1985, 1 ♀ (AMNH). Frutillar Bajo, Univ. Chile Forest Res., S. Peck J. Peck coll., 22.XII.1984, 1 ♀ (AMNH). Palena: Near Chaitén, R.Schuh, N. Platnick coll., 05.XII.1981, 1 ♀ 3 imm. (AMNH); Chaitén, N. Platnick, R.Schuh coll., 07.XII.1981, 2 ♀ 1 imm. (AMNH); 25–27 km N Chaitén, N. Platnick, P. Goloboff, R.Schuh coll., 17.I.1986, 1 imm. (AMNH), Termas Río Amarillo, E. Maury coll., 04.XI.1986, 2 ♂ 1 ♀ (MACN), 11 km O de Río Negro, E. Maury coll., 08.XII.1985, 9 ♂ 1 ♀ (MACN). La Cabaña, A. Hidalgo coll., 01.II.1982, 1 imm. Cautín: Bellavista, Fundo Flor del Lago, M. Ramírez, F. Labarque coll., 09.II.2005, 4 ♂ 1 ♀ 1 imm. (MACN), 3 ♂ 2 imm. (MACN). Llanquihue: Los Muermos, E.Ross, A.Michelbacher coll., 20. I.1951, 2 ♀; Frutillar, G. Kuschel coll., 29.IX.1954, 5 imm. Alerce Andino, Correntoso, M. Ramírez, F. Labarque coll., 03.II.2005, 6 imm. (MACN), 7 km N Enseada, N. Platnick, R.Schuh coll., 26.XI.1981, 1 imm. (AMNH), Lago Chapo, 11.7 km E Correntoso, A. Newton, M. Thayer coll., 16.XII.1982, 1 imm. (AMNH), 10–14 km E Correntoso, N. Platnick, O. Francke coll., 03.II.1985, 1 imm. (AMNH), Carretera Austral, Caleta La Arena, E. Maury coll., 07.XII.1985, 15 imm. (MACN); Playa Magui, 7 km NE Frutillar, E. Maury coll., 27.I.1991, 11 imm. (MACN); P.N. Alerce Andino, N. Platnick, K. Catley, M. Ramírez, T. Allen coll., 23.XI.1993, 1 imm. (AMNH). Argentina. Neuquén: 4 km W de Poracá, L. Herman coll., 21.I.1972, 2 imm., Road between Pucará y Laguna Venados, L. Herman coll., 25.I.1972, 4 imm., Hua Hum, E. Maury coll., 19.I.1983, 1 ♂ (MACN), Lago Huechulafquen, M. Ramírez coll., 07.I.1985, 1 imm. (MACN); Hua Hum, E. Maury coll., 17.I.1985, 2 imm. (MACN); Río Pucará S shore, Lago Lacar, 8 km E Hua Hum, N. Platnick, R.Schuh coll., 13.I.1986, 1 imm. (AMNH), Lago Tromen, E. Maury coll., 18.I.1987, 1 imm. (MACN), Nahuel Huapi, E. Maury coll., 23.XI.1987, 2 imm. (MACN). Chile. Chiloé: 5 km de Chepu, T.Cekalovic coll., 23.II.1968, 3 imm. (MACN), Cruce camino a San Pedro, T.Cekalovic coll., 26.II.1976, 8 imm. (MACN); Chepu, N. Platnick, R. Schuh coll., 29.XI.1981, 1 imm. (AMNH); 5 km N Quellón, N. Platnick, R.Schuh coll., 01.XII.1981, 2 imm. (AMNH), Piruquina, T. Cekalovic coll., 09.II.1983, 1 imm. (MACN), Isla Chiloé, 8 km S Ancud, S.Peck, J.Peck coll., 01.II.1985, 2 imm. (AMNH); Chepu, E. Maury coll., 11.XII.1985, 1 imm. (MACN); Cucas, E. Maury coll., 12.XII.1985, 5 imm. (MACN). Cautín: Parque Nacional Villarrica, M. Ramírez, F. Labarque coll., 08.II.2005, 6 imm. (MACN); Bellavista, Lago Villarrica, N. Platnick, K. Catley, M. Ramírez, T. Allen coll., 20.XI.1993, 1 imm. (AMNH); Huerquehue, Laguna Toro, M. Ramírez, F. Labarque coll., 02.VII.2005, 1 imm. (MACN), Volcán Villarrica, site 654, A. Newton, M. Thayer coll., 15.XII.1982, 7 imm. (AMNH), site 653, A. Newton, M. Thayer coll., 15.XII.1982, 1 imm. (AMNH), Molco, T. Cekalovic coll., 22.II.1983, 1 imm. (MACN), 15 km NE Villarrica, Flor del Lago, S. Peck, J. Peck coll., 01.XI.1985, 7 imm. (AMNH); Bellavista, Lago Villarrica, N. Platnick, O. Francke coll., 28.I.1985, 15 imm. (AMNH), R. Schuh, N. Platnick coll., 30.X.1986, 13 imm. (AMNH); Molco Alto, T.Cekalovic coll., 18.II.1986, 3 imm. (MACN); Ojos del Caburgua, 15 km NE de Pucón, E. Maury coll., 16.I.1987, 3 imm. (MACN), Termas de Palguin, SE de Pucón, E. Maury coll., 17.I.1987, 11 imm. (MACN). Maule: Talca, Parque Nacional Gil de Vilches, N. Platnick, P. Goloboff, M. Ramírez coll., 08.II.1992, 1 imm. (AMNH). Talca: Vilches, E. Maury coll., 16.1.1984, 3 imm. (MACN). Same collector, 07.I.1989, 10 imm. (MACN). Las Tacitas, Bordon coll., II.1972, 1 imm. Talca: A. Roig coll., 17.I.1984, 3 imm. (MACN). Maule: Curicó, Los Queñes, P. Goloboff, K. Catley coll., 17.X.1992, 2 imm. (AMNH). Malleco: Parque Nacional Nahuelbuta, M. Ramírez, F. Labarque coll., 12.II.2005, 2 ♂ 3 ♀ 4 imm. (MACN), E. Schlinger, E. Irwin coll., 09.IX.1966, 1 ♀ 1 imm., Monumento Nacional Contulmo, M. Ramírez, F. Labarque coll., 10.II.2005, 1 ♂ 1 ♀ (MACN), Puren Contulmo Nat. Mon, S.Peck, J.Peck coll., 13.II.1985, 1 ♂ (AMNH); Malalcahuello, E. Maury coll., 08.I.1987, 2 ♂ 1 ♀ (MACN); Parque Nacional Nahuelbuta, E. Maury coll., 23.XII.1985, 2 ♂ 1 ♀ (MACN). Arauco: Caramávida, E. Maury coll., 16.XII.1985, 2 ♂ 5 ♀ 7 imm. (MACN). Ñuble: 2 km E de Las Trancas, E. Maury coll., 09.I.1989, 4 ♂ 1 ♀ (MACN). Malleco: Monumento Natural Contulmo, E. Maury coll., 10.I.1987, 3 ♀ (MACN), same collector, 13.I.1989, 2 ♂ 1 imm. (MACN), Parque Nacional Tolhuaca, 2 km E Lago Malleco, A. Newton, M. Thayer coll., 01.I.1983, 1 ♀ (AMNH). Osorno: 36 km W. La Union, L. Peña coll., 25.III.1987, 2 imm. (AMNH). Ñuble: 22.7 km ESE Recinto, site 646, A. Newton, M. Thayer coll., 10.XII.1982, 1 imm. (AMNH). Valdivia: 26 km SE Panguipulli, S. Peck, J. Peck coll., 16.XII.1984, 1 imm. (AMNH). Osorno: Águas Calientes, P. N. Puyehue, N. Platnick, R.Schuh coll., 28.I.1986, 1 imm. (AMNH), Hills of Maicolpue, N. Platnick, P. Goloboff, M. Ramírez coll., 19.II.1992, 1 imm. (AMNH), 10 km E of Bahia Mansa, N. Platnick, O. Francke coll., 30.I.1985, 4 imm. (AMNH); Puyehue, Anticura Sector, Parque Nacional Puyehue, Sendero Pionero, M. Ramírez, F. Labarque coll., 05.II.2005, 10 imm. (MACN); Antillanca road, 6–8 km SE Aguas Calientes, P. N. Puyehue, N. Platnick, R.Schuh coll., 28.I.1986, 3 imm. (AMNH), Termas de Puyehue, N. Platnick, R.Schuh coll., 24.XI.1981, 2 imm. (AMNH). Valdivia: Lago Calafquen, T.Cekalovic coll., 18.II.1977, 2 imm. (MACN). Osorno: Los Derrumbes, 5 km S de Termas de Puyehue, E. Maury coll., 09.I.1988, 5 imm. (MACN). Valdivia: Río Nahuicán, 24 km SE Corral, E. Maury coll., 16.I.1989, 5 imm. (MACN). Osorno: Anticura, E. Maury coll., 09.I.1988, 3 imm. (MACN). Valdivia: Pirehucico, E. Maury coll., 01.XII.1985, 10 imm. (MACN). Osorno: Los Derrumbes, E. Maury coll., 04.XII.1985, 9 imm. (MACN). Valdivia: Niebla, Camping “La Herradura”, 8 km East of Niebla, E. Maury coll., 23.I.1991, 10 imm. (MACN); Pirehucico, E. Maury coll., 18.I.1985, 5 imm. (MACN). Osorno: Los Derrumbes, E. Maury coll., 09.I.1988, 1 imm. (MACN). Termas Puyehue, S. Roig coll., I.1988, 1 imm. (MACN), Arrededores de Puerto Montt, L. Pereira coll., 28.XI.1992, 1 ♀ (MACN). Osorno: Camping “No me olvides”, 7 km E de Entrelagos, E. Maury coll., 30.I.1991, 7 imm. (MACN). 3 imm. (MACN), Termas de Río Amarillo, SE de Chaitén, E. Maury coll., 01.XII.1986, 3 imm. (MACN). Los Lagos: Sector Anticura, Parque Nacional Puyehue, Sendero Pionero, G. Giribet, G. Hormiga, A. Pérez-González coll., 17.XI.2014, 1 ♂ (MACN). Cautín: Parque Nacional Villarrica, M. Ramírez, F. Labarque coll., 08.II.2005, 1 imm. (MACN). Malleco: Parque Nacional Nahuelbuta, 2 imm. (MACN). Vilches: Las Tacitas, Bordon coll., II.1972, 1 imm. La Cabaña, T. Cekalovic coll., 01.II.1987, 1 imm. (MACN).

###### Diagnosis.

This species, *C.chilensis*, can be readily distinguished from *C.rostrata* by its much shorter ocular process. Furthermore, it possesses a notch on tarsus I and six tarsomeres on leg II (males), whereas *C.rostrata* exhibits seven tarsomeres on the same leg.

###### Distribution.

Argentina: Provinces of Neuquén and Río Negro. Chile, Metropolitan Region of Santiago, O’Higgins, Maule, Ñuble, Bío-Bío, Araucanía, Los Ríos, and Los Lagos (Fig. 4C).

###### Redescription male.

Measurements: Total length 3.40, carapace length 1.44, dorsal scutum length 2.66, carapace max. width 1.63, mesotergum max. width 2.31. Appendage measurements: Pedipalps. Trochanter length 0.25, femora length 1.32, patella length 0.79, tibia length 1.16, tarsus length 0.91. Leg I: trochanter (tr) 0.25, femora (fe) 1.22, patella (pa) 0.61, tibia (ti) 0.80, metatarsus (mt) 1.08, tarsus (ta) 0.82. II: tr 0.30, fe 1.71, pa 0.69, ti 1.23, mt 1.25, ta 1.37. III: tr 0.36, fe 1.05, pa 0.53, ti 0.83, mt 0.86, ta 0.97. IV: tr 0.41, fe 1.55, pa 0.81, ti 1.24, mt 1.44, ta 1.16.

Dorsum (Fig. 44, 45). Eta (η) hourglass-shaped dorsal scutum. Ocularium elevated and rounded, with a small frontal small apophysis perpendicular to the body. Dorsal scutum microgranulate, without distinct delimitation of areas. Areas I–IV with an arcuate row of small setiferous tubercles; posterior margin with two rows of small setiferous tubercles. All free tergites ornamented with small setae and with two rows of small setiferous tubercles.

Chelicerae (Fig. 46A, B). Segment I with an acute tubercle in the dorso-distal portion, providing a distinctive feature. Segment II with a small frontal tubercle and adorned with only a few setae.

Pedipalps (Fig. 46C, D). Trochanter with a dorsal tubercle adorned with setae and a small ventral spine. Femora with dorsal row of four small spines with subdistal setae, in ventral view with two long proximal spines with subdistal setae and a row of small tubercles. In mesal view, with six low setiferous tubercles. Patella with a smooth surface; tibia with two ectal and mesal tubercles with subdistal setae, a row of four small ventroproximal setiferous tubercles, and a row of three small ectal setiferous tubercles. Tarsus with three mesal and ectal tubercles with subdistal setae, with a few setae.

Legs (Fig. 47). Coxa I with two rows of setiferous tubercles, along two distal setiferous tubercles with subdistal setae. Coxa II with two rows of setiferous tubercles, while coxae III and IV feature only microgranulation. Three bridges between legs II and III, six between III and IV, and 4–8 between leg IV and the opisthosoma, with the distal bridge longer than the others. Spiracles unobstructed by bridges. A smooth surface covers ~ 1/3 of leg II, ¾ of leg III, and < 1/3 of leg IV. Within the smooth area of leg II, there is a small tubercle on each side, accompanied by subdistal setae. Sternum arrow-shaped, with serrated margins, and the posterior area forms a triangle. Segments I–IV covered in setae, tarsal area, and calcaneus densely setose. Tibiae I–III with a ventral and dorsal row of small setiferous tubercles, whereas tibia IV has a row of four distal-ventral tubercles with setae. Calcaneus smaller than astragalus, ≥ 3× smaller in leg I, 4× smaller in legs II–III, and 5× smaller in leg IV. Tarsal count: 4–6–4–4.

Penis (Figs 48, 49). Pars distalis with a large ventral cleft that divides the plate into two lamellae. Each lamella is equipped with three pointed macrosetae on the ventral surface and one macroseta on the dorsal surface. Capsula externa covers dorsal and lateral surfaces and exhibits a V-shaped medial notch, which divides the dorsal fold into two halves. A dorsolateral plate appears attached to the pars basalis. Capsula interna slightly longer than capsula externa and partially covers ventral plate. Capsula interna formed by two parts fused in the apical region and with a visible stylus in its medial portion.

**Female.** Similar to male, with shorter pedipalpal femora, presence of interocular process and a different tarsal count: 3–6–4–4.

Female measurements: Total length 2.87, carapace length 1.19, dorsal scutum length 2.38, carapace max. width 1.47, mesotergum max. width 2.14. Appendage measurements: Pedipalps. Trochanter length 0.28, femora length 0.96, patella length 0.55, tibia length 0.82, tarsus length 0.75. Leg I: trochanter (tr) 0.20, femora (fe) 1.08, patella (pa) 0.55, tibia (ti) 0.76, metatarsus (mt) 0.89, tarsus (ta) 0.74. II: tr 0.29, fe 1.32, pa 0.66, ti 1.08, mt 1.17, ta 1.30. III: tr 0.26, fe 1.01, pa 0.48, ti 0.69, mt 0.88, ta 0.78. IV: tr 0.32, fe 1.26, pa 0.71, ti 1.17, mt 1.28, ta 1.09.

##### ﻿ Chilenuncia rostrata

Taxon classificationAnimaliaOpilionesTriaenonychidae

(Maury, 1990) comb. nov.

053E6B73-5850-55FC-9197-B03F9BDD990D

**Figure 50.**
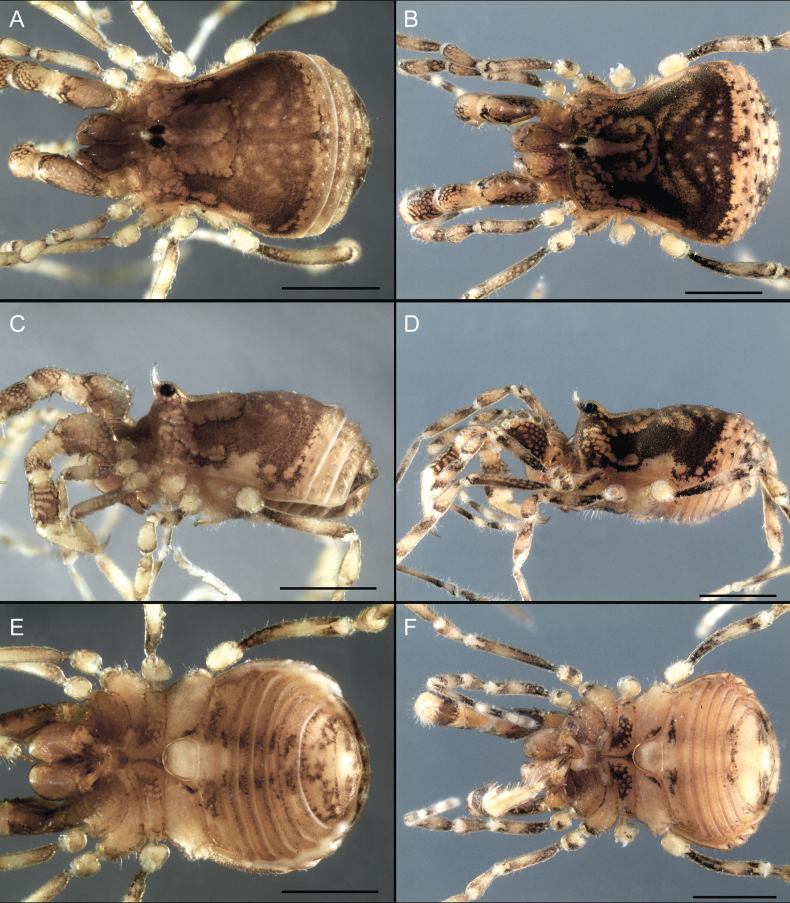
*Chilenunciarostrata* comb. nov. habitus, male **A** dorsal view **C** lateral view **E** ventral view. Female **B** dorsal view **D** lateral view **F** ventral view. Scale bars: 1 mm.

**Figure 51.**
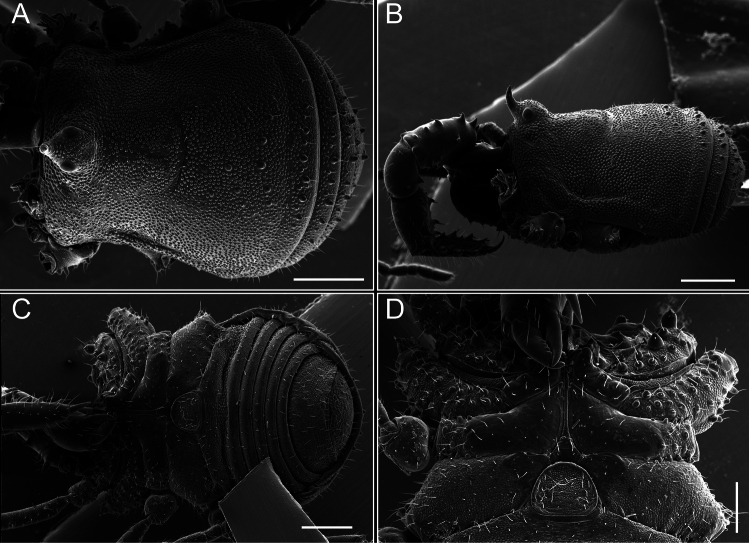
*Chilenunciarostrata* comb. nov. male, SEM images of habitus **A** dorsal view **B** lateral view **C, D** ventral view. Scale bars: 500 µm (**A, B, C**); 200 µm (**D**).

**Figure 52.**
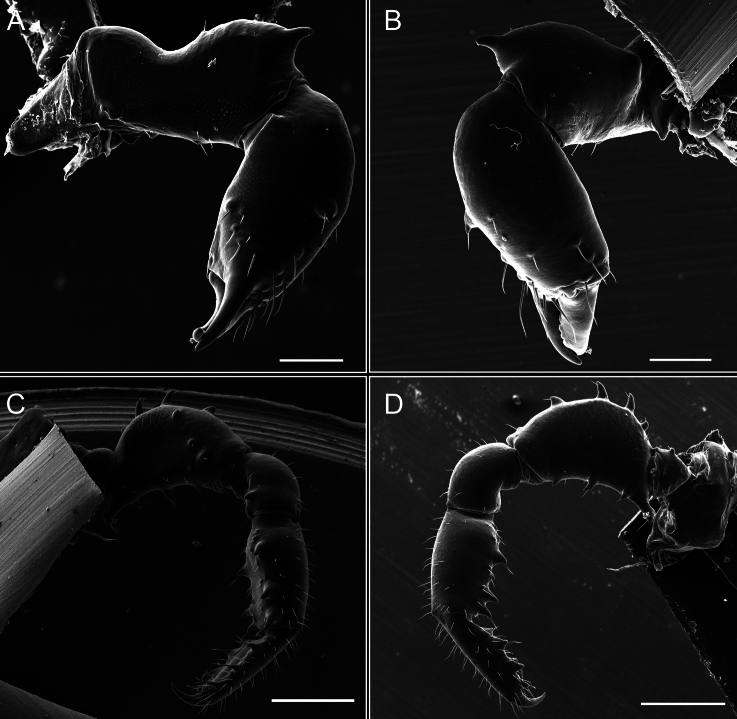
*Chilenunciarostrata* comb. nov. chelicerae: mesal **A** ectal **B** Pedipalps: mesal **C** ectal **D**. Scale bars: 200 µm (**A, B**); 500 µm (**C, D**).

**Figure 53.**
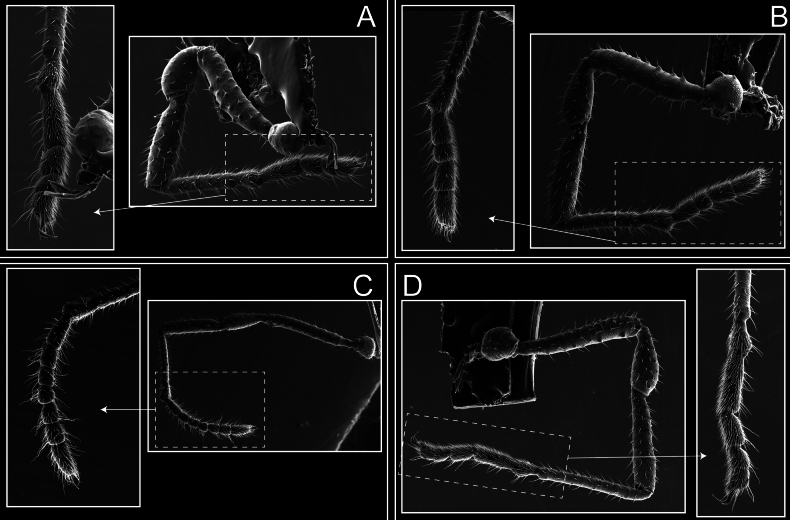
*Chilenunciarostrata* comb. nov. legs I **A** II **B** III **C** IV **D**. Scale bars: 500 µm (**A, B, D**); 200 µm (**C**).

**Figure 54.**
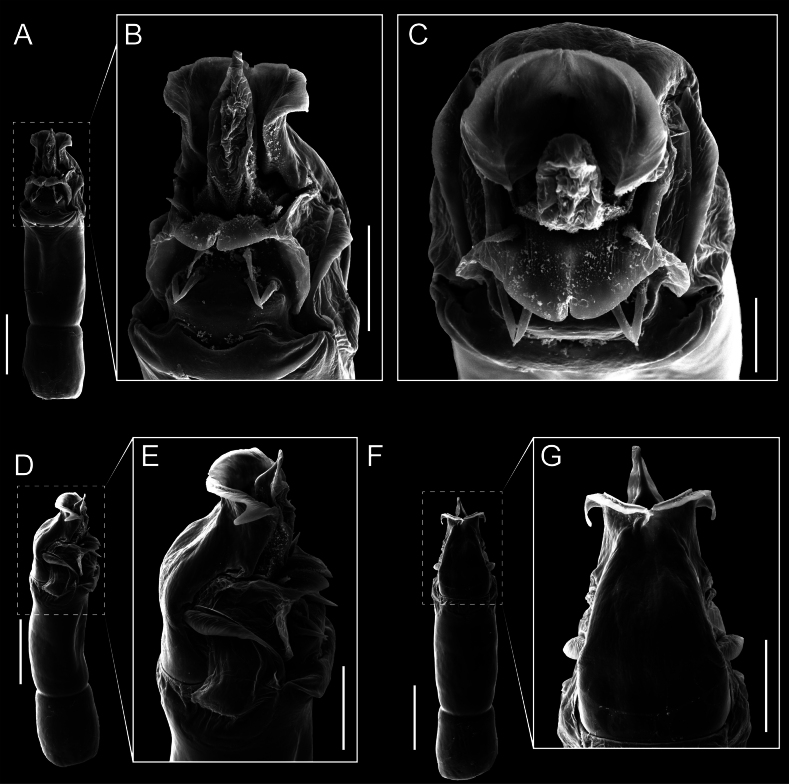
*Chilenunciarostrata* comb. nov. penis: ventral **A, B** apical **C** lateral **D, E** dorsal **F, G**. Scale bars: 200 µm (**A, D, F**); 100 µm (**B, E, G**); 50 µm (**C**).

**Figure 55.**
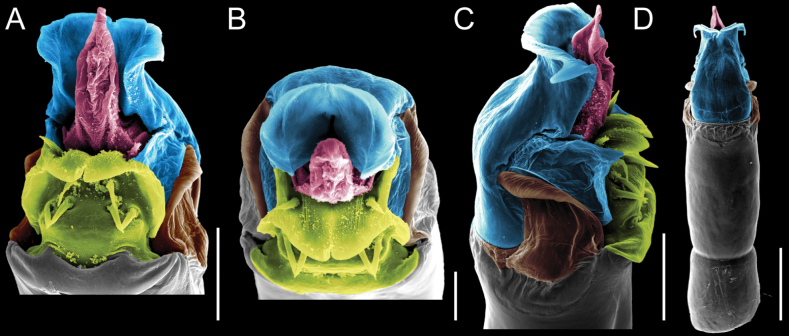
*Chilenunciarostrata* comb. nov. penis: ventral **A** apical **B** lateral **C** dorsal **D**. Colors: ventral plate (yellow), capsula externa (blue), capsula interna (red). Scale bars: 100 µm (**A, C, D**); 50 µm (**B**).

Figs 50 , 51 , 52 , 53 , 54 , 55

Nuncia rostrata Maury, 1990: 110, figs 25–35; Maury 1992: 4; Kury 2003: 22; Pérez-Schultheiss et al. 2019: 20; Pérez-Schultheiss et al. 2021: 412, fig. 3a, d.

###### Material examined.

***Holotype*.** ♂ **Chile.** Llanquihue, Caleta La Arena, 50 km S de Puerto Montt, Carretera Austral, 07–08.XII.1985 (MACN 8703). ***Paratype* (allotype** ♀) **. Chile.** Llanquihue, Caleta La Arena, 50 km S de Puerto Montt, Carretera Austral, 07–08.XII.1985 (MACN 8704). ***Paratypes*.** Chile. Llanquihue, Caleta La Arena, 50 km S de Puerto Montt, Carretera Austral, 07–08.XII.1985 (MACN 8705).

###### Additional material.

Chile. Llanquihue: 35 km W. Río Negro, R.Schuh, N. Platnick coll., 24.I.1986, 1 ♀ (AMNH). Chiloé: Chiloé Island, 5 km N of Quellón, R.Schuh, N. Platnick coll., 01.XII.1981, 1 ♀ 1 imm. (AMNH), Isla Chiloé, R.Schuh, N. Platnick coll., 29.XI.1981, 1 ♂ 1 ♀ (AMNH). Llanquihue: P.N. Alerce Andino, N. Platnick, K.Catley, M. Ramírez, T.Allen coll., 23.XI.1993, 1 ♀ 2 imm. (AMNH). Palena: Termas de Pichicolo, 11 km west of Río Negro, Carretera Austral, E. Maury coll., 08.XII.1985, 1 ♀ (MACN 8706). Aysén: 30 km NE de Puerto Cisnes, E. Maury coll., 08.XII.1986, 2 ♀ 3 imm. (MACN 8707).

###### Diagnosis.

This species can be readily distinguished from *C.chilensis* by the prominent interocular apophysis, which is notably longer in *C.rostrata*. Additionally, *C.rostrata* possesses seven tarsomeres on leg II (males), whereas *C.chilensis* has six. These characteristics serve as key distinguishing features between the two species.

###### Distribution.

Chile: Los Lagos and Aysén Regions (Fig. 4C).

###### Redescription of male

**(MACN 8705).** Measurements: Total length 2.97. Carapace length 1.07, dorsal scutum length 2.09, carapace max. width 1.49, mesotergum max. width 1.94. Appendage measurements: Pedipalps. Trochanter length 0.25, femora length 0.95, patella length 0.55, tibia length 0.68, tarsus length 0.66. Leg I: trochanter (tr) 0.25, femora (fe) 0.87, patella (pa) 0.50, tibia (ti) 0.70, metatarsus (mt) 0.89, tarsus (ta) 0.85. II: tr 0.33, fe 1.28, pa 0.54, ti 0.96, mt 1.08, ta 1.45. III: tr 0.32, fe 0.81, pa 0.43, ti 0.66, mt 0.77, ta 0.85. IV: tr 0.34, fe 1.15, pa 0.61, ti 0.95, mt 1.31, ta 1.03.

Dorsum (Fig. 50, 51). Eta (η) hourglass-shaped dorsal scutum. Ocularium elevated, with a long, backward-curving apophysis. Eyes located laterally in the middle of the ocular structure. Dorsal scutum microgranulate and without clear delimitation of areas. Area I with a pair of setae, while areas II–IV display an arcuate row of small setae tubercles. Posterior margin characterized by a row of small setiferous tubercles. All free tergites with a row of small setiferous tubercles.

Chelicerae (Fig. 52A, B). Segment I with a small tubercle on its dorso-distal surface; segment II with a mesal tubercle and bears a few setae.

Pedipalps (Fig. 52C, D). Trochanter with a small dorsal and ectal tubercle. Femora with two ventroproximal and one ventromedial spine with subdistal setae, with two distal setiferous granules and three dorsoproximal tubercles with subdistal setae. Mesal surface of femora with 2–3 distal setiferous granules. Patella with a ventral setiferous tubercle. Tibia with four ectal and three mesal spines with subdistal setae, while the ventral surface presents small setiferous granules. Tarsus with three mesal and four ectal spines with subdistal setae, as well as a few setae and granules.

Legs (Fig. 53). Coxa I with a row of setiferous tubercles, with two distal setiferous tubercles with subdistal setae. Coxae II–III also with setiferous tubercles, while Coxa IV with only microgranulation. Four bridges between legs II and III, and five or six bridges between legs III and IV. Spiracles not obstructed by bridges. A smooth surface covers ~ 1/3 of leg II, ¾ of leg III, and < 1/3 of leg IV. Within the smooth area of leg II, two small tubercles with subdistal setae can be observed on each side. Sternum arrow-shaped, with a triangular posterior area. Smooth, covered in setae, without a notch on tarsus I. Tarsal count 4–7–4–4.

Penis (Figs 54, 55). Pars distalis with large ventral cleft that divides the plate into two lamellae. Each lamella with three pointed macrosetae on the ventral surface and one macroseta on the dorsal surface. Capsula externa covers dorsal and lateral surfaces, exhibiting a small medial notch that divides the apical region of the capsula externa. With a dorsolateral plate attached to the pars basalis. Capsula interna slightly longer than capsula externa and partially covers the ventral plate. Capsula interna sac-shaped in appearance.

**Female.** Similar to male, with shorter pedipalpal femora and interocular apophysis.

Female measurements. Total length 3.17. Carapace length 0.98, dorsal scutum length 2.12, carapace max. width 1.38, mesotergum max. width 2.08. Appendage measurements: Pedipalps. Trochanter length 0.21, femora length 0.83, patella length 0.45, tibia length 0.59, tarsus length 0.62. Leg I: trochanter (tr) 0.23, femora (fe) 0.84, patella (pa) 0.52, tibia (ti) 0.69, metatarsus (mt) 0.82, tarsus (ta) 0.64. II: tr 0.30, fe 1.13, pa 0.52, ti 0.89, mt 0.92, ta 1.23. III: tr 0.30, fe 0.73, pa 0.40, ti 0.72, mt 0.79, ta 0.73. IV: tr 0.30, fe 1.13, pa 0.60, ti 0.90, mt 1.27, ta 0.93. Tarsal count: 3–6–4–4.

##### ﻿Genus Laftrachia gen. nov.

Taxon classificationAnimaliaOpilionesTriaenonychidae

166B4133-778F-59B6-93CA-84E45B606F49

https://zoobank.org/4BE5CC16-9A40-4F4C-A0EB-7632F000E1F5

**Figure 56.**
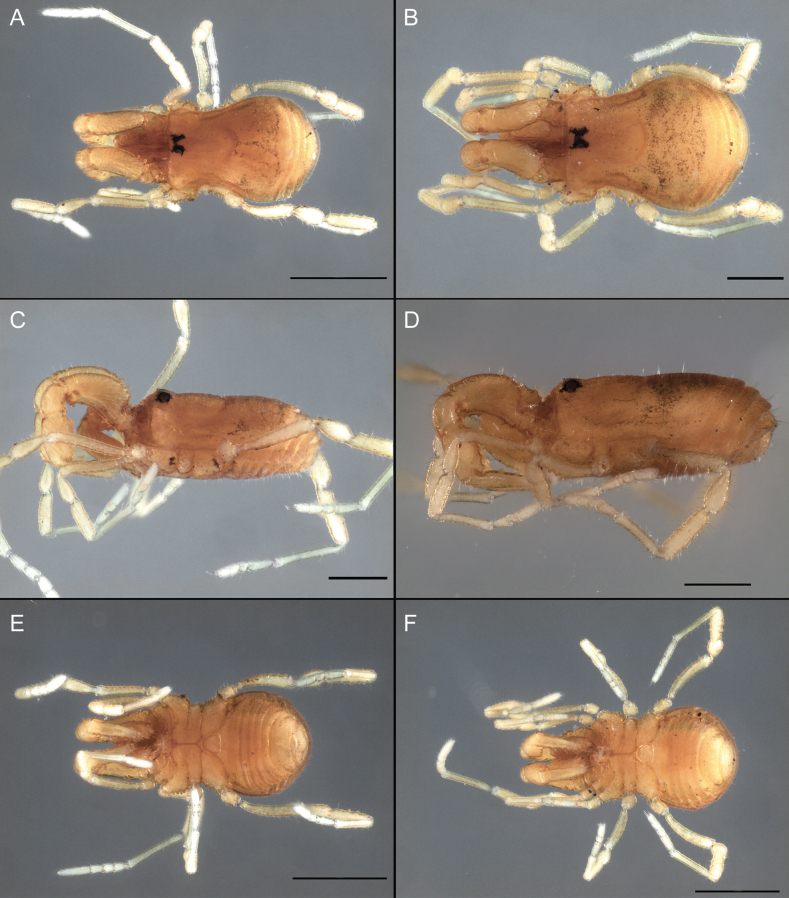
*Laftrachiarobin* sp. nov. habitus, male **A** dorsal view **C** lateral view **E** ventral view. Female **B** dorsal view **D** lateral view **F** ventral view. Scale bars: 500 µm. Species of Clade C, see Fig. 3.

**Figure 57.**
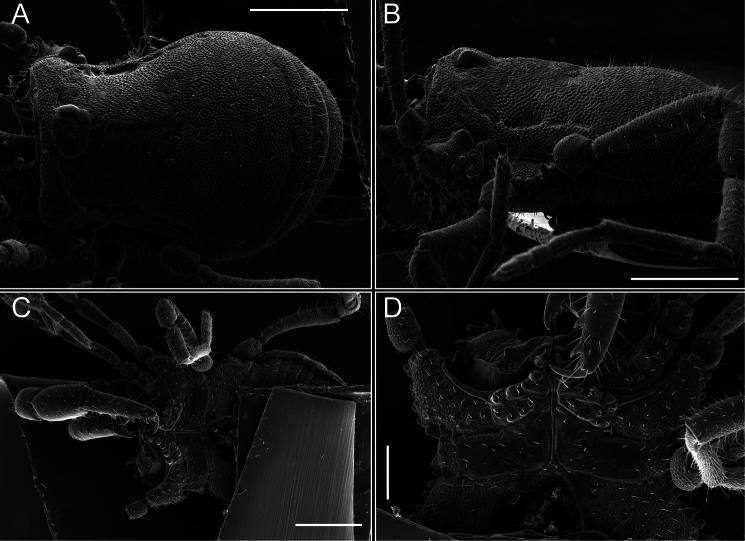
*Laftrachiarobin* sp. nov. male, SEM images of habitus **A** dorsal view **B** lateral view **C, D** ventral view. Scale bars: 500 µm.

**Figure 58.**
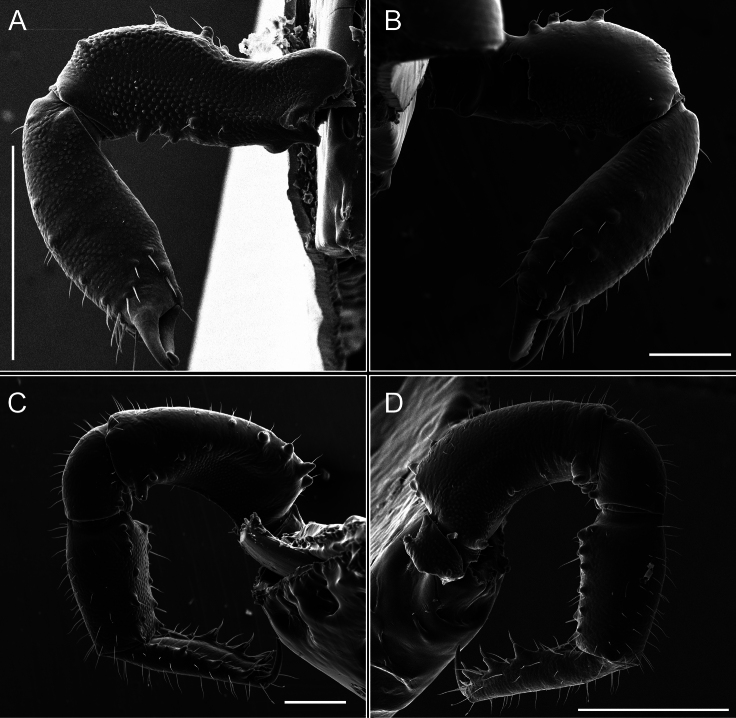
*Laftrachiarobin* sp. nov. chelicerae: mesal **A** ectal **B** Pedipalps: mesal **C** ectal **D**. Scale bars: 500 µm.

**Figure 59.**
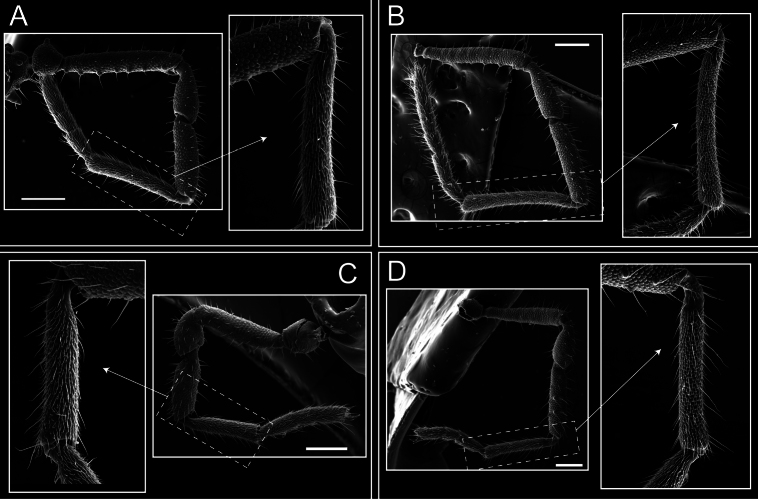
*Laftrachiarobin* sp. nov. legs I **A** II **B** III **C** IV **D**. Scale bars: 200 µm.

**Figure 60.**
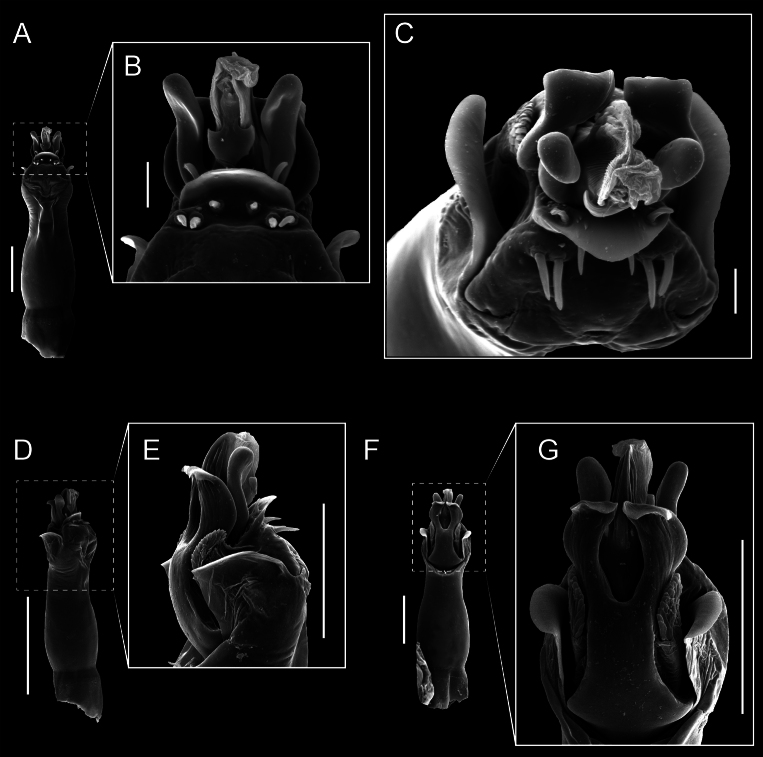
*Laftrachiarobin* sp. nov. penis: ventral **A, B** apical **C** lateral **D, E** dorsal **F, G**. Scale bars: 100 µm (**A, D, E, F**); 20 µm (**B, C**); 50 µm (**G**).

**Figure 61.**
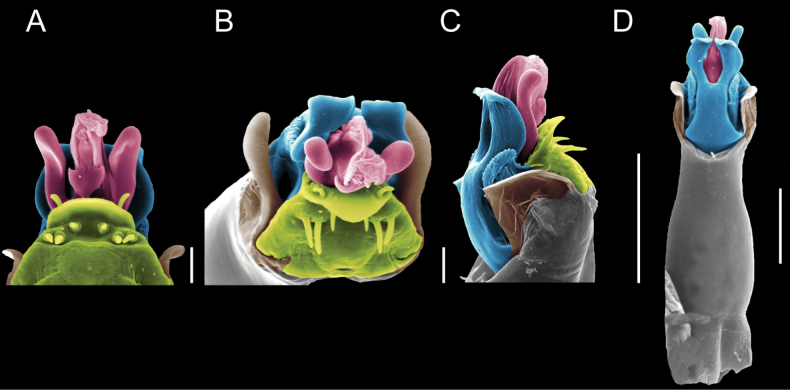
*Laftrachiarobin* sp. nov. penis: ventral **A** apical **B** lateral **C** dorsal **D**. Colors: ventral plate (yellow), capsula externa (blue), capsula interna (red). Scale bars: 20 µm (**A, B**); 100 µm (**C, D**).

Figs 56 , 57 , 58 , 59 , 60 , 61

###### Etymology.

The specific epithet derives from Laftrache (which in Mapudungun, Mapuche Language, means “little people”) also known as Caftranche, a mythical being present in Mapuche mythology. Feminine grammatical gender.

###### Diagnosis.

This monotypic genus is characterized by its small size, yellow-orange coloration, a prominent ectal-distal process on the pedipalp femora, a low ocularium, and distinct male genital morphology. The male genitalia exhibits an arc-shaped ventral plate and a pair of parallel projections within the capsula interna. These distinguishing features differentiate it from other genera within the family Triaenonychidae.

###### Type species.

*Laftrachiarobin* sp. nov.

###### Included species.

*Laftrachiarobin* sp. nov.

###### Distribution.

Chile: Bío-Bío Region (Fig. 4D).

##### ﻿ Laftrachia robin sp. nov.

Taxon classificationAnimaliaOpilionesTriaenonychidae

92AB55C3-DB38-5504-948B-E79606D4AB65

https://zoobank.org/2BBB0B84-397C-4384-B5A9-3B8A73478123

Figs 56 , 57 , 58 , 59 , 60 , 61

###### Material examined.

***Holotype*.** ♂ **Chile.** Arauco: San Alfonso, Quebrada Caramávida, Arauco Reserve, 37.70942°S, 73.17107°W, 750 m, M. Ramírez, A. Ojanguren, A. Pérez-González, G. Azevedo, W. Porto coll., 15.I.2018 (MNHNCL). ***Paratypes*. Chile.** Arauco: San Alfonso, Quebrada Caramávida, Arauco Reserve, 37.70942°S, 73.17107°W, 750 m, M. Ramírez, A. Ojanguren, A. Pérez-González, G. Azevedo, W. Porto coll., 15.I.2018, 1 ♂ 6 ♀ (MACN).

###### Etymology.

The species name, a noun in apposition, is a reference to the DC comics character “Robin” (https://www.dccomics.com/characters/robin). The black pigmentation in the eye region of the species is similar to the mask used by the character in his appearances.

###### Description of male.

Measurements: Total length 1.65, carapace length 0.66, dorsal scutum length 1.28, max. carapace width 0.87, max. dorsal scutum width 1.14. Appendage measurements: Pedipalps. Trochanter length 0.18, femora length 0.88, patella length 0.30, tibia length 0.51, tarsus length 0.50. Leg I: trochanter (tr) 0.16, femora (fe) 0.56, patella (pa) 0.29, tibia (ti) 0.42, metatarsus (mt) 0.37, tarsus (ta) 0.46. II: tr 0.17, fe 0.75, pa 0.32, ti 0.53, mt 0.82, ta 0.57. III: tr 0.18, fe 0.51, pa 0.21, ti 0.27, mt 0.44, ta 0.50. IV: tr 0.22, fe 0.69, pa 0.39, ti 0.57, mt 0.67, ta 0.73.

Dorsum (Fig. 56, 57). Eta (η) hourglass-shaped dorsal scutum. Ocularium low, lacking a medial spine. Carapace covering ~ ½ of the dorsal scutum, displaying furrows on the posterior area and distinct lateral edges. Free tergites with a smooth surface.

Chelicerae (Fig. 58A, B). Segment I characterized by the presence of five ventral tubercles and three dorsal tubercles, all adorned with subdistal setae. Segment II with a total of 11 ectal tubercles, five mesal tubercles, and approximately seven frontal tubercles.

Pedipalps (Fig. 58C, D). Trochanter with a dorsal tubercle. Femora with a row of four ventral tubercles, a row of dorsal tubercles adorned with subdistal setae, and a long distal ectal apophysis. Patella with a pair of small ventral tubercles with setae. Tibia with two ventral rows of tubercles, with three small tubercles and two setiferous tubercles on the mesal side, and a row of six small setiferous tubercles on the ectal side. Tarsus equipped with three tubercles featuring ectal and mesal subdistal setae.

Legs (Fig. 59). Coxa I with four small proximal tubercles, one medial tubercle with subdistal setae, and three distal tubercles (two small and one with subdistal seta). Coxa II with scattered small tubercles with setae. Coxae III and IV smooth, while the cerotegument covers the distal portion of leg III and almost the entire leg IV. Bridge is present. Spiracles visible. Sternum arrow-shaped. Trochanter of leg I and femora of legs I and II covered in small tubercles with setae. Tibia of leg IV covered with small tubercles with setae. Metatarsus-tarsus of all legs densely setose. Tarsus occupies almost the entire length of metatarsus. Tarsal count: 3–4–3–4.

Penis (Figs 60, 61). Pars distalis with an arcuate ventral plate without a cleft, characterized by three ventral and one dorsal macrosetae each side. Capsula externa shorter than capsula interna and with a dorsal cleft that divides it into two dorsal halves. Additionally, there are lateral folds present on each side, and a dorsolateral plate surrounds the capsula externa. Capsula interna trifid, with two lateral processes running parallel to stylus. Stylus membranous and shaped like an inflatable bag.

**Female.** Similar to male, but with certain differences: pedipalps are shorter, lacks ectal-distal apophysis present on pedipalp femora of male.

Female measurements. Total length 1.75, carapace length 0.71, dorsal scutum length 1.46, carapace max. width 0.84, mesotergum max. width 1.23. Appendage measurements: Pedipalps. Trochanter length 0.17, femora length 0.63, patella length 0.34, tibia length 0.46, tarsus length 0.45. Leg I: trochanter (tr) 0.12, femora (fe) 0.57, patella (pa) 0.31, tibia (ti) 0.38, metatarsus (mt) 0.48, tarsus (ta) 0.44. II: tr 0.17, fe 0.80, pa 0.35, ti 0.51, mt 0.77, ta 0.80. III: tr 0.18, fe 0.47, pa 0.25, ti 0.32, mt 0.44, ta 0.50. IV: tr 0.22, fe 0.72, pa 0.38, ti 0.53, mt 0.66, ta 0.60.

##### ﻿Genus Lautaria gen. nov.

Taxon classificationAnimaliaOpilionesTriaenonychidae

2CA5E4F6-978B-5D74-A276-38E0B8F72064

https://zoobank.org/DBD404FC-68B8-4EE6-8BB9-8E58588C2867

**Figure 62.**
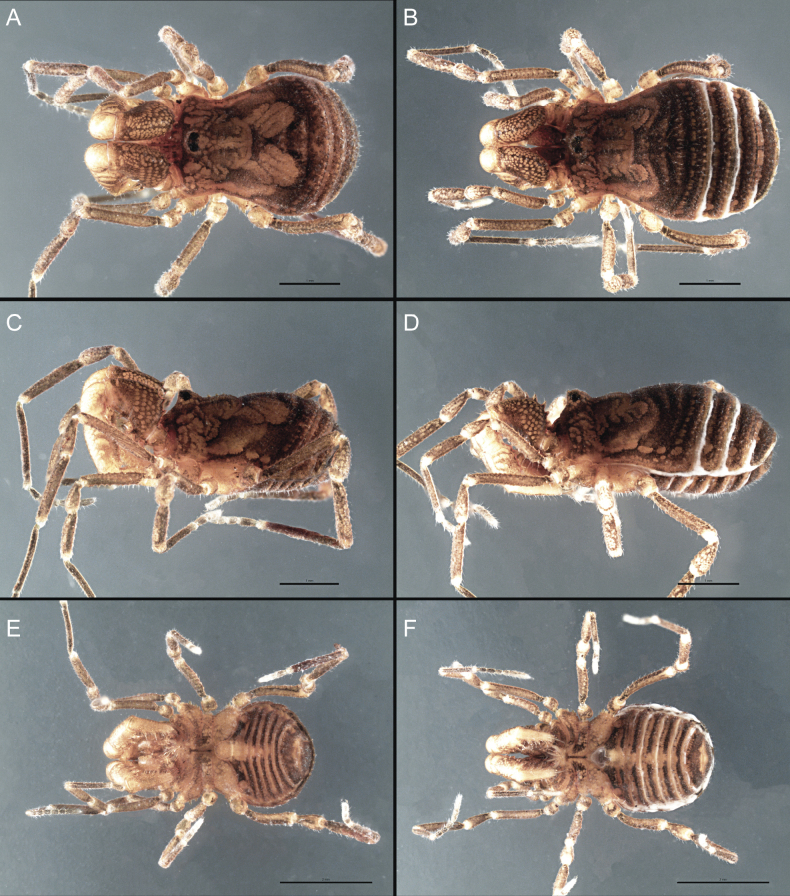
*Lautariaceachei* sp. nov. habitus, male **A** dorsal view **C** lateral view **E** ventral view. Female **B** dorsal view **D** lateral view **F** ventral view. Scale bars: 500 µm. Species of Clade C, see Fig. 3.

**Figure 63.**
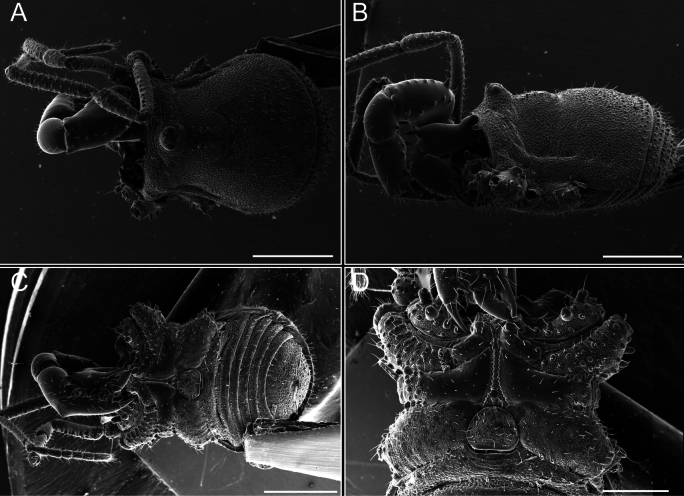
*Lautariaceachei* sp. nov. male, SEM images of habitus **A** dorsal view **B** lateral view **C, D** ventral view. Scale bars: 1 mm (**A, B, C**); 500 µm (**D**).

**Figure 64.**
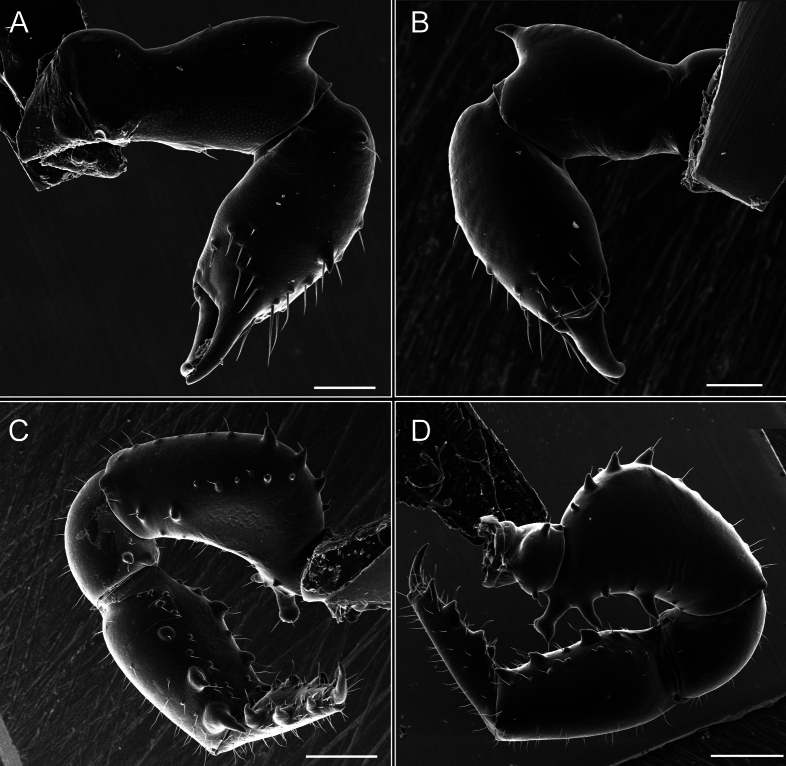
*Lautariaceachei* sp. nov. chelicerae: mesal **A** ectal **B** Pedipalps: mesal **C** ectal **D**. Scale bars: 200 µm (**A, B**); 500 µm (**C, D**).

**Figure 65.**
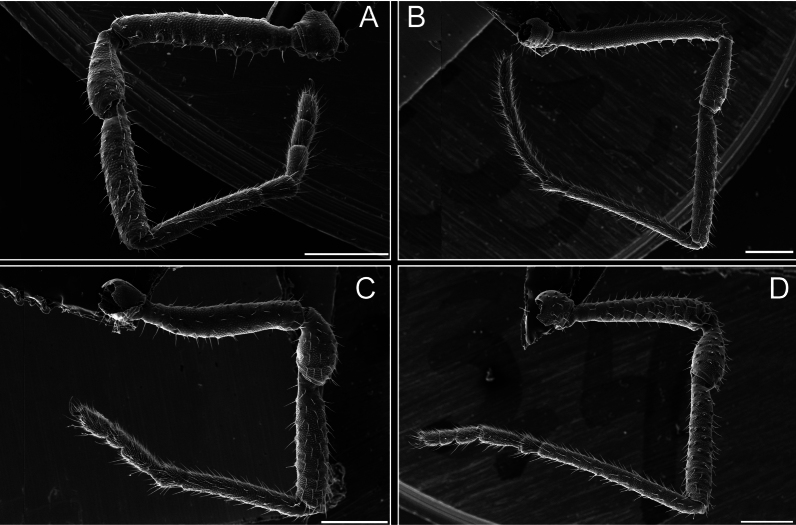
*Lautariaceachei* sp. nov. legs I **A** II **B** III **C** IV **D**. Scale bars: 500 µm.

**Figure 66.**
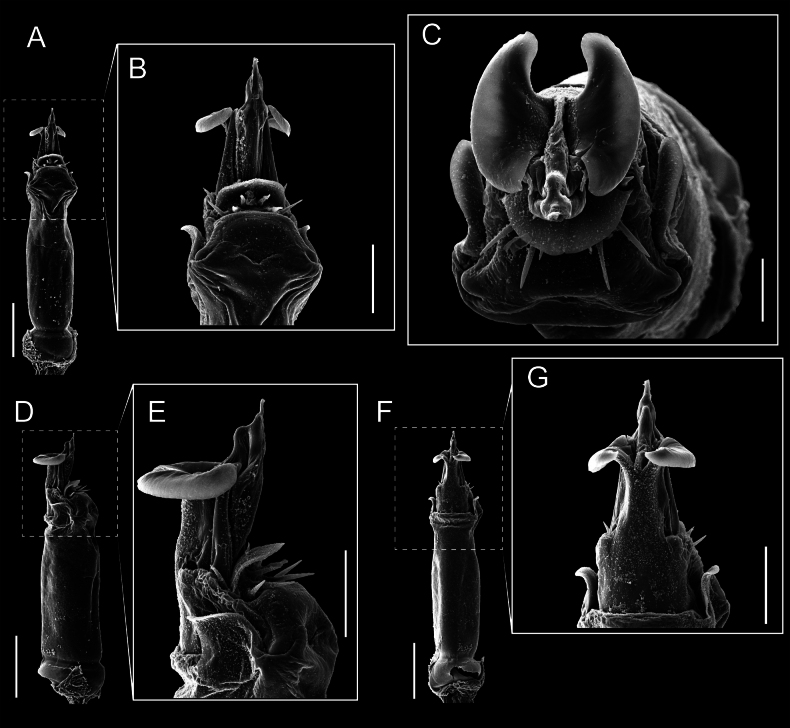
*Lautariaceachei* sp. nov. penis: ventral **A, B** apical **C** lateral **D, E** dorsal **F, G**. Scale bars: 200 µm (**A, D, F**); 100 µm (**B, E, G**); 50 µm (**C**).

**Figure 67.**
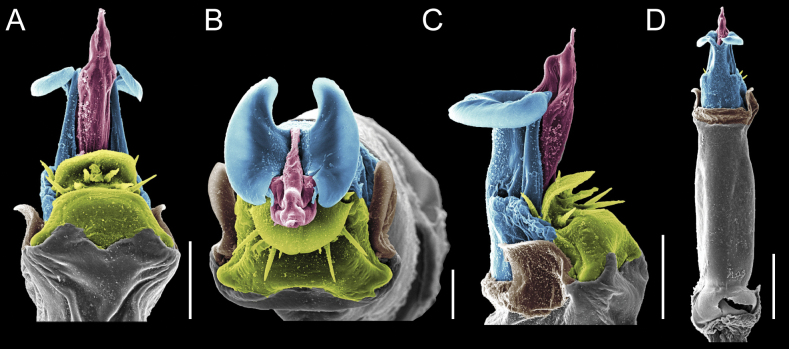
*Lautariaceachei* sp. nov. penis: ventral **A** apical **B** lateral **C** dorsal **D**. Colors: ventral plate (yellow), capsula externa (blue), capsula interna (red). Scale bars: 1 mm.

Figs 62 , 63 , 64 , 65 , 66 , 67

###### Etymology.

The genus name honors Lautaro (Leftraru), a renowned toqui (Mapuche military leader) who played a significant role in the Arauco War during the early stages of the Spanish conquest of what is now Chile. The name is derived from Mapudungun, with “lef” meaning “fast” and “traru” or “bald traro.” The feminine grammatical gender is used for the generic epithet.

###### Diagnosis.

*Lautaria* can be distinguished from all other genera of Triaenonychidae by the unique male genital morphology (Fig. 66, 67). This includes a capsula externa with an apical region divided into two halves, forming a 90° angle in relation to the axis of the pars basalis. The external morphology exhibits striking similarities to the genus *Chilenuncia*, and other members of clade C.

###### Type species.

*Lautariaceachei* sp. nov.

###### Included species.

*Lautariaceachei* sp. nov.

###### Distribution.

Chile: Ñuble Region (Fig. 4E).

##### ﻿ Lautaria ceachei sp. nov.

Taxon classificationAnimaliaOpilionesTriaenonychidae

22326C2D-3A43-5539-A90B-A62AF2AE2269

https://zoobank.org/B1D033C3-2192-4672-B2B9-517D1CA30851

Figs 62 , 63 , 64 , 65 , 66 , 67

###### Material examined.

***Holotype*.** ♂ **Chile.** Altos del Lircay RN, E Vilches Alto, 35.5987°S, 71.04097°W, 1380 m, M. Ramírez, A. Ojanguren, A. Pérez-González, G. Azevedo, W. Porto coll. (MNHNCL). ***Paratypes*. Chile.** Altos del Lircay RN, E Vilches Alto, 35.5987°S, 71.04097°W, 1380 m, M. Ramírez, A. Ojanguren, A. Pérez-González, G. Azevedo, W. Porto coll. 15.I.2018, 1 ♂ 4 ♀, 2 imm. (MACN).

###### Etymology.

The specific epithet “ceachei” is derived from the chanted cry “ceacheí,” commonly used during sporting events to cheer on Chilean representatives. This Chileanism is created by combining the initial letters of the word “Chile.” The epithet serves as noun in apposition, highlighting the connection to Chilean representation and support.

###### Description of male.

Measurements: Total length 2.49. Carapace length 1.14, dorsal scutum length 2.20, carapace max. width 1.63, mesotergum max. width 2.45. Appendage measurements: Pedipalps. Trochanter length 0.30, femora length 0.90, patella length 0.60, tibia length 0.71, tarsus length 0.70. Leg I: trochanter (tr) 0.27, femora (fe) 0.95, patella (pa) 0.54, tibia (ti) 0.83, metatarsus (mt) 1.08, tarsus (ta) 0.61 II: tr 0.32, fe 1.42, pa 0.61, ti 1.10, mt 1.36, ta 0.61. III: tr 0.38, fe 0.95, pa 0.54, ti 0.81, mt 1.02, ta 0.73. IV: tr 0.39, fe 1.41, pa 0.73, ti 1.16, mt 1.49, ta 0.87.

Dorsum (Fig. 62, 63). Eta (η) hourglass-shaped dorsal scutum, characterized by its distinctive form. Ocularium low and rounded, with an acute small apophysis. Both the dorsal scutum and free tergites covered with microgranulation, providing a textured surface. Mesotergal areas without clear separation but with small setiferous tubercles, with a stronger presence in the mesotergum and free tergites.

Chelicerae (Fig. 64A, B). Segment I of with a sharp spine on the dorso-distal surface, with three small ventral-proximal tubercles. Segment II with scattered setae in both ectal and ventral views, and in front view, with a triangular tubercle that stands out from the others.

Pedipalps (Fig. 64C, D). Trochanter with a small dorsal and a ventral tubercle. Femora with two parallel rows of dorso-mesal tubercles, with three ventral spines and a few setiferous tubercles. Patella with a mesal tubercle. Tibia with three ventral-ectal spines, two ventral-mesal spines, and small scattered ventral tubercles.

Legs (Fig. 65). Coxa I characterized by having 9–10 setiferous tubercles, the two apical ones being stronger and more prominent than the others. Coxa II with a higher number of setiferous tubercles, ranging from 20 to 25. Coxa III with 12–14 tubercles, while coxa IV has five or six small tubercles. Spiracles not obstructed. A smooth surface covers ~1/3 of leg II, ¾ of leg III, and < 1/3 of leg IV. Sternum arrow-shaped. Legs covered in small tubercles. Astragalus longer than the calcaneus on all legs. Tarsal count: 3–6–4–4.

Penis (Figs 66, 67). Pars distalis with a ventral plate and a small cleft, dividing it into two lamellae. Each lamella with three pointed macrosetae on the ventral surface and one macroseta on the dorsal surface. Capsula externa lower than the capsula interna, with a notch in the apical part, dividing into two lateral apical “wings”. With an additional dorsolateral plate. Capsula interna thin and laterally flattened, with a sharp apical area.

**Female.** Similar to male but with shorter pedipalpal femora and a reduced interocular process.

Female measurements: Total length 2.98. Carapace length 1.11, dorsal scutum length 2.39, carapace max. width 1.66, mesotergum max. width 2.46. Appendage measurements: Pedipalps. Trochanter length 0.33, femora length 1.09, patella length 0.53, tibia length 0.81, tarsus length 1.05. Leg I: trochanter (tr) 0.22, femora (fe) 1.09, patella (pa) 0.50, tibia (ti) 0.86, metatarsus (mt) 1.06, tarsus (ta) 0.63 II: tr 0.24, fe 1.42, pa 0.60, ti 1.12, mt 1.35, ta 1.17. III: tr 0.27, fe 1.06, pa 0.47, ti 0.86, mt 1.01, ta 0.62. IV: tr 0.37, fe 1.43, pa 0.72, ti 1.16, mt 1.42, ta 0.85.

##### ﻿Genus Nerudiella gen. nov.

Taxon classificationAnimaliaOpilionesTriaenonychidae

3F5E9325-7804-5E1F-8341-CBED0832F411

https://zoobank.org/DF572280-150A-44D7-9964-2B1E2749F912

Figs 68 , 69 , 70 , 71 , 72 , 73 , 74 , 75 , 76 , 77 , 78 , 79 , 80 , 81 , 82 , 83 , 84 , 85 , 86 , 87 , 88 , 89 , 90 , 91 , 92 , 93 , 94 , 95 , 96 , 97 , 98 , 99 , 100 , 101 , 102 , 103 , 104 , 105 , 106 , 107 , 108 , 109 , 110 , 111 , 112 , 113 , 114 , 115 , 116 , 117 , 118 , 119 , 120 , 121 , 122 , 123 , 124 , 125 , 126 , 127 , 128 , 129 , 130 , 131 , 132 , 133 , 134 , 135 , 136 , 137 , 138 , 139 , 140 , 141 , 142 , 143 , 144 , 145 , 146 , 147 , 148 , 149 , 150 , 151 , 152 , 153 , 154 , 155 , 156 , 157 , 158 , 159

Nuncia [part] (references to Nunciaamericana): Roewer 1961: 102, pl. 19, figs 13–15; Cekalovic 1968: 6; Besch 1969: 733; Muñoz-Cuevas 1971a: 873, fig. 28; Kury 2003: 21.
Nuncia (Nuncia) americana : Muñoz-Cuevas 1971b: 98, figs 1–17; Cekalovic 1985:11.

###### Etymology.

The generic epithet *Nerudiella* honors renowned Chilean poet and politician Pablo Neruda (1904–1973). Feminine grammatical gender.

###### Diagnosis.

*Nerudiella* species can be easily distinguished from other genera of Triaenonychidae by several notable characteristics. These include a relatively low to medium ocularium, which lacks an interocular apophysis or possesses a reduced apophysis (similar to that of the New Zealand *Nuncia*); a pedipalpal femora typically slightly curved; and presence of a subtle sexual dimorphism. The most salient feature is the male genitalia with a distinctive dorsolateral plate that originates from the genitalia’s pars basalis and extends onto the pars distalis. This plate appears wide in its lateral portion but small in dorsal view. These unique genital structures serve as reliable markers for the identification of *Nerudiella* species.

###### Type species.

*Nunciaamericana* Roewer, 1961

###### Included species.

*Nerudiellaamericana* (Roewer, 1961) comb. nov., *Nerudiellacachai* sp. nov., *Nerudiellacaramavida* sp. nov., *Nerudiellacautin* sp. nov., *Nerudiellachoapa* sp. nov., *Nerudiellacuri* sp. nov., *Nerudiellagoroi* sp. nov., *Nerudiellajaimei* sp. nov., *Nerudiellamalleco* sp. nov., *Nerudiellapenco* sp. nov., *Nerudiellapichi* sp. nov., *Nerudiellaportai* sp. nov., *Nerudiellaquenes* sp. nov., *Nerudiellavilches* sp. nov., *Nerudiellawekufe* sp. nov., *Nerudiellazapallar* sp. nov.

###### Distribution.

Chile: From Coquimbo to Los Lagos Region (Fig. 4F).

##### ﻿ Nerudiella americana

Taxon classificationAnimaliaOpilionesTriaenonychidae

(Roewer, 1961) comb. nov.

B030A9B5-FD05-5DF9-BB95-EAE01433EE2D

**Figure 68.**
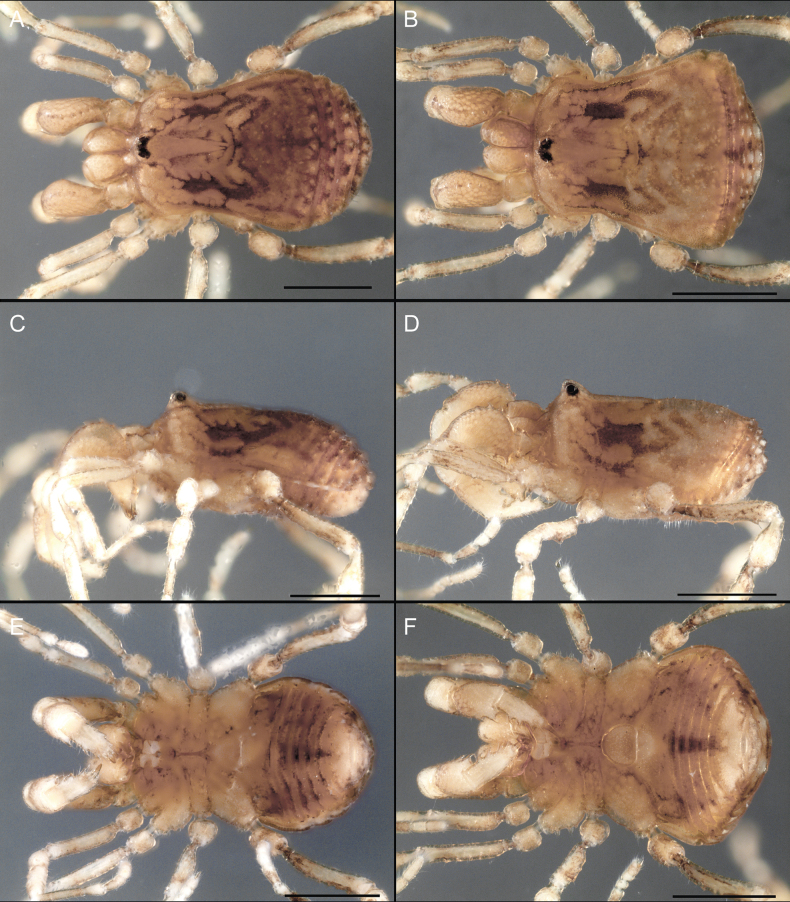
*Nerudiellaamericana* comb. nov. habitus, male **A** dorsal view **C** lateral view **E** ventral view. Female **B** dorsal view **D** lateral view **F** ventral view. Scale bars: 1 mm. Species of Clade C, see Fig. 3.

**Figure 69.**
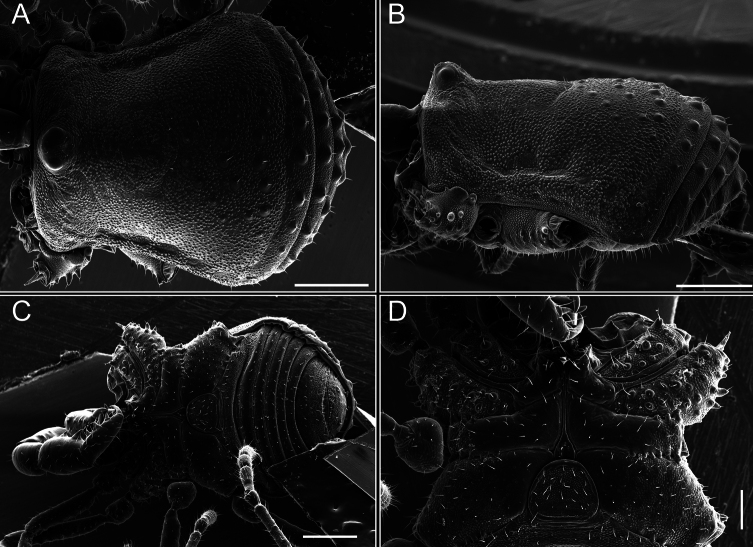
*Nerudiellaamericana* comb. nov. male, SEM images of habitus **A** dorsal view **B** lateral view **C, D** ventral view. Scale bars: 500 µm (**A, B, C**); 200 µm (**D**).

**Figure 70.**
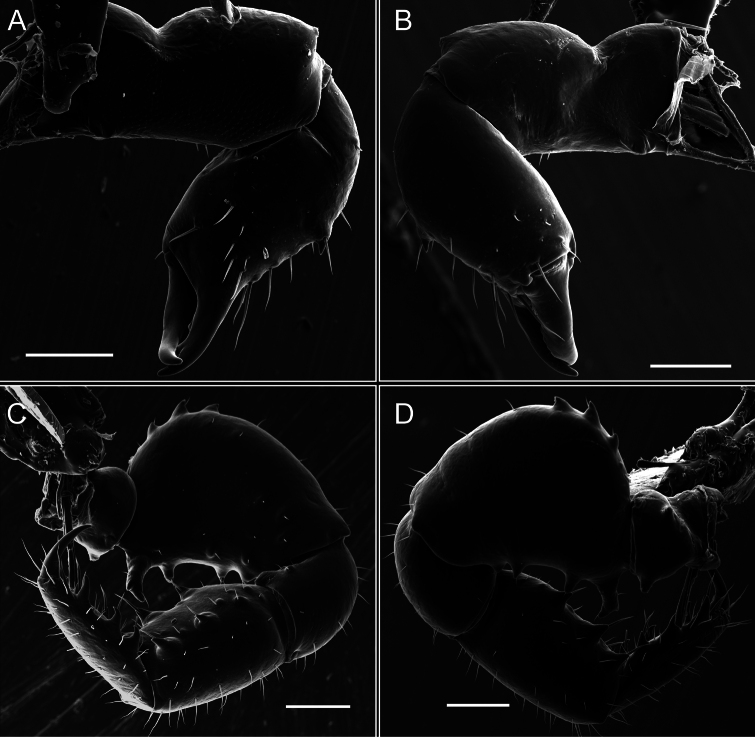
*Nerudiellaamericana* comb. nov. chelicerae: mesal **A** ectal **B** Pedipalps: mesal **C** ectal **D**. Scale bars: 200 µm.

**Figure 71.**
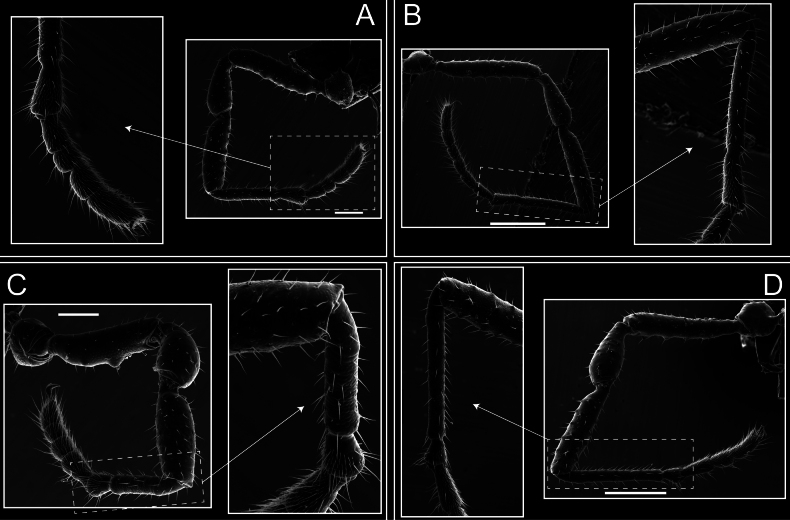
*Nerudiellaamericana* comb. nov. legs I **A** II **B** III **C** IV **D**. Scale bars: 200 µm (**A, C**); 500 µm (**B, D**).

**Figure 72.**
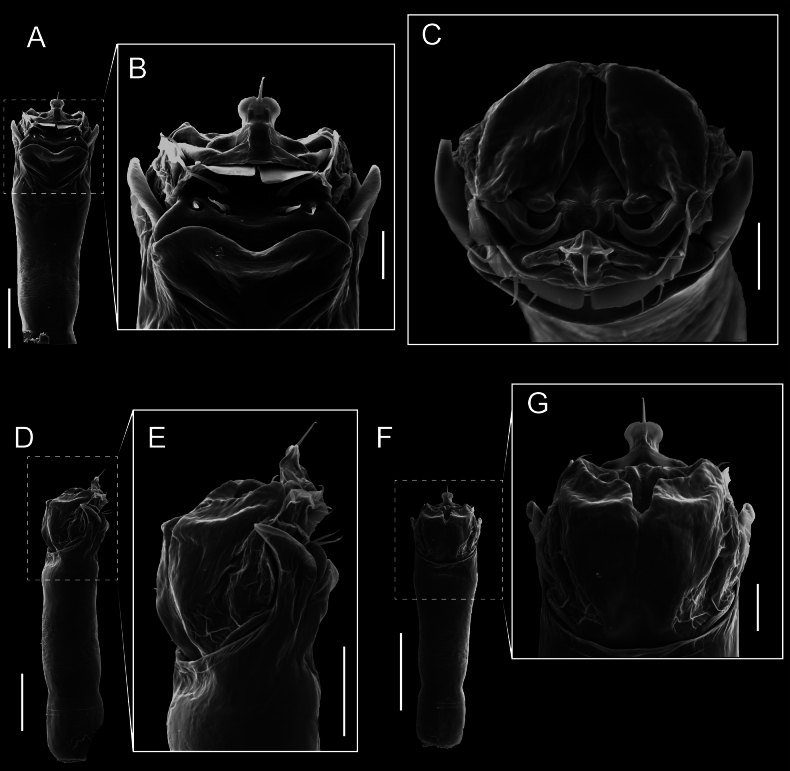
*Nerudiellaamericana* comb. nov. penis: ventral **A, B** apical **C** lateral **D, E** dorsal **F, G**. Scale bars: 200 µm (**A, D, F**); 50 µm (**B, C, G**); 100 µm (**E**).

**Figure 73.**
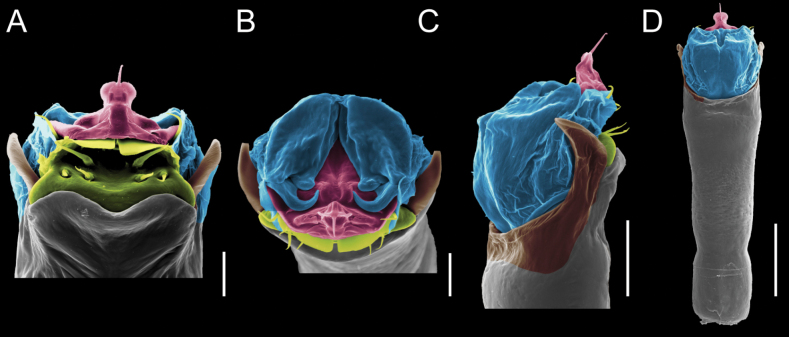
*Nerudiellaamericana* comb. nov. penis: ventral **A** apical **B** lateral **C** dorsal **D**. Colors: ventral plate (yellow), capsula externa (blue), capsula interna (red). Scale bars: 50 µm (**A, B, D**); 100 µm (**C**).

Figs 68 , 69 , 70 , 71 , 72 , 73

Nuncia americana Roewer 1961: 102, pl. 19, figs 13–15; Cekalovic 1968: 6; Besch 1969: 733; Muñoz-Cuevas 1971a: 873, fig. 28; Kury 2003: 21.
Nuncia (Nuncia) americana : Muñoz-Cuevas 1971b: 98, figs 1–17; Cekalovic 1985: 11; Pérez-Schultheiss et al. 2021: 410, fig. 2c, f.

###### Material examined.

***Paratypes*. Chile.** Chepu Peninsula, Chiloé, mixed woodland shrubs, 1 ♂, 1 ♀ (SMF RII/13414).

###### Additional material.

Chile. Chiloé: Lago Huillinco, M. Ramírez, M. Izquierdo, P. Michalik, C. Wirkner, K. Huckstorf coll., 16.II.2012, 12 imm. (MACN), 5 km of Quellón, N. Platnick, R.Schuh coll., 01.XII.1981, 4 ♂ 8 ♀ 26 imm. (AMNH), Isla Chiloé, R.Schuh, N. Platnick coll., 29.XI.1981, 4 ♂ 3 ♀ 2 imm. (AMNH), Cruce a Coinco, T.Cekalovic coll., 14.II.1983, 3 ♂ 2 ♀ 6 imm. (MACN); Mocopulli, T.Cekalovic coll., 20.II.1986, 1 ♂ 3 ♀ 5 imm. (MACN), Isla Chiloé, T.Cekalovic coll., 02.II.1983, 6 ♂ 5 ♀ 8 imm. (MACN); Isla Chiloé, T.Cekalovic coll., 26.II.1972, 3 imm. (MACN), Piruquina, T.Cekalovic coll., 26.II.1976, 1 ♂ 1 ♀ 1 imm. (MACN), Crossroad to San Pedro, T.Cekalovic coll., 27.II.1976, 4 imm. (MACN), Piruquina, T. Cekalovic coll., 10.II.1981, 2 ♂ 2 ♀ 1 imm. (MACN), same collector, 19.II.1983, 3 imm. (MACN); Chepu, 15 m, N. Platnick, O. Francke coll., 02.02.1985, 1 ♂ 2 imm. (AMNH); 15 km N. Chepu, M. Ramírez coll., 03.02.1991, 3 imm. (MACN), 15 km S de Chepu, M. Ramírez coll., 20.02.1991, 1 imm. (MACN), Lago Huillinco, M. Ramírez, M. Izquierdo, C. Wirkner, K. Huckstorf coll., 15.ii.2012, 1 ♂ (MACN), Chilloé island, 5 km N of Quellón, R.Schuh, N. Platnick coll., 01.12.1981, 1 imm. (AMNH), 8 km Ancud, Isla Chiloé, N. Platnick, O. Francke coll., 01.02.1985, 6 ♀ 22 imm. (AMNH), Isla Chiloé, 8 km S Ancud, S.Peck, J.Peck coll., 01.02.1985, 8 ♂ 5 ♀ 21 imm. (AMNH), 5 km N of Quellón, N. Platnick, R.Schuh coll., 01.12.1981, 1 ♂ 1 ♀ 1 imm. (AMNH); Chepu, E. Maury coll., 29.11.1981, 3 ♂ 1 ♀ 3 imm. (MACN). Llanquihue: Salto Petrohue, S.Peck, J.Peck coll., 23.12.1984, 1 imm. (AMNH).

###### Diagnosis.

This species stands out from the others in the genus due to its sturdy capsula externa and distinctive “T”-shaped dorsal fold. The capsula externa apex is extremely delicate. It can also be differentiated from almost all species of the genus using somatic characters (except from *N.cautin* sp. nov. and *N.jaimei* sp. nov.) by having the dorsal scutum with a low, broad-based tubercles.

###### Distribution.

Chile: Los Lagos Region (Fig. 4F).

###### Redescription of male

**paratype SMF RII/13414.** Measurements: Total length 2.73, dorsal scutum length 2.21, carapace max. width 1.47, mesotergum max. width 1.95. Appendage measurements: Pedipalps. Trochanter length 0.28, femora length 0.97, patella length 0.61, tibia length 0.74, tarsus length 0.71. Leg I: trochanter (tr) 0.26, femora (fe) 0.92, patella (pa) 0.54, tibia (ti) 0.69, metatarsus (mt) 0.82, tarsus (ta) 0.70. II: tr 0.32, fe 1.16, pa 0.57, ti 0.93, mt 1.07, ta 1.14. III: tr 0.30, fe 0.71, pa 0.43, ti 0.65, mt 0.59, ta 0.72.IV: tr 0.39, fe 1.08, pa 0.70, ti NA, mt NA, ta NA.

Dorsum (Fig. 68, 69). Eta (η) dorsal scutum in the shape of an hourglass. Ocularium slightly elevated. Dorsal scutum microgranulate with a group of low setiferous granules. Areas on scutum not clearly delimited. Along the back margin, with a row of low and broad setiferous granules. Free tergites with a row of low and broad setiferous granules.

Chelicerae (Fig. 70A, B). Segment I smooth in texture without prominent tubercles. Segment II with a small frontal tubercle and covered with scattered setae, giving it a textured appearance.

Pedipalps (Fig. 70C, D). Coxa I with a smooth surface without any notable tubercles. Trochanter with small tubercles on both the dorsal and ventral sides. In dorsal view, femora with three spines with subdistal setae. In ventral view, with one long bifurcated spine and two spines with subdistal setae. In mesal view, with six low setiferous tubercles on the femora. Patella with a mesal row of setiferous tubercles. Tibia with three ectal and two mesal spines with subdistal setae, and six low setiferous tubercles. Tarsus with three mesal and ectal spines with setae, with a few setae.

Legs (Fig. 71). Leg I with sparse setiferous tubercles. Leg II features two rows of setiferous tubercles, while legs III and IV exhibit only microgranulation without distinct tubercles. With three bridges connecting legs II and III, six bridges between legs III and IV, and four bridges connecting leg IV to the opisthosoma. Distal bridge longer than the others. Bridges do not obstruct the spiracles, although they appear partially covered with a subdistal tubercle. A smooth surface occupies ~ ¼ of leg II, ¾ of leg III, and 1/3 of leg IV. Sternum arrow-shaped. Femora III with a remarkable triangular tubercle. Leg IV with a row of four subtriangular tubercles. Legs I–IV covered in setae, with tarsal area and calcaneus densely setose. Calcaneus smaller than astragalus, ~ 3× smaller in legs I–III, 4× smaller in leg IV. Tarsal count: 4–6–4–4.

Penis (Figs 72, 73). Pars distalis with a prominent ventral plate divided into two lamellae by a cleft. Each lamella equipped with three pointed macrosetae on the ventral surface and one on the dorsal surface. Dorsal and lateral surfaces covered by a capsula externa, with a cleft that separates the capsula externa into two halves. With an additional dorsolateral plate attached to the pars basalis. Capsula interna longer than capsula externa and partially covers ventral plate.

**Female.** Similar to male, with shorter pedipalpal femora.

Female measurements. Total length 2.88, carapace length 1.17, dorsal scutum length 2.21, carapace max. width 1.47, mesotergum max. width 2.07. Appendage Measurements: Pedipalps. Trochanter length 0.23, femora length 0.74, patella length 0.46, tibia length 0.58, tarsus length 0.62. Leg I: trochanter (tr) 0.21, femora (fe) 0.88, patella (pa) 0.50, tibia (ti) 0.65, metatarsus (mt) 0.79, tarsus (ta) 0.65. II: tr 0.32, fe 1.12, pa 0.56, ti 0.90, mt 0.98, ta 1.18. III: tr 0.32, fe 0.81, pa 0.46, ti 0.59, mt 0.67, ta 0.64. IV: tr 0.35, fe 1.16, pa 0.70, ti 0.89, mt 1.21, ta 0.87. Tarsal count: 3–6–4–4.

##### ﻿ Nerudiella cachai sp. nov.

Taxon classificationAnimaliaOpilionesTriaenonychidae

7B845AFC-A114-518D-B53C-DE8C09CA8986

https://zoobank.org/27F81489-12E3-4DC3-858D-5DF1C0ECB048

**Figure 74.**
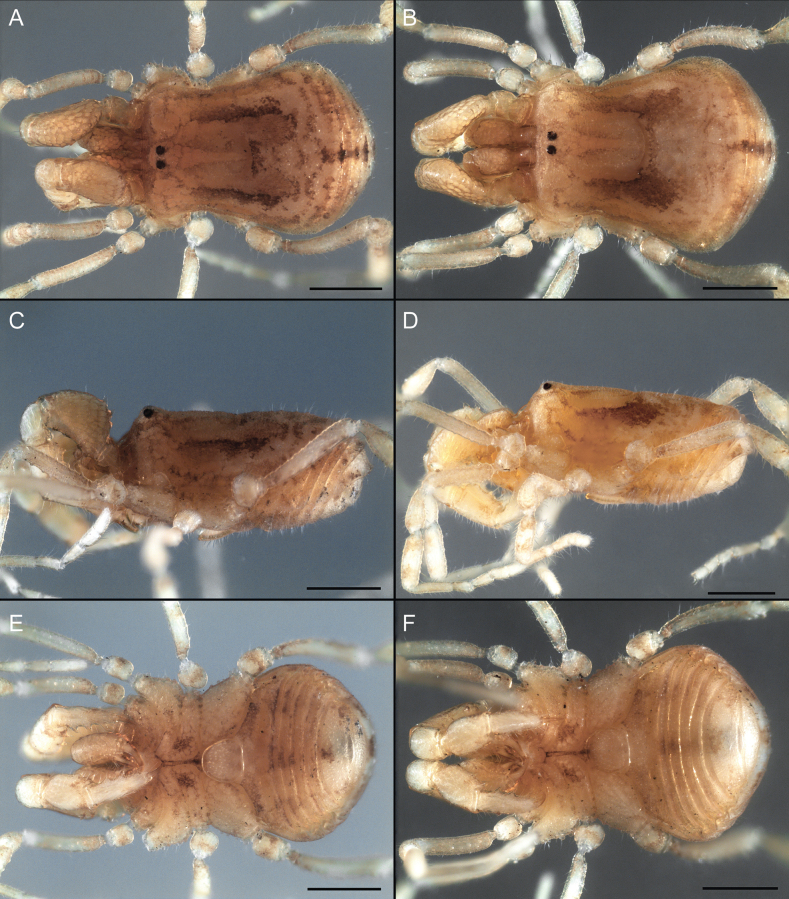
*Nerudiellacachai* sp. nov. habitus, male **A** dorsal view **C** lateral view **E** ventral view. Female **B** dorsal view **D** lateral view **F** ventral view. Scale bars: 500 µm. Species of Clade C, see Fig. 3

**Figure 75.**
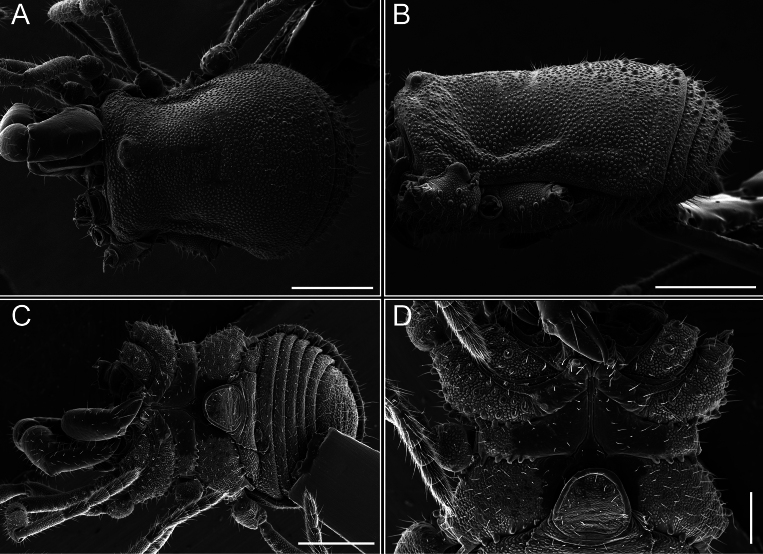
*Nerudiellacachai* sp. nov. male, SEM images of habitus **A** dorsal view **B** lateral view **C, D** ventral view. Scale bars: 500 µm (**A, B, C**); 200 µm (**D**).

**Figure 76.**
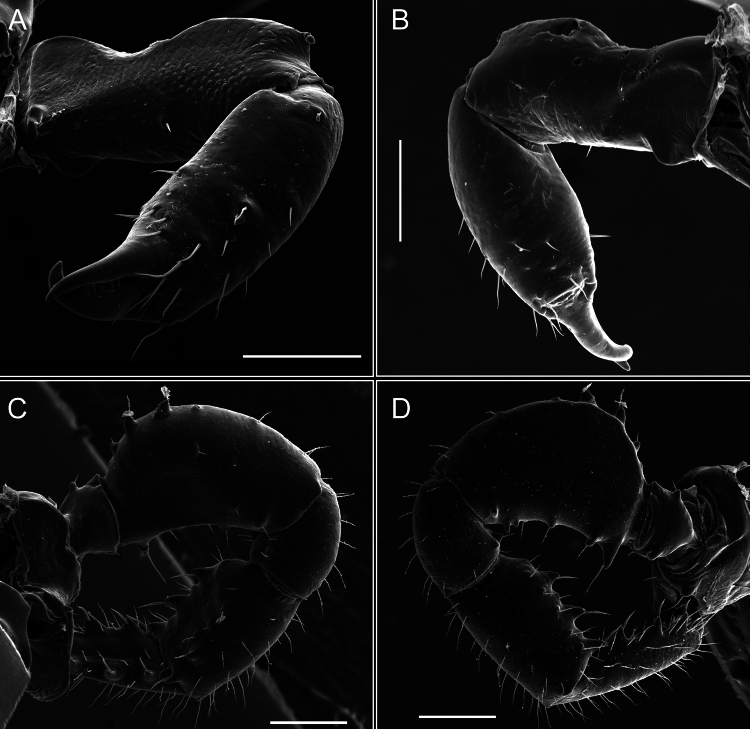
*Nerudiellacachai* sp. nov. chelicerae: mesal **A** ectal **B** pedipalps: mesal **C** ectal **D**. Scale bars: 200 µm.

**Figure 77.**
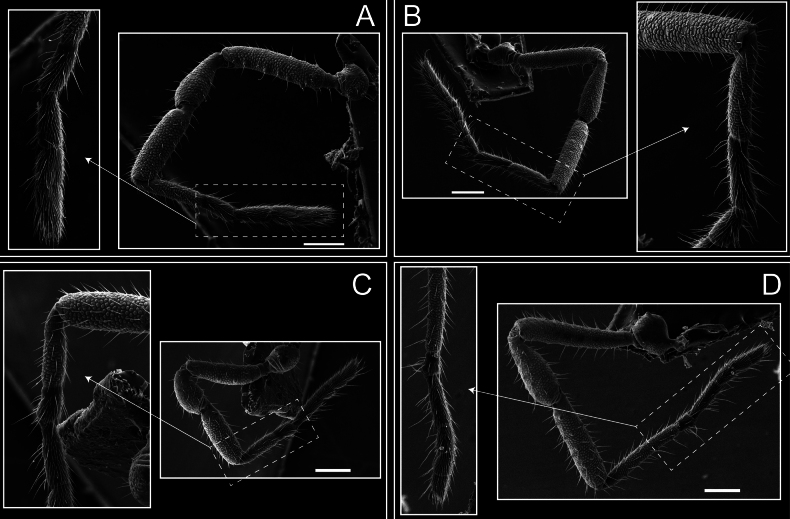
*Nerudiellacachai* sp. nov. legs I **A** II **B** III **C** IV **D**. Scale bars: 200 µm.

**Figure 78.**
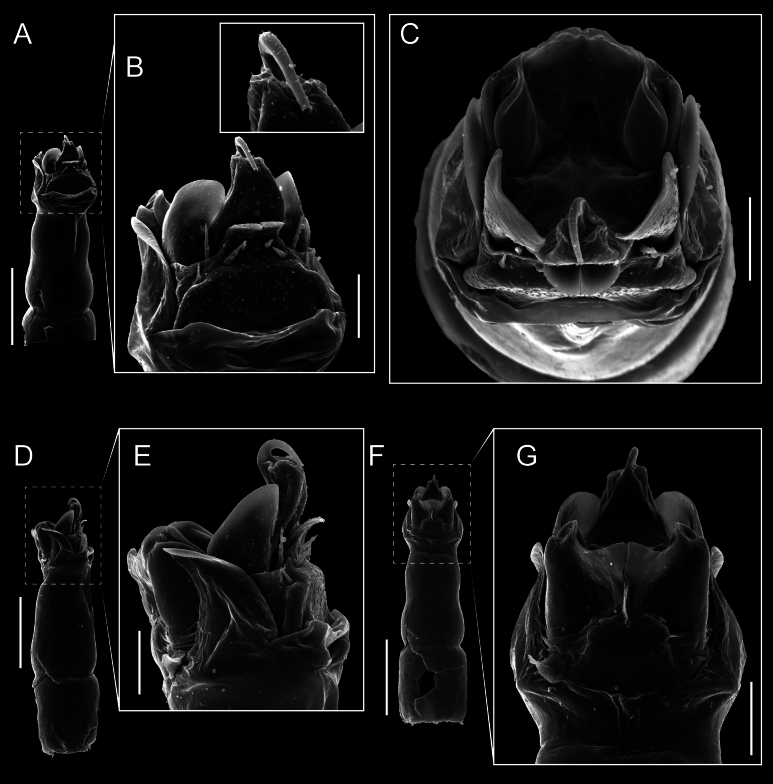
*Nerudiellacachai* sp. nov. penis: ventral **A, B** apical **C** lateral **D, E** dorsal **F, G**. Scale bars: 200 µm (**A, D, F**); 50 µm (**B, C, E, G**).

**Figure 79.**
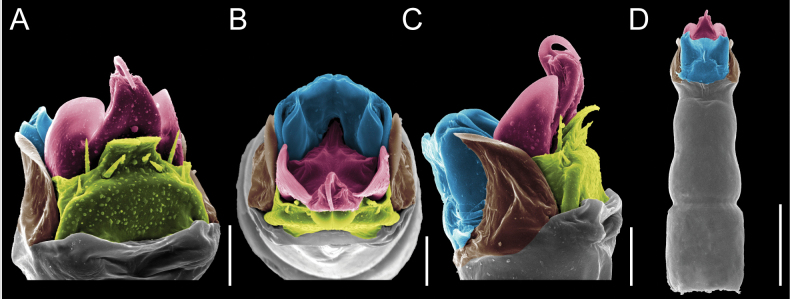
*Nerudiellacachai* sp. nov. penis: ventral **A** apical **B** lateral **C** dorsal **D**. Colors: ventral plate (yellow), capsula externa (blue), capsula interna (red). Scale bars: 50 µm.

Figs 74 , 75 , 76 , 77 , 78 , 79

###### Material examined.

***Holotype*.** ♂ **Chile.** Concepción: Estero Nonguén, 36.82106°S, 73.01649°W, T. Cekalovic coll., 21.IV.1976 (MNHNCL). ***Paratypes*. Chile.** Concepción: Estero Nonguén, T.Cekalovic coll., 13.III.1977, 7 ♂ 2 ♀ (MACN). Same locality, T.Cekalovic coll., 16.IV.1977, 1 ♀ 1 imm. (MACN). Same locality, 1250 m, N. Platnick, R.Schuh coll., 19.11.1981, 2 ♂ 1 imm. (AMNH). Agua de la Gloria, T.Cekalovic coll., 14.VIII.1978, 1 ♂ (MACN).

###### Additional material.

Chile. Arauco. Caramávida, San Alfonso, Quebrada Caramávida, San Alfonso, Arauco reserve, 37.70942°S, 73.17107°W, 750 m, A. Ojanguren, P. Goloboff, M. Ramírez, G. Azevedo, W. Porto coll., 15.I.2018, 30 imm. (MACN). 16 km N Tres Pinos, 37.54143°S, 73.41555°W, A. Newton, M. Thayer coll., 12.XII.1982, 2 ♂ (AMNH). Concepción. Penco, 36.734°S, 72.98006°W, T.Cekalovic coll., 23.IV.1977, 5 ♂ 5 ♀ 2 imm. (MACN). Cerro Caracol, 36.83465°S, 73.0488°W, T.Cekalovic coll., 10.IV.1977, 6 ♂ 1 ♀ (MACN). Estero Nonguén, 36.82096°S, 73.0164°W, T.Cekalovic coll., 03.XII.1982, 11 imm. (MACN). Esquadrón, 36.9824°S, 73.14165°W, T.Cekalovic coll., 10.08.1978, 1 imm. (MACN). Estero Nonguén, 36.82096°S, 73.0164°W, T.Cekalovic coll., 19.VIII.1978, 2 imm. (MACN). El Manzano, Camino de Santa Juana, 36.86677°S, 72.78498°W, T.Cekalovic coll., 13.I.1985, 1 ♂ (MACN). Colcura, 37.11386°S, 73.14724°W, T.Cekalovic coll., 30.I.1985, 1 imm. (MACN). Hualpén, Terrestrial Biology Station Univ. de Concepción, 36.79821°S, 73.16307°W, 52 m, A. Ojanguren, A. Pérez-González, M. Ramírez, G. Azevedo, W. Porto coll., 14.I.2018, 1 ♀ 3 mm (MACN). Estero Nonguén, 36.82129°S, 73.0164°W, N. Platnick, R.Schuh coll., 16.XI.1981, 6 ♂ 2 ♀ 6 imm. (AMNH). 8.4 km W La Florida, 36.84976°S, 72.78826°W, 170 m, A. Newton, M. Thayer coll., 02.I.1983, 3 ♀ (AMNH). Dichoco. Estero Bellavista, 36.71992°S, 72.91118°W, T.Cekalovic coll., 04.I.1984, 2 ♂ 4 ♀ 4 imm. (MACN). 6 km S San Pedro, 36.90616°S, 73.10894°W, N. Platnick, O. Francke coll., 22.I.1985, 2 ♂ 2 imm. (AMNH). Dichoco. Estero Bellavista, 36.71992°S, 72.91118°W, T.Cekalovic coll., 04.I.1984, 1 ♀ 1 imm. (MACN). Parque Nacional Nahuelbuta, T.Cekalovic coll., 19.XI.1981, 2 ♂ 1 ♀ (MACN). Estero Nonguén, 36.82096°S, 73.0164°W, T.Cekalovic coll., 03.XI.1982, 1 ♂ 4 imm. (MACN).

###### Etymology.

The specific epithet cachai refers to a popular expression in Chile that is roughly translated to “you know what I mean?” “am I right?”, or “get it?”. It is derived from the English term “to catch,” which refers to catching but is also used to mean “understanding or understanding something.” Noun in apposition.

###### Diagnosis.

This species can be easily distinguished from other species of the genus by its male genitalia, which exhibits a highly reduced ventral plate, with a stylus that is formed by a thin and slender tube.

###### Distribution.

Chile: Bío-Bío Region.

###### Description of male holotype.

Measurements: Total length 2.26, carapace length 0.92, dorsal scutum length 1.84, carapace max. width 1.23, mesotergum max. width 1.70. Appendage measurements: Pedipalps. Trochanter length 0.23, femora length 0.91, patella length 0.44, tibia length 0.68, tarsus length 0.59. Leg I: trochanter (tr) 0.22, femora (fe) 1.10, patella (pa) 0.51, tibia (ti) 0.79, metatarsus (mt) 0.92, tarsus (ta) 0.92. II: tr 0.27, fe 1.33, pa 0.57, ti 1.00, mt 1.22, ta 1.45. III: tr 0.21, fe 0.91, pa 0.31, ti 0.73, mt 0.75, ta 0.74. IV: tr 0.32, fe 1.29, pa 0.59, ti 1.04, mt 1.30, ta 0.86.

Dorsum (Fig. 74, 75). Eta (η) dorsal scutum in the shape of an hourglass. Ocularium low. Dorsal scutum microgranulate, with no clear delimitation of distinct areas. Areas III and IV with irregular rows of small rounded setiferous tubercles; posterior margin with a similar irregular row of small rounded setiferous tubercles. All free tergites covered in small setae and with a row of small rounded setiferous tubercles.

Chelicerae (Fig. 76A, B). Segment I with a small tubercle on its dorso-distal surface. Segment II with a small frontal tubercle and scattered setae.

Pedipalps (Fig. 76C, D). Trochanter characterized by the presence of two small dorsal tubercles and one ventral tubercle. In dorsal view, femora with three spines with subdistal setae; in ventral view, with five tubercles with subdistal setae, with four mesal setiferous tubercles and one ectal setiferous tubercle positioned beneath. Patella with both mesal and ectal setiferous tubercles. Tibia with three ectal and two mesal spines with subdistal setae. Tarsus with three mesal and ectal spines, accompanied by scattered setae.

Legs (Fig. 77). Segments I and II with two rows of setiferous tubercles; segments III and IV microgranulate but without setiferous tubercles. With three bridges connecting legs II and III, six bridges between III and IV, and four bridges between leg IV and the opisthosoma. Distal bridge longer than the others. The bridges do not obstruct the spiracles. The smooth area occupies ~ ¼ of leg II, ¾ of leg III, and < 1/3 of leg IV. Sternum arrow-shaped, with a triangular-shaped posterior margin. Legs I–IV covered with setae, the area of the tarsus and calcaneus are densely setose. Femora I–IV with a ventral row of setiferous granules. Calcaneus smaller than astragalus, ≥ 3× smaller (legs I, III) of the same size (II) and 4× smaller (leg IV). Tarsal count: 3–6–4–4.

Penis (Figs 78, 79). Pars distalis with a small ventral plate divided into two distinct small lamellae by a central cleft. Each lamella adorned with three pointed macrosetae on its ventral surface, with a single macroseta on its dorsal surface. Additionally, there is a capsula externa, characterized by its square shape, which covers the dorsal and lateral surfaces. Attached to the pars basalis is a dorsolateral plate. The capsula interna, longer in length compared to the capsula externa, partially overlays the ventral plate. Apex of the capsula interna with a tubular stylus.

**Female.** Pedipalp femora noticeably smaller than those of males.

Female measurements. Total length 2.30, carapace length 0.87, dorsal scutum length 1.85, carapace max. width 1.24, mesotergum max. width 1.75. Appendage Measurements: Pedipalps. Trochanter length 0.17, femora length 0.72, patella length 0.35, tibia length 0.54, tarsus length 0.57. Leg I: trochanter (tr) 0.22, femora (fe) 0.73, patella (pa) 0.43, tibia (ti) 0.53, metatarsus (mt) 0.65, tarsus (ta) 0.60. II: tr 0.28, fe 0.97, pa 0.49, ti 0.75, mt 0.82, ta 1.02. III: tr 0.22, fe 0.61, pa 0.38, ti 0.53, mt 0.57, ta 0.56. IV: tr 0.34, fe 0.90, pa 0.55, ti 0.76, mt 0.91, ta 0.75.

##### ﻿ Nerudiella caramavida sp. nov.

Taxon classificationAnimaliaOpilionesTriaenonychidae

F8C8242E-C12E-5790-AD42-70EBFC0EC275

https://zoobank.org/45A9D45E-90CE-43D9-9223-BD691DA31146

**Figure 80.**
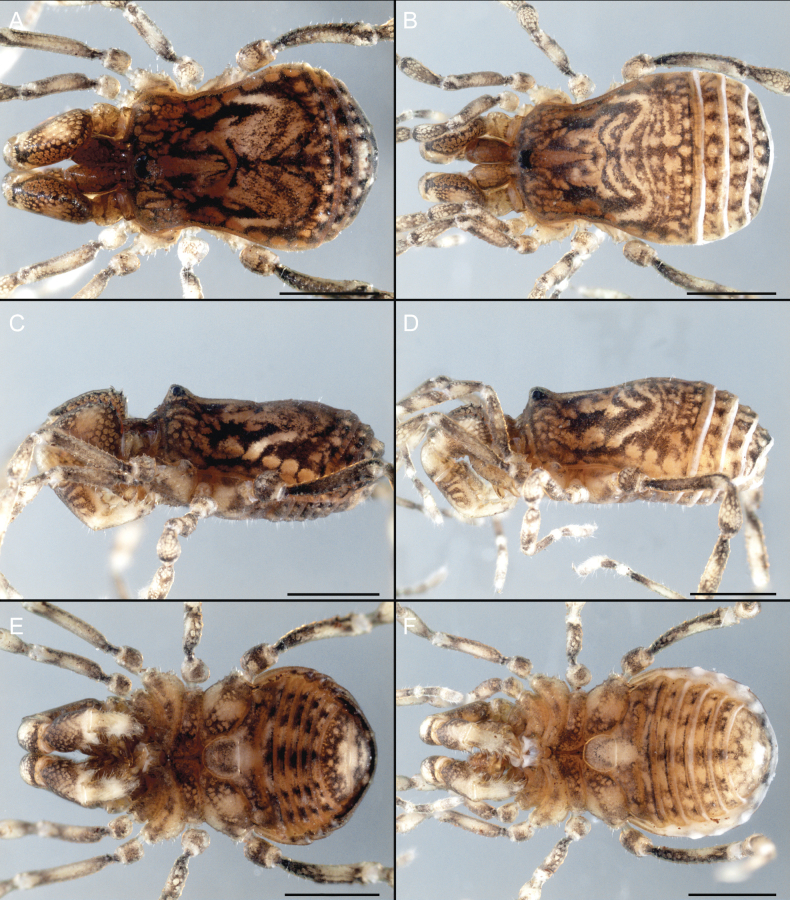
*Nerudiellacaramavida* sp. nov. habitus, male **A** dorsal view **C** lateral view **E** ventral view. Female **B** dorsal view **D** lateral view **F** ventral view. Scale bars: 1 mm. Species of Clade C, see Fig. 3.

**Figure 81.**
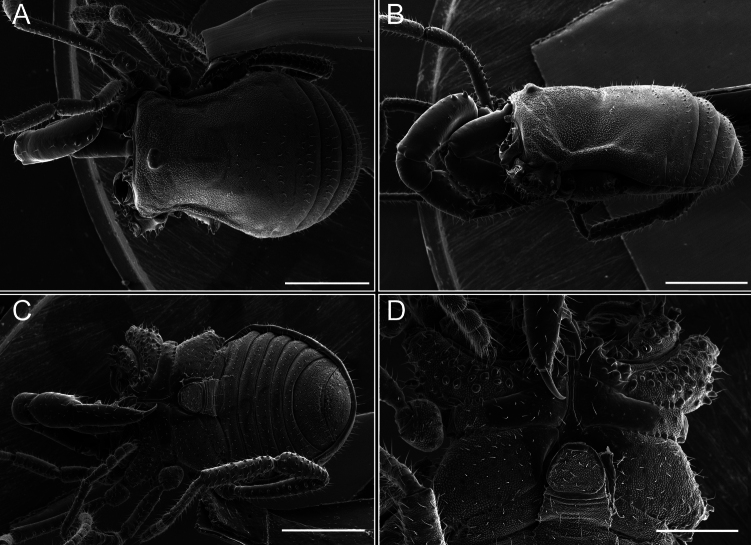
*Nerudiellacaramavida* sp. nov. male, SEM images of habitus **A** dorsal view **B** lateral view **C, D** ventral view. Scale bars: 1 mm (**A, B, C**); 500 µm (**D**).

**Figure 82.**
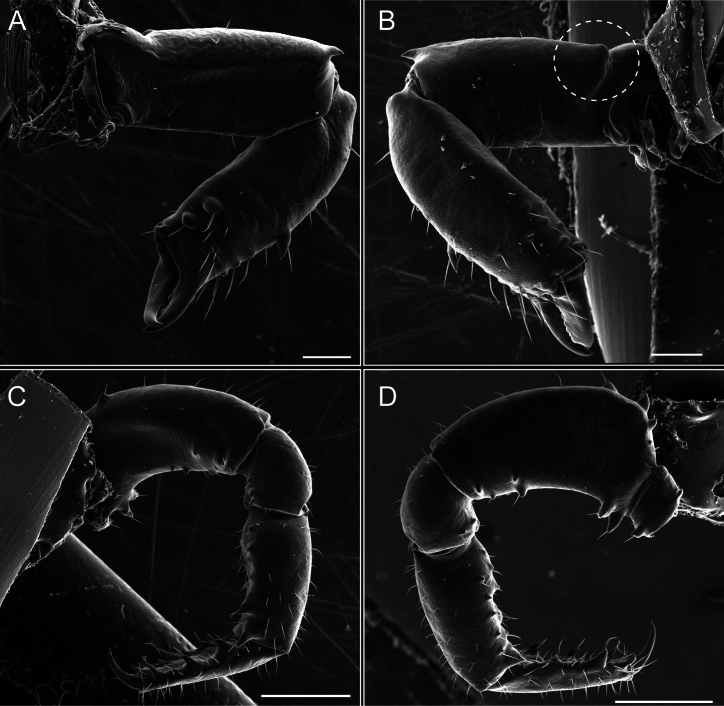
*Nerudiellacaramavida* sp. nov. chelicerae: mesal **A** ectal **B** pedipalps: mesal **C** ectal **D**. Scale bars: 200 µm (**A, B**); 500 µm (**C, D**).

**Figure 83.**
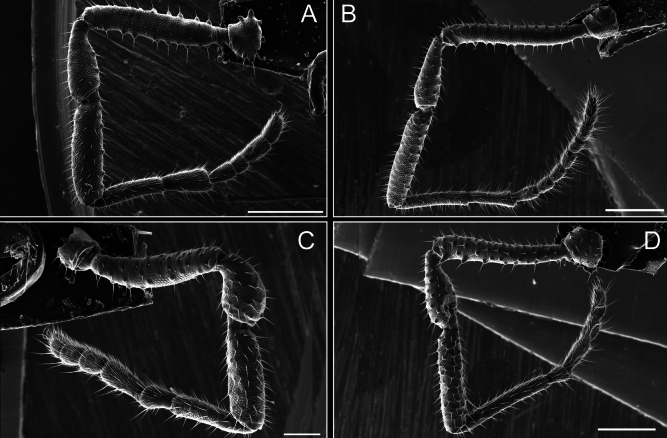
*Nerudiellacaramavida* sp. nov. legs I **A** II **B** III **C** IV **D**. Scale bars: 500 µm (**A, B, D**); 200 µm (**C**).

**Figure 84.**
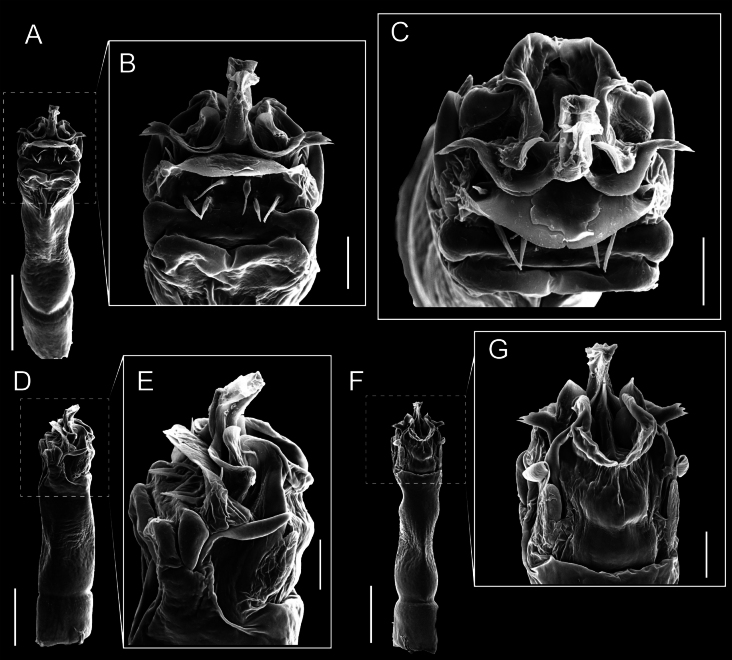
*Nerudiellacaramavida* sp. nov. penis: ventral **A, B** apical **C** lateral **D, E** dorsal **F, G**. Scale bars: 200 µm (**A, D, F**); 50 µm (**B, C, E, G**).

**Figure 85.**
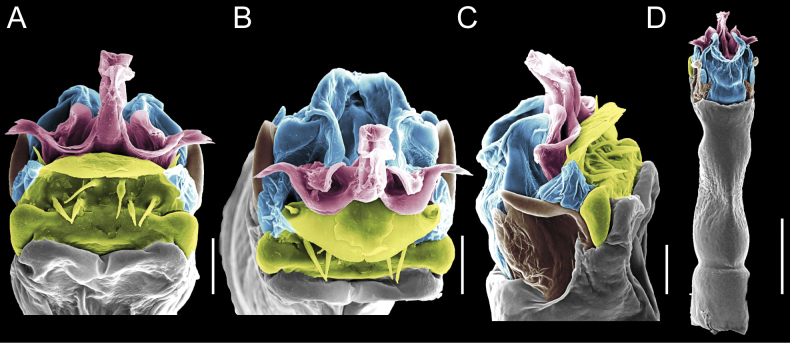
*Nerudiellacaramavida* sp. nov. penis: ventral **A** apical **B** lateral **C** dorsal **D**. Colors: ventral plate (yellow), capsula externa (blue), capsula interna (red). Scale bars: 50 µm (**A–C**); 200 µm (**D**).

Figs 80 , 81 , 82 , 83 , 84 , 85

###### Material examined.

***Holotype*.** ♂ **Chile.** Malleco: P.N. Nahuelbuta, M. Ramírez, F. Labarque, 12.II.2005 (MNHNCL). ***Paratypes*. Chile.** Malleco: P.N. Nahuelbuta, M. Ramírez, F. Labarque, 12.II.2005, 9 ♂ 7 ♀ 3 imm. (MACN), Malleco: P.N. Nahuelbuta, 1250 m, N. Platnick, R.Schuh. 19.XI.1981, 1 ♂ 2 ♀ 6 imm. (AMNH). Ñuble: 2 km E de Las Trancas, E. Maury. 09.I.1989, 1 ♂ 1 ♀ (MACN).

###### Additional material.

Chile. Arauco. Hualpén, Univ. Concepción Terrestrial Biology Station, 36.79821°S, 73.16307°W, 52 m, A. Ojanguren, A. Pérez-González, M. Ramírez, G. Azevedo, W. Porto coll., 14.I.2018, 1 ♂ (MACN). Caramávida, San Alfonso, Quebrada Caramávida, Arauco Reserve, 37.70942°S, 73.17107°W, 750 m, A. Ojanguren, A. Pérez-González, M. Ramírez, G. Azevedo, W. Porto coll., 3 mm. 14.I.2018, 1 ♀, 1 ♂ 3.

###### Etymology.

The specific epithet “*caramavida*” is derived from the type locality of the species, Quebrada Caramávida, located on the western slope of the Cordillera de Nahuelbuta in the Bío-Bío Region. The choice of this name, a noun in apposition, is based on the geographic location where the species was originally discovered.

###### Diagnosis.

This species can be easily distinguished from its congeners by the capsula interna of the male genitalia, which has two long lateral processes.

###### Distribution.

Chile: Bío-Bío Region (Fig. 4F).

###### Description of male holotype.

Measurements: Total length 2.74, carapace length 1.1, dorsal scutum length 2.05, carapace max. width 1.37, mesotergum max. width 1.96. Appendage measurements: Pedipalps. Trochanter length 0.20, femora length 0.92, patella length 0.40, tibia length 0.62, tarsus length 0.83. Leg I: trochanter (tr) 0.20, femora (fe) 0.85, patella (pa) 0.46, tibia (ti) 0.62, metatarsus (mt) 0.78, tarsus (ta) 0.65. II: tr 0.25, fe 1.84, pa 0.57, ti 0.90, mt 1.31, ta 1.29. III: tr 0.31, fe 0.79, pa 0.38, ti 0.64, mt 0.66, ta 0.60. IV: tr 0.31, fe 1.08, pa 0.63, ti 0.88, mt 1.04, ta 0.82.

Dorsum (Fig. 80, 81). Eta (η) hourglass-shaped dorsal scutum. Ocularium low and rounded, pointing a bit forward. Dorsal scutum microgranulate. Mesotergal areas of dorsal scutum not well delimited; with small setiferous tubercles. Free tergites with two rows of small setiferous tubercles.

Chelicerae (Fig. 82A, B). Segment I with an acute tubercle on the dorso-distal surface. Segment II with two mesal setiferous tubercles and 7–8 small setiferous tubercles, with one triangular tubercle more prominent than others in frontal view.

Pedipalps (Fig. 82C, D). Trochanter with tiny dorsal and ventral tubercles. Femora with two robust ventral-proximal tubercles and four minor ventral-distal tubercles. Additionally, with a row of setiferous tubercles along dorsal surface of femora, with the three largest tubercles located in the proximal section. Patella with two small ventral-ectal tubercles and two small ventral-mesal tubercles. Tibia with two rows of weaker apical tubercles and two rows of minor ventral tubercles. Tarsus characterized by three mesal and ectal spines; Also, with a few setae and subdistal setae in this region.

Legs (Fig. 83). Segment I with nine or ten setiferous tubercles; segment II with 18–20 setiferous tubercles; segment IV with five or six small tubercles connected to the opisthosoma. Spiracles not obstructed by bridges. Smooth areas with ~ 1/3 of leg II smooth, with two or three small tubercles on each side featuring subdistal setae. The smooth portion extends to ¾ of leg III and < 1/3 of leg IV. Sternum arrow-shaped. Legs smooth, with notch in tarsus I. Tarsal count: 3–6/7–4–4.

Penis (Figs 84, 85). Pars distalis with a large ventral plate bearing a cleft that divides the plate into two halves. Each half with three pointed macrosetae on the ventral surface and one macroseta on the dorsal surface. Capsula externa covering dorsal and lateral surface, its apical region U-shaped. With a dorsolateral plate attached to the pars basalis. Capsula interna longer than the capsula externa, which has a long lateral process, perpendicular to the axis of the genitalia, with a visible stylus in its apical portion.

**Female.** Similar to male, with shorter pedipalpal femora.

Female measurements. Total length 2.6, carapace length 1.13, dorsal scutum length 2.1, carapace max. width 1.39, mesotergum max. width 1.98. Appendage measurements: Pedipalps. Trochanter length 0.26, femora length 0.99, patella length 0.46, tibia length 0.70, tarsus length 0.87. Leg I: trochanter (tr) 0.24, femora (fe) 1.04, patella (pa) 0.54, tibia (ti) 0.79, metatarsus (mt) 0.94, tarsus (ta) 0.78. II: tr 0.26, fe 1.51, pa 0.64, ti 1.08, mt 1.26, ta 1.44. III: tr 0.31, fe 0.93, pa 0.41, ti 0.69, mt 0.78, ta 0.78. IV: tr 0.32, fe 1.26, pa 0.61, ti 1.09, mt 1.28, ta 0.97.

##### ﻿ Nerudiella cautin sp. nov.

Taxon classificationAnimaliaOpilionesTriaenonychidae

E16A1BE4-5AAD-5ABB-980F-3F5845157001

https://zoobank.org/49BC3855-C82F-42EC-81D3-F4D32C7B23B4

**Figure 86.**
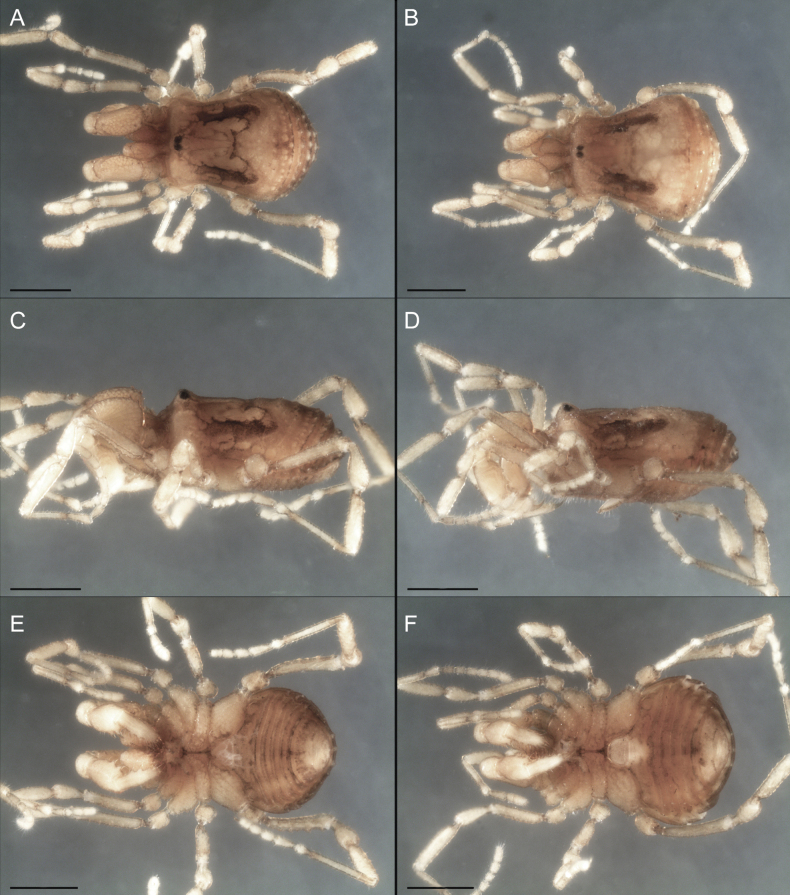
*Nerudiellacautin* sp. nov. habitus, male **A** dorsal view **C** lateral view **E** ventral view. Female **B** dorsal view **D** lateral view **F** ventral view. Scale bars: 1 mm.

**Figure 87.**
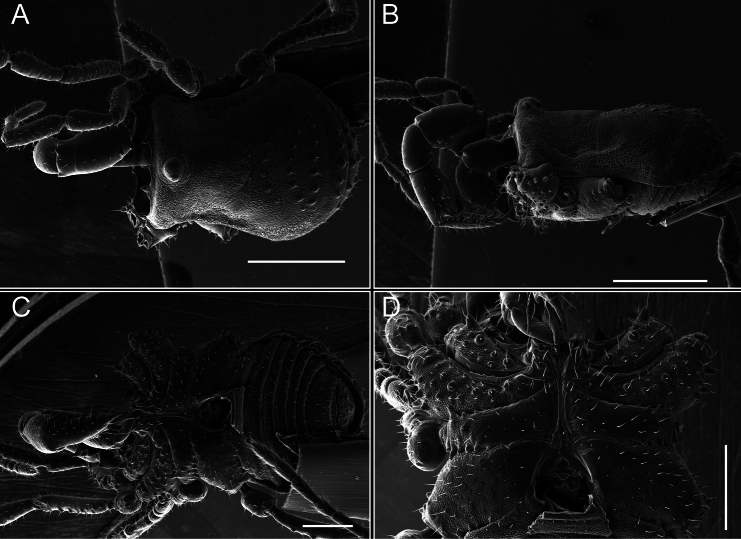
*Nerudiellacautin* sp. nov. male, SEM images of habitus **A** dorsal view **B** lateral view **C, D** ventral view. Scale bars: 500 µm.

**Figure 88.**
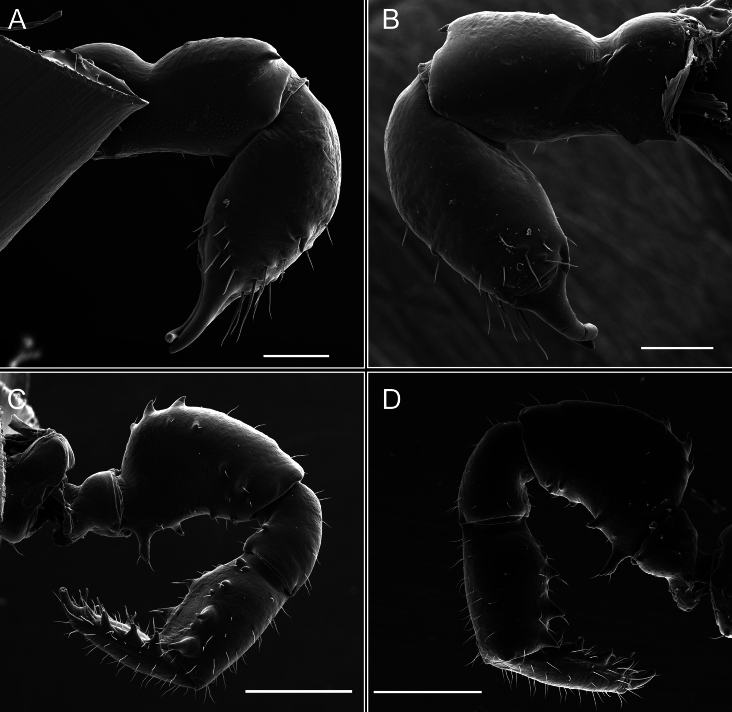
*Nerudiellacautin* sp. nov. chelicerae: mesal **A** ectal **B** pedipalps: mesal **C** ectal **D**. Scale bars: 200 µm (**A, B**); 500 µm (**C, D**).

**Figure 89.**
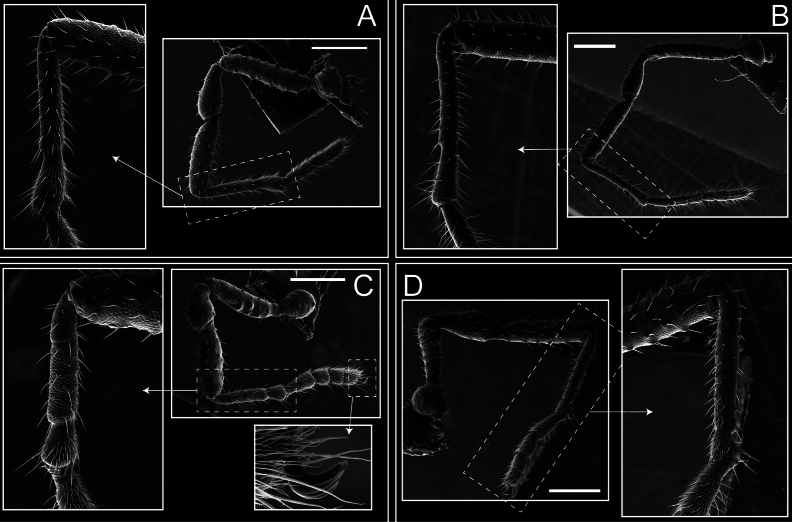
*Nerudiellacautin* sp. nov. legs I **A** II **B** III **C** IV **D**. Scale bars: 500 µm.

**Figure 90.**
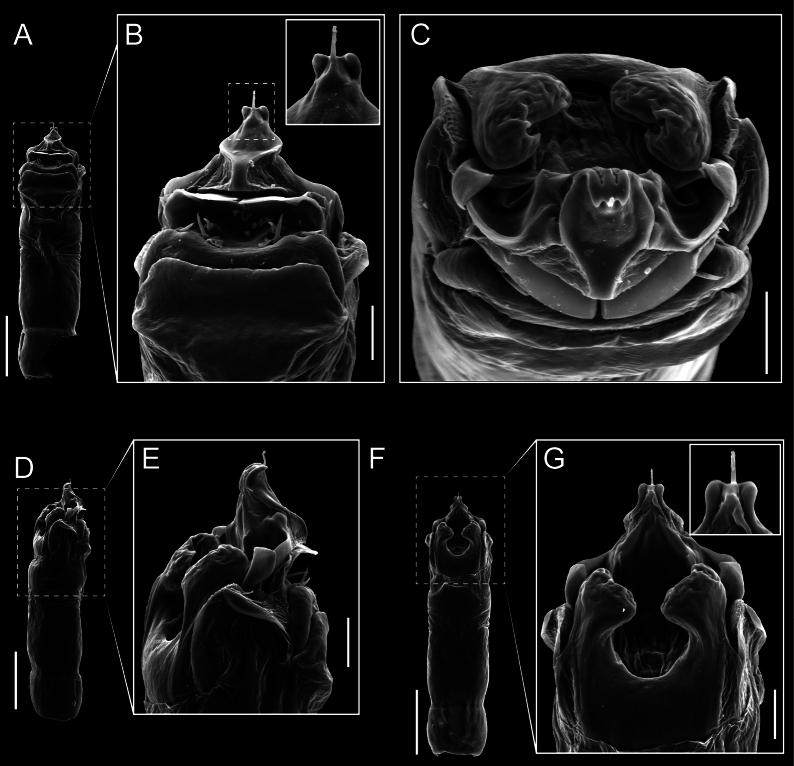
*Nerudiellacautin* sp. nov. penis: ventral **A, B** apical **C** lateral **D, E** dorsal **F, G**. Scale bars: 200 µm (**A, D, F**); 50 µm (**B, C, E, G**).

**Figure 91.**
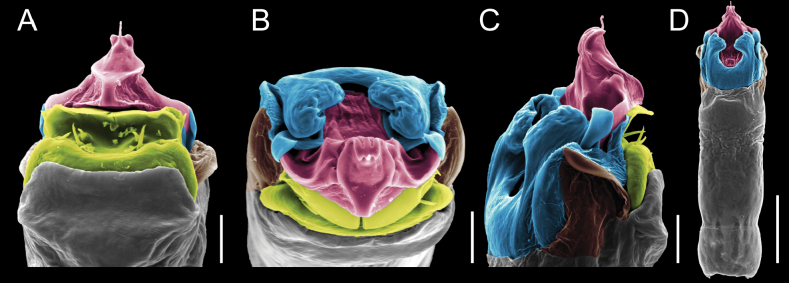
*Nerudiellacautin* sp. nov. penis: ventral **A** apical **B** lateral **C** dorsal **D**. Colors: ventral plate (yellow), capsula externa (blue), capsula interna (red). Scale bars: 50 µm.

Figs 86 , 87 , 88 , 89 , 90 , 91

###### Material examined.

***Holotype*.** ♂ **Chile.** Región de Los Lagos, Llanquihue, 13 km W Río Negro. Berlese. N. Platnick, R.Schuh coll., coll, 24.I.1986 (AMNH). ***Paratypes*. Chile.** Palena, Vicinity of Chaitén, 0–100 m. Berlese. N. Platnick, R.Schuh 5–7.XII.1981 2 ♂ 2 ♀ 7 imm. (AMNH). Same data, 1 ♀ 3 imm. (AMNH). Los Lagos, Palena, 25–27 km North Chaitén, 40 m. P. Goloboff, R.Schuh 17.I.1986 2 ♂ (AMNH). Coihaique, 10 km N Reserva Nacional Coyhaique. S.Peck, J.Peck. 22.I.1985 1 ♀ (FMNH). Aysén, 30 km N Puerto Cisnes. E. Maury . 09.XII.1986 (MACN).

###### Etymology.

The specific epithet refers to the type locality of the species, the province of Cautín, located in the southern zone of Chile. Noun in apposition.

###### Diagnosis.

The sharp tubercles on the dorsal scutum surface distinguish this species from others its congeners, particularly when observed from a dorsal view. The genitalia shows a unique U-shaped capsula externa (Fig. 91D).

###### Distribution.

Chile: Bío-Bío, Araucanía, Los Ríos, and Los Lagos Regions (Fig. 4F).

###### Description of male holotype.

Total length 4.17, carapace length 1.26, dorsal scutum length 2.64, carapace max. width 1.51, mesotergum max. width 2.06. Appendage measurements: Pedipalps. Trochanter length 0.35, femora length 1.05, patella length 0.58, tibia length 0.79, tarsus length 0.73. Leg I: trochanter (tr) 0.22, femora (fe) 0.83, patella (pa) 0.48, tibia (ti) 0.67, metatarsus (mt) 0.80, tarsus (ta) 0.71. II: tr 0.23, fe 1.17, pa 0.64, ti 0.89, mt 1.03, ta 1.33. III: NA. IV: tr 0.35, fe 1.15, pa 0.64, ti 0.96, mt 1.22, ta 0.98.

Dorsum (Fig. 86, 87). Dorsal scutum microgranulate, without clear delimitation of areas. Area I characterized by a row of three setae, while areas II–III display a row of six low, broad, arch-shaped setiferous tubercles. Area IV and posterior margin with a row of low, broad setiferous tubercles. Free tergites with a row of low, broad setiferous tubercles.

Chelicerae (Fig. 88A, B). Segment I with a small tubercle on the dorso-distal surface. Segment II with a mesal tubercle and bearing few setae.

Pedipalps (Fig. 88C, D). Trochanter with a small dorsal and ectal tubercle. Femora with two ventroproximal tubercles with subdistal setae, four distal setiferous granules, and three dorsoproximal tubercles with subdistal setae. Mesal surface of the femora with two rows of setiferous granules. Patella with ventral setiferous granules. Tibia with three ectal and two mesal spines with subdistal setae, the ventral surface adorned with small setiferous granules. Tarsus with three mesal and ectal spines with subdistal setae, with a few setae and granules.

Legs (Fig. 89). Coxae I–II with setiferous tubercles, with the longest distal tubercle bearing a subdistal seta. Coxae III and IV with microgranulation only, lacking setiferous tubercles. With four bridges between legs II and III, five or six bridges between III and IV, and four bridges between leg IV and the opisthosoma, the distal bridge longer than the others. Spiracles not obstructed by bridges. The smooth surface occupies ~ 1/3 of leg II, with two small tubercles and two rounded tubercles present. In leg III, the smooth area covers ~ ¾ of the surface, while in leg IV, it occupies < 1/3 of the surface. Sternum arrow-shaped. Legs smooth, with sparse setae. Tarsal count: 4–7–4–4.

Penis (Figs 90, 91). Pars distalis with large ventral plate bearing a cleft dividing the plate into two lamellae. Each lamella with three pointed macrosetae on the ventral surface and one macroseta on the dorsal surface. Capsula externa covering dorsal and lateral surfaces, with a long cleft dividing capsula externa into two parts; with a dorsolateral plate attached to the pars basalis. Capsula interna longer than capsula externa, partially covering the ventral plate, the apical region of the capsula interna thin and sharp, the stylus not visible in its apical portion.

**Female.** Similar to males, but with a noticeable shorter pedipalpal femora.

Female measurements. Total length 2.2, carapace length 1.00, dorsal scutum length 2.1, carapace max. width 1.3, mesotergum max. width 2.0. Appendage measurements: Pedipalps. Trochanter length 0.21, femora length 0.85, patella length 0.50, tibia length 0.50, tarsus length 0.63. Leg I: trochanter (tr) 0.22, femora (fe) 0.87, patella (pa) 0.47, tibia (ti) 0.66, metatarsus (mt) 0.76, tarsus (ta) 0.65. II: tr 0.25, fe 1.10, pa 0.60, ti 0.89, mt 0.99, ta 1.21. III: tr 0.25, fe 0.75, pa 0.36, ti 0.58, mt 0.61, ta 0.67, IV: tr 0.33, fe 1.05, pa 0.66, ti 0.89, mt 1.09, ta 0.78. Tarsal count 3–6–4–4.

##### ﻿ Nerudiella choapa sp. nov.

Taxon classificationAnimaliaOpilionesTriaenonychidae

C8C7BFC9-6D8A-5F7D-A758-9234D3632D32

https://zoobank.org/B4A37CD7-79B1-49D3-9B3C-8ACC7193480E

**Figure 92.**
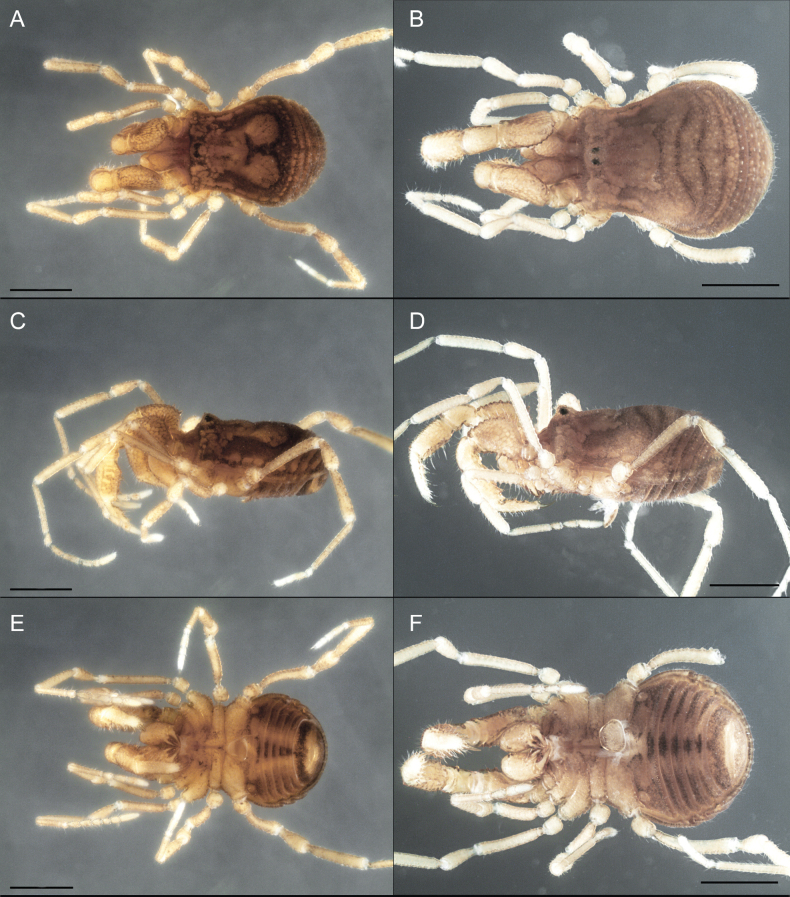
*Nerudiellachoapa* sp. nov. habitus, male **A** dorsal view **B** lateral view **C** ventral view. Scale bars: 1 mm.

**Figure 93.**
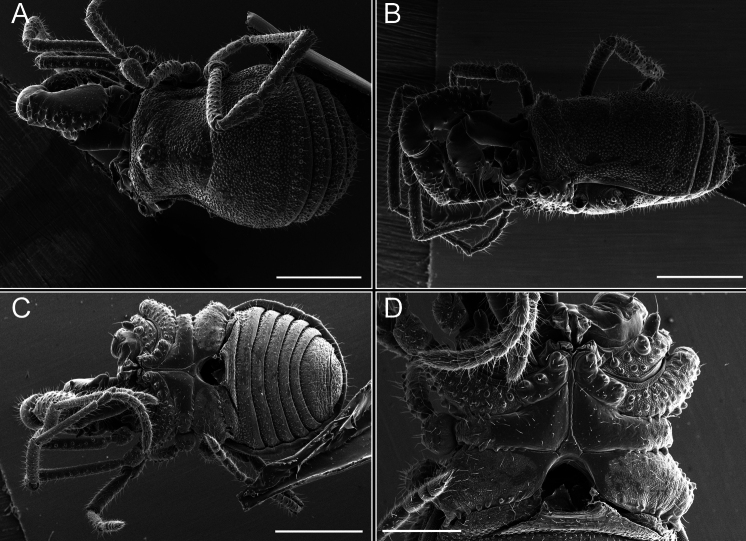
*Nerudiellachoapa* sp. nov. male, SEM images of habitus **A** dorsal view **B** lateral view **C, D** ventral view. Scale bars: 1 mm (**A, B, C**); 500 µm (**D**).

**Figure 94.**
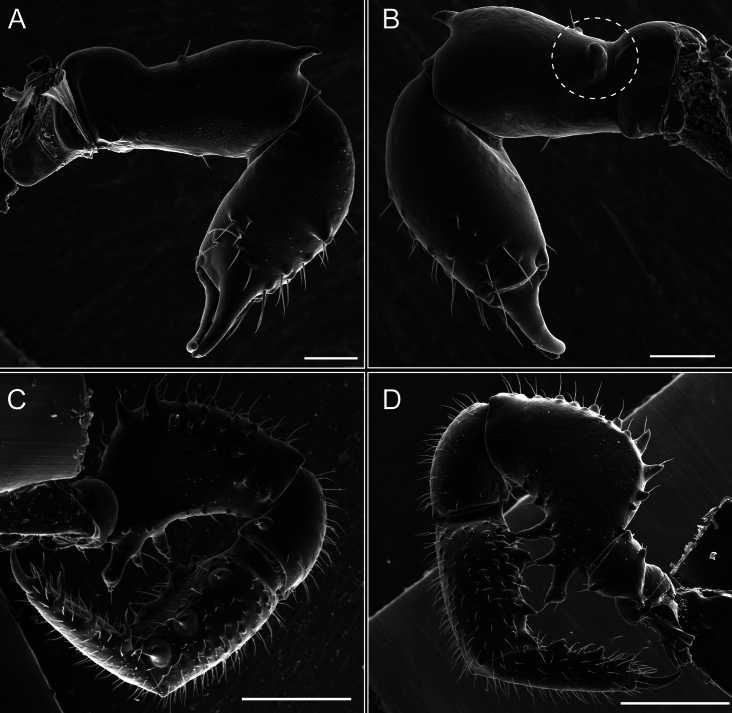
*Nerudiellachoapa* sp. nov. chelicerae: mesal **A** ectal **B** pedipalps: mesal **C** ectal **D**. Scale bars: 200 µm (**A, B**); 500 µm (**C, D**).

**Figure 95.**
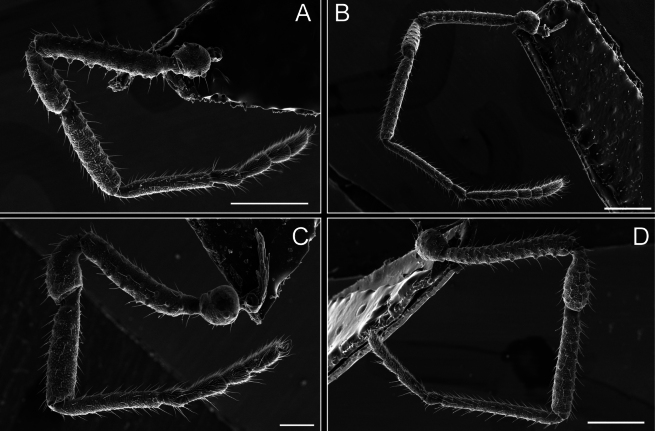
*Nerudiellachoapa* sp. nov. legs I **A** II **B** III **C** IV **D**. Scale bars: 500 µm (**A, B, D**); 200 µm (**C**).

**Figure 96.**
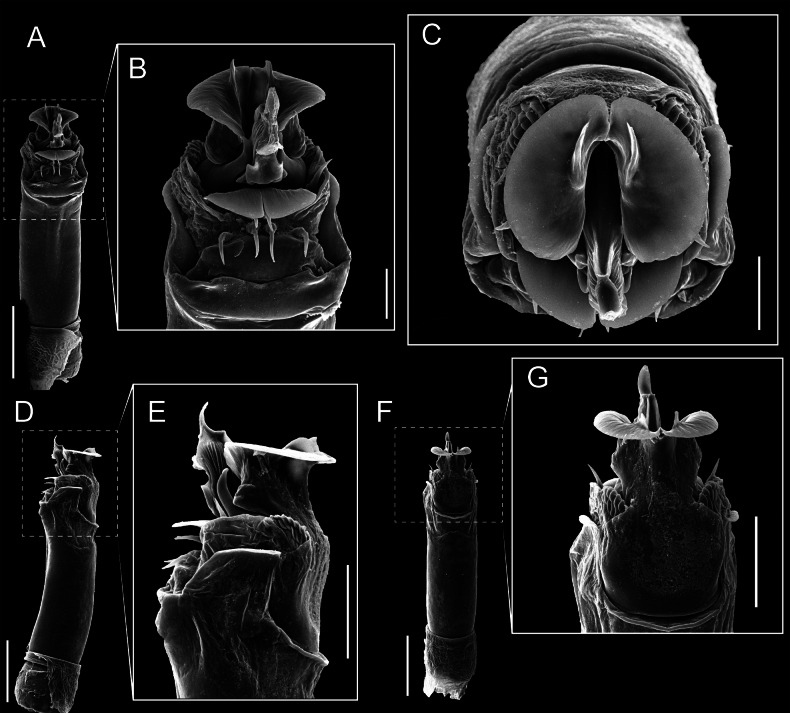
*Nerudiellachoapa* sp. nov. penis: ventral **A, B** apical **C** lateral **D, E** dorsal **F, G**. Scale bars: 200 µm (**A, D, F**); 50 µm (**B, C**); 100 µm (**E, G**).

**Figure 97.**
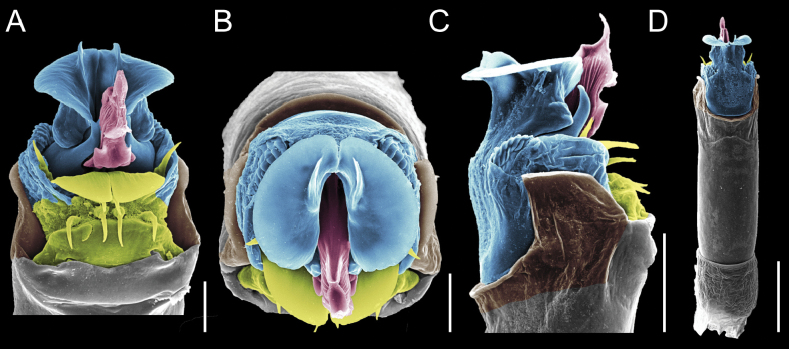
*Nerudiellachoapa* sp. nov. penis: ventral **A** apical **B** lateral **C** dorsal **D**. Colors: ventral plate (yellow), capsula externa (blue), capsula interna (red). Scale bars: 50 µm (**A, B**); 100 µm (**C**); 200 µm (**D**).

Figs 92 , 93 , 94 , 95 , 96 , 97

###### Material examined.

***Holotype*.** ♂ **Chile.** Valparaíso: Pichicuy, Quebrada Huaquén, E. Maury coll., 25.X.1988 (MNHNCL). ***Paratypes*. Chile.** Coquimbo (Choapa province): Pichidangui, Cerro La Silla del Gobernador, E. Maury coll., 31.X.1988, 3 ♂ (MACN). Same data 6 ♀ 2 imm. (MACN).

###### Etymology.

The specific epithet “choapa” is derived from one of the locality where the species was collected, the Choapa province in the Coquimbo region of Chile. It is used as noun in apposition to indicate the association of the species with this specific geographic location.

###### Diagnosis.

This species can be distinguished from its congeners by several key characteristics. Firstly, its dorsal surface and pedipalps are densely setose. Additionally, the femora and tibia of the pedipalp are covered with small tubercles. The unique male genitalia has a capsula externa that covers the dorsal and lateral surfaces. The apical region of the capsula externa is bent at a 90-degree angle in relation to the axis of the genitalia, and there are two small parallel apical structures present. This species shares similarities with *Nerudiellazapallar* sp. nov., particularly in the apical region of the capsula externa, although it is relatively shorter in comparison in the latter species.

###### Distribution.

Chile: Coquimbo and Valparaíso Regions (Fig. 4F).

###### Description of male holotype.

Measurements: Total length 2.19, carapace length 0.94, dorsal scutum length 1.77, carapace max. width 1.33, dorsal scutum max. width 1.77. Appendage measurements: Pedipalps. Trochanter length 0.21, femora length 0.90, patella length 0.49, tibia length 0.70, tarsus length 0.86. Leg I: trochanter (tr) 0.21, femora (fe) 0.86, patella (pa) 0.45, tibia (ti) 0.67, metatarsus (mt) 0.82, tarsus (ta) 0.61. II: tr 0.20, fe 1.21, pa 0.53, ti 0.97, mt 1.07, ta 1.23. III: tr 0.24, fe 0.83, pa 0.39, ti 0.68, mt 0.77, tr 0.64. IV: tr 0.24, fe 1.19, pa 0.57, ti 0.96, mt 1.13, ta 0.75.

Dorsum (Fig. 92, 93). Eta (η) hourglass-shaped dorsal scutum. Ocularium low and rounded, with small tubercles. Dorsal scutum and free tergites with microgranulation. Mesotergal areas lack clear separation but are covered with small setiferous tubercles.

Chelicerae (Fig. 94A, B). Segment I with a sharp tubercle on the dorso-distal surface, with two proximal tubercles. In segment II, there are scattered setae visible in both the ectal and ventral views. In the front view, there is a prominent triangular tubercle that stands out from the others.

Pedipalps (Fig. 94C, D). Trochanter with two small dorsal tubercles and one ventral tubercle. Femora with a dorso-mesal area with setiferous tubercles, including two stronger ones in the proximal region. In the ventral view, there are three prominent proximal spines and a row of small tubercles. Patella with a mesal setiferous tubercle. Tibia with three ventral-ectal spines and two ventral-mesal spines, with the lateral and dorsal areas with small setiferous tubercles. Tarsus with three mesal and ectal spines with subdistal setae, as well as additional setae and a few setae.

Legs (Fig. 95). Coxa I with 11–13 setiferous tubercles, the two apical ones being stronger and more prominent than the others. Coxa II with 18–20 setiferous tubercles, while coxa IV has 5–6 small tubercles. Bridges between legs do not obstruct the spiracles. The smooth surface occupies ~ 1/3 of leg II, ¾ of leg III, and < 1/3 of leg IV. Within smooth area of leg II, there are two small tubercles with subdistal setae on each side. Sternum arrow-shaped. Legs covered in small tubercles, and the astragalus is longer than the calcaneus on all legs (Fig. 95). Tarsal count: 3–5–4–4.

Penis (Figs 96, 97). Genitalia: Pars distalis with a ventral plate bearing a cleft that divides the plate into two lamellae. Each lamella has three pointed macrosetae on the ventral surface and one macroseta on the dorsal surface; capsula externa covering the dorsal and lateral surface, having the apical region bent at an angle of 90 ° in relation to the axis of the genitalia. It includes a pair of small apical processes in the form of small “wings”. The capsula interna also has a pair of lateral processes, which are long and ventrally sloping. Capsula interna longer than capsula externa, with its apical portion thinner.

**Female.** Similar to males, with shorter pedipalpal femora.

Female measurements. Total length 2.63, carapace length 1.0, dorsal scutum length 2.11, carapace max. width 1.47, mesotergum max. width 2.07. Appendage measurements: Pedipalps. Trochanter length 0.27, femora length 0.84, patella length 0.52, tibia length 0.61, tarsus length 0.92. Leg I: trochanter (tr) 0.28, femora (fe) 0.88, patella (pa) 0.48, tibia (ti) 0.68, metatarsus (mt) 0.84, tarsus (ta) 0.63. II: tr 0.29, fe 1.16, pa 0.56, ti 0.89, mt 1.05, ta 1.20. III: tr 0.28, fe 0.81, pa 0.38, ti 0.64, mt 0.84, ta 0.67, IV: tr 0.29, fe 1.18, pa 0.61, ti 0.98, mt 1.25, ta 0.75. Tarsal count 3–6–4–4.

##### ﻿ Nerudiella curi sp. nov.

Taxon classificationAnimaliaOpilionesTriaenonychidae

EAF1DB24-3B20-535D-8E45-803F91FCD9F3

https://zoobank.org/9F17E25B-8E00-4677-B1C3-9FD8DDAB3731

**Figure 98.**
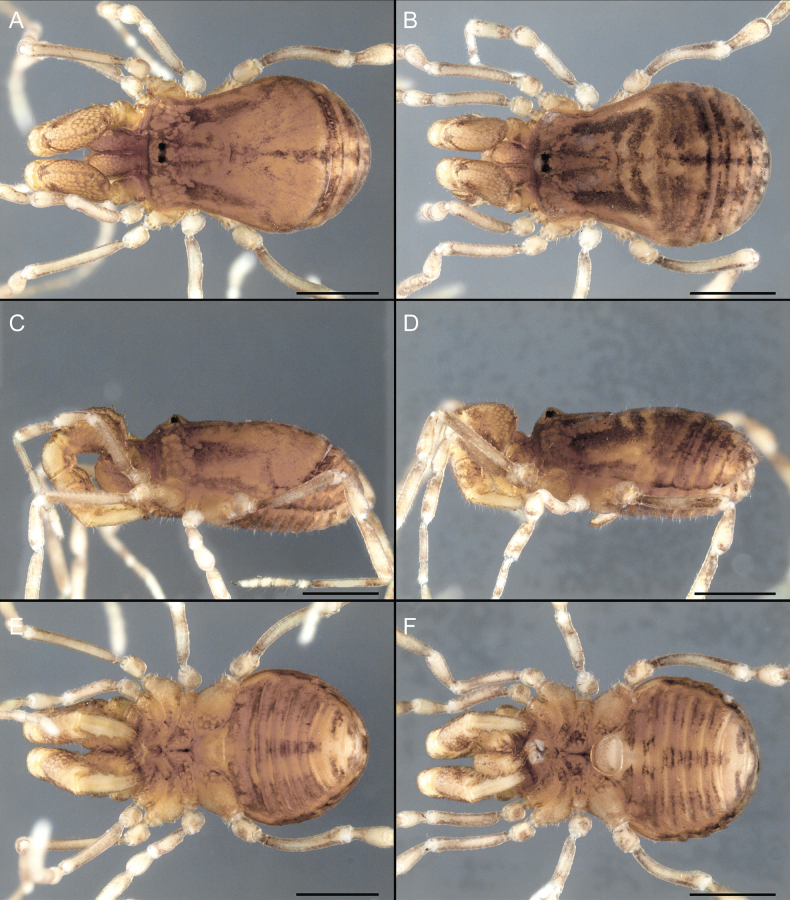
*Nerudiellacuri* sp. nov. habitus, male **A** dorsal view **C** lateral view **E** ventral view. Female **B** dorsal view **D** lateral view **F** ventral view. Scale bars: 1 mm.

**Figure 99.**
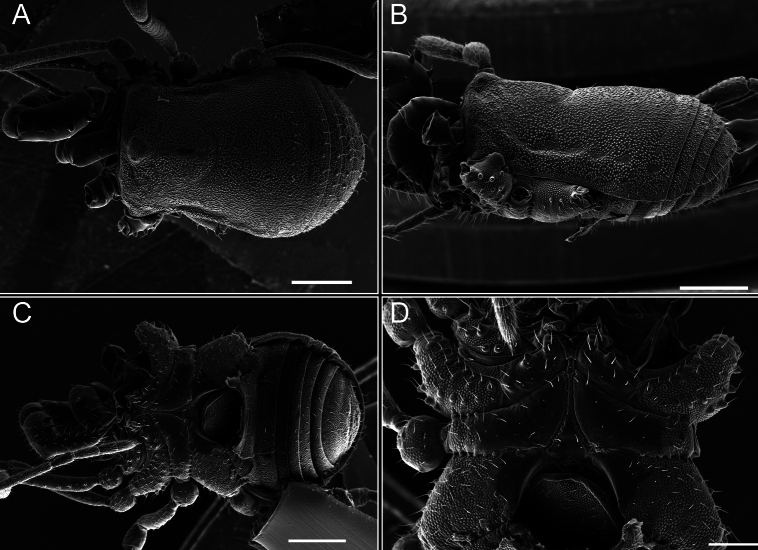
*Nerudiellacuri* sp. nov. male, SEM images of habitus **A** dorsal view **B** lateral view **C, D** ventral view. Scale bars: 500 µm (**A, B, C**); 200 µm (**D**).

**Figure 100.**
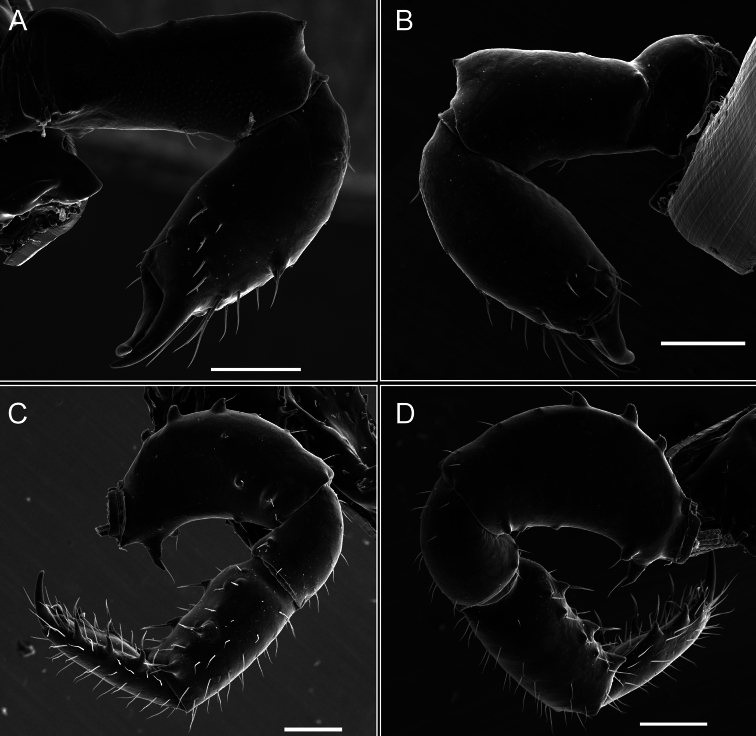
*Nerudiellacuri* sp. nov. chelicerae: mesal **A** ectal **B** pedipalps: mesal **C** ectal **D**. Scale bars: 200 µm.

**Figure 101.**
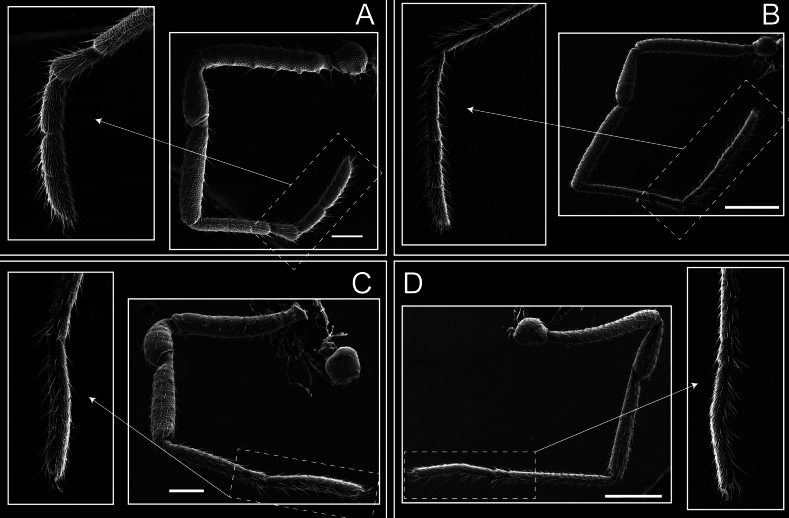
*Nerudiellacuri* sp. nov. legs I **A** II **B** III **C** IV **D**. Scale bars: 200 µm (**A, C**); 500 µm (**B, D**).

**Figure 102.**
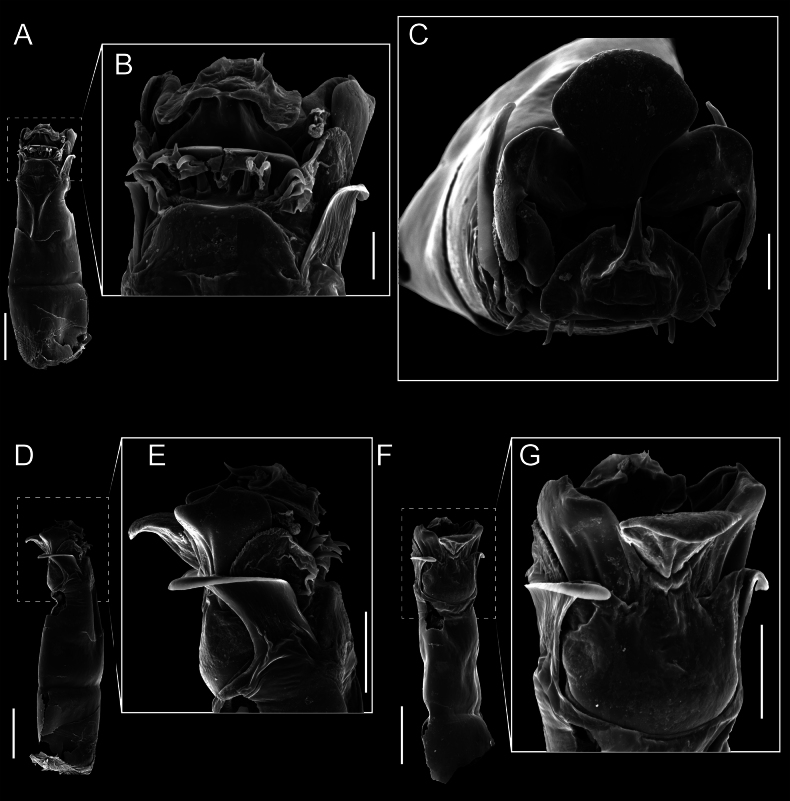
*Nerudiellacuri* sp. nov. penis: ventral **A, B** apical **C** lateral **D, E** dorsal **F, G**. Scale bars: 200 µm (**A, D, F**); 50 µm (**B, C**); 100 µm (**E, G**).

**Figure 103.**
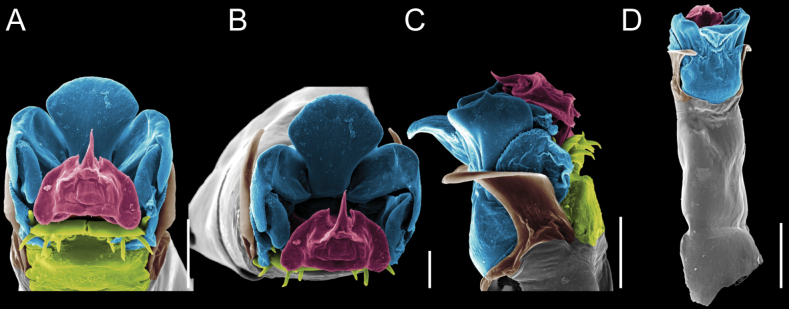
*Nerudiellacuri* sp. nov. penis: ventral **A** apical **B** lateral **C** dorsal **D**. Colors: ventral plate (yellow), capsula externa (blue), capsula interna (red). Scale bars: 50 µm (**A, B**); 100 µm (**C**); 200 µm (**D**).

Figs 98 , 99 , 100 , 101 , 102 , 103

###### Material examined.

***Holotype*.** ♂ **Chile.** Curicó: Cerro Hueca-Hueca, J. Barriga coll., 10.12.2005 (MNHNCL). ***Paratypes*. Chile.** Curicó: Cerro Hueca-Hueca, J. Barriga coll., 10.12.2005, 3 ♂ 5 ♀ (MACN).

###### Etymology.

The specific epithet is a variation of the spelling of “kuri” or “kurü,” which is a word in the Mapuche language used to define the color black, which is present in spots on the dorsal surface of individuals of the group. Noun in apposition.

###### Diagnosis.

This species can be readily distinguished from other species in the genus by several characteristics. Firstly, it exhibits a wider genital operculum compared to other species. Additionally, the male genitalia display a distinct morphology, featuring a capsula externa consisting of three lamellae. Furthermore, the ventral plate of the male genitalia is characterized by a row of aligned macrosetae.

###### Distribution.

Chile: Maule Region, Curicó Province (Fig. 4F).

###### Description of male holotype.

Measurements: Total length 2.59, carapace length 0.91, dorsal scutum length 2.03, carapace max. width 1.30, max. mesotergum width 1.77. Appendage measurements. Pedipalps. Trochanter length 0.22, femora length 0.69, patella length 0.37, tibia length 0.52, tarsus length 0.49. Leg I: trochanter (tr) 0.24, femora (fe) 0.89, patella (pa) 0.43, tibia (ti) 0.70, metatarsus (mt) 0.73, tarsus (ta) 0.70. II: tr 0.29, fe 1.11, pa 0.50, ti 0.96, mt 1.05, ta 1.20. III: tr 0.27, fe 0.75, pa 0.36, ti 0.66, mt 0.78, ta 0.63. IV: tr 0.32, fe 1.19, pa 0.63, ti 0.98, mt 1.26, ta 0.77.

Dorsum (Fig. 98, 99). Eta (η) hourglass-shaped dorsal scutum. Ocularium low on the body. Dorsal scutum microgranulate, without clear delimitation of distinct areas. In the anterior region, specifically areas I and II, there are two small setiferous tubercles. In the area III, there is a row of four small setiferous tubercles, while area IV is adorned with a row of eight small setiferous tubercles. Posterior margin of dorsal scutum with a row of eight small setiferous tubercles. Free tergites with a row of small setiferous tubercles.

Chelicerae (Fig. 100A, B). Segment I with a small tubercle on the dorso-distal surface. Segment II with a small frontal tubercle and scattered setae.

Pedipalps (Fig. 100C, D). Trochanter with two small dorsal tubercles and one ventral. Femora with a row of three tubercles with subdistal setae on dorsal view, one long and one small spines with subdistal setae on ventral view, six low setiferous tubercles in mesal view, and one in ectal view. Patella with a small mesal and ectal setiferous tubercle. Tibia with a mesal row of five small ectal setiferous tubercles, three ventral ectal spines, and two ventral mesal spines with subdistal setae. Tarsus with three mesal and ectal spines with subdistal setae.

Legs (Fig. 101). Coxa I with two rows of setiferous tubercles and two distal setiferous tubercles with subdistal setae, coxa II with two rows of setiferous tubercles, III and IV only with microgranulation, three bridges between coxa legs II and III, six between coxa legs III and IV, four between leg IV and opisthosoma. Spiracles not obstructed by bridges. Smooth surface that occupies 1/3 of leg II, ¾ of III and 1/3 of IV. Genital operculum larger than other species of the genus. Smooth area of leg II with a setiferous tubercle on each side. Sternum arrow-shaped. Legs I–IV covered in setae, tarsal area, and calcaneus densely setose. Calcaneus smaller than astragalus, ≥ 3× smaller (legs I–III) and 4× (leg IV). Tarsal count: 4–6–4–4.

Penis (Figs 102, 103). Pars distalis with a ventral plate bearing a cleft dividing the plate into two lamellae. Each lamella has a row of three pointed macrosetae on the ventral surface and one macroseta on the dorsal surface. Capsula externa covering the dorsal and lateral surface, divided into three folds, one pair covering the dorsolateral surface and the last one covering the dorsal surface, there is a dorsolateral plate attached to the pars basalis. Capsula interna divided into three parts, two lateral processes and a central process in which it bears an apical stylus. The ventral plate bears a cleft that divides the plate into two halves, each with three ventral and one dorsal macrosetae. Ventral macrosetae arranged in a row.

**Female.** Similar to males, with shorter pedipalpal femora.

Female measurements. Total length 2.40, carapace length 0.85, dorsal scutum length 1.89, carapace max. width 1.23, mesotergum max. width 1.67. Appendage measurements: Pedipalps. Trochanter length 0.22, femora length 0.69, patella length 0.37, tibia length 0.52, tarsus length 0.49. Leg I: trochanter (tr) 0.14, femora (fe) 0.24, patella (pa) 0.89, tibia (ti) 0.43, metatarsus (mt) 0.70, tarsus (ta) 0.73. II: tr 0.70, fe 0.29, pa 1.11, ti 0.50, mt 0.96, ta 1.05. III: tr 1.20, fe 0.27, pa 0.75, ti 0.36, mt 0.66, ta 0.78. IV: tr 0.63, fe 0.32, pa 1.19, ti 0.63, mt 0.98, ta 1.26, tr 0.77.

##### ﻿ Nerudiella goroi sp. nov.

Taxon classificationAnimaliaOpilionesTriaenonychidae

9525F6CA-3D9D-51DA-91B1-D870BF986208

https://zoobank.org/83521462-412E-44DC-8DEB-07C518E25495

**Figure 104.**
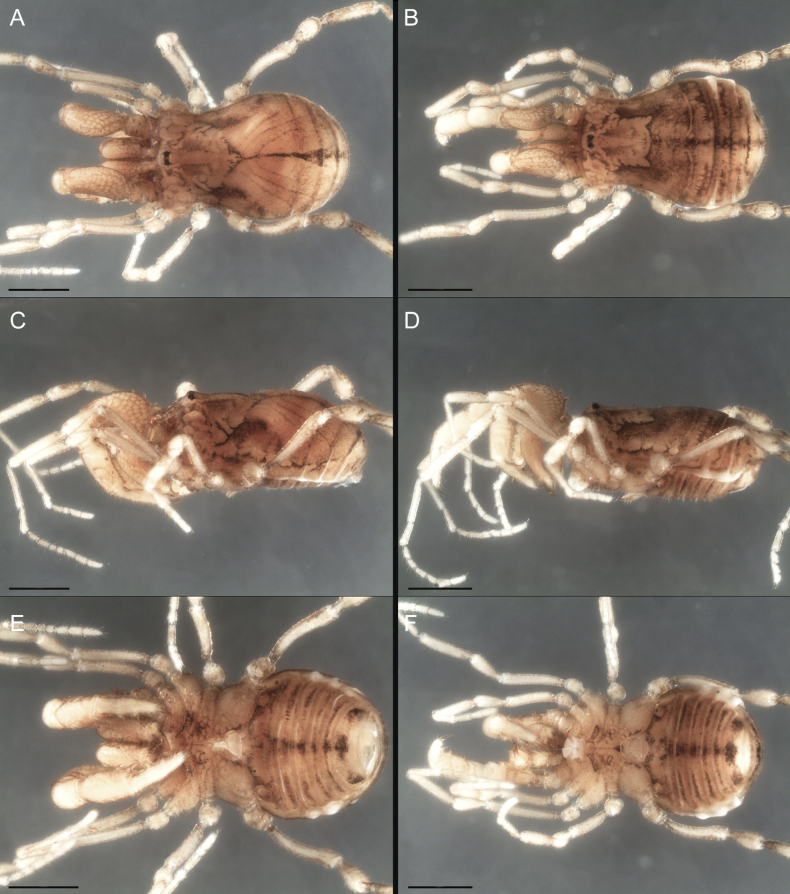
*Nerudiellagoroi* sp. nov. habitus, male **A** dorsal view **C** lateral view **E** ventral view. Female **B** dorsal view **D** lateral view **F** ventral view. Scale bars: 1 mm.

**Figure 105.**
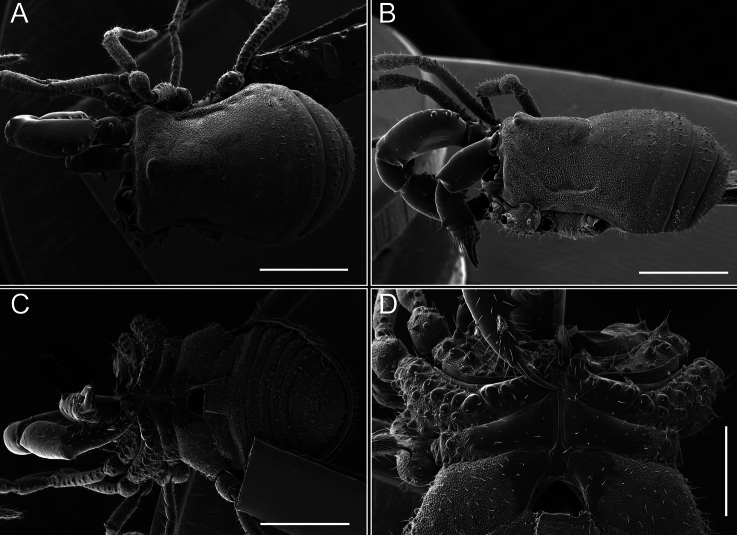
*Nerudiellagoroi* sp. nov. male, SEM images of habitus **A** dorsal view **B** lateral view **C, D** ventral view. Scale bars: 1 mm (**A, B, C**); 500 µm (**D**).

**Figure 106.**
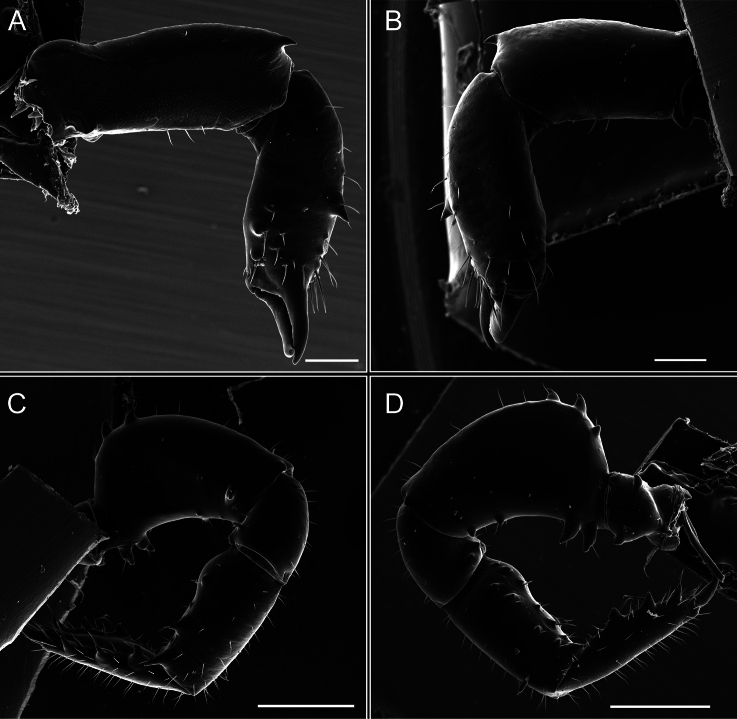
*Nerudiellagoroi* sp. nov. chelicerae: mesal **A** ectal **B** pedipalps: mesal **C** ectal **D**. Scale bars: 200 µm (**A, B**); 500 µm (**C, D**).

**Figure 107.**
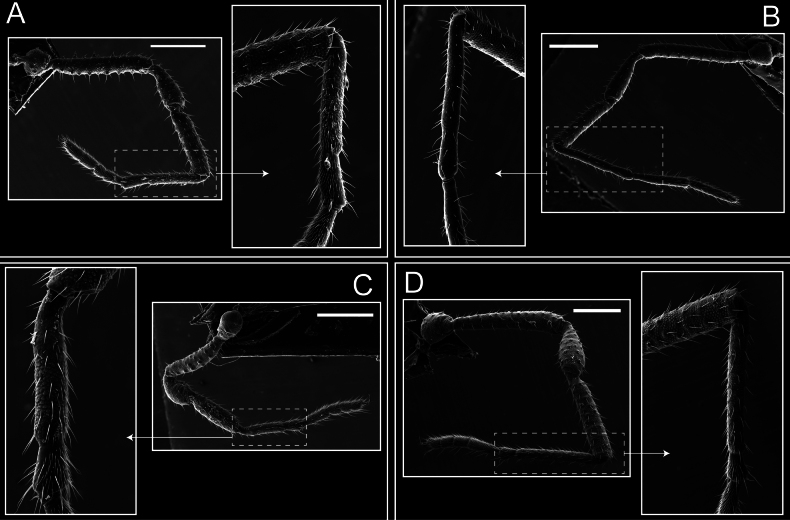
*Nerudiellagoroi* sp. nov. legs I **A** II **B** III **C** IV **D**. Scale bars: 500 µm.

**Figure 108.**
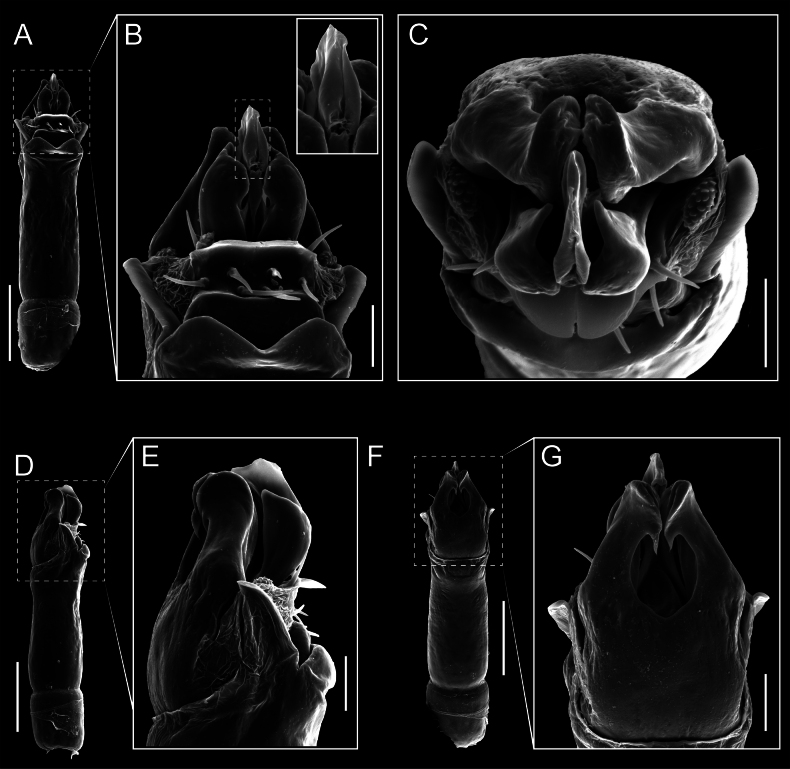
*Nerudiellagoroi* sp. nov. penis: ventral **A, B** apical **C** lateral **D, E** dorsal **F, G**. Scale bars: 200 µm (**A, D, F**); 50 µm (**B, C, E, G**).

**Figure 109.**
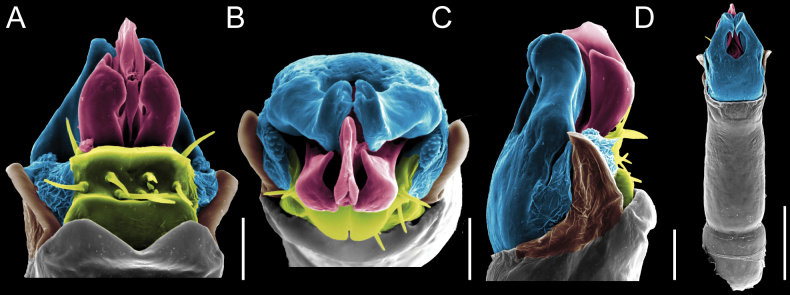
*Nerudiellagoroi* sp. nov. penis: ventral **A** apical **B** lateral **C** dorsal **D**. Colors: ventral plate (yellow), capsula externa (blue), capsula interna (red). Scale bars: 50 µm.

Figs 104 , 105 , 106 , 107 , 108 , 109

###### Material examined.

***Holotype*.** ♂ **Chile.** Concepción: Estero Nonguén, T.Cekalovic coll., 21.IV.1976 (MNHNCL). ***Paratypes*. Chile.** Concepción: 6 km S San Pedro, 360 m, A. Newton, M. Thayer coll., 12.XII.1982, 1 ♂ (AMNH). Camino a Ramuncho, Cruce Hualpén, T.Cekalovic coll., 02.XI.1986, 4 ♂ 1 ♀ 18 imm. (MACN). Cautín: Ojos del Caburgua, 16 km NE de Pucón, E. Maury coll., 16.I.1987, 1 ♂ 7 ♀ 6 imm. (MACN). Bellavista, Lago Villarrica, 260 m, R.Schuh, N. Platnick coll., 30.I.1986, 1 ♂ 4 ♀ 7 imm. (AMNH). Cautín: Cerro Ñielol Temuco, IX Región (Araucaria), E. Maury coll., 21.I.1991, 1 ♂ 1 imm. (MACN). Cautín: Cerro Ñielol Temuco, IX Región (Araucaria), E. Maury coll., 15.I.1989, 3 ♀ 2 imm. (MACN). Termas de Palguin SE de Pucón, E. Maury coll., 17.01.1987, 1 ♀ 1 imm. (MACN). Estero Chauilco, T.Cekalovic coll., 25.I.1980, 1 ♀ (MACN)

###### Additional material.

Chile. Cautín: Bellavista, Fundo Flor del Lago, 40.6663°S, 72.1733°W, 270 m, M. Ramírez, F. Labarque coll., 09.02.2005, 3 ♂ 5 ♀ 12 imm. (MACN). Same data, 2 ♀ 31 imm. (MACN). Same data 3 ♂ 1 ♀ 2 imm. (MACN). Cautín: Volcán, Villarrica, 1100 m, N. Platnick, O. Francke coll., 28.I.1985, 2 imm. (AMNH). Bellavista, Lago Villarrica, N. Platnick, O. Francke coll., 28.I.1985, 2 ♂ 2 ♀ 4 imm. (AMNH). Bellavista, Lago Villarrica, 260 m, R.Schuh, N. Platnick coll., 30.01.1986, 2 imm. (AMNH). Same locality N. Platnick, K.Catley, M. Ramírez, T.Allen coll., 20.XI.1993, 1 ♂ 1 ♀ 1 imm. (AMNH). Osorno: Pucatrihue, L.Peña coll., 10.II.1985, 2 imm. (FMNH).

###### Etymology.

The specific epithet is bestowed upon the Chilean entomologist Raúl Briones Parra, widely recognized by his nickname “Goro”, in recognition to his significant contributions to the field of entomology and his unwavering dedication to the conservation of the Chilean forests. A noun in apposition.

###### Diagnosis.

This species can be easily distinguished from the other species in the genus by having two sharp tubercles on the ventral-proximal region of the pedipalp femora. The male genitalia has a pair of ventrally curved capsula externa processes.

###### Distribution.

Chile: Regions of Bío-Bío, Araucanía, Los Ríos, Los Lagos.

###### Description of male holotype.

Total length 3.9. Carapace length 1.1, dorsal scutum length 2.5, carapace max. width 1.4, mesotergum max. width 1.6. Appendage measurements. Pedipalps. Trochanter length 0.25, femora length 0.94, patella length 0.60, tibia length 0.70, tarsus length 0.68. Leg I: trochanter (tr) 0.27, femora (fe) 1.37, patella (pa) 0.59, tibia (ti) 0.90, metatarsus (mt) 1.13, tarsus (ta) 0.83. II: tr 0.28, fe 1.75, pa 0.79, ti 1.30, mt 1.50, ta 1.73. III: tr 0.32, fe 1.12, pa 0.50, ti 0.71, mt 0.99, ta 0.86. IV: tr 0.41, fe 1.53, pa 0.84, ti 1.26, mt 1.69, ta 1.04.

Dorsum (Fig. 104, 105). Eta (η) hourglass-shaped dorsal scutum. Ocularium low and rounded. Dorsal scutum microgranulate, with mesotergal areas that lack distinct demarcation but with small setiferous tubercles. Additionally, the mesotergal areas, posterior edge, and free tergites feature a row of small setiferous tubercles.

Chelicerae (Fig. 106A, B). Segment I with an acute tubercle on the dorso-distal surface. Segment II bearing scattered setae in ectal and ventral views, with one triangular tubercle prominent from the others in front view.

Pedipalps (Fig. 106C, D). Trochanter with a small dorsal and ventral tubercle. Femora bearing three dorsoproximal setiferous tubercles; 4–6 small ventral-distal tubercles and two ventral-proximal spines. Patella with 2–3 small ventral tubercles. Tibia possesses two prominent ventral-mesal spines, in addition to small sparse setiferous tubercles. Tarsus with three mesal and ectal spines in addition to setae and sparse setae.

Legs (Fig. 107). Coxa I with 10 setiferous tubercles, II with 18–20 setiferous tubercles, III with three. Spiracles not obstructed by bridges. Smooth surface occupying 1/3 of leg II, ¾ of III and < 1/3 of IV. Smooth area of leg II with 3 tubercles with subdistal setae on each side. Sternum arrow-shaped. Legs covered in small tubercles, astragalus longer than calcaneus on all legs. Tarsal count: 4–8–4–4.

Penis (Figs 108, 109). Pars distalis characterized by a ventral plate divided into two halves by a small cleft. Each half of the plate bears three pointed macrosetae on the ventral surface and one small macroseta on the dorsal surface. The dorsal surface is covered by a capsula externa, with the upper portion of the plate featuring a wide notch that forms a pair of sharp pointed processes at the base of the genitalia. The capsula externa also exhibits a pair of broad lateral processes. It is slightly lower than the capsula interna, which is laterally flattened and compressed between the lateral processes of the capsula externa. The capsula interna does not have a visible stylus. Additionally, there is a dorsolateral plate connected to the pars basalis.

**Female.** Similar to males, with a shorter pedipalpal femora.

Female measurements. Measurements: Total length 2.50, carapace length 1.1, dorsal scutum length 2.17, carapace max. width 1.55, mesotergum max. width. Appendage measurements: Pedipalps. Trochanter length 0.26, femora length 0.90, patella length 0.55, tibia length 0.57, tarsus length 0.67. Leg I: trochanter (tr) 0.25, femora (fe) 0.96, patella (pa) 0.53, tibia (ti) 0.71, metatarsus (mt) 0.84, tarsus (ta) 0.64. II: tr 0.24, fe 1.27, pa 0.65, ti 0.98, mt 1.12, ta 1.27. III: tr 0.27, fe 0.82, pa 0.41, ti 0.61, mt 0.71, ta 0.69. IV: tr 0.31, fe 1.19, pa 0.74, ti 1.03, mt 1.26, ta 0.79. Tarsal count: 4–8–4–4.

##### ﻿ Nerudiella jaimei sp. nov.

Taxon classificationAnimaliaOpilionesTriaenonychidae

0FD906E4-908E-5BC4-A17E-760F8E39D414

https://zoobank.org/4986C4AB-EB4A-4F3E-86F6-063A7C4604B7

**Figure 110.**
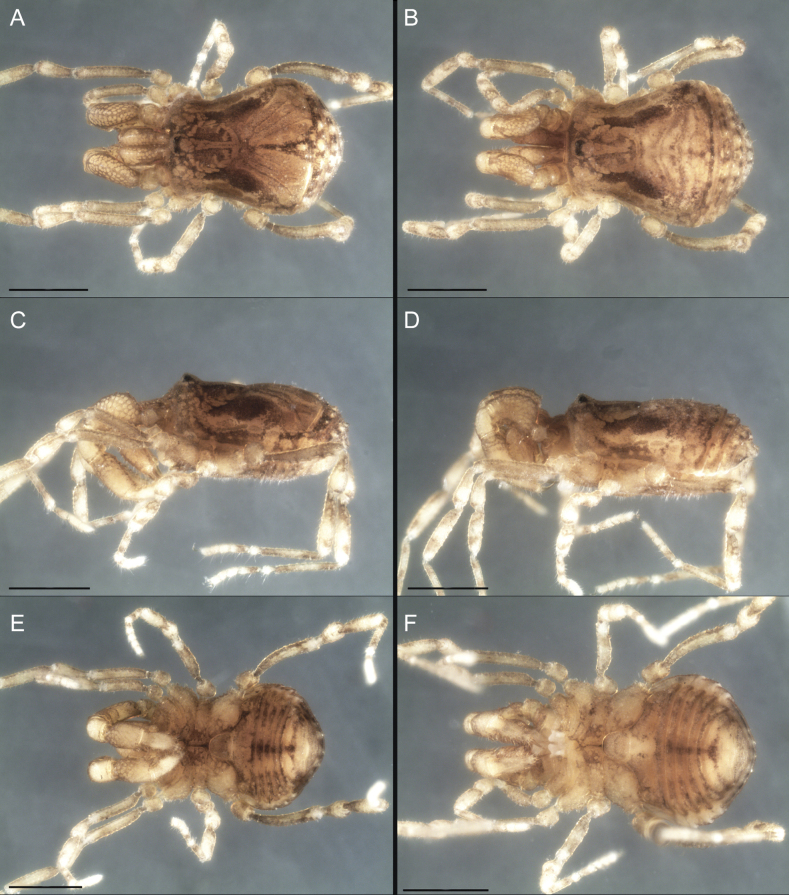
*Nerudiellajaimei* sp. nov. habitus, male **A** dorsal view **C** lateral view **E** ventral view. Female **B** dorsal view **D** lateral view **F** ventral view. Scale bars: 1 mm.

**Figure 111.**
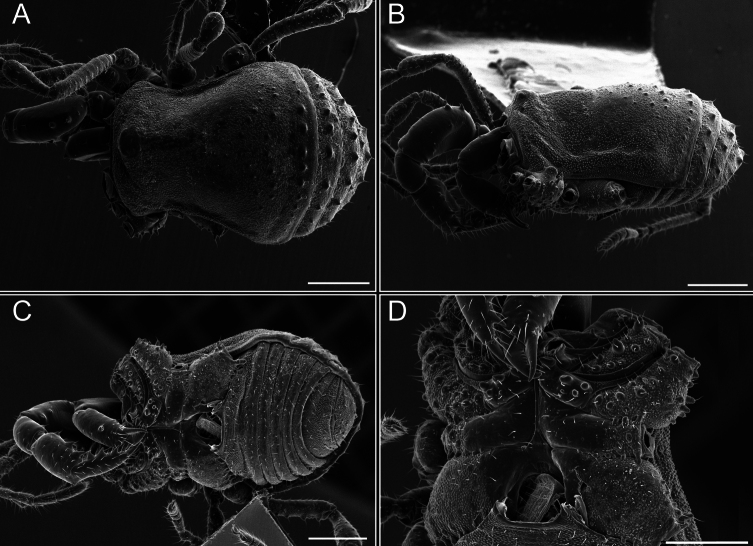
*Nerudiellajaimei* sp. nov. male, SEM images of habitus **A** dorsal view **B** lateral view **C, D** ventral view. Scale bars: 500 µm (**A, B, C**); 200 µm (**D**).

**Figure 112.**
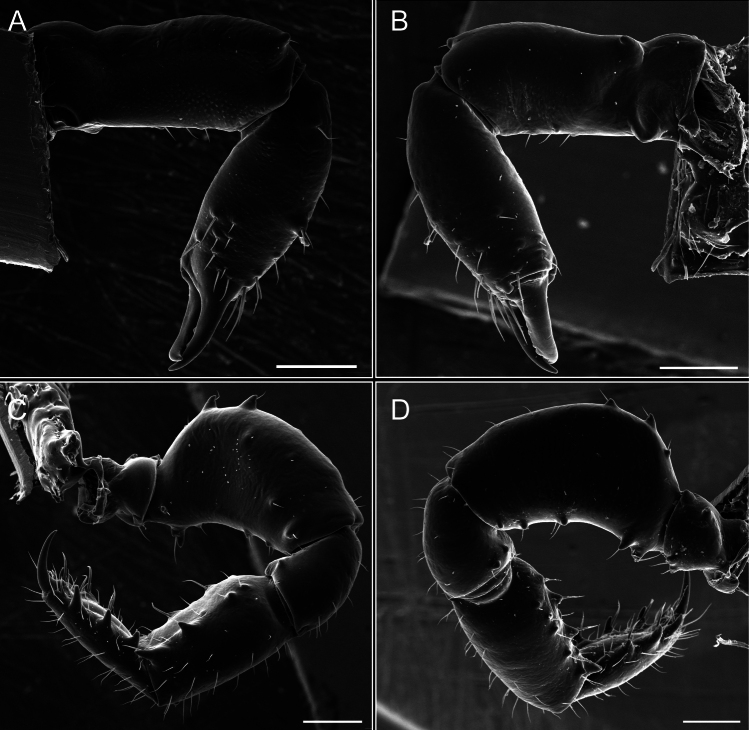
*Nerudiellajaimei* sp. nov. chelicerae: mesal **A** ectal **B** pedipalps: mesal **C** ectal **D**. Scale bars: 200 µm.

**Figure 113.**
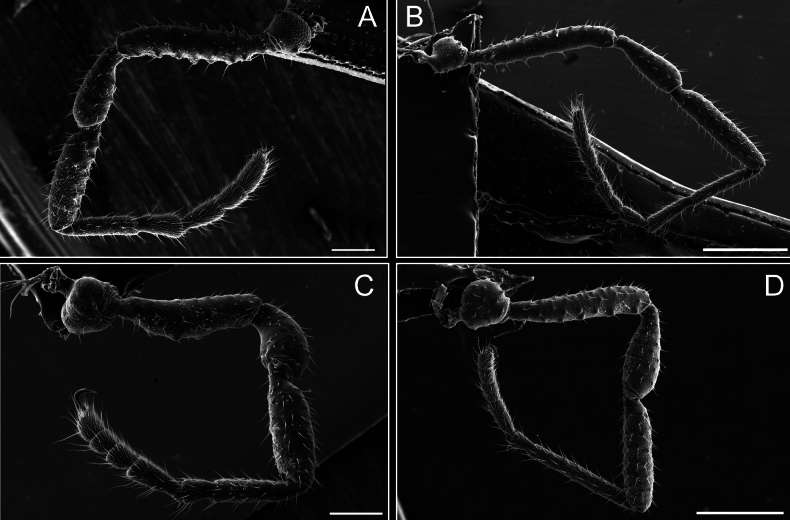
*Nerudiellajaimei* sp. nov. legs I **A** II **B** III **C** IV **D**. Scale bars: 200 µm (**A, C**); 500 µm (**B, D**).

**Figure 114.**
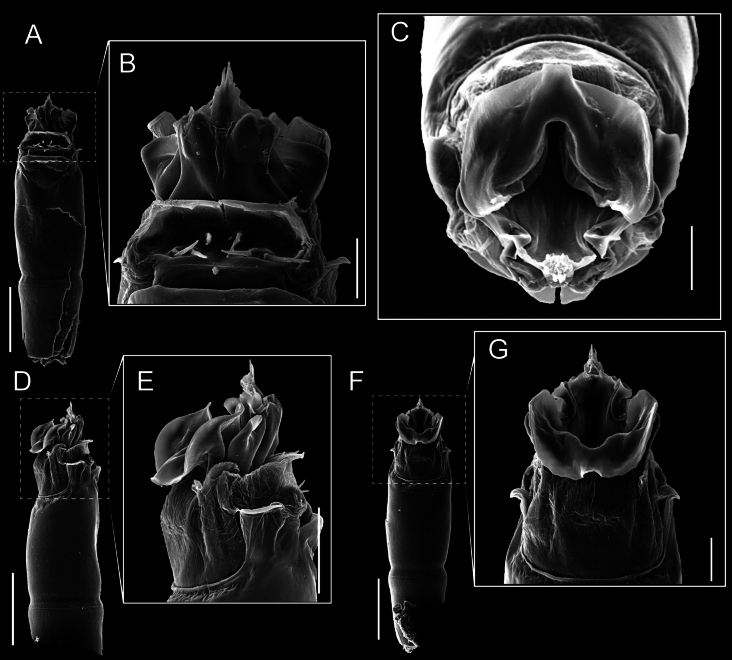
*Nerudiellajaimei* sp. nov. penis: ventral **A, B** apical **C** lateral **D, E** dorsal **F, G**. Scale bars: 200 µm (**A, D, F**); 50 µm (**B, C, G**); 100 µm (**E**).

**Figure 115.**
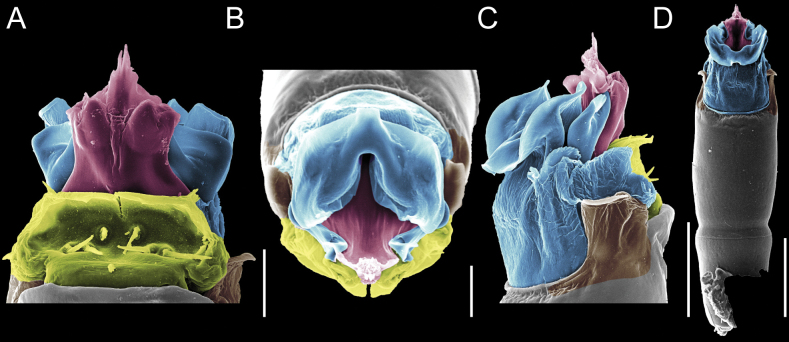
*Nerudiellajaimei* sp. nov. penis: ventral **A** apical **B** lateral **C** dorsal **D**. Colors: ventral plate (yellow), capsula externa (blue), capsula interna (red). Scale bars: 50 µm (**A, B**); 100 µm (**C**); 200 µm (**D**).

Figs 110 , 111 , 112 , 113 , 114 , 115

###### Material examined.

***Holotype*.** ♂ **Chile.** Malleco. Monumento Natural Contulmo, 350 m, S. Peck, J. Peck coll. (FMNH). ***Paratypes*. Chile.** Malleco. P.N. Nahuelbuta 1250 m, Berlese, N. Platnick, R. Schuh coll.19.XI.1981. 1 ♂ 2 imm. (AMNH). Same data, 4 ♂ 4 imm. (AMNH). Malleco. Monumento Natural Contulmo, 300 m, N. Platnick, R. Schuh coll.31.1.1986 2 ♀ 4 imm. (AMNH). Malleco. Monumento Natural Contulmo, 350 m, S. Peck, J. Peck coll. 13.II.1985. 5 ♂ 6 ♀ 13 imm. (FMNH). 45 KM W Angol, Nahuelbuta N.P. 1500 m, S. Peck, J. Peck coll. 09.XII.1984. 11 ♂ 6 ♀ 13 imm. (FMNH).

###### Etymology.

The specific epithet is bestowed upon the Chilean entomologist Jaime Pizarro Araya, in recognition to his significant contributions to the field of entomology and his unwavering dedication to the conservation of the Chilean forests. A noun in apposition.

###### Diagnosis.

This species can be distinguished from the other species of the genus by the morphology of the male genitalia, which includes the capsula externa with a robust process, which forms a dorsally curved plate.

###### Distribution.

Chile: Araucanía Region (Fig. 4F).

###### Description of male holotype.

Measurements: Total length 4.0, carapace length 1.0, dorsal scutum length 2.5, carapace max. width 1.2, mesotergum max. width 1.7. Appendage measurements: Pedipalps. Trochanter length 0.26, femora length 0.96, patella length 0.59, tibia length 0.74, tarsus length 0.69. Leg I: trochanter (tr) 0.19, femora (fe) 0.70, patella (pa) 0.40, tibia (ti) 0.52, metatarsus (mt) 0.63, tarsus (ta) 0.55. II: tr 0.23, fe 0.91, pa 0.50, ti 0.78, mt 0.80, ta 0.92. III: tr 0.22, fe 0.58, pa 0.34, ti 0.47, mt 0.50, ta 0.53. IV: tr 0.26, fe 0.89, pa 0.51, ti 0.75, mt 0.94, ta 0.63.

Dorsum (Fig. 110, 111). Eta (η) hourglass-shaped dorsal scutum. Ocularium low and rounded in shape. The dorsal scutum microgranulate, and both the opisthosoma and free tergites display rows of small setiferous tubercles. It is worth noting that the tubercle bases of these setiferous tubercles increase in length posteriorly.

Chelicerae (Fig. 112A, B). Segment I with a small tubercle on the dorso-distal surface. Segment II bearing sparse small setiferous tubercles, with one triangular tubercle prominent from the others in frontal view.

Pedipalps (Fig. 112C, D). Trochanter with two small dorsal and ventral tubercles. Femora bearing three dorsal spines with setae, five ventral-distal and two ventral-proximal. Patella with 2–3 small ventral setiferous tubercles. Tibia covered in small ventral tubercles, with two spines in the ventral-mesal area and three in the ventral-ectal areas. Tarsus with three mesal and ectal spines with subdistal setae in addition to setae and few granules.

Legs (Fig. 113). Coxa I with 9–10 setiferous tubercles (two distal tubercles stronger than the others), II with 12–13, III with six or seven, IV with four or five tubercles connected to the opisthosoma. Spiracles not obstructed by bridges. Smooth surface occupying 1/3 of leg II, ¾ of III and < 1/3 of IV. Leg zone II smooth with two or three small tubercles with subdistal setae on each side. Sternum arrow-shaped. Legs covered with small tubercles, astragalus longer than calcaneus in all legs, in leg II the calcaneus is slightly shorter than the astragalus. Tarsal count: 3–6–4–4.

Penis (Figs 114, 115). Pars distalis with a ventral plate bearing a thin cleft dividing the plate into two lamellae. Each lamella has three pointed macrosetae on the ventral surface and one short macroseta on the dorsal surface; capsula externa covering dorsal surface, with apical part folded dorsally, with a pair of long lateral processes; there is a dorsolateral plate attached to the pars basalis. Capsula externa shorter in length compared to the capsula interna. The capsula interna, on the other hand, is wide in shape, and it features a sharp apical area.

**Female.** Similar to males, with a shorter pedipalpal femora.

Female measurements. Total length 2.39, carapace length 1.1, dorsal scutum length 2.2, carapace max. width 1.57, mesotergum max. width 2.15. Appendage Measurements: Pedipalps. Trochanter length 0.23, femora length 0.77, patella length 0.50, tibia length 0.52, tarsus length 0.65. Leg I: trochanter (tr) 0.18, femora (fe) 0.72, patella (pa) 0.39, tibia (ti) 0.50, metatarsus (mt) 0.57, tarsus (ta) 0.47. II: tr 0.24, fe 0.90, pa 0.48, ti 0.71, mt 0.79, ta 0.87. III: tr 0.22, fe 0.56, pa 0.32, ti 0, 39, mt 0.49, ta 0.47. IV: tr 0.27, fe 0.81, pa 0.51, ti 0.70, mt 0.92, ta 0.59.

##### ﻿ Nerudiella malleco sp. nov.

Taxon classificationAnimaliaOpilionesTriaenonychidae

5668F87C-E7FA-5BA1-95FF-E2A82F4A008E

https://zoobank.org/18A11F7E-3279-470D-898C-9CB9A84C5599

**Figure 116.**
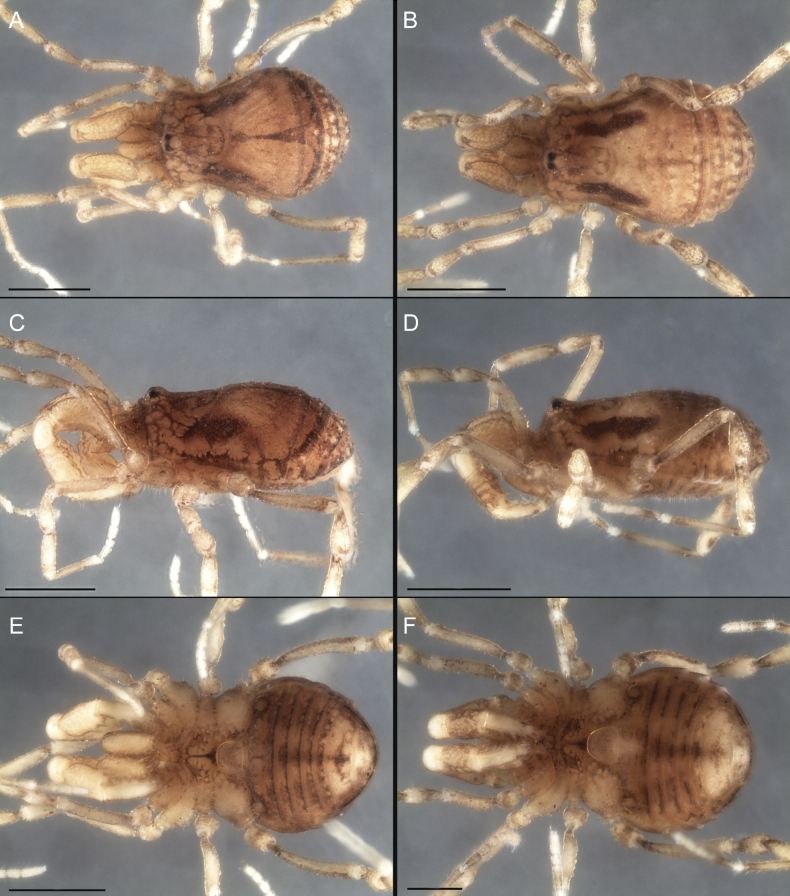
*Nerudiellamalleco* sp. nov. habitus, male **A** dorsal view **C** lateral view **E** ventral view. Female **B** dorsal view **D** lateral view **F** ventral view. Scale bars: 1 mm. Species of Clade C, see Fig. 3.

**Figure 117.**
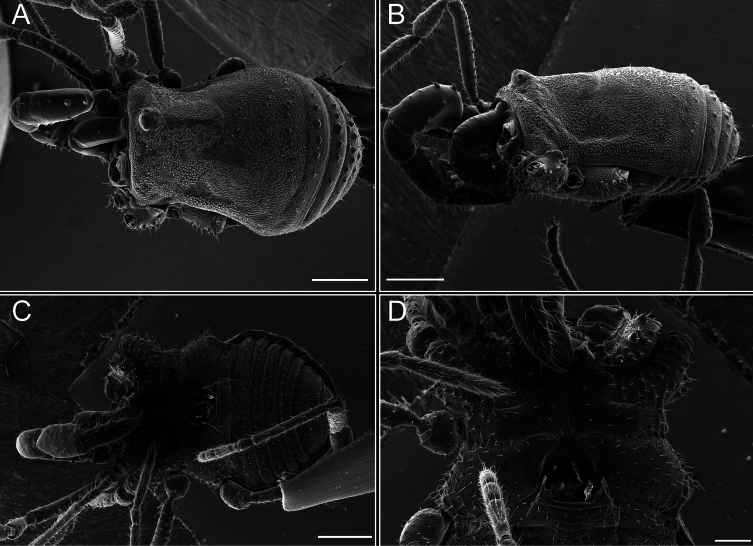
*Nerudiellamalleco* sp. nov. male, SEM images of habitus **A** dorsal view **B** lateral view **C, D** ventral view. Scale bars: 500 µm (**A, B, C**); 200 µm (**D**).

**Figure 118.**
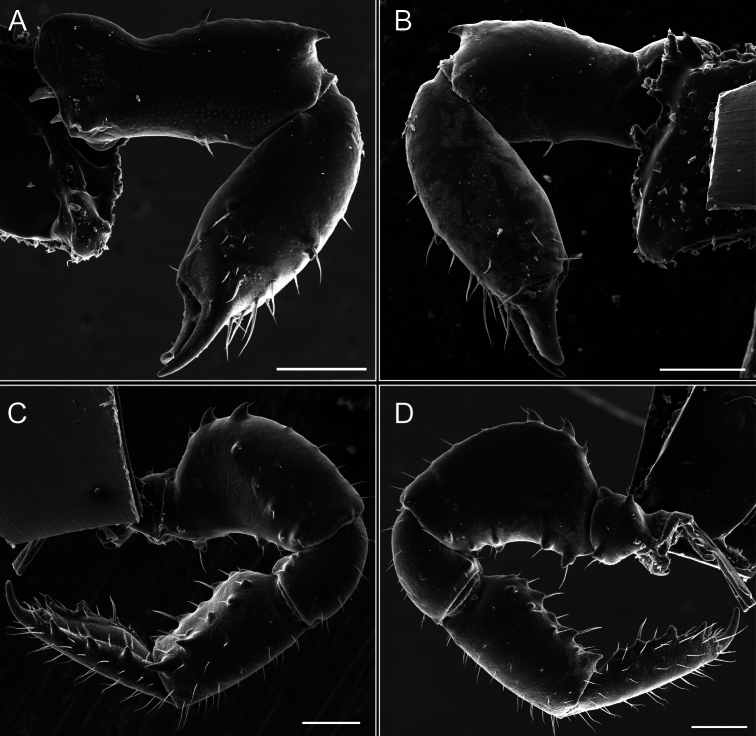
*Nerudiellamalleco* sp. nov. chelicerae: mesal **A** ectal **B** pedipalps: mesal **C** ectal **D**. Scale bars: 200 µm.

**Figure 119.**
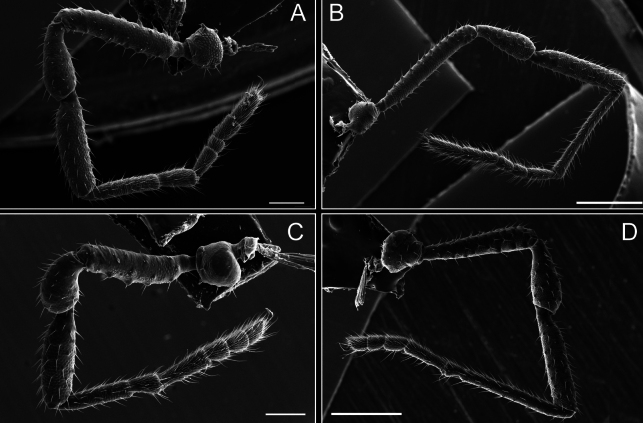
*Nerudiellamalleco* sp. nov. legs I **A** II **B** III **C** IV **D**. Scale bars: 200 µm (**A, C**); 500 µm (**B, D**).

**Figure 120.**
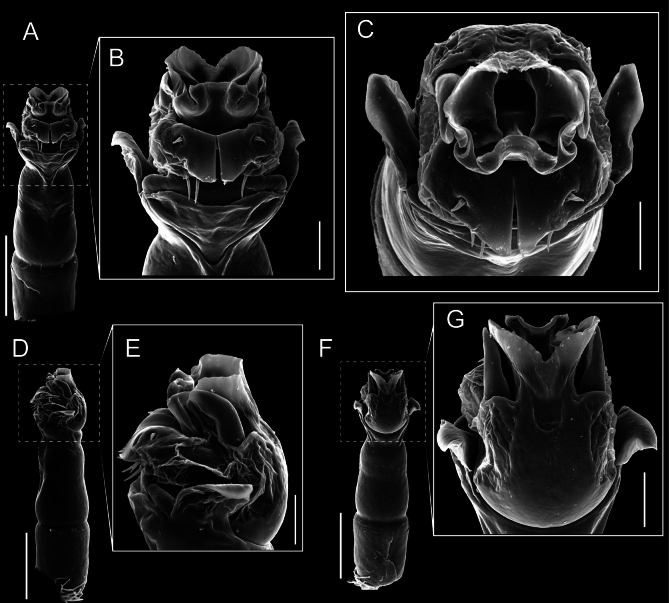
*Nerudiellamalleco* sp. nov. penis: ventral **A, B** apical **C** lateral **D, E** dorsal **F, G**. Scale bars: 200 µm (**A, D, F**); 50 µm (**B, C, E, G**).

**Figure 121.**
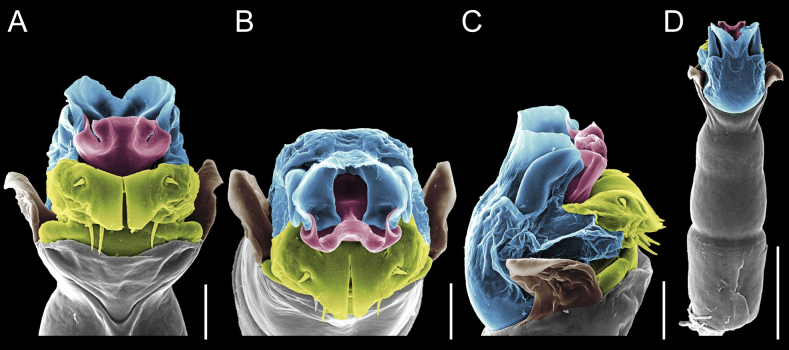
*Nerudiellamalleco* sp. nov. penis: ventral **A** apical **B** lateral **C** dorsal **D**. Colors: ventral plate (yellow), capsula externa (blue), capsula interna (red). Scale bars: 50 µm (**A–C**); 200 µm (**D**).

Figs 116 , 117 , 118 , 119 , 120 , 121

###### Material examined.

***Holotype*.** ♂ **Chile.** Malleco: Fundo Maria Esther, 15 km W from Victoria, E. Maury coll., 14.I.1989 (MNHNCL). ***Paratypes*. Chile.** Malleco: Fundo Maria Esther, 15 km W from Victoria, E. Maury coll., 14.I.1989, 2 ♂ (MACN), 08.I.1987 4 ♂ 1 ♀ 6 imm. (MACN), Monumento Natural Contulmo, E.M coll., 13. I.1989, 2 ♂ 3 ♀ (MACN), 15 km W Victoria, N. Platnick, O. Francke coll., 26.I.1985, 2 ♂ 3 ♀ 7 imm. (AMNH); Malalcahuello, E. Maury coll., 08. I.1987, 1 ♀ (MACN); 17 km E from Curacautín, N. Platnick, R. Schuh coll., 22.XI.1981, 1 ♂ 1 ♀ (AMNH), 1 imm. (AMNH), Monumento Natural Contulmo, E. Maury coll., 19.I.1991, 1 ♂ 4 ♀ (MACN). Same data, 1 imm. (AMNH).

###### Etymology.

The specific epithet refers to the type locality of the species, the Malleco province, located in the Araucanía region of Chile. Noun in apposition.

###### Diagnosis.

This species can be differentiated from other species within the genus by the distinct morphology of the male genitalia. Specifically, the male genitalia feature a robust and V-shaped process on the capsula externa when observed from a dorsal view.

###### Distribution.

Chile: Bío-Bío and Araucanía Regions (Fig. 4F).

###### Description of male holotype.

Measurements: Total length 4.1. Carapace length 1.1, Dorsal scutum length 2.3, Carapace max. width 1.21, Dorsal scutum max. width 1.6. Appendage measurements. Pedipalps. Trochanter length 0.26, femora length 0.98, patella length 0.47, tibia length 0.73, tarsus length 0.71. Leg I: trochanter (tr) 0.21, femora (fe) 0.82, patella (pa) 0.45, tibia (ti) 0.61, metatarsus (mt) 0.67, tarsus (ta) 0.61. II: tr 0.22, fe 1.05, pa 0.53, ti 0.73, mt 0.87, ta 1.03. III: tr 0.22, fe 0.74, pa 0.33, ti 0.55, mt 0.83, ta 0.67. IV: tr 0.25, fe 1.08, pa 0.61, ti 0.83, mt 1.12, ta 0.76.

Dorsum (Fig. 116, 117). Eta (η) hourglass-shaped dorsal scutum. Ocularium low and rounded. The dorsal scutum microgranulate. Mesotergal areas on the dorsal scutum do not have a clear separation but are covered in small setiferous tubercles. Additionally, the free tergites of the species bear a row of small setiferous tubercles.

Chelicerae (Fig. 118A, B). Segment I with an acute tubercle on the dorso-distal surface. Segment II bearing scattered setae in ectal and ventral views, with one triangular tubercle being more prominent than the others in front view.

Pedipalps (Fig. 118C, D). Trochanter with a small dorsal and ventral tubercle. Femora with three dorsoproximal setiferous spines, four small ventral-distal tubercles, and two ventral-proximal spines. Tibia with three ventral-ectal and two ventral-mesal spines. Tarsus with three mesal and ectal spines, accompanied by subdistal setae.

Legs (Fig. 119). Coxa I with nine or ten setiferous tubercles, II with 18–20 setiferous tubercles, IV with seven or eight small tubercles connected to the opisthosoma. Spiracles not obstructed by bridges. Smooth surface occupying 1/3 of leg II, ¾ of III and < 1/3 of IV. Smooth area of leg II with two small tubercles with subdistal setae on each side. Sternum arrow-shaped. Legs covered with small tubercles, astragalus longer than calcaneus on all legs. Tarsal count: 3–6–4–4.

Penis (Figs 120, 121). Pars distalis with a ventral plate bearing a fine cleft dividing the plate into two lamellae. Each lamella has three pointed macrosetae on the ventral surface and one small macroseta on the dorsal surface; capsula externa covering dorsal surface, apical area of capsula externa with a V-shaped notch and a pair of lateral processes; there is a dorsolateral plate connected to the pars basalis; dorsolateral plate present. Capsula interna short, with two slits in ventral view, without visible stylus.

**Female.** Females similar to males, with shorter pedipalpal femora.

Female measurements. Total length 4.07, carapace length 1.16, dorsal scutum length 2.71, carapace max. width 1.41, mesotergum max. width 1.96. Appendage measurements: Pedipalps. Trochanter length 0.26, length of the femora 0.95, length of the patella 0.47, length of the tibia 0.73, length of the tarsus 0.74. Leg I: trochanter (tr) 0.28, femora (fe) 0.98, patella (pa) 0.55, tibia (ti) 0.77, metatarsus (mt) 0.85, tarsus (ta) 0.77. II: tr 0.31, fe 1.27, pa 0.68, ti 1.06, mt 1.17, ta 1.34. III: tr 0.30, fe 0.90, pa 0.41, ti 0.61, mt 0.72, ta 0.76. IV: tr 0.37, fe 1.28, pa 0.70, ti 0.99, mt 1.22, ta 0.92.

##### ﻿ Nerudiella penco sp. nov.

Taxon classificationAnimaliaOpilionesTriaenonychidae

6C688D81-8382-5D82-9C4D-0B48E94CCC7F

https://zoobank.org/F00EE6F1-AC0A-4EBE-A71F-D5A7AC8DDD35

**Figure 122.**
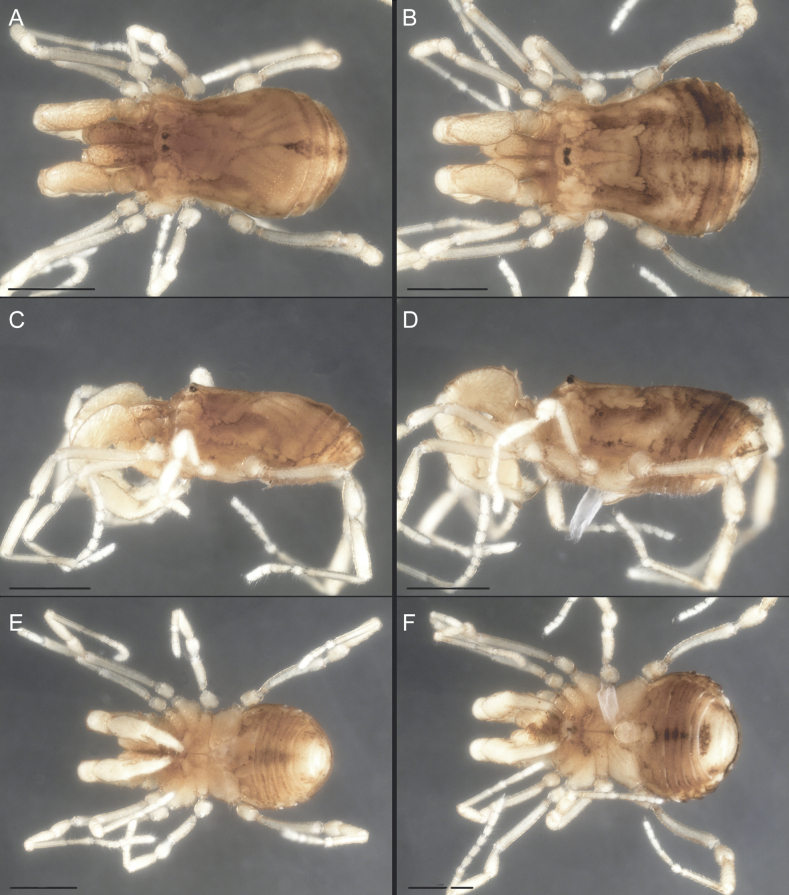
*Nerudiellapenco* sp. nov. habitus, male **A** dorsal view **C** lateral view **E** ventral view. Female **B** dorsal view **D** lateral view **F** ventral view. Scale bars: 1 mm.

**Figure 123.**
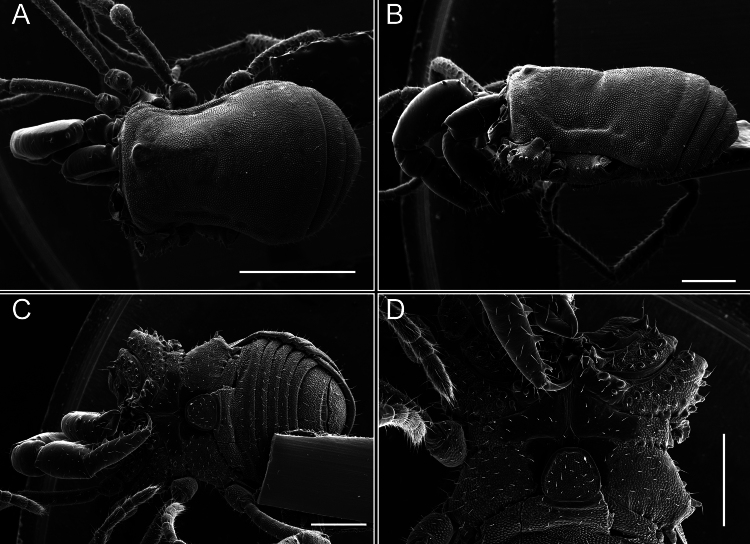
*Nerudiellapenco* sp. nov. male, SEM images of habitus **A** dorsal view **B** lateral view **C, D** ventral view. Scale bars: 500 µm.

**Figure 124.**
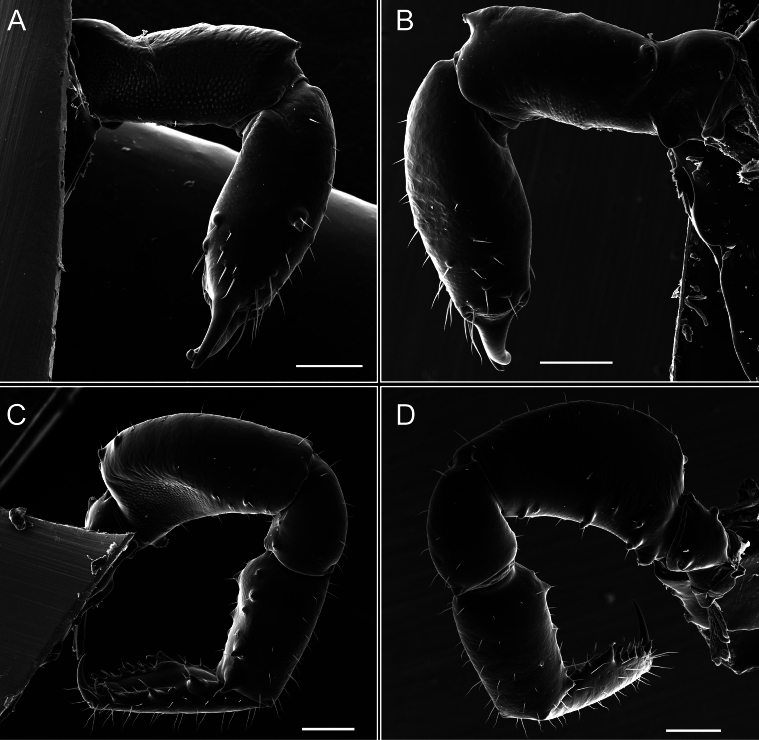
*Nerudiellapenco* sp. nov. chelicerae: mesal **A** ectal **B** pedipalps: mesal **C** ectal **D**. Scale bars: 200 µm.

**Figure 125.**
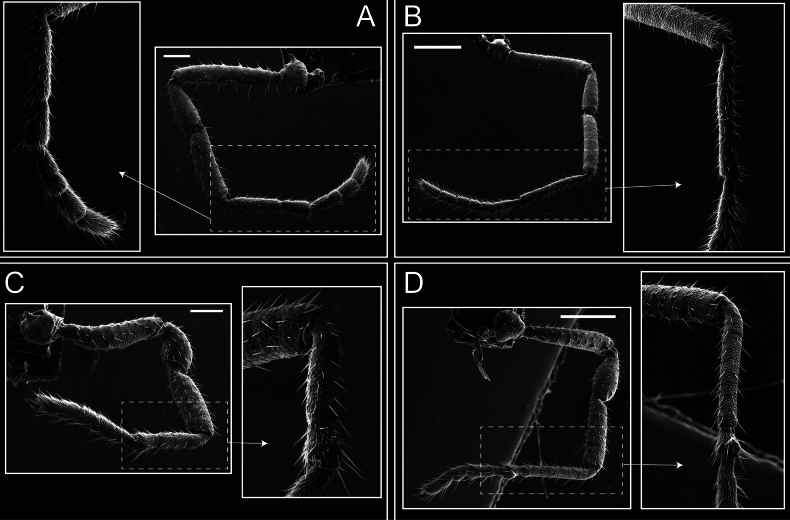
*Nerudiellapenco* sp. nov. legs I **A** II **B** III **C** IV **D**. Scale bars: 200 µm (**A, C**); 500 µm (**B, D**).

**Figure 126.**
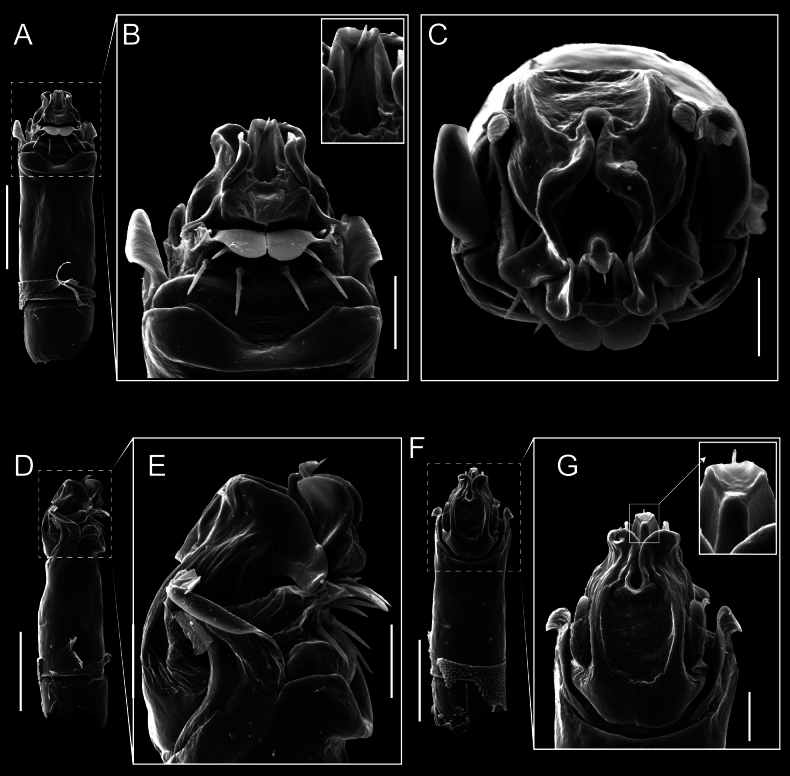
*Nerudiellapenco* sp. nov. penis: ventral **A, B** apical **C** lateral **D, E** dorsal **F, G**. Scale bars: 200 µm (**A, D, F**); 50 µm (**B, C, E, G**).

**Figure 127.**
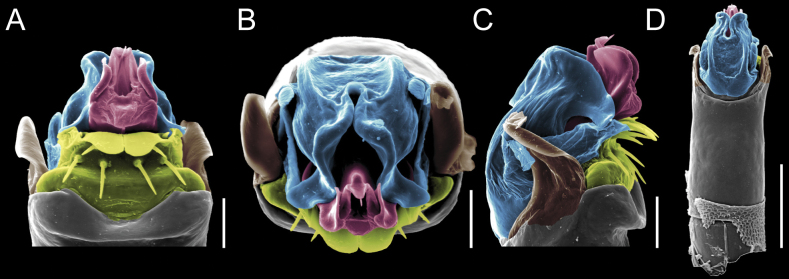
*Nerudiellapenco* sp. nov. penis: ventral **A** apical **B** lateral **C** dorsal **D**. Colors: ventral plate (yellow), capsula externa (blue), capsula interna (red). Scale bars: 50 µm.

Figs 122 , 123 , 124 , 125 , 126 , 127

###### Material examined.

***Holotype*.** ♂ **Chile.** Concepción: Estero Nonguén, N. Platnick, R. Schuh coll., 16.XI.1981 (AMNH). ***Paratypes*. Chile.** Concepción: Penco, T. Cekalovic coll., 11.II.1979, 1 ♀ (AMNH).

###### Etymology.

The specific epithet refers to the type locality of the species, the commune of Penco, located in the province of Concepción, Chile. Noun in apposition.

###### Diagnosis.

This species can be distinguished from the other species in the genus by having the dorsal scutum without tubercles. The male genitalia has longer macrosetae than other species of the genus, capsula externa divided into two halves that touch in the apical portion, leaving a gap between the two halves.

###### Distribution.

Chile: Bío-Bío Region (Fig. 4F).

###### Description of male holotype.

Measurements: Total length 3.67. Carapace length 0.97, dorsal scutum length 1.90, carapace max. width 1.26, mesotergum max. width 1.55. Appendage measurements: Pedipalps. Trochanter length 0.24, femora length 0.98, patella length 0.53, tibia length 0.70, tarsus length 0.71. Leg I: trochanter (tr) 0.19, femora (fe) 0.85, patella (pa) 0.46, tibia (ti) 0.60, metatarsus (mt) 0.70, tarsus (ta) 0.63. II: tr 0.25, fe 1.17, pa 0.56, ti 0.83, mt 0.99, ta 1.27. III: tr 0.26, fe 0.77, pa 0.32, ti 0.55, mt 0.57, ta 0.71. IV: tr 0.30, fe 1.07, pa 0.55, ti 0.85, mt 1.04, ta 0.89.

Dorsum (Fig. 122, 123). Eta (η) hourglass-shaped dorsal scutum. Ocularium low. Dorsal scutum microgranulate, without clear delimitation of areas. Areas III–IV smooth, with few setae; posterior margin with a row of setae. Free tergites have a row of setae.

Chelicerae (Fig. 124A, B). Segment I with a small tubercle on the dorsal-distal surface. Segment II with a mesal tubercle and bearing few setae.

Pedipalps (Fig. 124C, D). Trochanter with a small dorsal and ectal tubercle. Femora with a row of three spines and one setiferous granule on the ventral surface, three small dorsal spines with subdistal setae, and three meso-distal setiferous granules. Patella with 1–2 ventral setiferous granules. Tibia with three ectal and mesal spines with subdistal setae; presence of scant granules in ventral view. Tarsus with an ectal row of two subdistal setiferous spines and 6–8 small setiferous tubercles, there are three spines with subdistal setae on the mesal surface.

Legs (Fig. 125). Coxae I and II covered in setiferous tubercles, III and IV only with the microgranulation, bearing three bridges between legs II and III, five or six between III and IV, four between leg IV and the opisthosoma (the longest being the most distal). Spiracles not obstructed by bridges. Smooth area occupying 1/3 of leg II (with two small tubercles and two rounded tubercles), 2/3 of III and < 1/3 of IV. Sternum arrow-shaped. Legs covered in small tubercles, astragalus longer than calcaneus on all legs. Tarsal count: 3–6–4–4.

Penis (Figs 126, 127). Pars distalis with a large ventral plate bearing a cleft that divides the plate into two lamellae. Each lamella has three long pointed macrosetae on the ventral surface and one macroseta on the dorsal surface; capsula externa covering dorsal and lateral surfaces, having a cleft dividing the capsula externa into two halves; there is a dorsolateral plate attached to the pars basalis. Capsula interna longer than the capsula externa, partially covering the ventral plate, with a visible stylus in its apical portion.

**Female.** Similar to males, with shorter pedipalpal femora.

Female measurements. Total length 4.06, carapace length 1.12, dorsal scutum length 2.16, carapace max. width 1.46, mesotergum max. width 2.04. Appendage Measurements: Pedipalps. Trochanter length 0.27, femora length 0.97, patella length 0.51, tibia length 0.68, tarsus length 0.82. Leg I: trochanter (tr) 0.27, femora (fe) 0.99, patella (pa) 0.50, tibia (ti) 0.73, metatarsus (mt) 0.81, tarsus (ta) 0.73. II: tr 0.30, fe 1.15, pa 0.65, ti 0.82, mt 1.00, ta 1.00. III: tr 0.24, fe 0.81, pa 0.45, ti 0.60, mt 0.68, ta 0.77. IV: tr 0.36, fe 1.24, pa 0.69, ti 1.04, mt 1.26, ta 1.20.

##### ﻿ Nerudiella pichi sp. nov.

Taxon classificationAnimaliaOpilionesTriaenonychidae

C40285C4-CFEE-5739-B347-AB8F0C277BE4

https://zoobank.org/7727BE05-A618-4D2A-A79C-291D73318A6D

**Figure 128.**
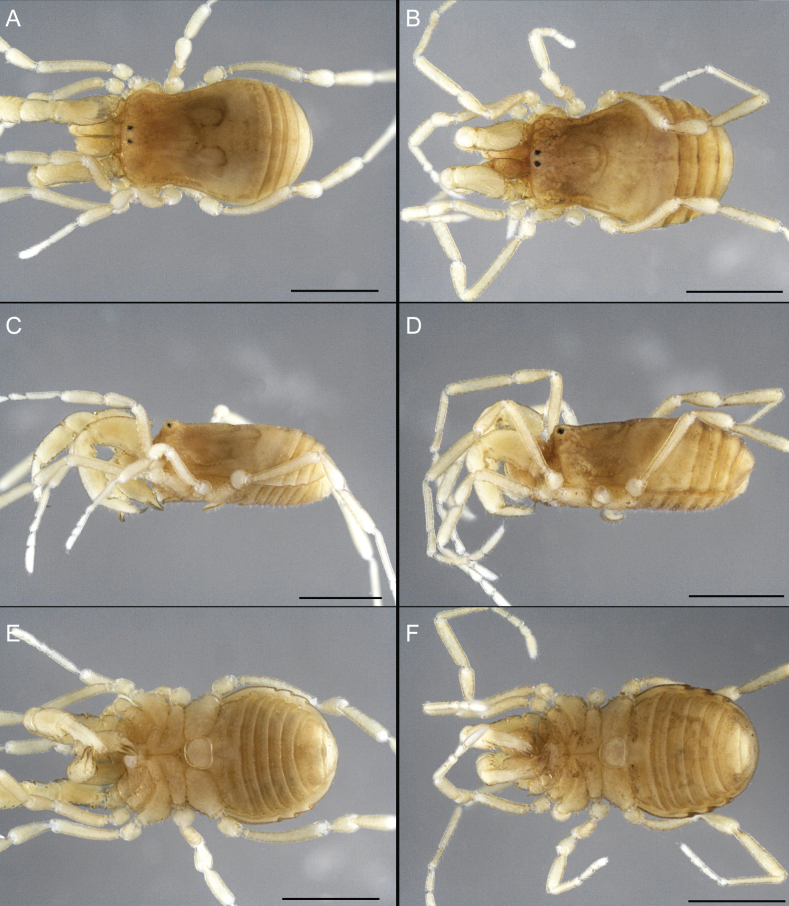
*Nerudiellapichi* sp. nov. habitus, male **A** dorsal view **C** lateral view **E** ventral view. Female **B** dorsal view **D** lateral view **F** ventral view. Scale bars: 1 mm.

**Figure 129.**
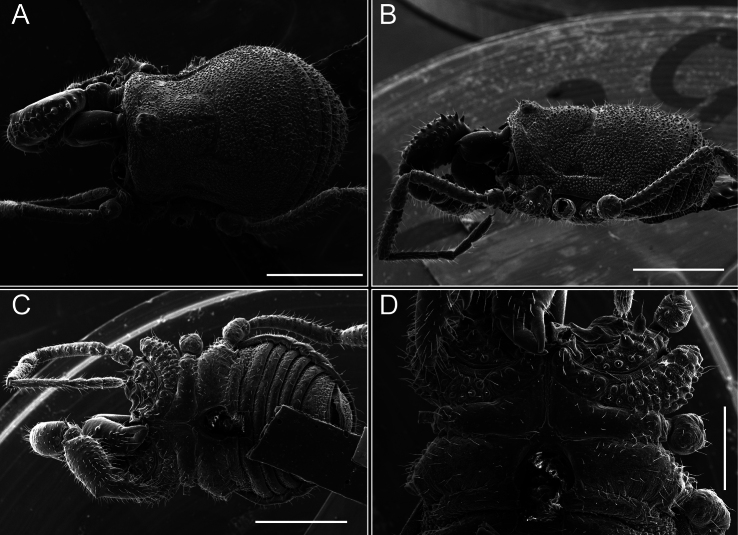
*Nerudiellapichi* sp. nov. male, SEM images of habitus **A** dorsal view **B** lateral view **C, D** ventral view. Scale bars: 500 µm.

**Figure 130.**
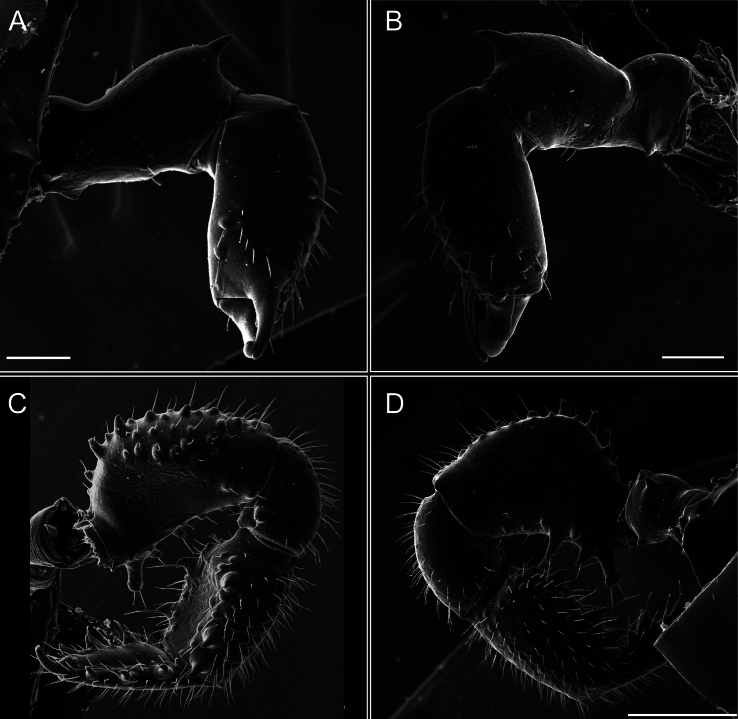
*Nerudiellapichi* sp. nov. chelicerae: mesal **A** ectal **B** pedipalps: mesal **C** ectal **D**. Scale bars: 200 µm (**A, B**); 500 µm (**C, D**).

**Figure 131.**
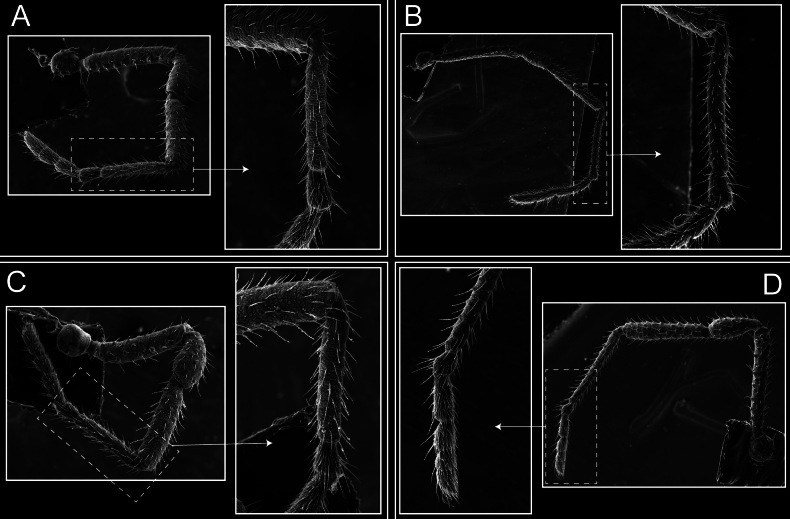
*Nerudiellapichi* sp. nov. legs I **A** II **B** III **C** IV **D**. Scale bars: 200 µm (**A, C**); 500 µm (**B, D**).

**Figure 132.**
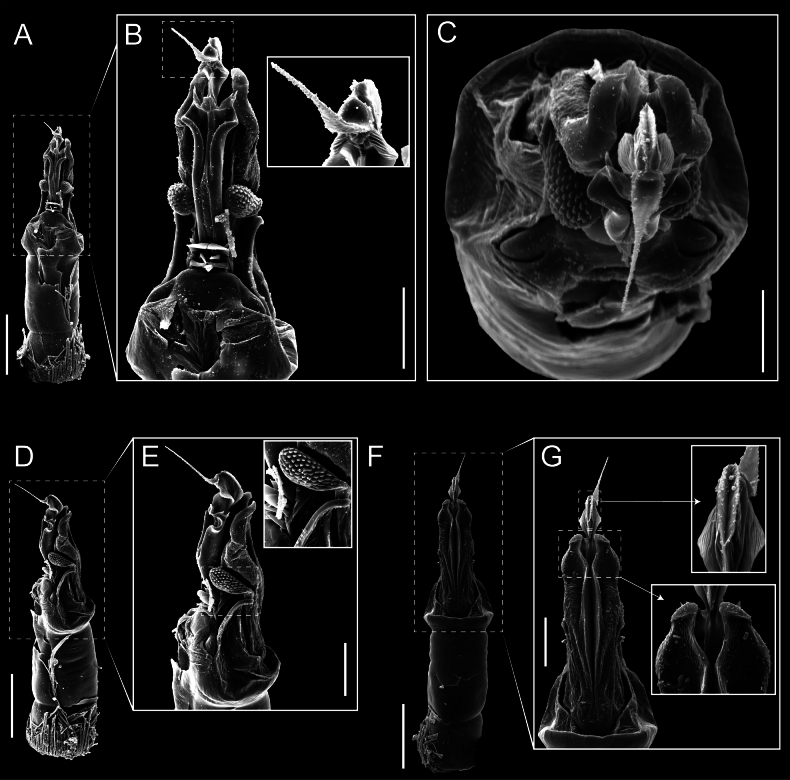
*Nerudiellapichi* sp. nov. penis: ventral **A, B** apical **C** lateral **D, E** dorsal **F, G**. Scale bars: 200 µm (**A, D, F**); 100 µm (**B, E, G**); 50 µm (**C**).

**Figure 133.**
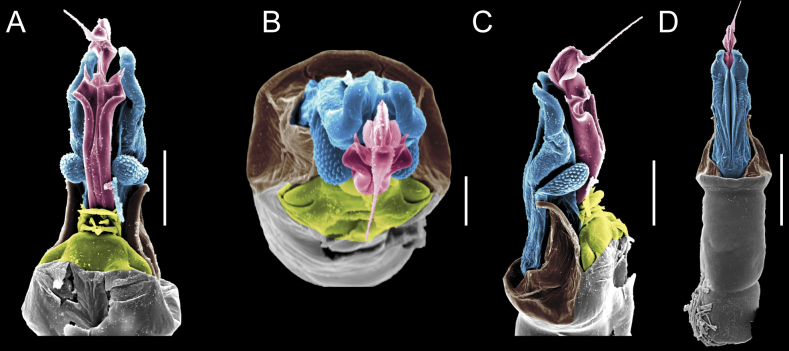
*Nerudiellapichi* sp. nov. penis: ventral **A** apical **B** lateral **C** dorsal **D**. Colors: ventral plate (yellow), capsula externa (blue), capsula interna (red). Scale bars: 100 µm (**A, C, D**); 50 µm (**B**).

Figs 128 , 129 , 130 , 131 , 132 , 133

###### Material examined.

***Holotype*.** ♂ **Chile.** Choapa: Pichidangui, Valparaíso, Cerro La Silla del Gobernador, E. Maury coll. 30.X.1988 (MNHNCL). ***Paratypes*. Chile.** Choapa: Pichidangui, Valparaíso, Quebrada near Puente Totoralillo, 13.XI.1987, E. Maury coll. 1 ♂ (MACN). Los Vilos, Cuesta Caviolén, E. Maury, 12.XI.1987 1 ♂ 2 ♀ (MACN). Valparaíso, 10 km S de Casablanca, E. Maury, 13.I.1984 2 ♀ (MACN). Pichidangui, Valparaíso, Quebrada near Puente Totoralillo, E. Maury coll. 13.XI.1987, 7 ♂ 1 ♀ (MACN). Valparaíso: Quebrada El Tigre 25 km E Zapallar, M. Ramírez, J. Pizarro coll., 12.II.2011, 1 ♀ (MACN).

###### Etymology.

The specific epithet refers to “Pichí” which comes from Mapudungun, the language of the Mapuche (original people of the region), meaning small. Noun in apposition.

###### Diagnosis.

This species can be distinguished from the other species of the genus by having the dorsal microgranulate; Femora and tibia of the pedipalp with small tubercles. The male genitalia have a reduced ventral plate, a very long capsula interna and capsula externa, as well as a long fine structure at the apex of the capsula interna.

###### Distribution.

Chile: Valparaíso Region.

###### Description of male holotype.

Measurements: Total length 2.65, carapace length 1.12, dorsal scutum length 2.21, carapace max. width 1.64, mesotergum max. width 2.02. Appendage measurements: Pedipalps. Trochanter length 0.28, femora length 1.12, patella length 0.73, tibia length 0.77, tarsus length 0.80. Leg I: trochanter (tr) 0.20, femora (fe) 1.00, patella (pa) 0.52, tibia (ti) 0.73, metatarsus (mt) 0.90, tarsus (ta) 0.77. II: tr 0.27, fe 1.37, pa 0.63, ti 1.03, mt 1.14, ta 1.36. III: tr 0.27, fe 0.87, pa 0.40, ti 0.71, mt 0.90, ta 0.76. IV: tr 0.35, fe 1.29, pa 0.65, ti 1.05, mt 1.32, ta 0.86.

Dorsum (Fig. 128, 129). Eta (η) hourglass-shaped dorsal scutum. Low ocularium. Dorsal scutum microgranulate, without clear delimitation of areas. The microgranulation and the setiferous tubercles are almost the same size, so it is difficult to delimit the rows of tubercles that are present in the dorsal scute and free tergites.

Chelicerae (Fig. 130A, B). Segment I with a small tubercle on the dorso-distal surface. Segment II bearing scant setae.

Pedipalps (Fig. 130C, D). Trochanter with a small dorsal and ectal tubercle. Femora bearing an ectal row of setiferous tubercles; dorsal surface with a row of spines and a parallel row of setiferous granules on each side. Patella with a ventral setiferous tubercle, dorsal surface covered with setae. Tibia covered in setiferous granules, with three ectal and mesal spines with subdistal setae on each side. Tarsus with three mesal and four ectal spines with subdistal setae and setae and scant granules.

Legs (Fig. 131). Coxa I with setiferous tubercles, the large one with subdistal setae, II with setiferous tubercles, III and IV only with the microgranulation, three bridges between legs II and III, three between III and IV, five between leg IV and opisthosoma. Spiracles not obstructed by bridges. Smooth surface occupying 1/3 of leg II, ¾ of leg III and < 1/3 of leg IV. Sternum arrow-shaped. Legs covered in small tubercles, astragalus longer than calcaneus on all legs. Tarsal count: 3–5–4–4.

Penis (Figs 132, 133). Pars distalis with a ventral plate reduced in its apical portion bearing a cleft that divides the plate into two halves. Each half has three pointed macrosetae on the ventral surface and one macroseta on the dorsal surface; capsula externa covering dorsal and lateral surfaces, having a very long cleft dividing the capsula interna into two halves, including two rounded, microsculpted processes; there is a dorsolateral plate attached to the pars basalis. Capsula interna longer than the capsula externa, the apical portion is long and thin.

**Female.** Similar to male, with shorter pedipalpal femora.

Female measurements. Total length 2.49, carapace length 1.00, dorsal scutum length 2.15, carapace max. width 1.54, mesotergum max. width 2.10. Appendage measurements: Pedipalps. Trochanter length 0.25, femora length 0.89, patella length 0.53, tibia length 0.56, tarsus length 0.67. Leg I: trochanter (tr) 0.24, femora (fe) 0.89, patella (pa) 0.48, tibia (ti) 0.62, metatarsus (mt) 0.82, tarsus (ta) 0.57. II: tr 0.28, fe 1.17, pa 0.45, ti 0.90, mt 1.06, ta 1.14. III: tr 0.30, fe 0.80, pa 0.38, ti 0.63, mt 0.80, ta 0.67. IV: tr 0.33, fe 1.17, pa 0.55, ti 0.96, mt 1.11, ta 0.80.

##### ﻿ Nerudiella portai sp. nov.

Taxon classificationAnimaliaOpilionesTriaenonychidae

6ECE74A1-9D0C-5B3C-B4B7-929BA41A192C

https://zoobank.org/A678AF8D-AC23-4F53-95F2-2EA3D2DF35A9

**Figure 134.**
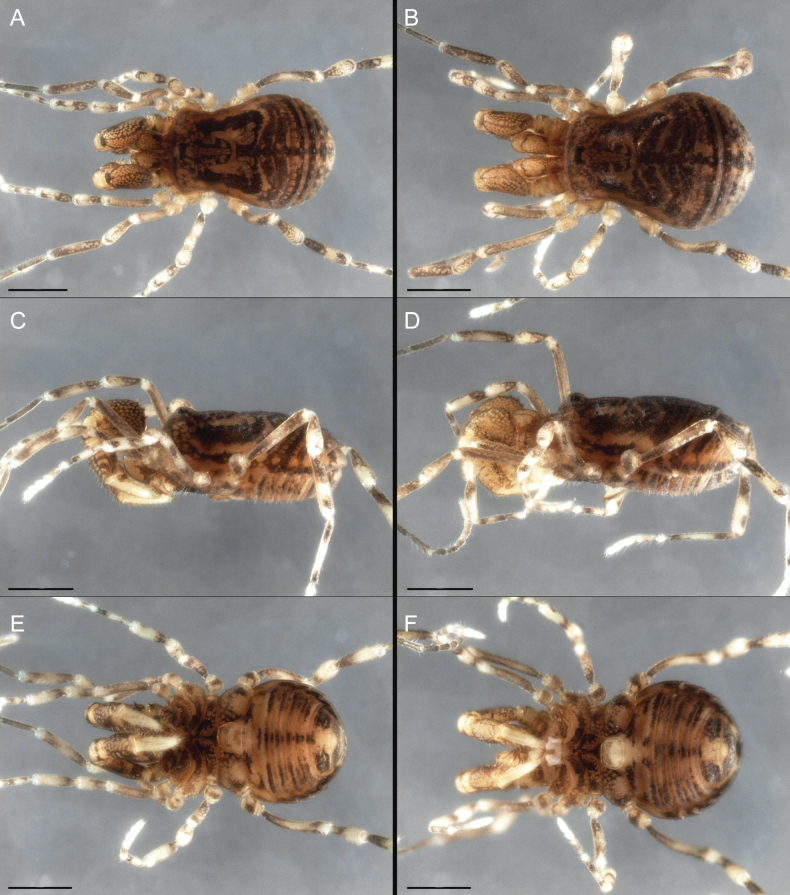
*Nerudiellaportai* sp. nov. habitus, male **A** dorsal view **C** lateral view **E** ventral view. Female **B** dorsal view **D** lateral view **F** ventral view. Scale bars: 1 mm. Species of Clade C, see Fig. 3.

**Figure 135.**
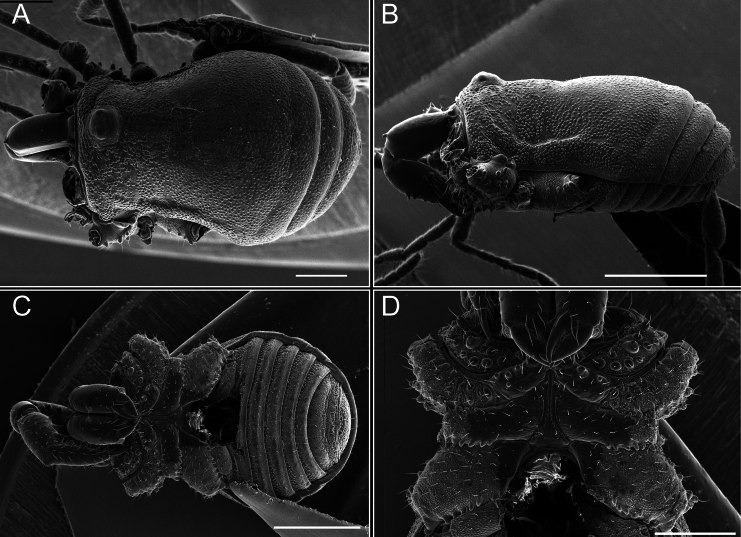
*Nerudiellaportai* sp. nov. male, SEM images of habitus **A** dorsal view **B** lateral view **C, D** ventral view. Scale bars: 500 µm.

**Figure 136.**
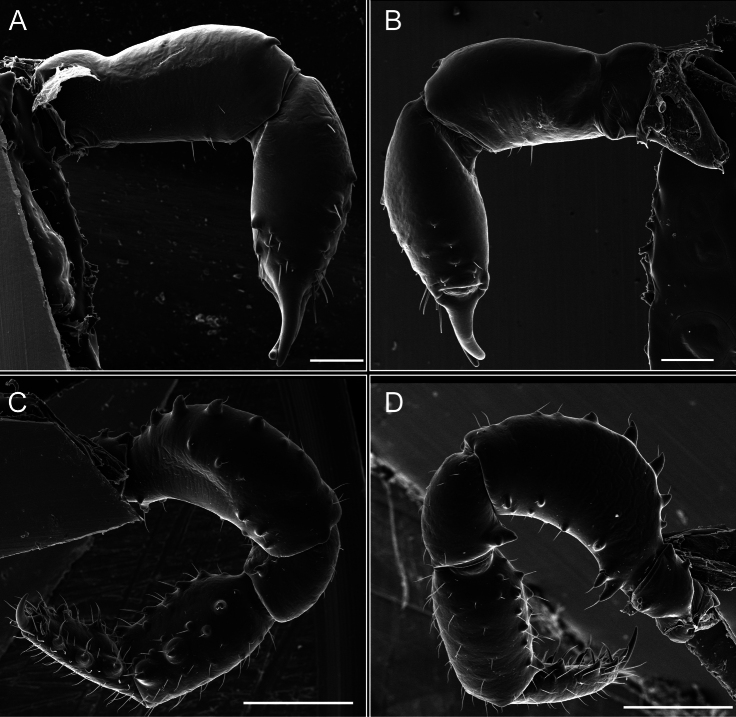
*Nerudiellaportai* sp. nov. chelicerae: mesal **A** ectal **B** pedipalps: mesal **C** ectal **D**. Scale bars: 200 µm (**A, B**); 500 µm (**C, D**).

**Figure 137.**
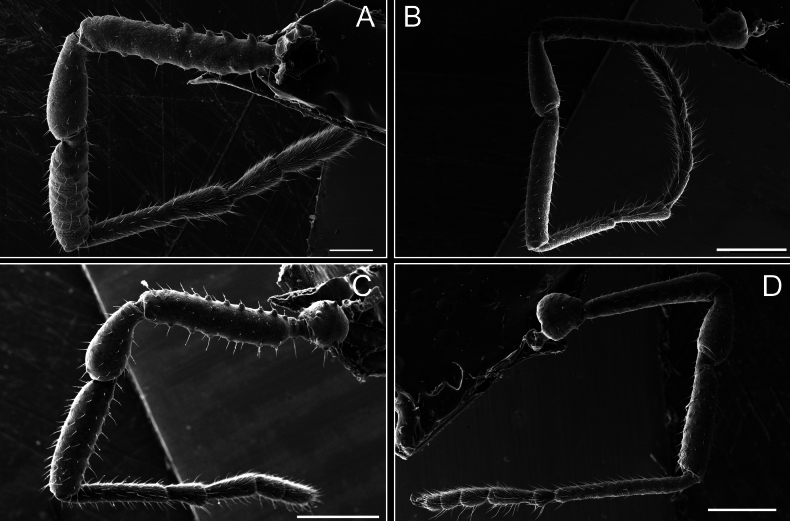
*Nerudiellaportai* sp. nov. legs I **A** II **B** III **C** IV **D**. Scale bars: 200 µm (**A**); 500 µm (**B–D**).

**Figure 138.**
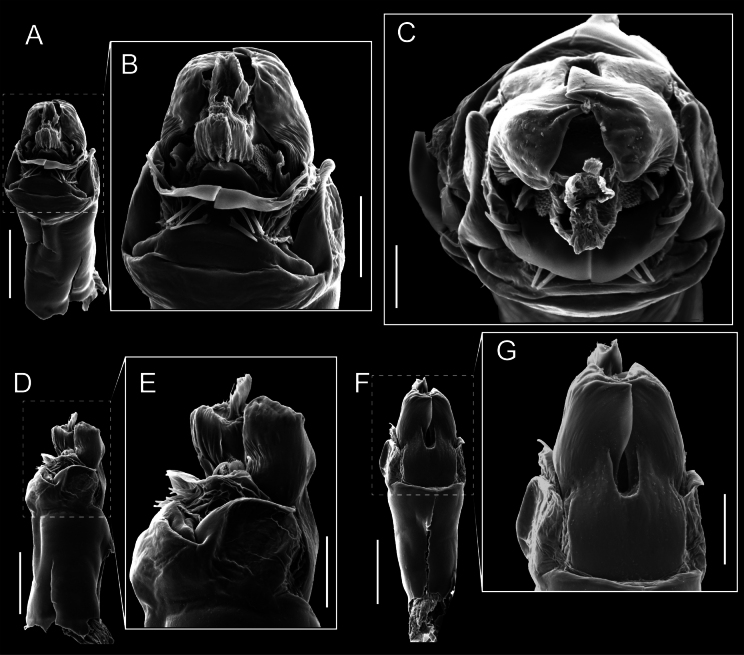
*Nerudiellaportai* sp. nov. penis: ventral **A, B** apical **C** lateral **D, E** dorsal **F, G**. Scale bars: 200 µm (**A, D, F**); 100 µm (**B, C, E, G**).

**Figure 139.**
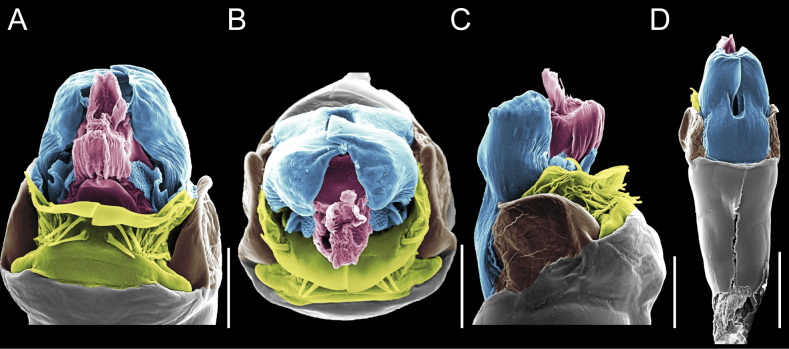
*Nerudiellaportai* sp. nov. penis: ventral **A** apical **B** lateral **C** dorsal **D**. Colors: ventral plate (yellow), capsula externa (blue), capsula interna (red). Scale bars: 100 µm (**A–C**); 200 µm (**D**).

Figs 134 , 135 , 136 , 137 , 138 , 139

###### Material examined.

***Holotype*.** ♂ **Chile.** Petorca: Cachagua, Quebrada El Tigre, E. Maury coll. 08.XI.1988 (MNHNCL). ***Paratypes*. Chile.** Petorca: Cachagua, Quebrada El Tigre, E. Maury coll., 08.XI.1988, 10 ♂, 12 ♀, 2 imm. (MACN). Same data, 6 ♂ (MACN).

###### Additional material.

Chile. Quillota: Olmue, La Campana, S. Peck, J. Peck coll., 02.XII.1984, 4 ♂ 3 ♀ 4 imm. (FMNH). Valparaíso: Petorca: Zapallar -32.551318°, -71.459492° 200 m (GE), Tullgren Berlese, park bordering the beach, A. Porta coll., 28.XII.2017, 2 ♂, 20 imm. (MACN).

###### Etymology.

Patronym in honor of Argentine arachnologist, acarologist, and mathematician Andrés Porta.

###### Diagnosis.

This species can be distinguished from the other species in the genus by the morphology of the male genitalia, which includes the robust capsula externa with a U-shaped slit in the middle of the capsula externa; and wide capsula interna with an apical constriction.

###### Distribution.

Chile: Valparaíso Region (Fig. 4F).

###### Description of male holotype.

Measurements: Total length 4.00, carapace length 1.05, dorsal scutum length 2.1, carapace max. width 1.34, mesotergum max. width 1.91. Appendage measurements. Pedipalps. Trochanter length 0.18, femora length 0.93, patella length 0.52, tibia length 0.68, tarsus length 0.65. Leg I: trochanter (tr) 0.21, femora (fe) 0.95, patella (pa) 0.52, tibia (ti) 0.70, metatarsus (mt) 0.82, tarsus (ta) 0.69. II: tr 0.20, fe 1.29, pa 0.61, ti 0.88, mt 1.14, ta 1.38. III: tr 0.30, fe 0.75, pa 0.41, ti 0.57, mt 0.68, ta 0.82. IV: tr 0.36, fe 1.18, pa 0.55, ti 1.00, mt 1.025, ta 0.90.

Dorsum (Fig. 134, 135). Eta (η) hourglass-shaped dorsal scutum. Ocularium low, rounded. Dorsal scutum microgranulate, without prominent tubercles.

Chelicerae (Fig. 136A, B). Segment I with a small tubercle on the dorso-distal surface. Segment II bearing sparse small setiferous tubercles, with one triangular tubercle prominent from the others in frontal view.

Pedipalps (Fig. 136C, D). Trochanter with a small dorsal and ventral tubercle. Femora bearing two parallel dorsal rows of setiferous spines, the ectal row has stronger spines than the mesal, it also has two proximal spines. Patella with a ventral-ectal setiferous tubercle and two ventral-ectal spines. Tibia covered in small ventral tubercles and two mesal spines. Tarsus with three mesal and ectal spines with subdistal setae in addition to setae and few granules.

Legs (Fig. 137). Coxa I with nine or ten setiferous tubercles, IV with six or seven tubercles connected to opisthosoma. Spiracles not obstructed by bridges. Smooth surface occupying 1/3 of leg II, ¾ of III and < 1/3 of IV. Leg II smooth with two or three small tubercles with subdistal setae on each side. Sternum arrow-shaped. Legs covered in small tubercles, astragalus longer than calcaneus on all legs. Tarsal count: 3–6–4–4.

Penis (Figs 138, 139). Pars distalis with a ventral plate bearing a cleft dividing the plate into two halves. Each half has three pointed macrosetae on the ventral surface and one macroseta on the dorsal surface; capsula externa covering dorsal surface, bearing a U-shaped slit halfway up plate; there is a dorsolateral plate attached to the pars basalis. Capsula interna slightly longer than the capsula externa, without rigid structures. The apical area of the structure is irregularly shaped and does not have a visible stylus.

**Female.** Similar to male, with shorter pedipalpal femora.

Female measurements. Total length 4.11, carapace length 1.02, dorsal scutum length 2.15, carapace max. width 1.33, mesotergum max. width 1.99. Appendage measurements: Pedipalps. Trochanter length 0.30. Femora length 0.87, patella length 0.41, tibia length 0.64, tarsus length 0.66. Leg I: trochanter (tr) 0.24, femora (fe) 0.95, patella (pa) 0.54, tibia (ti) 0.59, metatarsus (mt) 0.78, tarsus (ta) 0.71. II: tr 0.27, fe 1.23, pa 0.54, ti 0.93, mt 1.09, ta 1.25. III: tr 0.28, fe 0.75, pa 0.38, ti 0.55, mt 0.71, ta 0.69. IV: tr 0.28, fe 1.28, pa 0.69, ti 0.93, mt 1.25, ta 0.87.

##### ﻿ Nerudiella quenes sp. nov.

Taxon classificationAnimaliaOpilionesTriaenonychidae

DB05928D-09F1-5657-8CC9-12537A214598

https://zoobank.org/5E95016E-5E90-4131-8289-9DA206E43881

Figs 140 , 141

###### Material examined.

***Holotype*.** ♂ **Chile.** Curicó Quebrada in front of Los Queñes, 34.99614°S, 70.80994°W, 665 m A. Ojanguren, A. Pérez-González, M. Ramírez, G. Azevedo, W. Porto coll., 10.I.2018 (MNHNCL). ***Paratypes*. Chile.** Curicó: Quebrada in front of Los Queñes, gorge in front of Los Queñes, 34.99614°S, 70.80994°W, 665 m, A. Ojanguren, A. Pérez-González, M. Ramírez, G. Azevedo, W. Porto coll.,10. I.2018, 1 ♀ 3 imm.

###### Etymology.

The specific epithet refers to the type locality of the species, the department of Los Queñes, located in the commune of Romeral, province of Curicó, Chile. Noun in apposition.

###### Diagnosis.

This species can be distinguished from the other species in the genus by the morphology of the male genitalia, which includes the robust capsula externa with a “V”-shaped slit and long capsula interna with a triangular apical portion.

###### Distribution.

Chile: Maule Region (Fig. 4F).

###### Description of male holotype.

Measurements: Total length 2.0, carapace length 0.84, dorsal scutum length 1.7, carapace max. width 12, mesotergum max. width 1.6. Appendage measurements: Pedipalps. Trochanter length 0.22, femora length 0.79, patella length 0.43, tibia length 0.56, tarsus length 0.66. Leg I: trochanter (tr) 0.21, femora (fe) 0.87, patella (pa) 0.47, tibia (ti) 0.69, metatarsus (mt) 0.77, tarsus (ta) 0.66. II: tr 0.20, fe 1.1, pa 0.55, ti 0.86, mt 0.96, ta 1.2. III: tr 0.28, fe 0.75, pa 0.34, ti 0.58, mt 0.70, ta 0.60. IV: tr 0.30, fe 1.1, pa 0.53, ti 0.85, mt 1.1, ta 0.66.

**Figure 140.**
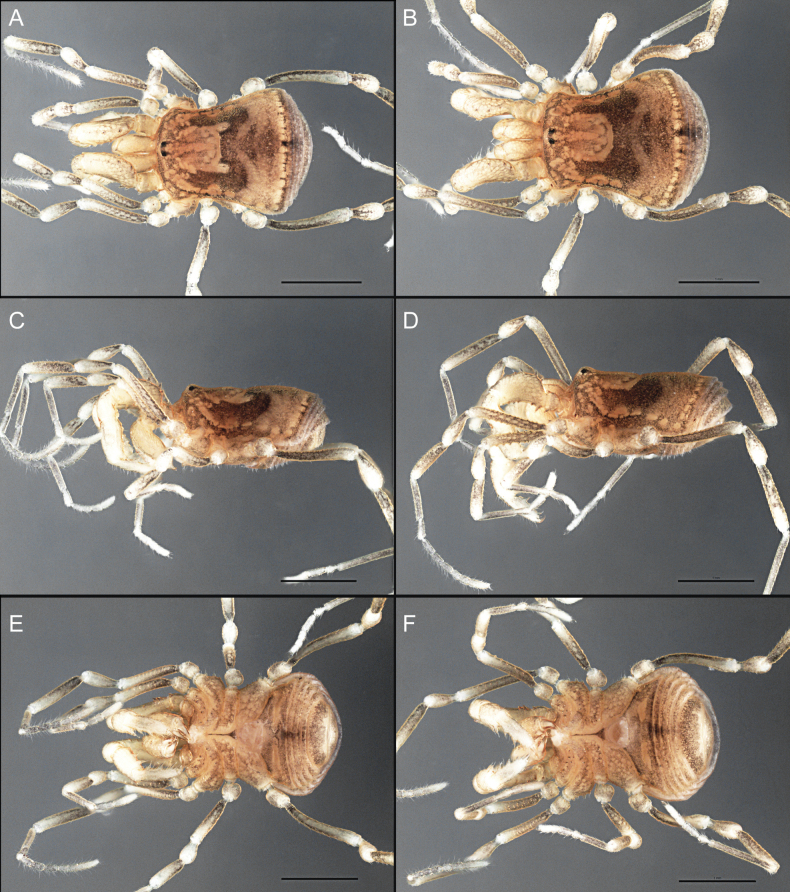
*Nerudiellaquenes* sp. nov. habitus, male **A** dorsal view **C** lateral view **E** ventral view. Female **B** dorsal view **D** lateral view **F** ventral view. Scale bars: 1 mm. Species of Clade C, see Fig. 3.

Dorsum (Fig. 140). Eta (η) hourglass-shaped dorsal scutum. Low ocularium. Dorsal scutum microgranulate, without clear delineation of areas.

Chelicerae. Segment I with a small tubercle on the dorso-distal surface. Segment II with a frontal tubercle and bearing few setae.

Pedipalps. Trochanter with a small ectal and dorsal tubercles. Femora with two prominent ventroproximal spines and three prominent dorsal spines. Patella with two ventral setiferous granules. Tibia with three ectal and two mesal spines with subdistal setae, with scant granules in ventral view. Tarsus with four ectal and three mesal spines with subdistal setae.

Legs. Coxa II with ~8 rounded setiferous tubercles. Spiracles not obstructed by bridges. Sternum arrow-shaped. Legs I–IV covered in setae, tarsal area, and calcaneus densely setose. Tibiae I–III with a ventral and dorsal row of small setiferous tubercles, IV with a row of four distal-ventral tubercles with setae. Calcaneus smaller than astragalus, ≥ 3× smaller (leg I), 4× (II, III), and 5× (leg IV). Tarsal count: 3–6–4–4.

**Figure 141.**
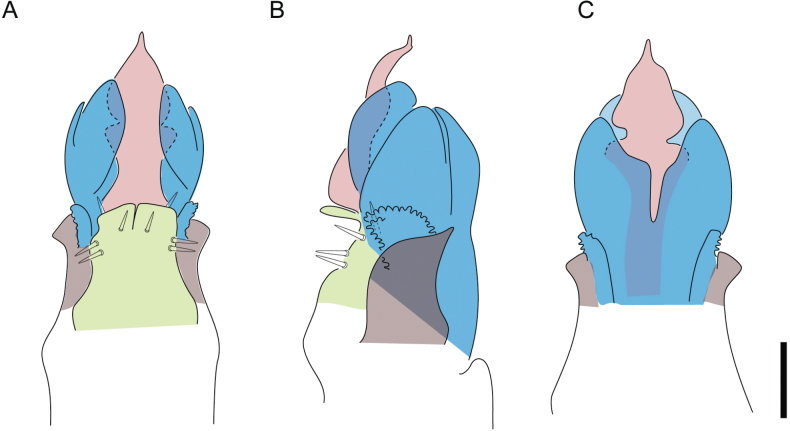
*Nerudiellaquenes* sp. nov. Penis ventral **A** lateral **B** dorsal **C** capsula interna (red), Capsula externa (blue), dorsolateral plate (brown), ventral plate (green). Scale bar: 100 µm.

Penis (Fig. 141). Pars distalis with a large ventral plate bearing a cleft that divides the plate into two lamellae. Each lamella has three pointed macrosetae on the ventral surface and one macroseta on the dorsal surface; capsula externa covering dorsal and lateral surfaces, having a cleft dividing dorsal fold into two halves; there is a dorsolateral plate attached to the pars basalis. Capsula interna longer than the capsula externa, with a visible stylus in its apical portion.

**Female.** Similar to male, with shorter pedipalpal femora.

Female measurements. Total length 2.07, carapace length 0.89, dorsal scutum length 1.72, carapace max. width 1.24, mesotergum max. width 1.77. Appendage measurements: Pedipalps. Trochanter length 0.19, femora length 0.76, patella length 0.47, tibia length 0.50, tarsus length 0.68. Leg I: trochanter (tr) 0.22, femora (fe) 0.84, patella (pa) 0.44, tibia (ti) 0.65, metatarsus (mt) 0.71, tarsus (ta) 0.64. II: tr 0.24, fe 1.09, pa 0.51, ti 0.87, mt 0.95, ta 1.3. III: tr 0.23, fe 0.75, pa 0.35, ti 0.60, mt 0.72, ta 0.61. IV: tr 0.31, fe 1.08, pa 0.57, ti 0.93, mt 1.16, ta 0.68.

##### ﻿ Nerudiella vilches sp. nov.

Taxon classificationAnimaliaOpilionesTriaenonychidae

ABD8C78B-7FEB-5C97-9601-2327FBEB1F2C

https://zoobank.org/1E1E59C3-6A5F-415A-B640-C49EB3A2AC77

**Figure 142.**
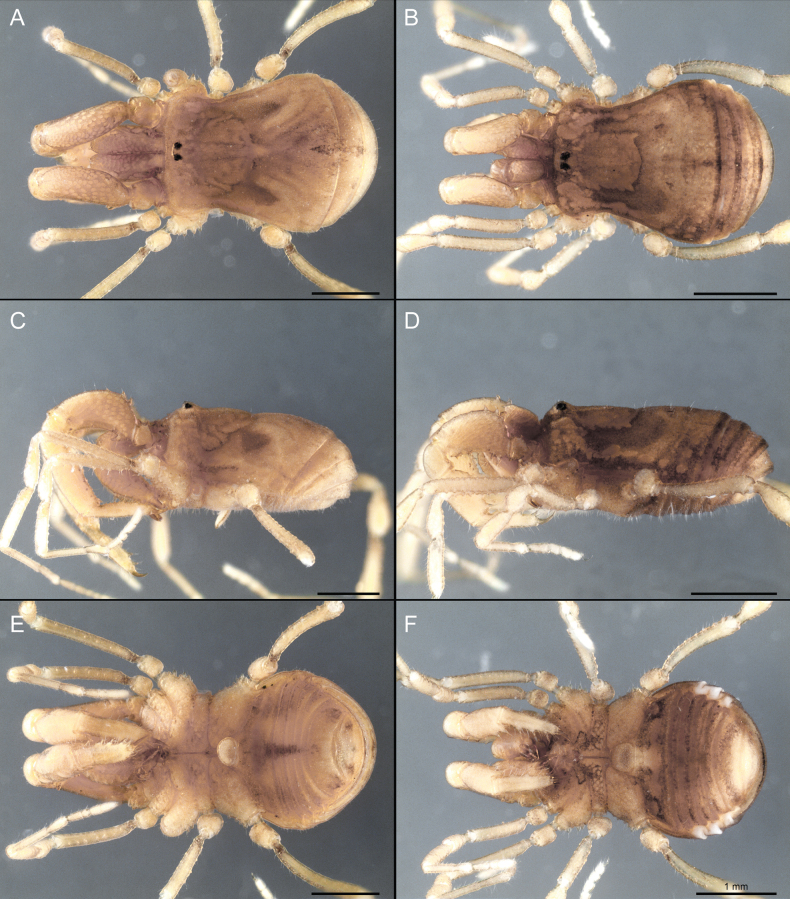
*Nerudiellavilches* sp. nov. habitus, male **A** dorsal view **C** lateral view **E** ventral view. Female **B** dorsal view **D** lateral view **F** ventral view. Scale bars: 1 mm. Species of Clade C, see Fig. 3.

**Figure 143.**
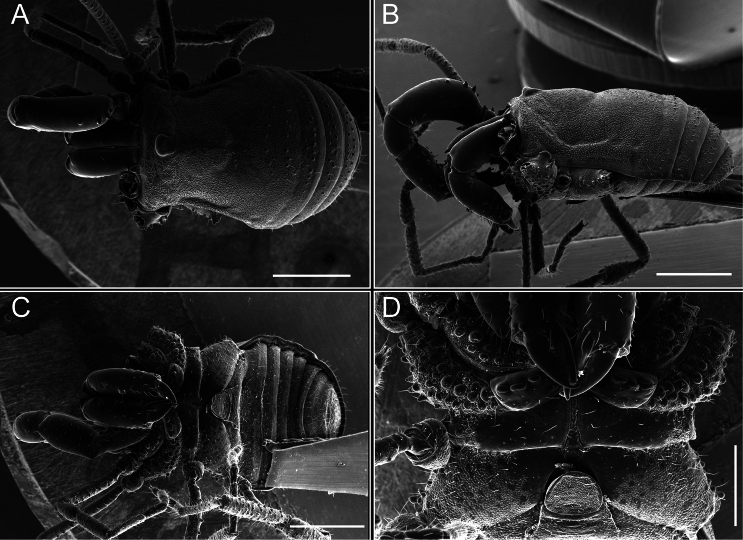
*Nerudiellavilches* sp. nov. male, SEM images of habitus **A** dorsal view **B** lateral view **C, D** ventral view. Scale bars: 1 mm (**A, B, C**); 500 µm (**D**).

**Figure 144.**
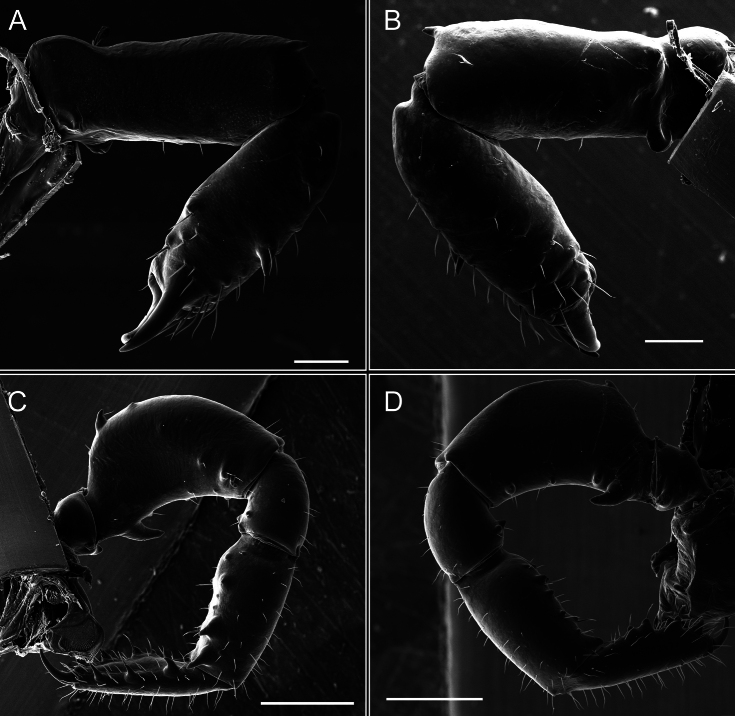
*Nerudiellavilches* sp. nov. chelicerae: mesal **A** ectal **B** pedipalps: mesal **C** ectal **D**. Scale bars: 200 µm (**A, B**); 500 µm (**C, D**).

**Figure 145.**
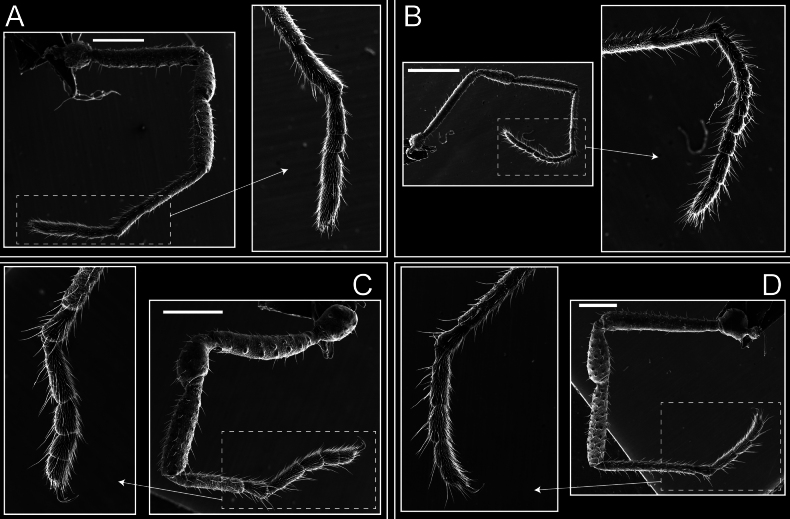
*Nerudiellavilches* sp. nov. legs I **A** II **B** III **C** IV **D**. Scale bars: 500 µm (**A, C, D**); 1 mm (**B**).

**Figure 146.**
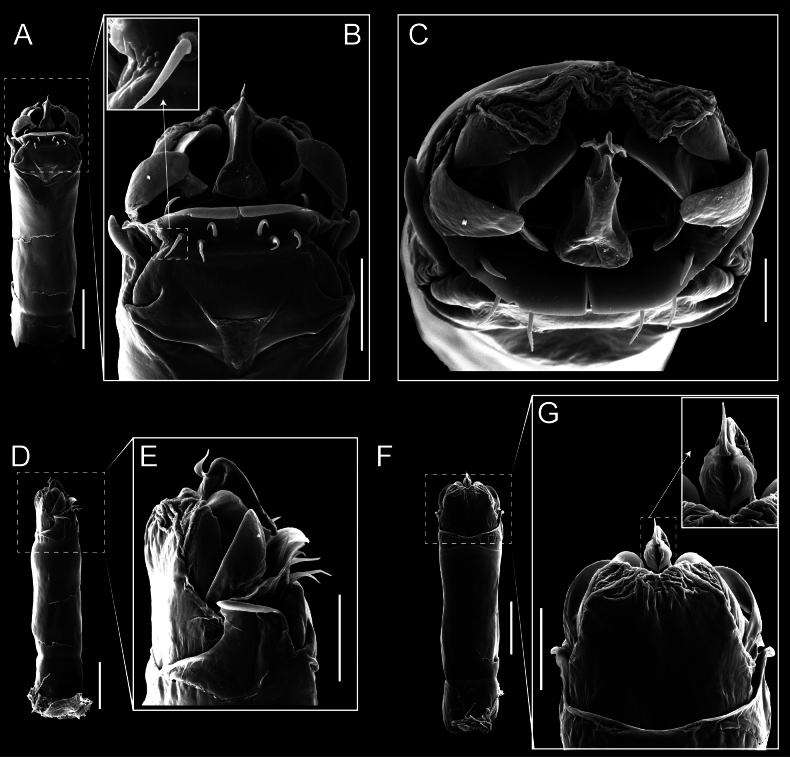
*Nerudiellavilches* sp. nov. penis: ventral **A, B** apical **C** lateral **D, E** dorsal **F, G**. Scale bars: 200 µm (**A, D, F**); 100 µm (**B, E, G**); 50 µm (**C**).

**Figure 147.**
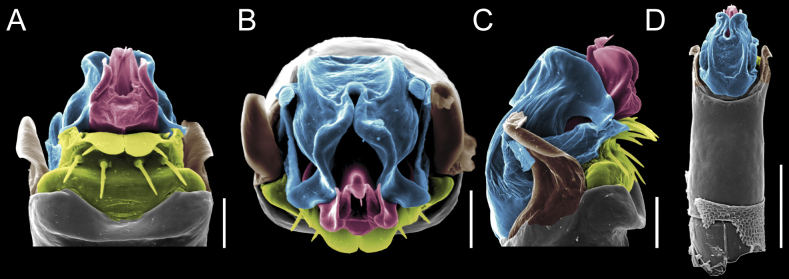
*Nerudiellavilches* sp. nov. penis: ventral **A** apical **B** lateral **C** dorsal **D**. Colors: ventral plate (yellow), capsula externa (blue), capsula interna (red). Scale bars: 100 µm (**A, C, D**); 50 µm (**B**).

Figs 142 , 143 , 144 , 145 , 146 , 147

###### Material examined.

***Holotype*.** ♂ **Chile.** Cachapoal: Río de los Cipreses National Reserve, Near Potrero, A. Ojanguren, A. Pérez-González, M. Ramírez, G. Azevedo, W. Porto coll., 09.I.2018 (MNHNCL). ***Paratypes*. Chile.** Cachapoal: Río de los Cipreses National Reserve, Near Potrero, A. Ojanguren, A. Pérez-González, M. Ramírez, G. Azevedo, W. Porto coll., 09.I.2018, 1 ♂ 2 ♀ 5 imm. (MACN). Osorno. Talca, Alto de Vilches, N. Platnick, K.Catley, M. Ramírez, T.Allen coll., 14–15/XI/1993, 1 ♂ 2 imm. (AMNH). Talca. Vilches, 132 km E. of Talca, E. Maury coll., 07–08.I.1989, 2 ♂ 1 ♀ (MACN). Alto de Vilches, 70 km E Talca, S.Peck, J.Peck coll., 05.XII.1985, 3 ♂ 3 ♀ 4 imm. (FMNH). Talca. Vilches, A.Roig coll., 17.I.1984, 2 ♂ 1 ♀ (MACN).

###### Additional material.

Chile. RN Altos del Lircay, E Vilches Alto, A. Ojanguren, A. Pérez-González, M. Ramírez, G. Azevedo, W. Porto coll. 11.I.2018 1 ♂ (MACN).

###### Etymology.

The specific epithet refers to the type locality of the species, Vilches, located in the commune of San Clemente, province of Talca, Chile. Noun in apposition.

###### Diagnosis.

This species can be easily distinguished from the other species in the genus by having a projecting process on the ventral femoral region of the pedipalp. The capsula externa of the genitalia does not have dorsal slits but bears a pair of lateral processes.

###### Distribution.

Chile: Maule Region (Fig. 4F).

###### Description of male holotype.

Measurements: Total length 4.12, carapace length 1.05, dorsal scutum length 2.42, carapace max. width 1.33, mesotergum max. width 1.83. Appendage measurements: Pedipalps. Trochanter length 0.24, femora length 0.94, patella length 0.47, tibia length 0.80, tarsus length 0.71. Leg I: trochanter (tr) 0.30, femora (fe) 0.20, patella (pa) 0.87, tibia (ti) 0.41, metatarsus (mt) 0.69, tarsus (ta) 0.76. II: tr 0.64, fe 0.30, pa 1.13, ti 0.52, mt 0.86, ta 0.94. III: tr 1.24, fe 0.29, pa 0.79, ti 0.32, mt 0.60, ta 0.62. IV: tr 0.68, fe 0.30, pa 1.16, ti 0.55, mt 0.92, ta 1.12, ta 0.74.

Dorsum (Fig. 142, 143). Eta (η) hourglass-shaped dorsal scutum. Low ocularium; dorsal scutum microgranulate, without clear delimitation of areas. Areas III–IV with a row of 6–7 small setiferous tubercles; posterior margin with a row of 12 small rounded setiferous tubercles. All free tergites have two rows of small setiferous tubercles.

Chelicerae (Fig. 144A, B). Segment I with a small tubercle on the dorso-distal surface. Segment II with a frontal tubercle and bearing few setae.

Pedipalps (Fig. 144C, D). Trochanter with a small ectal and dorsal tubercle. Femora bearing a forward-curved proximal ventral spine, a ventral row of three small setiferous granules, a dorsal tubercle with subdistal setae, and three distal setiferous granules. Patella with two ventral setiferous granules. Tibia with three ectal and two mesal spines with subdistal setae, with scant granules in ventral view. Tarsus with four ectal and three mesal spines with subdistal setae.

Legs (Fig. 145). Coxa I–II covered with rounded setiferous tubercles, the distal one is acute and has a subdistal seta, III and IV only with microgranulation. Spiracles not obstructed by bridges. Smooth area occupying 1/3 of leg II (with three tubercles), ¾ of III and 1/3 of IV. Sternum arrow-shaped. Legs I–IV covered in setae. Tibiae I–III with a ventral and dorsal row of small setiferous tubercles, IV with a row of four distoventral tubercles with setae. Calcaneus smaller than the astragalus, ≥ 3× smaller (leg I), 4× (II, III), and 5× (leg IV). Tarsal count: 3–6–4–4.

Penis (Figs 146, 147). Pars distalis with a ventral plate bearing a cleft dividing the plate into two lamellae. Each lamellae has three pointed macrosetae on the ventral surface and one macroseta on the dorsal surface; capsula externa covering dorsal and lateral surfaces, without cleft; it has a pair of lateral processes that are projected ventrally. The capsula interna bears a pair of laminar processes; the apical region has a small dorsal opening and a fine process.

**Female.** Similar to male, with shorter pedipalpal femora.

Female measurements. Total length 3.67, carapace length 0.93, dorsal scutum length 1.90, carapace max. width 1.16, mesotergum max. width 1.77. Appendage measurements: Pedipalps. Trochanter length 0.22, femora length 0.85, patella length 0.47, tibia length 0.68, tarsus length 0.59. Leg I: trochanter (tr) 0.19, femora (fe) 0.84, patella (pa) 0.46, tibia (ti) 0.63, metatarsus (mt) 0.77, tarsus (ta) 0.67. II: tr 0.27, fe 1.12, pa 0.51, ti 0.86, mt 0.97, ta 1.30. III: tr 0.27, fe 0.82, pa 0.35, ti 0.55, mt 0.65, ta 0.66. IV: tr 0.33, fe 1.20, pa 0.56, ti 0.91, mt 1.07, ta 0.82.

##### ﻿ Nerudiella wekufe sp. nov.

Taxon classificationAnimaliaOpilionesTriaenonychidae

E6609E94-F304-59ED-B6CA-97A2163EC6A6

https://zoobank.org/F57E3734-45AE-4AEF-AD70-1A3A27AA7CC7

**Figure 148.**
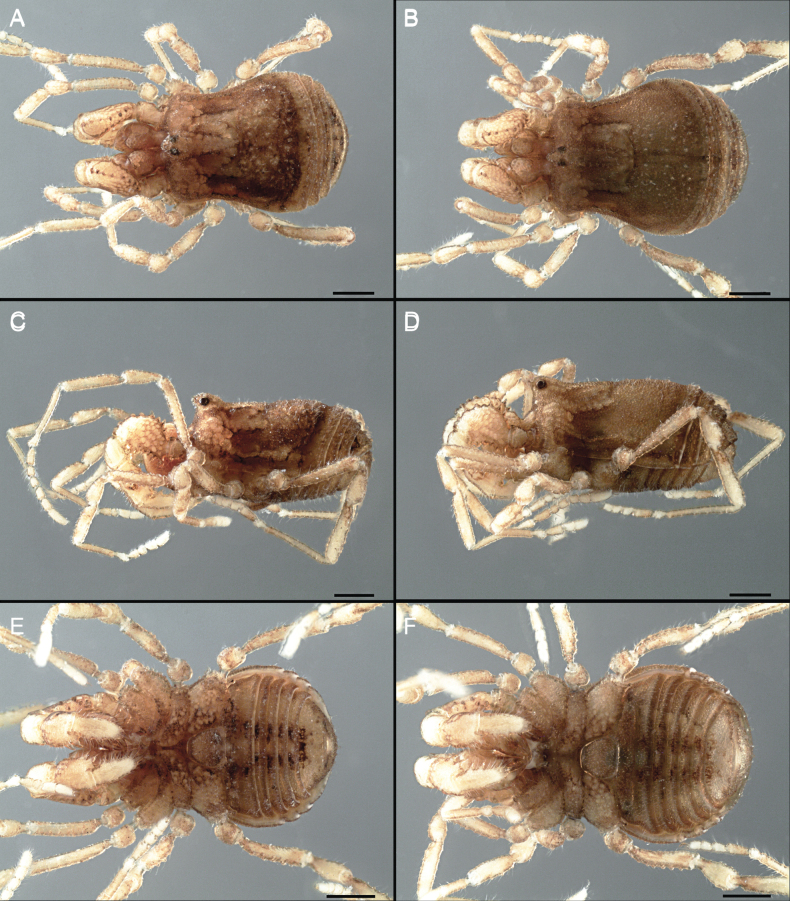
*Nerudiellawekufe* sp. nov. habitus, male **A** dorsal view **C** lateral view **E** ventral view. Female **B** dorsal view **D** lateral view **F** ventral view. Scale bars: 500 µm.

**Figure 149.**
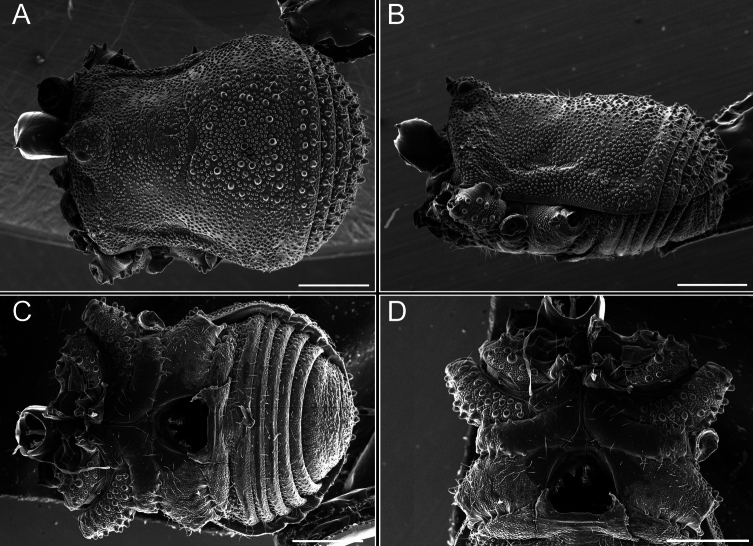
*Nerudiellawekufe* sp. nov. male, SEM images of habitus **A** dorsal view **B** lateral view **C, D** ventral view. Scale bars: 500 µm (**A, B, C**); 1 mm (**D**).

**Figure 150.**
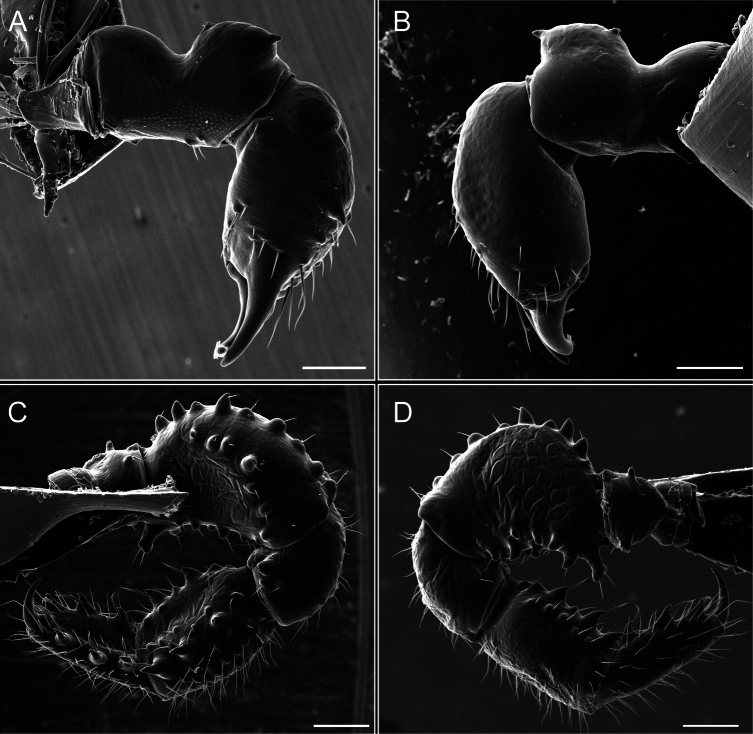
*Nerudiellawekufe* sp. nov. chelicerae: mesal **A** ectal **B** pedipalps: mesal **C** ectal **D**. Scale bars: 200 µm.

**Figure 151.**
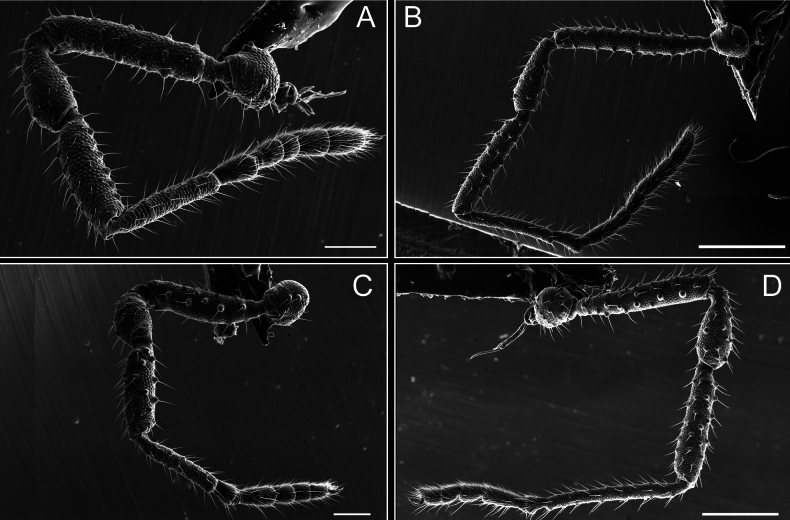
*Nerudiellawekufe* sp. nov. legs I **A** II **B** III **C** IV **D**. Scale bars: 200 µm (**A, C**); 500 µm (**B, D**).

**Figure 152.**
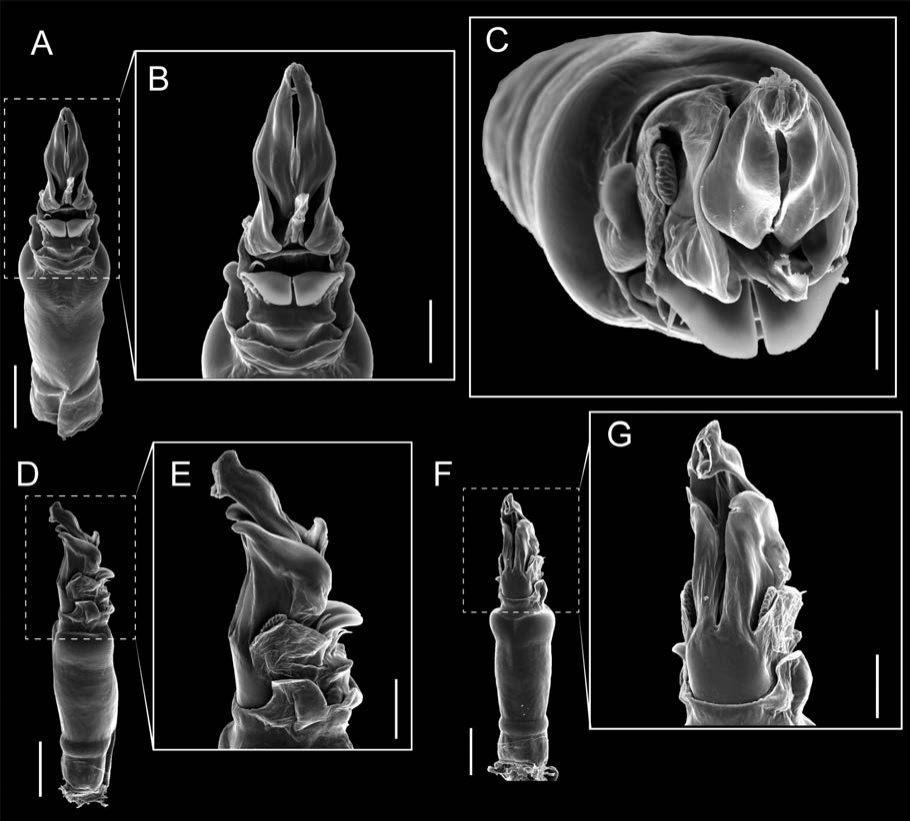
*Nerudiellawekufe* sp. nov. penis: ventral **A, B** apical **C** lateral **D, E** dorsal **F, G**. Scale bars: 200 µm (**A, D, F**); 100 µm (**B, E, G**); 50 µm (**C**).

**Figure 153.**
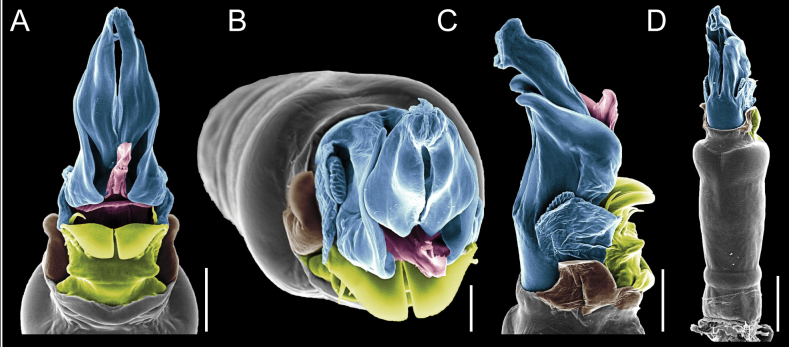
*Nerudiellawekufe* sp. nov. penis: ventral **A** apical **B** lateral **C** dorsal **D**. Colors: ventral plate (yellow), capsula externa (blue), capsula interna (red). Scale bars: 100 µm (**A, C**); 50 µm (**B**); 200 µm (**D**).

Figs 148 , 149 , 150 , 151 , 152 , 153

###### Material examined.

***Holotype*.** ♂ **Chile.** Concepción: El Manzano, T. Cekalovic coll. 31.III.1984 (MNHNCL). ***Paratypes*. Chile.** Concepción: El Manzano, T. Cekalovic coll., 03.I.1985, 1 ♂ (MACN). Same data, 03.I.1985, 1 ♀ (MACN). Same locality and collector, 31.III.1984, 1 ♂, 1 ♀, 2 imm. (MACN).

###### Etymology.

The specific epithet refers to the “Wekufe”, a spirit and/or harmful force (energy) from Mapuche belief and mythology. Noun in apposition.

###### Diagnosis.

This species can be distinguished from other species in the genus by the presence of an apophysis in the anterior portion of the ocularium. Additionally, in males, the genitalia exhibit a longer capsula externa compared to the capsula interna, providing a distinguishing characteristic.

###### Distribution.

Chile: Bío-Bío Region (Fig. 4F).

###### Description of male holotype.

Measurements: Total length 2.4, carapace length 1.0, dorsal scutum length 1.9, carapace max. width 1.4, mesotergum max. width 1.8. Appendage measurements: Pedipalps. Trochanter length 0.22, femora length 0.8, patella length 0.47, tibia length 0.57, tarsus length 0.76. Leg I: trochanter (tr) 0.25, femora (fe) 0.81, patella (pa) 0.40, tibia (ti) 0.62, metatarsus (mt) 0.73, tarsus (ta) 0.61. II: tr 0.26, fe 1.00, pa 0.54, ti 0.82, mt 0.89, ta 1.2. III: tr 0.28, fe 0.71, pa 0.37, ti 0.56, mt 0.69, ta 0.63. IV: tr 0.29, fe 0.98, pa 0.55, ti 0.85, mt 1.1, ta 0.68.

Dorsum (Fig. 148, 149). Eta (η) hourglass-shaped dorsal scutum. Ocularium low and rounded, with an anterior apophysis. Dorsal scutum and free tergites microgranulate. Although the areas of the dorsal scutum do not exhibit clear separation, they are covered with small setiferous tubercles, which are more prominent on the dorsal scutum and free tergites.

Chelicerae (Fig. 150A, B). Segment I with a sharp tubercle on the dorso-distal surface and three small ventral-proximal tubercles. Segment II with scattered setae in ectal and ventral views, with one triangular tubercle prominent from the others in front view.

Pedipalps (Fig. 150C, D). Trochanter with two small dorsal tubercles and a ventral one. Femora with two parallel rows of dorsal and spines. Patella with a mesal tubercle and small sparse tubercles. Tibia with three ventral-ectal and two ventral-mesal spines, lateral and dorsal areas with small setiferous tubercles. Tarsus with three mesal and ectal spines with subdistal setae in addition to setae and few setae.

Legs (Fig. 151). Coxa I with 12 or 13 setiferous tubercles the two apical ones are stronger than the others, II with 25–30 setiferous tubercles, III with seven or eight tubercles, IV with five or six small tubercles. Spiracles not obstructed by bridges. Smooth surface occupying 1/3 of leg II, ¾ of III and < 1/3 of IV. Sternum arrow-shaped. Legs covered in small tubercles, astragalus longer than calcaneus on all legs. Tarsal count: 4–6–4–4.

Penis (Figs 152, 153). Pars distalis with a ventral plate that is divided into two halves by a fine cleft. Each half of the ventral plate with three pointed macrosetae on the ventral surface and one macroseta on the dorsal surface. Capsula externa remarkably long, covering the dorsal surface. It is further divided into two halves by a long cleft and possesses a pair of long processes that curve ventrally. Additionally, with a dorsolateral plate attached to the pars basalis. Capsula externa longer than the capsula interna. Capsula interna thin and laterally flattened, with a sharp apical area.

**Female.** Similar to male, with shorter pedipalpal femora.

Female measurements. Measurements: Total length 2.7, carapace length 1.1, dorsal scutum length 2.1, carapace max. width 1.5, mesotergum max. width 2.0. Appendage measurements: Pedipalps. Trochanter length 0.23, femora length 0.7, patella length 0.47, tibia length 0.58, tarsus length 0.66, leg I: trochanter (tr) 0.25, femora (fe) 0.82, patella (pa) 0.41, tibia (ti) 0.54, metatarsus (mt) 0.74, tarsus (ta) 0.57. II: tr 0.23, fe 1.0, pa 148, ti 0.78, mt 0.83, ta 1.1. III: tr 0.28, fe 0.74, pa 0.37, ti 0.57, mt 0.73, ta 0.65. IV: tr 0.30, fe 0.97, pa 0.54, ti 0.85, mt 1.1, ta 0.7. Tarsal count: 3–6–4–4.

##### ﻿ Nerudiella zapallar sp. nov.

Taxon classificationAnimaliaOpilionesTriaenonychidae

7FFF130C-A549-5095-B640-5302BA364ED4

https://zoobank.org/DBDA77EA-D4D0-4AF3-BB05-9B3292CC24C7

**Figure 154.**
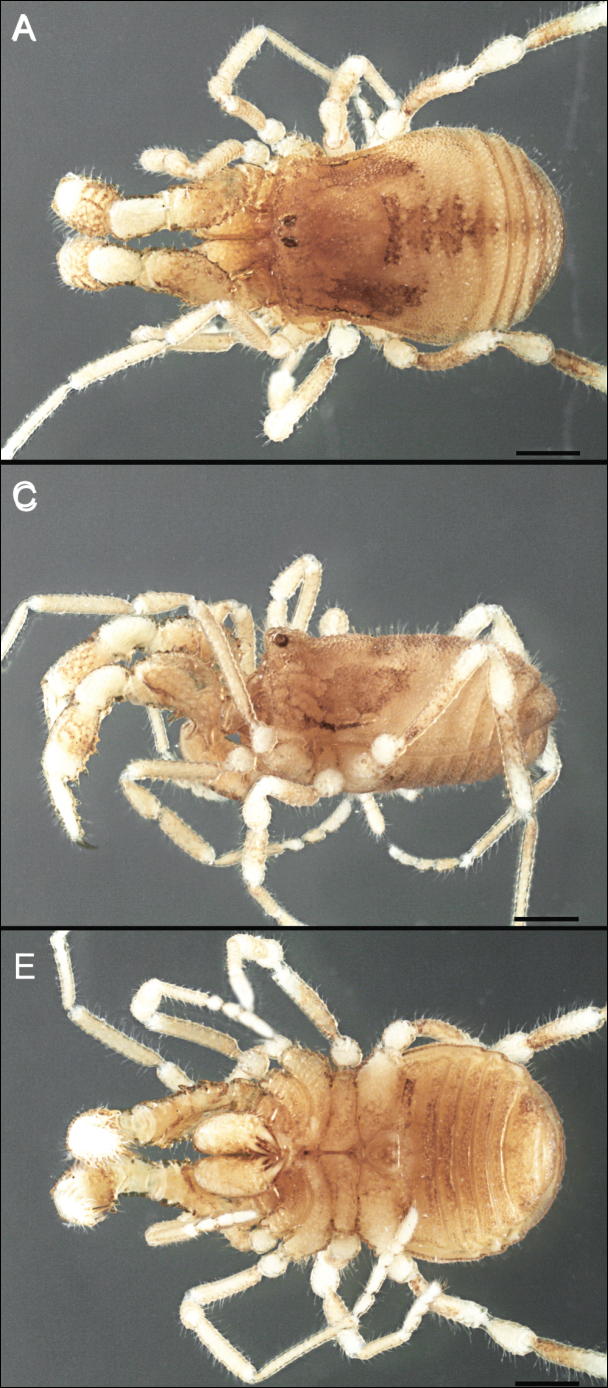
*Nerudiellazapallar* sp. nov. habitus, male **A** dorsal view **B** lateral view **C** ventral view. Scale bars: 1 mm.

**Figure 155.**
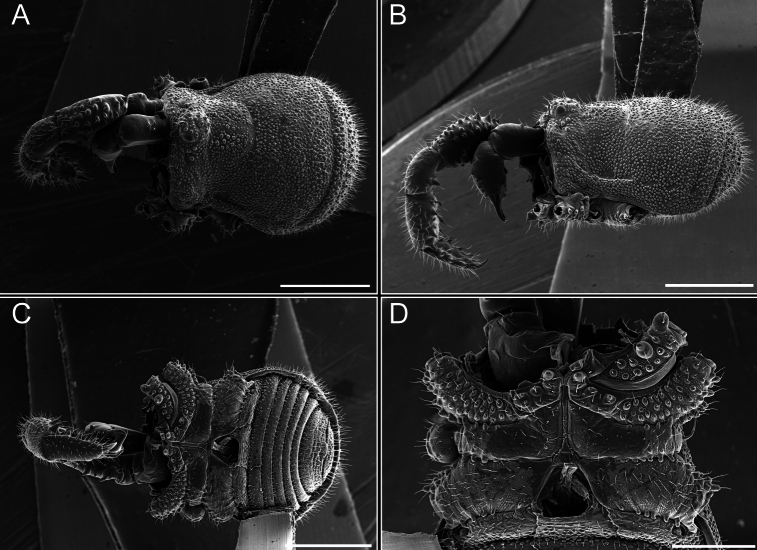
*Nerudiellazapallar* sp. nov. male, SEM images of habitus **A** dorsal view **B** lateral view **C, D** ventral view. Scale bars: 1 mm (**A, B, C**); 500 µm (**D**).

**Figure 156.**
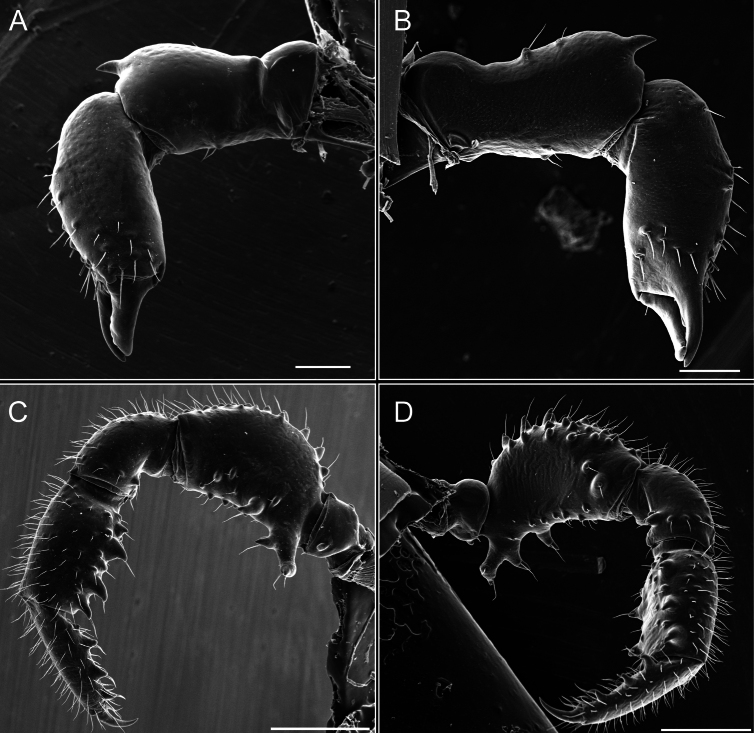
*Nerudiellazapallar* sp. nov. chelicerae: mesal **A** ectal **B** pedipalps: mesal **C** ectal **D**. Scale bars:200 µm.

**Figure 157.**
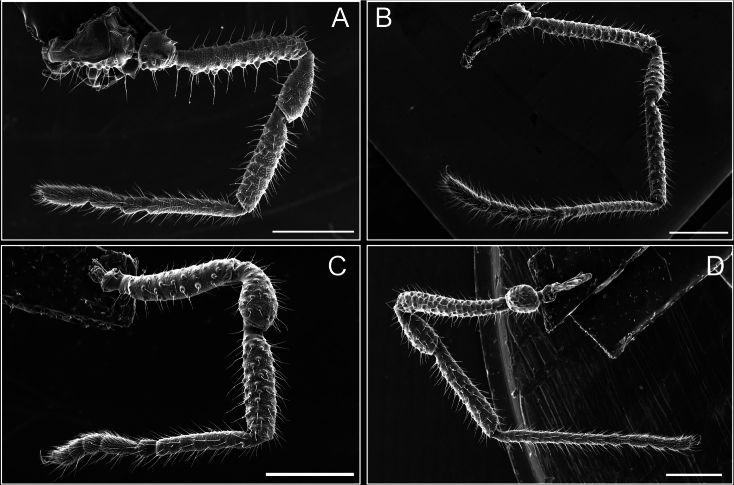
*Nerudiellazapallar* sp. nov. legs I **A** II **B** III **C** IV **D**. Scale bars: 200 µm (**A, C**); 500 µm (**B, D**).

**Figure 158.**
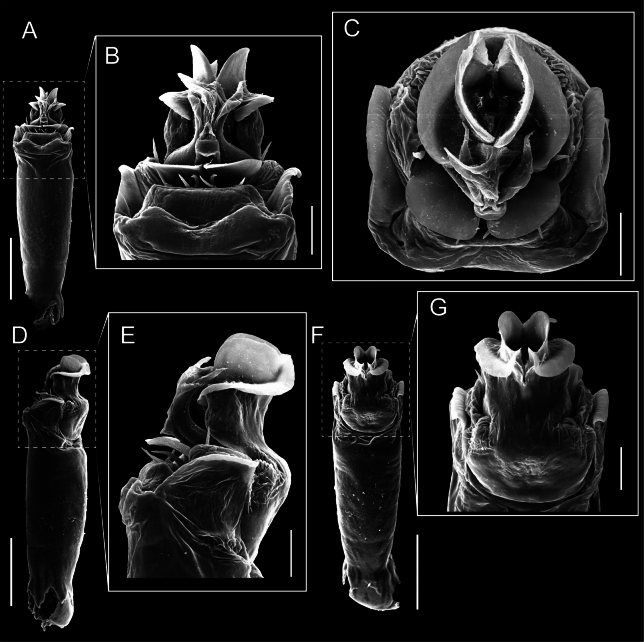
*Nerudiellazapallar* sp. nov. penis: ventral **A, B** apical **C** lateral **D, E** dorsal **F, G**. Scale bars: 200 µm (**A, D, F**); 50 µm (**B, C, E, G**).

**Figure 159.**
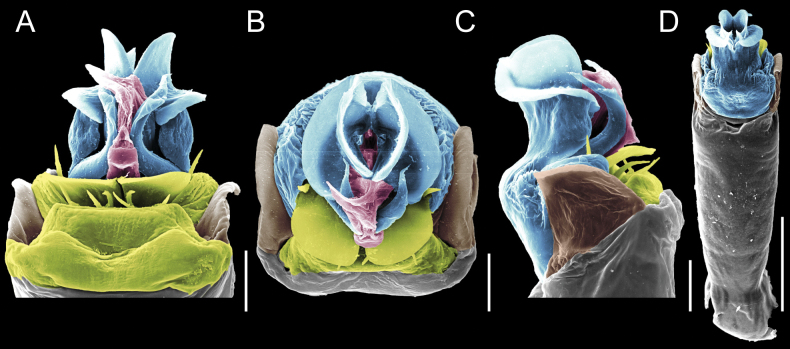
*Nerudiellazapallar* sp. nov. penis: ventral **A** apical **B** lateral **C** dorsal **D**. Colors: ventral plate (yellow), capsula externa (blue), capsula interna (red). Scale bars: 50 µm (**A–C**); 200 µm (**D**).

Figs 154 , 155 , 156 , 157 , 158 , 159

###### Material examined.

***Holotype*.** ♂ **Chile.** Zapallar: E. Ross, A. Michelbacher coll. 27.XI.1950 (CAS).

###### Etymology.

The specific epithet refers to the type locality of the species, the commune of Zapallar, located in the province of Petorca, Region of Valparaíso, Chile. Noun in apposition.

###### Diagnosis.

This species can be distinguished from other species in the genus by a unique combination of features. The dorsal surface and pedipalps exhibit a dense population of setae. Additionally, the femoras and tibiae of the pedipalps are covered with small tubercles. In terms of male genitalia, it features a capsula externa that envelops the dorsal and lateral surfaces. A notable characteristic is the apical region of the capsula externa, which bends at a 90-degree angle in relation to the genitalia’s axis. Moreover, two small parallel apical structures are also present. It is worth noting that this species shares some similarities with *Nerudiellachoapa* sp. nov., especially in the apical region of the capsula externa. However, it is relatively larger in comparison.

###### Distribution.

Chile: Valparaíso Region (Fig. 4F).

###### Description of male holotype.

Measurements: Total length 2.39. Carapace length 0.94, Dorsal scutum length 1.88, Carapace max. width 1.2, Dorsal scutum max. width 1.8. Appendage measurements. Pedipalps. Trochanter length 0.26, femora length 0.82, patella length 0.43, tibia length 0.65, tarsus length 0.76. Leg I: trochanter (tr) 0.24, femora (fe) 0.85, patella (pa) 0.44, tibia (ti) 0.62, metatarsus (mt) 0.73, tarsus (ta) 0.55. II: tr 0.25, fe 1.27, pa 0.52, ti 0.85, mt 0.85, ta 0.95. III: tr 0.25, fe 0.72, pa 0.35, ti 0.64, mt 0.72, ta 0.57. IV: tr 0.24, fe 1.08, pa 0.52, ti 0.93, mt 1.09, ta 0.68.

Dorsum. (Fig. 154, 155). Eta (η) hourglass-shaped dorsal scutum. Ocularium low, rounded, with small tubercles. Dorsal scutum and free tergites microgranulate, mesotergal areas without clear separation but covered with small setiferous tubercles.

Chelicerae (Fig. 156A, B). Segment I with a sharp tubercle on the dorso-distal surface and three small proximal tubercles. Segment II bearing scattered setae in ectal and ventral views, with one triangular tubercle prominent from the others in front view.

Pedipalps (Fig. 156C, D). Trochanter with a small dorsal tubercle. Femora bearing the dorso-mesal area with setiferous spines, there are three proximal ones that are stronger than the others; in ventral view there are three strong proximal spines, and a row of small tubercles. Patella with a row of setiferous tubercles. Tibia with three ventral-ectal and two ventral-mesal spines, lateral and dorsal areas with small setiferous tubercles. Tarsus with three mesal and ectal spines with subdistal setae.

Legs (Fig. 157). Coxa I with 11–13 setiferous tubercles the two apical ones are stronger than the others, II with 25–30 setiferous tubercles, III with nine or ten tubercles, IV with six or seven small tubercles. Spiracles not obstructed by bridges. Smooth surface occupying 1/3 of leg II, ¾ of III, and < 1/3 of IV. Smooth area of leg II with two small tubercles with subdistal setae on each side. Sternum arrow-shaped. Legs covered in small tubercles, astragalus longer than calcaneus on all legs. Tarsal count: 3–4/5–4–4.

Penis (Figs 158, 159). Pars distalis with a ventral plate bearing a fine cleft dividing the plate into two halves. Each half with three pointed macrosetae on the ventral surface and one macroseta on the dorsal surface; capsula externa covering the dorsal surface, with the apical part bent at an angle of 90 ° to the axis of the pars basalis of the genitalia, with a pair of long apical processes and a pair of long lateral processes; there is a dorsolateral plate attached to the pars basalis. Capsula externa taller than capsula interna. Capsula interna thin, with a sharp apical area.

**Female.** Unknown.

## Supplementary Material

XML Treatment for Fresiax

XML Treatment for Fresiax conica

XML Treatment for Fresiax fray

XML Treatment for Fresiax mauryi

XML Treatment for Fresiax pichicuy

XML Treatment for Fresiax spinulosa

XML Treatment for Mistralia

XML Treatment for Mistralia ramirezi

XML Treatment for Mistralia verrucosa

XML Treatment for Chilenuncia

XML Treatment for Chilenuncia chilensis

XML Treatment for Chilenuncia rostrata

XML Treatment for Laftrachia

XML Treatment for Laftrachia robin

XML Treatment for Lautaria

XML Treatment for Lautaria ceachei

XML Treatment for Nerudiella

XML Treatment for Nerudiella americana

XML Treatment for Nerudiella cachai

XML Treatment for Nerudiella caramavida

XML Treatment for Nerudiella cautin

XML Treatment for Nerudiella choapa

XML Treatment for Nerudiella curi

XML Treatment for Nerudiella goroi

XML Treatment for Nerudiella jaimei

XML Treatment for Nerudiella malleco

XML Treatment for Nerudiella penco

XML Treatment for Nerudiella pichi

XML Treatment for Nerudiella portai

XML Treatment for Nerudiella quenes

XML Treatment for Nerudiella vilches

XML Treatment for Nerudiella wekufe

XML Treatment for Nerudiella zapallar

## ﻿Additional information

### Author ORCIDs

Willians Porto https://orcid.org/0000-0002-4209-5664

Shahan Derkarabetian https://orcid.org/0000-0002-9163-9277

Gonzalo Giribet https://orcid.org/0000-0002-5467-8429

Abel Pérez-González https://orcid.org/0000-0002-4245-3302

### Data availability

All of the data that support the findings of this study are available in the main text or Supplementary Information.

## Supplementary materials

Supplementary material 1 Collection information and GenBank accession numbers Data type docx Explanation note Collection information and GenBank accession numbers for specimens used in this study (focus group in red). https://binary.pensoft.net/file/1090773This dataset is made available under the Open Database License (http://opendatacommons.org/licenses/odbl/1.0/). The Open Database License (ODbL) is a license agreement intended to allow users to freely share, modify, and use this Dataset while maintaining this same freedom for others, provided that the original source and author(s) are credited.Willians Porto, Shahan Derkarabetian, Gonzalo Giribet, Abel Pérez-González

Supplementary material 2 Complete IQ-TREE results Data type jpg Explanation note Results obtained from IQ-TREE, with the SH-aLRT and UFBoot supports indicated at the nodes. The red branches in the illustration represents South American species. https://binary.pensoft.net/file/1090774This dataset is made available under the Open Database License (http://opendatacommons.org/licenses/odbl/1.0/). The Open Database License (ODbL) is a license agreement intended to allow users to freely share, modify, and use this Dataset while maintaining this same freedom for others, provided that the original source and author(s) are credited.Willians Porto, Shahan Derkarabetian, Gonzalo Giribet, Abel Pérez-González
