# Supplementary material for: ﻿Systematic revision of the South American “Nuncia” (Opiliones, Laniatores, Triaenonychidae)
Source: Zookeys. 2024 Jul 12;1207:1–149. doi: 10.3897/zookeys.1207.120068 (PMC11273004; doi:10.3897/zookeys.1207.120068)
Supplement: Supplementary material 1 — Collection information and GenBank accession numbers [file zookeys-1207-001_article-120068__-s001.docx]

Supplementary table 1. Collection information and GenBank accession numbers for specimens used in this study (focus group in red).

| **Species** | **Family** | **Voucher** | **UCE** |
| --- | --- | --- | --- |
| *Abaddon despoliator* | Lomanellidae | MCZ:IZ:132893 | *Derkarabetian *et al*. (2021) |
| *Acumontia* sp. | Triaenonychidae | SDSU_TAC:OP4295 | *Derkarabetian *et al*. (2018) |
| *Adaelum areolatum* | Triaenonychidae | MCZ30072_1 | *Baker *et al*. (2020) |
| *Adaelum humifer* | Triaenonychidae | MCZ59004 | *Baker *et al*. (2020) |
| *Adaeulum bicolor* | Triaenonychidae | MCZ126991_2 | *Baker *et al*. (2020) |
| *Adaeulum godfreyi* | Triaenonychidae | MCZ138019 | *Baker *et al*. (2020) |
| *Adaeulum moruliferum* | Triaenonychidae | MCZ138016 | *Baker *et al*. (2020) |
| *Adaeulum robustum* | Triaenonychidae | MCZ132883 | *Baker *et al*. (2020) |
| *Adaeulum* sp. | Triaenonychidae | MCZ:IZ:138019 | *Derkarabetian *et al*. (2021) |
| *Adaeum* sp. | Triaenonychidae | MCZ73548_2 | *Baker *et al*. (2020) |
| *Adrianonyx contulmo* | Triaenonychidae | MCZ:IZ:138057 | *Derkarabetian *et al*. (2021) |
| *Algidia* cf *nigriflava* | Triaenonychidae | MCZ136202 | *Baker *et al*. (2020) |
| *Algidia chiltoni* | Triaenonychidae | MCZ136192 | *Baker *et al*. (2020) |
| *Algidia cuspidata multispinosa* | Triaenonychidae | MCZ136151 | *Baker *et al*. (2020) |
| *Algidia nigriflava* | Triaenonychidae | MCZ136171 | *Baker *et al*. (2020) |
| *Algidia* sp. | Triaenonychidae | MCZ:IZ:133223 | *Derkarabetian *et al*. (2021) |
| *Allobunus distinctus* | Triaenonychidae | TAS167 | *Derkarabetian *et al*. (2021) |
| *Allonuncia grandis* | Triaenonychidae | TAS154 | *Derkarabetian *et al*. (2021) |
| *Amatola* (*Cryptobunus*) sp. | Triaenonychidae | MCZ:IZ:127007 | *Derkarabetian *et al*. (2021) |
| *Amatola dentifrons* | Triaenonychidae | SDSU_TAC:OP4606 | *Derkarabetian *et al*. (2021) |
| *Amatola* sp. | Triaenonychidae | MCZ132881 | *Baker *et al*. (2020) |
| *Americobunus ringueleti* | Triaenonychidae | SDSU_TAC:OP4299 | *Derkarabetian *et al*. (2018) |
| *Ankaratrix illota* | Triaenonychidae | SDSU_TAC:OP4300 | *Derkarabetian *et al*. (2018) |
| *Ankylonuncia mestoni* | Triaenonychidae | TAS029 | *Derkarabetian *et al*. (2021) |
| *Antongila spinigera* | Triaenonychidae | SDSU_TAC:OP4607 | *Derkarabetian *et al*. (2021) |
| *Araucanobunus juberthiei* | Triaenonychidae | MCZ:IZ:152638 | *Derkarabetian *et al*. (2021) |
| *Austromontia* sp. | Triaenonychidae | MCZ73484_3 | *Baker *et al*. (2020) |
| *Austromontia* sp. | Triaenonychidae | MCZ134926 | *Baker *et al*. (2020) |
| *Austromontia* sp. | Triaenonychidae | MCZ:IZ:73525 | *Derkarabetian *et al*. (2021) |
| *Biacumontia* sp. | Triaenonychidae | MCZ:IZ:73478 | *Derkarabetian *et al*. (2019) |
| *Bishopella laciniosa* | Grassatores: Phalangodidae | SDSU_TAC:OP569 | *Starrett *et al*. (2017) |
| *Breviacantha gisleni* (imm) | Triaenonychidae | MCZ:IZ:132906 | *Derkarabetian *et al*. (2021) |
| *Briggsus pacificus* | Travunioidea: Cladonychiidae | SDSU_TAC:OP3625 | *Starrett *et al*. (2017) |
| *Bryonuncia distincta* | Triaenonychidae | TAS115 | *Derkarabetian *et al*. (2021) |
| *Callihamina* sp. | Triaenonychidae | ZMUC 18A | *Derkarabetian *et al*. (2019) |
| *Callihamus* sp. | Triaenonychidae | MCZ:IZ:152666 | *Derkarabetian *et al*. (2021) |
| *Calliuncus* cf. *labrynthus* | Triaenonychidae | MCZ99220 | *Baker *et al*. (2020) |
| *Calliuncus* cf. *odoratus* | Triaenonychidae | SDSU_TAC:OP2778 | *Derkarabetian *et al*. (2021) |
| *Calliuncus odoratus* | Triaenonychidae | TAS229 | *Derkarabetian *et al*. (2019) |
| *Calliuncus* sp. | Triaenonychidae | MCZ:IZ:132901 | *Derkarabetian *et al*. (2021) |
| *Triaenonyx* | Triaenonychidae | OPI241 | - |
| *Triaenonyx* | Triaenonychidae | OPI216 | - |
| *Triaenonyx* | Triaenonychidae | OPI226 | - |
| *Triaenonyx* | Triaenonychidae | OPI215 | - |
| *Triaenonyx* | Triaenonychidae | OPI228 | - |
| *Triaenonyx* | Triaenonychidae | MCZ138029 | - |
| *Triaenonyx* | Triaenonychidae | CHITR039-18 | - |
| *Triaenonyx* | Triaenonychidae | MCZ138132 | *Baker *et al*. (2020) |
| *Triaenonyx* | Triaenonychidae | SDSU_TAC:OP4610 | *Derkarabetian *et al*. (2021) |
| *Cenefia* cf. *adaeiformis* | Triaenonychidae | MCZ:IZ:135771 | *Derkarabetian *et al*. (2019) |
| *Cenefia westlandica* | Triaenonychidae | MCZ:IZ:152329 | *Derkarabetian *et al*. (2021) |
| *Ceratomontia* sp. | Triaenonychidae | MCZ30065_2 | *Baker *et al*. (2020) |
| *Ceratomontia* sp. | Triaenonychidae | SDSU_TAC:OP4296 | *Derkarabetian *et al*. (2018) |
| *Chilenuncia chilensis* **comb.nov.** | Triaenonychidae | OPI194 | - |
| *Chilenuncia chilensis* **comb.nov.** | Triaenonychidae | OPI256 | - |
| *Chilenuncia chilensis* **comb.nov.** | Triaenonychidae | SDSU_TAC:OP4297 | *Derkarabetian *et al*. (2018) |
| *Chilenuncia chilensis* **comb.nov.** | Triaenonychidae | MCZ138133 | *Baker *et al*. (2020) |
| *Chrestobunus fuscus* | Triaenonychidae | TAS074 | *Derkarabetian *et al*. (2021) |
| *Cluniella distincta* | Triaenonychidae | MCZ:IZ:150624 | *Derkarabetian *et al*. (2021) |
| *Cluniella* sp. | Triaenonychidae | MCZ:IZ:23247 | *Derkarabetian *et al*. (2021) |
| *Cryptomaster leviathan* | Travunioidea: Cryptomastridae | SDSU_TAC:OP3794 | *Derkarabetian *et al*. (2018) |
| *Decarynella gracilipes* | Triaenonychidae | CASENT9040838 | *Derkarabetian *et al*. (2021) |
| *Diaenobunus armatus* | Triaenonychidae | MCZ:IZ:151609_2 | *Derkarabetian *et al*. (2021) |
| *Diaenobunus* sp. | Triaenonychidae | MCZ151611_2 | *Baker *et al*. (2020) |
| *Diasia michaelsenii* | Triaenonychidae | OPI019 | - |
| *Diasia michaelsenii* | Triaenonychidae | MCZ:IZ:138126 | *Derkarabetian *et al*. (2019) |
| *Dingupa glauerti* | Triaenonychidae | MCZ:IZ:132874 | *Derkarabetian *et al*. (2021) |
| *Equitius montanus* | Triaenonychidae | MCZ:IZ:133934 | *Derkarabetian *et al*. (2019) |
| *Erebomaster acanthinus* | Travunioidea: Cladonychiidae | SDSU_TAC:OP1603 | *Derkarabetian *et al*. (2018) |
| *Flavonuncia* sp. | Buemarinoidae | MCZ:IZ:23002 | *Derkarabetian *et al*. (2021) |
| *Fresiax conica* **sp.nov.** | Triaenonychidae | OPI257 | - |
| *Fresiax mauryi* **sp.nov.** | Triaenonychidae | OPI249 | - |
| *Fresiax pichicuya* **sp.nov.** | Triaenonychidae | OPI224 | - |
| *Fresiax spinulosa* **comb.nov.** | Triaenonychidae | OPI195 | - |
| *Fresiax spinulosa* **comb.nov.** | Triaenonychidae | OPI650 | - |
| *Fumontana deprehendor* | Buemarinoidae | SDSU_TAC:OP623 | *Starrett *et al*. (2017) |
| *Fumontana deprehendor* | Buemarinoidae | MCZ46881 | *Baker *et al*. (2020) |
| *Fumontana deprehendor* | Buemarinoidae | MCZ134565 | *Baker *et al*. (2020) |
| *Glyptobunus ornatus* | Triaenonychidae | WSU10008 | *Baker *et al*. (2020) |
| *Glyptobunus ornatus* | Triaenonychidae | WSU10077 | *Baker *et al*. (2020) |
| *Glyptobunus signatus* | Triaenonychidae | TAS132 | *Derkarabetian *et al*. (2021) |
| *Glyptobunus signatus* | Triaenonychidae | WSU10044 | *Baker *et al*. (2020) |
| *Glyptobunus signatus* | Triaenonychidae | WSU10049 | *Baker *et al*. (2020) |
| *Graemontia* sp. | Triaenonychidae | MCZ73506_2 | *Baker *et al*. (2020) |
| *Graemontia* sp. | Triaenonychidae | MCZ:IZ:73486 | *Derkarabetian *et al*. (2021) |
| *Hadziani clavigera* | Travunioidea: Cladonychiidae | SDSU_TAC:OP2770 | *Derkarabetian *et al*. (2018) |
| *Hedwiga manubriata* | Triaenonychidae | MCZ:IZ:136129 | *Derkarabetian *et al*. (2021) |
| *Hendea bucculenta* | Triaenonychidae | MCZ136167 | *Baker *et al*. (2020) |
| *Hendea maitaia* | Triaenonychidae | MCZ135991 | *Baker *et al*. (2020) |
| *Hendea myersi* | Triaenonychidae | MCZ:IZ:133379 | *Derkarabetian *et al*. (2021) |
| *Hendea myersi* | Triaenonychidae | MCZ135987 | *Baker *et al*. (2020) |
| *Hendea oconnori* | Triaenonychidae | SDSU_TAC:OP4612 | *Derkarabetian *et al*. (2021) |
| *Hendea phillippsi* | Triaenonychidae | MCZ136180 | *Baker *et al*. (2020) |
| *Hendeola* sp. | Triaenonychidae | MCZ:IZ:149488 | *Derkarabetian *et al*. (2021) |
| *Heteronuncia robusta* | Triaenonychidae | MCZ:IZ:35919 | *Derkarabetian *et al*. (2019) |
| *Hickmanoxyomma cavaticum* | Triaenonychidae | TAS081 | *Derkarabetian *et al*. (2019) |
| *Hickmanoxyomma tasmanicum* | Triaenonychidae | TAS394 | *Derkarabetian *et al*. (2021) |
| *Hinzuanius* sp. | Grassatores: Biantidae | SDSU_TAC:OP4301 | *Derkarabetian *et al*. (2018) |
| *Holonuncia* sp. | Triaenonychidae | MCZ134219 | *Baker *et al*. (2020) |
| *Holonuncia* sp. | Triaenonychidae | MCZ:IZ:23313 | *Derkarabetian *et al*. (2021) |
| *Holoscotolemon unicolor* | Travunioidea: Cladonychiidae | SDSU_TAC:OP2771 | *Derkarabetian *et al*. (2018) |
| *Hovanuncia* sp. | Triaenonychidae | CASENT9040838 | *Derkarabetian *et al*. (2019) |
| *Isolachus spinosus* | Travunioidea: Cladonychiidae | SDSU_TAC:OP3630 | *Derkarabetian *et al*. (2018) |
| *Izunonychus ohruii* | Travunioidea: Paranonychidae | SDSU_TAC:OP2158 | *Derkarabetian *et al*. (2018) |
| *Kaolinonychus coreanus* | Travunioidea: Paranonychidae | SDSU_TAC:OP4291 | *Derkarabetian *et al*. (2018) |
| *Karamea lobata* | Triaenonychidae | MCZ136012 | *Baker *et al*. (2020) |
| *Karamea lobata australis* | Triaenonychidae | MCZ136031 | *Baker *et al*. (2020) |
| *Karamea tricerata* | Triaenonychidae | MCZ:IZ:64653_1 | *Derkarabetian *et al*. (2021) |
| *Karamea tricerata* | Triaenonychidae | MCZ135507 | *Baker *et al*. (2020) |
| *Karamea tuthilli* | Triaenonychidae | MCZ:IZ:29556_3 | *Derkarabetian *et al*. (2021) |
| *Triaenonyx* sp. | Triaenonychidae | OPI218 | - |
| *Triaenonyx* sp. | Triaenonychidae | OPI220 | - |
| *Tryaenonyx* sp. | Triaenonychidae | MCZ138140 | *Baker *et al*. (2020) |
| *Laftrachia robin* **sp.nov.** | Triaenonychidae | OPI017 | - |
| *Larifuga capensis* | Triaenonychidae | MCZ133938 | *Baker *et al*. (2020) |
| *Larifuga* sp. | Triaenonychidae | MCZ98386 | *Baker *et al*. (2020) |
| *Larifuga* sp. | Triaenonychidae | MCZ132909_2 | *Baker *et al*. (2020) |
| *Larifuga* sp. | Triaenonychidae | MCZ:IZ:49524 | *Derkarabetian *et al*. (2021) |
| *Larifugella* cf. *afra* | Triaenonychidae | MCZ132913 | *Baker *et al*. (2020) |
| *Larifugella* sp. | Triaenonychidae | MCZ:IZ:132916 | *Derkarabetian *et al*. (2021) |
| *Larifugella zuluana* | Triaenonychidae | MCZ98906_5 | *Baker *et al*. (2020) |
| *Lautaria ceachei* **sp.nov.** | Triaenonychidae | OPI233 | - |
| *Leiobunum calcar* | Eupnoi | SDSU_TAC:OP1089 | *Starrett *et al*. (2017) |
| *Lizamontia* sp. | Triaenonychidae | MCZ132885 | *Baker *et al*. (2020) |
| *Lizamontia* sp. | Triaenonychidae | MCZ:IZ:88577 | *Derkarabetian *et al*. (2021) |
| *Lomanella raniceps* | Lomanellidae | TAS071 | *Derkarabetian *et al*. (2021) |
| *Lomanella raniceps* | Lomanellidae | WSU10046 | *Baker *et al*. (2020) |
| *Lomanella troglophilia* | Lomanellidae | TAS212 | *Derkarabetian *et al*. (2021) |
| *Ceratomontia argentina* | Triaenonychidae | MCZ:IZ:152640 | *Derkarabetian *et al*. (2021) |
| *Ceratomontia argentina* | Triaenonychidae | OPINE1 | - |
| *Mensamontia* sp. | Triaenonychidae | MCZ127856 | *Baker *et al*. (2020) |
| *Mestonia acris* | Triaenonychidae | TAS063 | *Derkarabetian *et al*. (2021) |
| *Metanippononychus* sp. | Travunioidea: Paranonychidae | SDSU_TAC:OP4076 | *Derkarabetian *et al*. (2018) |
| *Micromontia flava* | Triaenonychidae | MCZ:IZ:88285 | *Derkarabetian *et al*. (2021) |
| Micromontia sp. | Triaenonychidae | MCZ73542_2 | *Baker *et al*. (2020) |
| *Miobunus thoracicus* | Triaenonychidae | TAS322 | *Derkarabetian *et al*. (2021) |
| *Mistralia verrucosa* **comb.nov.** | Triaenonychidae | OPI191 | - |
| *Mistralia verrucosa* **comb.nov.** | Triaenonychidae | MCZ:IZ:138122 | *Derkarabetian *et al*. (2021) |
| *Monomontia* sp. | Triaenonychidae | MCZ73250 | *Baker *et al*. (2020) |
| *Monomontia* sp. | Triaenonychidae | MCZ:IZ:58975 | *Derkarabetian *et al*. (2021) |
| *Monxyomma* cf. *manicatum* | Triaenonychidae | ZMUC | *Baker *et al*. (2020) |
| *Monxyomma* sp. | Triaenonychidae | MCZ152643 | *Baker *et al*. (2020) |
| *Nahuelonyx* sp. | Triaenonychidae | OPI015 | - |
| *Nahuelonyx* sp. | Triaenonychidae | SDSU_TAC:OP4611 | *Derkarabetian *et al*. (2019) |
| *Nahuelonyx* sp. | Triaenonychidae | OPI014 | - |
| *Nahuelonyx* sp. | Triaenonychidae | MCZ138056 |  |
| *Nahuelonyx* sp. | Triaenonychidae | CASENT9027532 | *Derkarabetian *et al*. (2021) |
| *Nerudiella americana* **comb.nov.** | Triaenonychidae | OPI206 | - |
| *Nerudiella americana* **comb.nov.** | Triaenonychidae | OPI198 | - |
| *Nerudiella americana* **comb.nov.** | Triaenonychidae | OPI204 | - |
| *Nerudiella cachai* **sp.nov.** | Triaenonychidae | OPI205 | - |
| *Nerudiella caramavida* **sp.nov.** | Triaenonychidae | OPI252 | - |
| *Nerudiella malleco* **sp.nov.** | Triaenonychidae | OPI203 | - |
| *Nerudiella portai* **sp.nov.** | Triaenonychidae | OPI207 | - |
| *Nerudiella portai* **sp.nov.** | Triaenonychidae | OPI225 | - |
| *Nerudiella quenes* **sp.nov.** | Triaenonychidae | OPI259 | - |
| *Nerudiella* sp. | Triaenonychidae | MCZ138091 |  |
| *Nerudiella* sp. | Triaenonychidae | OPI199 | - |
| *Nerudiella* sp. | Triaenonychidae | SDSU_TAC:OP4609 | *Derkarabetian *et al*. (2021) |
| *Nerudiella vilches* **sp.nov.** | Triaenonychidae | OPI230 | - |
| new genus B Australia | Triaenonychidae | ZMUC Aus025b DNA106091 | *Derkarabetian *et al*. (2019) |
| new genus B Australia | Triaenonychidae | ZMUC Aus025b 32A | *Derkarabetian *et al*. (2019) |
| *Nippononychus japonicus* | Travunioidea: Paranonychidae | SDSU_TAC:OP4079 | *Derkarabetian *et al*. (2018) |
| *Notonuncia diversa* | Triaenonychidae | TAS059 | *Derkarabetian *et al*. (2021) |
| *Nucina silvestris* | Triaenonychidae | TAS263 | *Derkarabetian *et al*. (2021) |
| *Nuncia arcuata aorangiensis* | Triaenonychidae | MCZ136113 | *Baker *et al*. (2020) |
| *Nuncia coriacea* | Triaenonychidae | MCZ58942 | *Baker *et al*. (2020) |
| *Nuncia coriacea* | Triaenonychidae | MCZ135438 | *Baker *et al*. (2020) |
| *Nuncia coriacea* | Triaenonychidae | MCZ136043 | *Baker *et al*. (2020) |
| *Nuncia coriacea cockaini* | Triaenonychidae | MCZ136038 | *Baker *et al*. (2020) |
| *Nuncia dentifera* | Triaenonychidae | MCZ135460 | *Baker *et al*. (2020) |
| *Nuncia heteromorpha* | Triaenonychidae | MCZ136049 | *Baker *et al*. (2020) |
| *Nuncia levis* | Triaenonychidae | MCZ136022 | *Baker *et al*. (2020) |
| *Nuncia nigriflava parva* | Triaenonychidae | MCZ135989 | *Baker *et al*. (2020) |
| *Nuncia obesa* | Triaenonychidae | MCZ135975 | *Baker *et al*. (2020) |
| *Nuncia obesa grimmetti* | Triaenonychidae | MCZ135970 | *Baker *et al*. (2020) |
| *Nuncia obesa magna* | Triaenonychidae | MCZ136076 | *Baker *et al*. (2020) |
| *Nuncia obesa rotunda* | Triaenonychidae | MCZ135990 | *Baker *et al*. (2020) |
| *Nuncia oconnori paucispinosa* | Triaenonychidae | MCZ135469 | *Baker *et al*. (2020) |
| *Nuncia pallida* | Triaenonychidae | MCZ136099 | *Baker *et al*. (2020) |
| *Nuncia roeweri demissa* | Triaenonychidae | MCZ135439 | *Baker *et al*. (2020) |
| *Nuncia roeweri gelida* | Triaenonychidae | MCZ135468 | *Baker *et al*. (2020) |
| *Nuncia roeweri humilis* | Triaenonychidae | MCZ135463 | *Baker *et al*. (2020) |
| *Nuncia roeweri seditiosa* | Triaenonychidae | MCZ136112 | *Baker *et al*. (2020) |
| *Nuncia smithi* | Triaenonychidae | MCZ135436 | *Baker *et al*. (2020) |
| *Nuncia* sp. | Triaenonychidae | MCZ:IZ:58597 | *Derkarabetian *et al*. (2021) |
| *Nuncia* sp. | Triaenonychidae | SDSU_TAC:OP4298 | *Derkarabetian *et al*. (2018) |
| *Nuncia stewartia* | Triaenonychidae | MCZ136086 | *Baker *et al*. (2020) |
| *Nuncia stewartia tumosa* | Triaenonychidae | MCZ136088 | *Baker *et al*. (2020) |
| *Nuncia sublaevis* | Triaenonychidae | MCZ135481 | *Baker *et al*. (2020) |
| *Nuncia tumula* | Triaenonychidae | MCZ135999 | *Baker *et al*. (2020) |
| *Nuncia variegata delli* | Triaenonychidae | MCZ135985 | *Baker *et al*. (2020) |
| *Nuncia variegata granulata* | Triaenonychidae | MCZ149353 | *Baker *et al*. (2020) |
| *Nunciella aspera* | Triaenonychidae | MCZ:IZ:132908 | *Derkarabetian *et al*. (2021) |
| *Nunciella cheliplus* | Triaenonychidae | SDSU_TAC:OP2780 | *Derkarabetian *et al*. (2021) |
| *Nunciella kangarooensis* | Triaenonychidae | MCZ:IZ:152566 | *Derkarabetian *et al*. (2021) |
| *Nunciella karriensis* | Triaenonychidae | MCZ132904 | *Baker *et al*. (2020) |
| *Nunciella* sp. | Triaenonychidae | MCZ132908 | *Baker *et al*. (2020) |
| *Nunciella tasmaniensis* | Triaenonychidae | TAS344 | *Derkarabetian *et al*. (2021) |
| *Nunciella tasmaniensis* | Triaenonychidae | WSU10027 | *Baker *et al*. (2020) |
| *Nunciella tasmaniensis* | Triaenonychidae | WSU10009 | *Baker *et al*. (2020) |
| *Nuncioides infrequens* | Triaenonychidae | TAS100 | *Derkarabetian *et al*. (2021) |
| *Odontonuncia saltuensis* | Triaenonychidae | WSU10007 | *Derkarabetian *et al*. (2021) |
| *Paradaeum* sp. | Triaenonychidae | MCZ73504 | *Baker *et al*. (2020) |
| *Paramontia* sp. | Triaenonychidae | MCZ:IZ:132878 | *Derkarabetian *et al*. (2021) |
| *Paranonychus brunneus* | Travunioidea: Paranonychidae | SDSU_TAC:OP3427 | *Derkarabetian *et al*. (2018) |
| *Paranonychus fuscus* | Travunioidea: Paranonychidae | SDSU_TAC:OP3966 | *Derkarabetian *et al*. (2018) |
| *Paranuncia gigantea* | Triaenonychidae | TAS242 | *Derkarabetian *et al*. (2019) |
| *Paranuncia ingens* | Triaenonychidae | MCZ:IZ:48421 | *Derkarabetian *et al*. (2021) |
| *Paranuncia* sp. | Triaenonychidae | MCZ:IZ:152660 | *Derkarabetian *et al*. (2021) |
| *Paulianyx* sp. | Triaenonychidae | CASENT9040819 | *Derkarabetian *et al*. (2021) |
| *Phanerobunus hebes* | Triaenonychidae | TAS021 | *Derkarabetian *et al*. (2021) |
| *Phoxobunus* sp. | Triaenonychidae | TAS323 | *Derkarabetian *et al*. (2021) |
| *Picunchenops spelaeus* | Triaenonychidae | OPI574 | - |
| *Picunchenops spelaeus* | Triaenonychidae | MCZ:IZ:152639 | *Derkarabetian *et al*. (2021) |
| *Planimontia goodnightorum* | Triaenonychidae | MCZ:IZ:133943 | *Derkarabetian *et al*. (2021) |
| *Planimontia* sp. | Triaenonychidae | MCZ73493_3 | *Baker *et al*. (2020) |
| *Planimontia* sp. | Triaenonychidae | MCZ134889 | *Baker *et al*. (2020) |
| *Prasma* sp. | Triaenonychidae | MCZ117475 | *Baker *et al*. (2020) |
| *Prasma* sp. | Triaenonychidae | MCZ133199 | *Baker *et al*. (2020) |
| *Prasma* sp. | Triaenonychidae | MCZ:IZ:133105 | *Derkarabetian *et al*. (2021) |
| *Prasma tuberculata mearosa* | Triaenonychidae | MCZ136190 | *Baker *et al*. (2020) |
| *Prasma tuberculata mulsa* | Triaenonychidae | SDSU_TAC:OP4613 | *Derkarabetian *et al*. (2021) |
| *Pristobunus acuminatus acantheis* | Triaenonychidae | MCZ136194 | *Baker *et al*. (2020) |
| *Pristobunus heterus* | Triaenonychidae | MCZ:IZ:133243 | *Derkarabetian *et al*. (2021) |
| *Pristobunus heterus* | Triaenonychidae | MCZ133351 | *Baker *et al*. (2020) |
| *Pristobunus* sp. | Triaenonychidae | MCZ:IZ:133422 | *Derkarabetian *et al*. (2019) |
| *Pseudobiantes japonica* | Grassatores: Epedanidae | SDSU_TAC:OP4292 | *Derkarabetian *et al*. (2018) |
| *Pyenganella striata* | Triaenonychidae | TAS386 | *Derkarabetian *et al*. (2021) |
| *Rhynchobunus arrogans* | Triaenonychidae | TAS145 | *Derkarabetian *et al*. (2021) |
| *Roewerania* sp. | Triaenonychidae | MCZ30076_2 | *Baker *et al*. (2020) |
| *Roewerania* sp. | Triaenonychidae | MCZ84507_2 | *Baker *et al*. (2020) |
| *Roewerania* sp. | Triaenonychidae | MCZ127275_2 | *Baker *et al*. (2020) |
| *Roewerania* sp. | Triaenonychidae | MCZ:IZ:86756 | *Derkarabetian *et al*. (2021) |
| *Rostromontia capensis* | Triaenonychidae | MCZ133944 | *Baker *et al*. (2020) |
| *Rostromontia* sp. | Triaenonychidae | MCZ132911 | *Baker *et al*. (2020) |
| *Rostromontia* sp. | Triaenonychidae | MCZ:IZ:148968 | *Derkarabetian *et al*. (2021) |
| *Sabacon cavicolens* | Dyspnoi | SDSU_TAC:OP1518 | *Starrett *et al*. (2017) |
| *Sclerobunus robustus* | Travunioidea: Paranonychidae | SDSU_TAC:OP1167 | *Derkarabetian *et al*. (2018) |
| *Siro boyerae* | Cyphophthalmi | SDSU_TAC:OP3383 | *Starrett *et al*. (2017) |
| *Sorensenella* sp. | Triaenonychidae | MCZ133429 | *Baker *et al*. (2020) |
| *Sorensenella* sp. | Triaenonychidae | MCZ:IZ:132849 | *Derkarabetian *et al*. (2021) |
| *Speleomaster lexi* | Travunioidea: Cryptomastridae | SDSU_TAC:OP1692 | *Derkarabetian *et al*. (2018) |
| *Speleomontia cavernicola* | Triaenonychidae | MCZ:IZ:132910 | *Derkarabetian *et al*. (2021) |
| *Speleonychia sengeri* | Travunioidea: Cladonychiidae | SDSU_TAC:OP1679 | *Derkarabetian *et al*. (2018) |
| *Synthetonychia* sp. | Synthetonychiidae | SDSU_TAC:OP4293 | *Derkarabetian *et al*. (2018) |
| *Synthetonychia* sp. | Synthetonychiidae | SDSU_TAC:OP4294 | *Derkarabetian *et al*. (2018) |
| *Tasmanobunus parvus* | Triaenonychidae | TAS288 | *Derkarabetian *et al*. (2021) |
| *Tasmanonyx montanus* | Triaenonychidae | TAS038 | *Derkarabetian *et al*. (2021) |
| *Thelbunus mirabilis* | Triaenonychidae | TAS337 | *Derkarabetian *et al*. (2021) |
| *Theromaster brunneus* | Travunioidea: Cladonychiidae | SDSU_TAC:OP1610 | *Derkarabetian *et al*. (2018) |
| *Travunia jandai* | Travunioidea: Travuniidae | SDSU_TAC:OP4617 | *Derkarabetian *et al*. (2018) |
| *Triaenobunus armstrongi* | Triaenonychidae | MCZ:IZ:58944 | *Derkarabetian *et al*. (2019) |
| *Triaenobunus armstrongi* | Triaenonychidae | MCZ58944 | *Baker *et al*. (2020) |
| *Triaenobunus armstrongi* | Triaenonychidae | MCZ134473 | *Baker *et al*. (2020) |
| *Triaenobunus asper* | Triaenonychidae | TAS451 | *Derkarabetian *et al*. (2021) |
| *Triaenobunus asper* | Triaenonychidae | WSU10016 | *Baker *et al*. (2020) |
| *Triaenobunus asper* | Triaenonychidae | WSU10021 | *Baker *et al*. (2020) |
| *Triaenobunus asper* | Triaenonychidae | WSU10070 | *Baker *et al*. (2020) |
| *Triaenobunus* cf *bicarinatus* | Triaenonychidae | WSU10006 | *Baker *et al*. (2020) |
| *Triaenobunus minutus* | Triaenonychidae | MCZ134220 | *Baker *et al*. (2020) |
| *Triaenobunus pectinatus* | Triaenonychidae | TAS011 | *Derkarabetian *et al*. (2021) |
| Triaenonychidae sp. | Triaenonychidae | MCZ35908 | *Baker *et al*. (2020) |
| Triaenonychidae sp. Australia | Triaenonychidae | SDSU_TAC:OP2779 | *Derkarabetian *et al*. (2021) |
| Triaenonychidae sp. Madagascar | Triaenonychidae | MCZ:IZ:52828 | *Derkarabetian *et al*. (2019) |
| *Triaenonychoides breviops* | Triaenonychidae | OPI033 | - |
| *Triaenonychoides breviops* | Triaenonychidae | MCZ:IZ:31332 | *Derkarabetian *et al*. (2019) |
| *Triaenonychoides cekalovici* | Triaenonychidae | OPI141 | - |
| *Triaenonychoides cekalovici* | Triaenonychidae | bold | - |
| *Triaenonyx rapax* | Triaenonychidae | OPI222 | - |
| *Triaenonyx* sp. | Triaenonychidae | MCZ138109 | *Baker *et al*. (2020) |
| *Triconobunus horridus* | Triaenonychidae | MCZ:IZ:151590 2 | *Derkarabetian *et al*. (2021) |
| *Triregia fairburni* | Triaenonychidae | MCZ:IZ:135963 | *Derkarabetian *et al*. (2021) |
| *Triregia fairburni grata* | Triaenonychidae | MCZ:IZ:135450 | *Derkarabetian *et al*. (2021) |
| *Trojanella serbica* | Travunioidea: Travuniidae | SDSU_TAC:OP2508 | *Derkarabetian *et al*. (2018) |
| *Turonychus fadriquei* | Buemarinoidae | MCZ:IZ:152705 | *Derkarabetian *et al*. (2021) |
| *Vonones sayi* | Grassatores: Cosmetidae | SDSU_TAC:OP3125 | *Derkarabetian *et al*. (2018) |
| *Yatala aspera* | Triaenonychidae | MCZ:IZ:23106 | *Derkarabetian *et al*. (2021) |
| *Yuria pulchra* | Travunioidea: incertae sedis | SDSU_TAC:OP4263 | *Derkarabetian *et al*. (2018) |
| *Zuma acuta* | Travunioidea: Paranonychidae | SDSU_TAC:OP3973 | *Derkarabetian *et al*. (2018) |
| *Zuma tioga* | Travunioidea: Paranonychidae | SDSU_TAC:OP1047 | *Derkarabetian *et al*. (2018) |
